# ProPhos: A Ligand for Promoting Nickel-Catalyzed Suzuki-Miyaura Coupling Inspired by Mechanistic Insights into Transmetalation

**DOI:** 10.1021/jacs.4c00370

**Published:** 2024-02-23

**Authors:** Jin Yang, Michelle C. Neary, Tianning Diao

**Affiliations:** † Department of Chemistry, New York University, 100 Washington Square East, New York, New York 10003, United States; ‡ Department of Chemistry, CUNY − Hunter College, 695 Park Ave, New York, New York 10065, United States

## Abstract

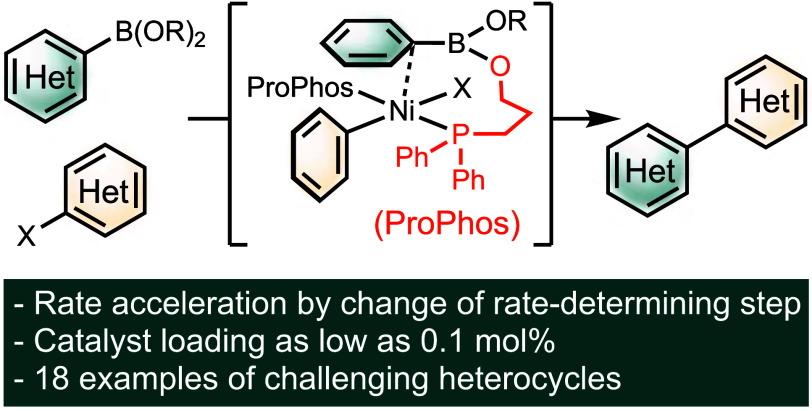

Nickel-catalyzed Suzuki–Miyaura coupling (Ni-SMC) offers the potential to reduce the cost of pharmaceutical process synthesis. However, its application has been restricted by challenges such as slow reaction rates, high catalyst loading, and a limited scope of heterocycles. Despite recent investigations, the mechanism of transmetalation in Ni-SMC, often viewed as the turnover-limiting step, remains insufficiently understood. We elucidate the “Ni-oxo” transmetalation pathway, applying PPh_2_Me as the ligand, and identify the formation of a nickel-oxo intermediate as the turnover-limiting step. Building on this insight, we develop a scaffolding ligand, ProPhos, featuring a pendant hydroxyl group connected to the phosphine via a linker. The design preorganizes both the nucleophile and the nickel catalyst, thereby facilitating transmetalation. This catalyst exhibits fast kinetics and robust activity across a wide range of heteroarenes, with a catalyst loading of 0.5–3 mol %. For arene substrates, the catalyst loading can be further reduced to 0.1 mol %.

## Introduction

**Scheme 1:**
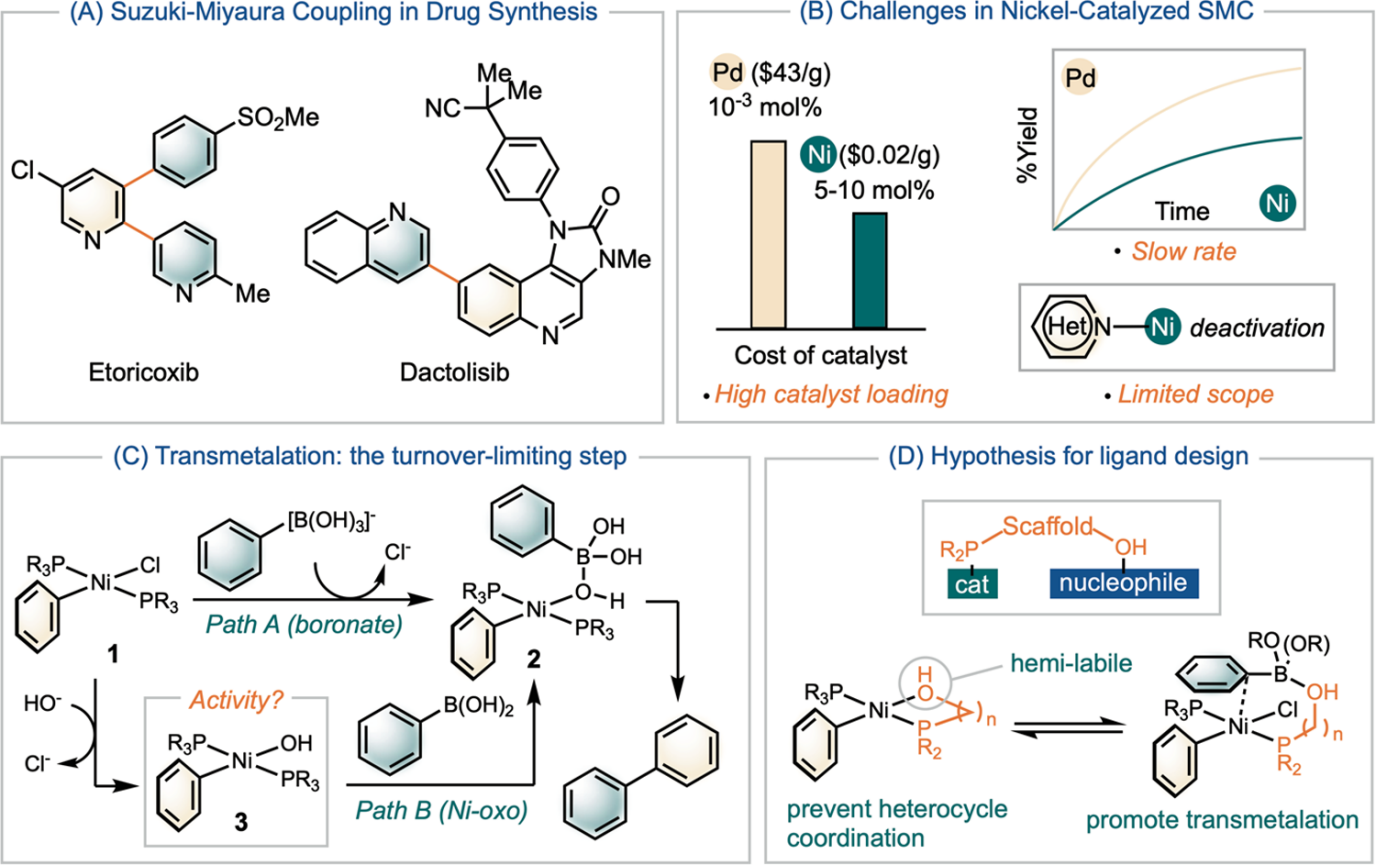
Suzuki–Miyaura Coupling (SMC) in the Synthesis of Pharmaceutical Products (A), Challenges Faced in Ni-SMC (B), Mechanistic Inquiries Regarding Transmetalation (C), and Proposed Scaffolding Ligand for Promoting Transmetalation (D)

Biaryl and heteroaryl motifs are prevalent in pharmaceutical products. The aromatic scaffolds provide a platform for the strategic spatial arrangement of substituents, imparting binding affinity and selectivity. Moreover, the aromatic and heteroaromatic rings themselves can engage in various noncovalent interactions with targets, such as π–π stacking, hydrogen bonding, and dipole interactions. The modern synthesis of aromatic frameworks often employs palladium-catalyzed Suzuki–Miyaura coupling (Pd-SMC) between aryl electrophiles, such as halides, and arylboron nucleophiles to construct Csp^2^–Csp^2^ bonds (Scheme 1A).^1,2^ The growing focus on sustainable pharmaceutical production with a minimal environmental impact, along with the motivation to reduce process costs, has led to an increase of interest in pursuing nonprecious metal alternatives to the Pd-SMC process.^3^ Nickel, belonging to the same group as palladium, emerges as a potential substitute.^4−10^ However, implementing Ni-SMC in process synthesis poses challenges, including the requirement for high catalyst loading (typically ranging from 5 to 10 mol %),^11^ which offsets the cost benefit, a slow reaction rate,^12^ and a limited scope of heteroarenes (Scheme 1B).^13,14^ The latter limitation may arise from potential catalyst poisoning through coordination.

The optimization of transition-metal-catalyzed reactions pivots crucially on ligand design. Recent efforts aimed at improving Ni-SMC have revealed that a monodentate phosphine ligand can facilitate oxidative addition and transmetalation, while a bidentate phosphine ligand can stabilize the catalyst against deactivation.^15^ This insight resonates with the recent application of Ph_2_MeP,^16^ Buchwald-type ligands,^17^ and dppb (dppb = 1,4-bis(diphenylphosphino)butane)^18^ in Ni-SMC. While data analysis and machine learning models emerge as powerful tools for identifying new ligands,^19^ a complementary strategy involves design based on mechanistic understanding. In this study, we demonstrate that the latter approach can lead to novel ligand frameworks that might otherwise evade discovery through the former method.

Prior mechanistic investigations determined that Ni-SMC operates through a Ni(0)/Ni(II) cycle,^20,21^ with transmetalation of arylboronic acid or ester nucleophiles to nickel identified as the turnover-limiting step, typically facilitated by a base.^22−24^ Generally, transmetalation can follow one of two possible pathways, distinguished by the role the base plays (Paths A and B, Scheme 1C).^25^ In Path A, the “boronate” mechanism, the base initially activates the boronic acid or ester by forming a boronate, which subsequently substitutes the halide on the nickel intermediate **1**, generating Ni–O–B intermediate **2**. In Path B, the “nickel-oxo” mechanism, the base first displaces the halide of **1**, resulting in the nickel-oxo intermediate **3**. Intermediate **3** is then associated with an arylboronic acid or ester to generate intermediate **2**. Studies on Pd-SMC have established Path B to be kinetically viable,^26^ and NMR characterization has verified the formation of a Pd–O–B intermediate as the pretransmetalation species.^27−29^ Regarding Ni-SMC, there remains ambiguity regarding the transmetalation pathway, particularly concerning the reactivity of nickel-oxo intermediates **3**.^30^ An investigation of the (PCy_3_)Ni catalyst supports Path B, suggesting that the formation of a nickel-oxo species before transmetalation represents the turnover-limiting step.^23^ Another study with a (PPh_3_)Ni catalyst implies the formation of a nickel-oxo dimer as an off-cycle species, suggesting that the catalytic reaction might be limited by the slow dissociation of the dimer prior to transmetalation.^24^ These varying proposals highlight the complexity of the transmetalation step in Ni-SMC, whose mechanisms may diverge depending on the specific catalysts and bases used.

In this work, we offer comprehensive insights into the transmetalation pathway using a Ni(PPh_2_Me) catalyst through kinetic and organometallic studies. We identify the formation of nickel-oxo intermediates as the turnover-limiting step and verify their fast reactivity in transmetalation. These findings led us to develop a phosphine scaffolding ligand featuring a tethered Lewis basic group designed to promote transmetalation (Scheme 1D). This ligand framework enables the colocation and preorganization of the catalyst and nucleophile, thus facilitating transmetalation in an intramolecular fashion.^31^ Moreover, the basic group could function as a hemilabile ligand, offering protection against catalyst poisoning through heteroatom coordination, while still readily dissociating to maintain high catalytic activity. This work not only unveils a highly reactive catalyst informed by a mechanistic hypothesis but also sets a course for ligand optimization, paving the way for the application of Ni-SMC in pharmaceutical process synthesis.

## Results and Discussion

### Ligand Design

**Figure 1:**
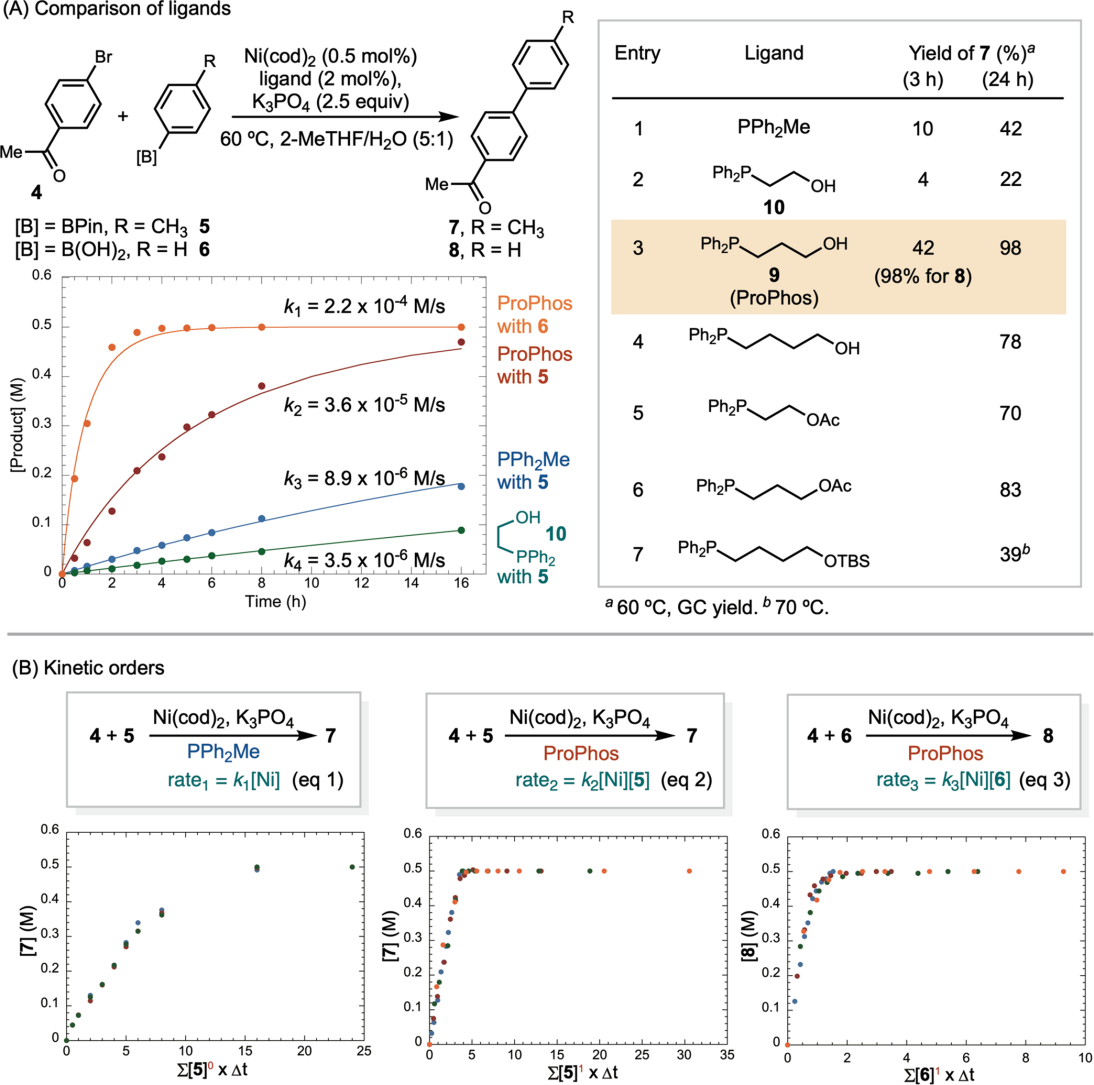
(A) Time-courses of Ni-SMC that reflect the effect of the ligand. Reaction conditions: [**4**]_0_ = 0.50 M, [**5**]_0_ or [**6**]_0_ = 0.55 M, reactions are monitored by GC with calibrations of the product. (B) Rate laws of Ni-SMC catalyzed by (Ph_2_MeP)Ni and (ProPhos)Ni catalysts.

Diphenylmethylphosphine (Ph_2_MeP) is currently recognized as one of the most effective catalysts in Ni-SMC.^16^ To develop a scaffolding ligand, we synthesized a series of ligands featuring hydroxyl, ester, and silyl ether groups tethered to diphenylphosphine through linkages of various lengths (Figure 1A). Our studies began with testing these ligands in a model SMC of **4** and **5** to afford **7** using a catalyst loading of 0.5 mol % at 60 °C. A comparison of their performances against Ph_2_MeP (entry 1, Figure 1A) after 24 h revealed that the ethanol-tethered phosphine ligand **10**, 2-(diphenylphosphino)ethanol, led to a reduced reactivity (entry 2). In contrast, ligands with longer linker tethered basic groups generally enhanced reactivity (entries 3–6). Among these ligands, the propanol-tethered phosphine ligand (ProPhos) **9**, 3-(diphenylphosphino)propanol, exhibits the highest activity (entry 3). Protecting the hydroxyl group with a *tert*-butyldimethylsilyl (TBS) group resulted in decreased reactivity (entry 7). An analysis at the 3 h time point indicated that ProPhos’s enhanced performance is attributed to an acceleration in reaction rate.

The kinetic analysis of the SMC further demonstrates the higher reactivity of ProPhos in comparison with Ph_2_MeP (Figure 1A). Fitting the time-course data to a first-order kinetic model resulted in *k*_obs_ for SMC of **4** and **5** catalyzed by **9**, **10**, and Ph_2_MeP. The rate with ProPhos **9** (*k*_2_) is higher than that with PPh_2_Me (*k*_3_) or 2-(diphenylphosphino)ethanol **10** (*k*_4_) by several folds. Applying ProPhos to SMC of **4** with **6** resulted in complete conversion to **8** within 3 h (*k*_1_).

**Figure 2:**
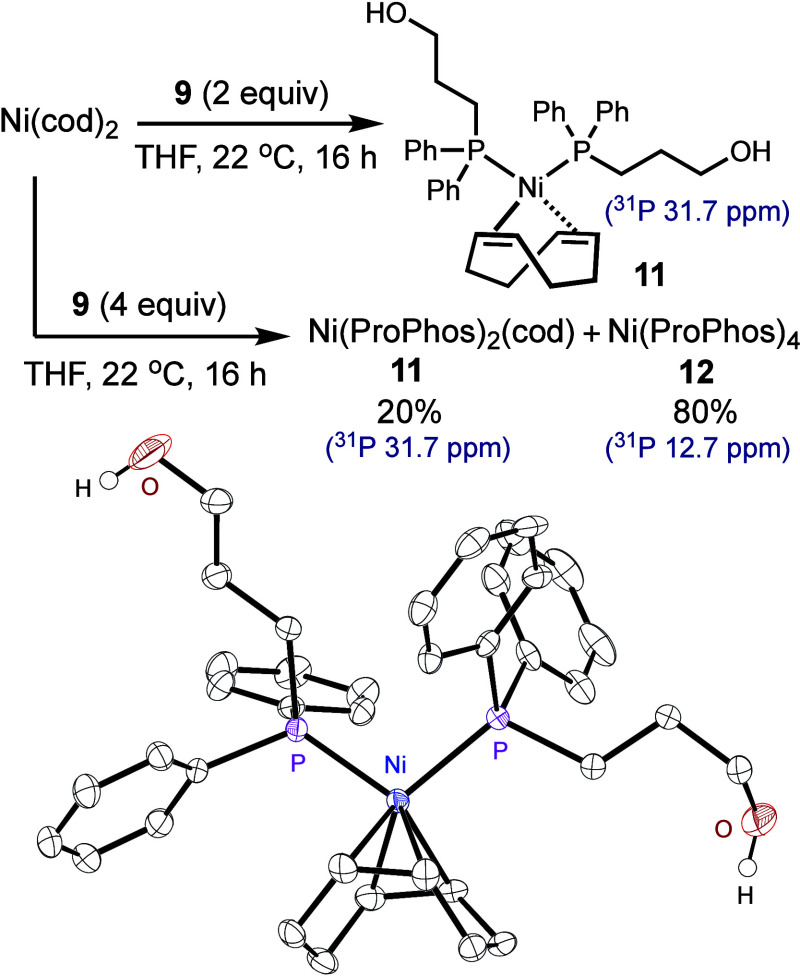
Synthesis and X-ray crystal structure of (ProPhos)Ni complexes (atomic displacement parameters at the 50% probability level). Hydrogen atoms bound to carbon have been omitted for clarity.

The excellent performance of ProPhos prompted us to investigate its coordination with nickel (Figure 2). Combining Ni(cod)_2_ with 2 equiv of ProPhos at room temperature afforded an orange crystal **11**. Analysis of **11** through single crystal X-ray diffraction and NMR spectroscopy revealed that ProPhos coordinates to nickel via the phosphine, while the hydroxyl group remains pendent and does not interact with nickel. In the presence of four equivalents of ProPhos, the reaction afforded a mixture of **11** and Ni(ProPhos)_4_**12**.

### Kinetics

To probe the mechanistic attributes for the faster rate with ProPhos **9**, we determined and compared the kinetic orders of the substrates and the catalysts using Variable Time Normalization Analysis (VTNA).^32^ With PPh_2_Me as the ligand, the reaction exhibited first-order dependence on the nickel catalyst and is independent of both [**4**] and [**5**] (Figure 1B). In contrast, with ProPhos **9**, the rate displayed first-order dependence on both the catalyst and the nucleophile, [**5**] or [**6**].

### Catalyst Resting State

**Figure 3:**
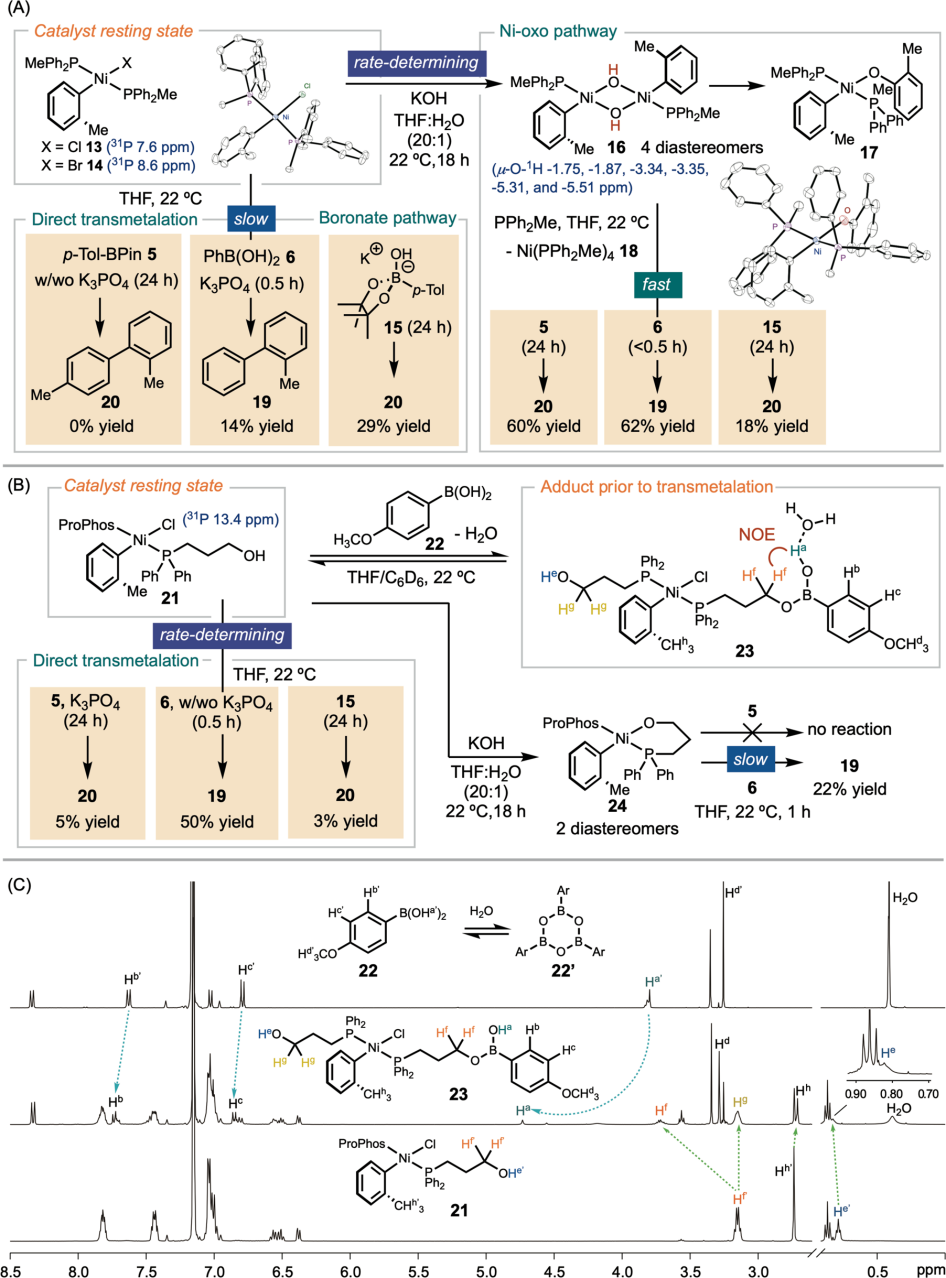
Organometallic studies of transmetalation in Ni-SMC.

Subsequently, we determined the catalyst resting state in the (PPh_2_Me)Ni-catalyzed SMC of *o-*tolyl bromide and **5** by monitoring the reaction using ^31^P NMR spectroscopy. During the reaction, we observed a ^31^P NMR resonance at 8.6 ppm (Figure S57). Independently, we prepared complexes (PPh_2_Me)_2_Ni(*o*-Tol)Cl **13** and (PPh_2_Me)_2_Ni(*o*-Tol)Br **14** through oxidative addition of Ni(PPh_2_Me)_4_**18** to *o-*Tol chloride and bromide, respectively. By comparing the ^31^P NMR signals, we infer that the catalyst resting state in the (PPh_2_Me)Ni-catalyzed SMC is likely (PPh_2_Me)_2_Ni(*o*-Tol)Br **14** (Figures 3A and S57).

Synergistically, we monitored the (ProPhos)Ni-catalyzed SMC of *o*-Tol chloride with **5** using ^31^P NMR spectroscopy for a direct comparison. The reaction mixture displayed a ^31^P NMR signal at 13.4 ppm, representing the major catalyst species in the resting state (Figure S59). We synthesized Ni(ProPhos)_2_(*o*-Tol)Cl **21** through ligand exchange of Ni(TMEDA)Cl(*o*-Tol) (TMEDA = tetramethylethylenediamine)^33,34^ with ProPhos **9**. Ni(ProPhos)_2_(*o*-Tol)Cl **21** displayed a characteristic ^31^P NMR resonance at 13.4 ppm, which is consistent with the major signal observed during the catalytic reaction. Thus, we attribute the resting state in the (ProPhos)Ni-catalyzed SMC to **21** (Figures 3B and S60).

### Organometallic Studies

In our following organometallic studies, we investigated the transmetalation reactivity of Ni(PPh_2_Me)_2_(o-Tol)Cl **13** and Ni(ProPhos)_2_(*o*-Tol)Cl **21** with boronic acid **6**, ester **5**, and boronate **15** (Figure 3).^35^ The formation of boronate **15** from **5** and KOH was a slow process, requiring heating at 70 °C for 16 h. No reaction occurred between **13** and **5**, regardless of whether K_3_PO_4_ was present. Additionally, the reactions of **13** with both PhB(OH)_2_**6** and boronate **15** were slow, forming **19** and **20**, respectively, but in low yields (Figure 3A).

Subjecting **13** to KOH resulted in the formation of **16**, appearing as a mixture of four diastereomers (Figures S39–S41). The ^1^H signals of the OH groups were identified at −1.75, −1.87, −3.34, –3.35, −5.31, and −5.51 ppm, diagnostic for *cis-* and *trans*-μ-O-dimers, respectively, as previously observed experimentally^23^ and verified computationally.^24^ The identity of **16** was further confirmed by HRMS. An analysis of the ^1^H/^31^P{^1^H}-HMBC spectra allowed us to assign the resonances for each diastereomer (Figure S39). Although the coexistence of diastereomers has complicated our attempts to obtain single-crystal structural characterization, **16** underwent C–O bond-forming reductive elimination to form **17**, whose structure was elucidated via X-ray crystallography. In contrast to nickel(dihydroxide)^6^ or nickel(dialkoxide),^36^ which have been reported to exhibit no reactivity toward nucleophiles, complex **16** reacted rapidly with **5** and **6**, to produce **19** and **20**, respectively (Figure S83). The reaction of **16** with **15** is slightly slower compared to the reaction of **13** with **15**. The modest yields were attributed to complications arising from the comproportionation of **16** with *in situ* generated **18**. In comparing the relative rates of stoichiometric reactions, we labeled the steps in kinetically slow pathways as “slow”, the rate-limiting step in the productive pathway as “rate-determining”, and the rapid processes within the productive pathway as “fast” (Figure 3).

We investigated the transmetalation reactivity of **21** (Figure 3B). Subjecting **21** to either **5** or **15** led to no significant change in the ^1^H and ^31^P NMR spectra. Over 16 h at room temperature, the reaction mixture yielded only trace amounts of **20**, regardless the presence or absence of K_3_PO_4_. However, combining **21** with PhB(OH)_2_**6** resulted in the rapid formation of **19**, even without K_3_PO_4_ (Figure S92); K_3_PO_4_ further accelerated the reaction. Upon treating **21** with 4-OMe-C_6_H_4_B(OH)_2_**22**, we observed the formation of a new species **23**. The ^1^H NMR spectrum of **22** displays a resonance at 3.81 ppm, attributed to the OH (H^a^), and AA’XX’ aromatic resonances at 7.63 and 6.79 ppm (H^b^ and H^c^) (Figure 3C). In **23**, H^a^ shifts downfield to 4.74 ppm, accompanied by minor downfield shifts of H^b^ and H^c^ signals to 7.73 and 6.85 ppm, respectively. A new methyl signal emerges at 3.30 ppm (H^d^), which is slightly more upfield compared to the methoxy signal in **22**.

In a comparison of the spectra of **23** with that of **21** (Figure 3C), we observed that the OH signal of ProPhos (H^e^) underwent a slight downfield shift from 0.79 to 0.84 ppm. Moreover, the resonance at 3.20 ppm, corresponding to the α-H of the alcohol (H^f′^), split into two signals at 3.75 (H^f^) and 3.20 (H^g^) ppm. Additionally, the signal at 2.72 ppm, corresponding to the methyl group on the *o-*Tol ligand, shifted upfield to 2.71 ppm (H^h^).

Analysis of the ^1^H NMR spectra led us to assign the series of new resonances to the nickel boronic adduct **23** formed from the association of **21** with **22** followed by the elimination of a water molecule. The connectivity of the H signals of **23** was further verified by ^1^H COSY and ^1^H/^31^P{^1^H}-HMBC (Figures S64 and S65). To further substantiate the spatial correlation between the nickel catalyst and **22**, we conducted ^1^H-NOESY experiments (*cf*. Figure S66). The spectra unambiguously established a correlation between H^a^ at 4.74 ppm and H^f^ at 3.75 ppm, supporting the bonding connectivity between ProPhos and the boronic acid. The loss of a water molecule and the formation of a three-coordinate boronic ester was substantiated by the ^11^B NMR signal at 29.3 ppm (Figure S67).^28,35^ At higher temperature, the equilibrium favors the formation of aryl boroxine **22**′, which drives the hydrolysis of **23** (Figure S75). Integration of peaks assigned to **21** and **23** allowed us to estimate the equilibrium constant (*K*) for the association of **21** with **22** to be approximately 1 at room temperature.

Subsequently, we probed the effect of strong bases on the speciation of the nickel catalyst and transmetalation. Addition of KOH to **21** led to dissociation of chloride and formation of **24** as a mixture of two diastereomers, in which the deprotonated alcohol reaches around to chelate on nickel. However, **24** is inactive with **5**, and it reacted with PhB(OH)_2_**6** slowly compared to **21**, giving **19** in 22% yield over 1 h (Figure S79).

### Proposed Mechanisms

**Scheme 2:**
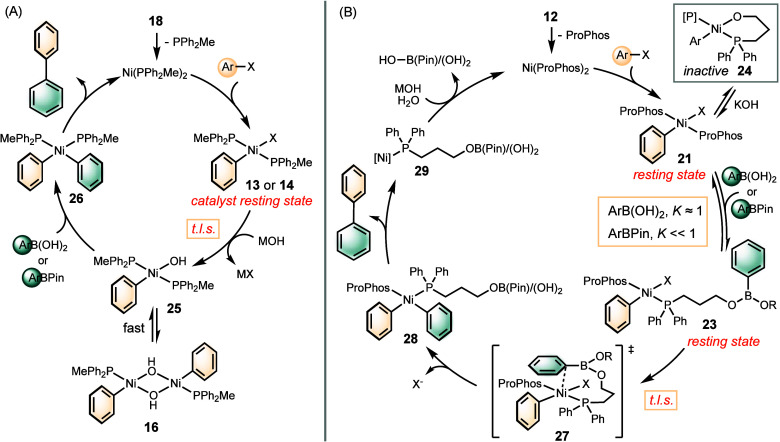
Mechanisms of Ni(PPh_2_Me) and Ni(ProPhos)-catalyzed SMC

Collectively, our data suggest two distinct scenarios for SMC catalyzed by Ni(PPh_2_Me) and Ni(ProPhos), respectively (Scheme 2). The mechanism of reactions facilitated by PPh_2_Me follows a classic “nickel-oxo” pathway (Scheme 2A). The rate law (eq 1, Figure 1B), with a first-order dependence on [Ni] and no dependence on either substrate, aligns with a turnover-limiting step involving the formation of the Ni–OH species **25**, consistent with previous proposals.^23^ This proposal is further supported by the observation of **14** as the catalyst’s resting state. Our stoichiometric studies reveal that the nickel-oxo intermediate **25** is stabilized by forming the *μ-*oxo dimer **16**.^37,38^ In these experiments, we observed a rapid transmetelation of **16** with boronic acids and esters leading to a reductive elimination product via formation of intermediate **26** (Figure 3A). The fast consumption of **16** suggests that the dissociation of **16** to **25** is rapid. In contrast, the slow reaction of **13** with boronate **15**, coupled with the even slower formation of boronate **15** from **5** and a base, suggests that the “boronate pathway” is not kinetically competent. Instead, it is plausible that the hydroxide from **15** might displace the halide on nickel, leading to the formation of the nickel-hydroxide intermediate **25** prior to transmetalation.^39^

The application of ProPhos led to a different pathway (Scheme 2B). The rate laws (eqs 2 and 3, Figure 1B) indicate that the boron nucleophile is involved during or before the turnover-limiting step. Characterization of the catalyst resting species and organometallic studies suggest that the coordination of the boron nucleophile to the pendant hydroxyl group of ProPhos in **21** to form **23** is indeed a critical pre-equilibrium prior to transmetalation. When ArBPin is used as the nucleophile, the equilibrium predominantly favors dissociation (*K* ≪ 1). In comparison, when ArB(OH)_2_ serves as the nucleophile, it exhibits a more favorable coordination to ProPhos (*K* ≈ 1), resulting in the faster catalytic rate of **6** relative to **5** (Figure 1A). The Lewis acidity of ArBPin is greater than that of ArB(OH)_2_, due to the tendency of boron to rehybridization from sp^2^ to sp^3^ to reduce the angle strain.^40^ The difference in equilibrium can be attributed to the steric hindrance of ArBPin and the ability of ArB(OH)_2_ to lose a molecule of water to form nickel boronic ester **23** upon the coordination of ProPhos. NMR experiments characterized the formation of a nickel intermediate, assigned to adduct **23** formed between **21** and ArB(OH)_2_. The Nuclear Overhauser Effect (NOE) between ProPhos and the boronic acid substantiates the association between these two species. In Ni(ProPhos)-catalyzed SMC, the formation of nickel-oxo intermediate **25** is not necessary. It is noteworthy that strong bases, such as KOH, can inhibit the reaction by fully deprotonating ProPhos, leading to the formation of cyclized species **24**. In this context, the formation of the nickel-alkoxy species is detrimental. The transmetalation of **23** to form **28** is expected to be the turnover-limiting step, proceeding via the formation of intramolecular transition state **27**.

Comparing the mechanisms of SMC catalyzed by Ni(PPh_2_Me) and Ni(ProPhos) sheds light on the mechanistic attributes for the rate acceleration observed with Ni(ProPhos) compared to Ni(PPh_2_Me). With PPh_2_Me as the ligand, the turnover rate depends on the formation of the nickel-oxo intermediate through ligand exchange of nickel halide with hydroxide, a step enhanced by a stronger base. In contrast, with PhoPhos, the formation of a nickel-oxo intermediate is not essential for transmetalation. The pendant hydroxyl group in ProPhos can coordinate to boronic acids and esters, directing the approach of the nucleophile and facilitating transmetalation without the need for a base. While a weak base is necessary for catalytic turnover, a strong base could inhibit the reaction by deprotonating ProPhos and forming **24**. This ligand-based mechanism alteration and the consequent change in turnover-limiting steps present a strategic alternative to the empirical ligand screening approach. The nucleophilicity of the tethered directing group determines the turnover rate and unveils avenues for further optimization.

### Synthetic Application of ProPhos

**Scheme 3:**
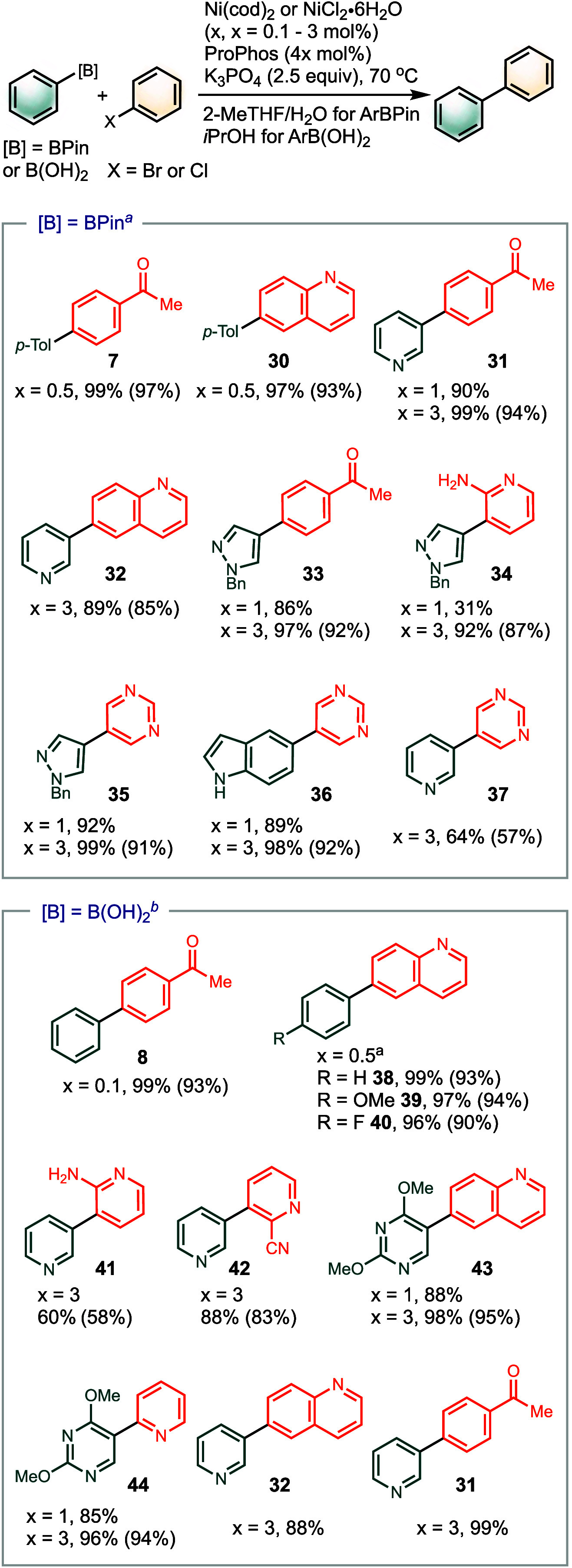
Scope of Ni(ProPhos)-Catalyzed SMC Conditions: Ni(cod)_2_ (x mol %), ProPhos (4x mol %), K_3_PO_4_ (2.5 equiv), 2-MeTHF/H_2_O (5:1), 16 h. NiCl_2_·6H_2_O (*x* mol %), ProPhos (4*x* mol %), K_3_PO_4_ (2.5 equiv), *i*PrOH, 16 h. % Yield determined by GC with calibrations and isolated % yield in the parentheses.

We evaluated the performance of ProPhos in Ni-SMC with respect to its compatibility with heterocycles, which are typically challenging substrates (Scheme 3).^16,41^ Without extensive catalyst optimization, we employed a mixed solvent system consisting of 2-MeTHF/H_2_O for ArBPin and *i*PrOH for ArB(OH)_2_. Notably, NiCl_2_·6H_2_O proved to be effective in *i*PrOH with a range of boronic acids, yielding the desired products. In pharmaceutical process synthesis, replacing Ni(cod)_2_ with an air-stable and cost-effective nickel precursor, such as NiCl_2_·6H_2_O, is highly desirable for large-scale applications.

With a catalyst loading of 0.5–1 mol %, a variety of substrates containing pyridine, quinoline, pyrazole, pyrimidine, and 2-aminopyridine underwent SMC, forming products in high yields. In cases of lower yields, increasing the catalyst loading to 3 mol % was sufficient to improve the performance. Five-membered heterocycles are uncommon examples in Ni-SMC. Pyrazole derivatives **33**–**35** proved to be compatible with the (ProPhos)Ni catalyst. Moreover, **34** and **41**, featuring the typically challenging unprotected 2-amino pyridine, were synthesized with excellent yields using (ProPhos)Ni. Compound **41** was previously unattainable with nickel catalysts and requiring a 6 mol % loading of palladium.^42^ Additionally, an indole product **36** required no protection by using the (ProPhos)Ni catalyst. Finally, we challenged ProPhos with a catalyst loading as low as 0.1 mol %. Under this condition, the SMC of **4** with **6** proceeded to give a quantitative yield of **8** in 16 h.

## Conclusion

Transmetalation plays a vital role in determining the turnover rate and the scope of Ni-SMC. We have elucidated that the formation of a nickel-oxo intermediate is the turnover-limiting step for (PPh_2_Me)Ni-catalyzed SMC. These insights informed us in designing the ProPhos scaffolding ligand, which alters the turnover-limiting step to transmetalation from a precoordinated intermediate formed between the ligand’s pendant hydroxyl group and boronic acids and esters. This ligand-substrate interaction enables faster catalytic turnover rates by directing the nucleophile toward the nickel center. The (ProPhos)Ni catalyst has demonstrated efficiency in SMC across a broad range of heteroarenes with a catalyst loading of 0.5–3 mol %. In the case of arene substrates, the Ni-SMC can operate at catalyst loadings as low as 0.1 mol %. The strategy of introducing scaffolding ligands to preorganize the nucleophile and catalyst represents a novel avenue for optimizing Ni-SMC toward pharmaceutical process production.

## Supporting Information Available

Full experimental procedures, characterization data, and NMR spectra (PDF)
