# Supplementary material for: ProPhos: A Ligand for Promoting Nickel-Catalyzed Suzuki-Miyaura Coupling Inspired by Mechanistic Insights into Transmetalation
Source: J Am Chem Soc. 2024 Feb 23;146(9):6360–8. doi: 10.1021/jacs.4c00370 (PMC10921396; doi:10.1021/jacs.4c00370)
Supplement: Supplementary file 1 — ja4c00370_si_001.pdf [file ja4c00370_si_001.pdf]

Supporting information

ProPhos: A Ligand for Promoting Nickel-Catalyzed Suzuki-Miyaura Coupling Inspired by Mechanistic Insights into Transmetalation

Jin Yang,[1] Michelle C. Neary,[2] and Tianning Diao[1]*

[1]Department of Chemistry, New York University, 100 Washington Square East, New York, NY 10003
*E-Mail: diao@nyu.edu

[2]Department of Chemistry, CUNY – Hunter College, 695 Park Ave, New York, NY 10065, United States

Table of contents

1.General comments 4
2. Synthesis and catalytic activity of phosphine ligands 5
2.1 Synthesis of $PPh_2CH_2CH_2CH_2OH$ (ProPhos, **9**) 5
2.2 Synthesis of $PPh_2CH_2CH_2OH$ (**10**) 5
2.3 Synthesis of $PPh_2CH_2CH_2CH_2CH_2OH$ (**S1**) 6
2.4 Synthesis of $PPh_2CH_2CH_2C(O)Me$ (**S2**) 6
2.5 Synthesis of $PPh_2CH_2CH_2C(O)OMe$ (**S3**) 7
2.6 Synthesis of $PPh_2CH_2CH_2OC(O)Me$ (**S4**) 7
2.7 Isolation of $PPh_2CH_2CH_2OCH_2CH_2OH$ (**S5**) 8
2.8 Synthesis of $PPh_2CH_2CH_2CH_2OC(O)Me$ (**S6**) 9
2.9 Synthesis of $PPh_2CH_2CH_2CH_2CH_2OSiMe_2Bu^t$ (**S8**) 9
2.10 NMR spectra of isolated phosphines 11
2.11 Evaluation of phosphine ligands on Ni-SMC 39
3. Kinetics experiments 42
3.1 General procedure for kinetic experiments 42
3.2 Variable Time Normalization Analysis (VTNA) results 43
3.3 Effect of potential hydrolysis of B(*p*-Tol)Pin (**5**) during catalysis 50
4. Synthesis of Ni complexes 51
4.1 Synthesis of $Ni(PPh_2CH_2CH_2CH_2OH)_2(cod)$ (**11**) 51
4.2 Attempted synthesis of $Ni(PPh_2CH_2CH_2CH_2OH)_4$ (**12**) 51
4.3 Synthesis of $Ni(PPh_2Me)_2Cl$(*o*-Tol) (**13**) 53
4.4 Synthesis of $Ni(PPh_2Me)_2Br$(*o*-Tol) (**14**) 54
4.5 Synthesis of $[Ni(PPh_2Me)(\mu\text{-}OH)(o\text{-}Tol)]_2$ (**16**) 55
4.6 Synthesis of $Ni(PPh_2Me)_4$ (**18**) 60
4.7 Synthesis of Ni(TMEDA)Cl(*o*-Tol) (**S9**) 60
4.8 Synthesis of $Ni(PPh_2CH_2CH_2CH_2OH)_2Cl$(*o*-Tol) (**21**) 61
4.9 NMR spectra of isolatable Ni complexes 62
5. Organometallic study of Ni phosphine complexes 76
5.1 Resting state analysis of the catalytic systems using PPh_2Me or ProPhos as a ligand 76
5.1.1 Reaction of 2-bromotoluene and B(*p*-Tol)Pin (**5**) using $Ni(cod)_2$ and PPh_2Me 77
5.1.2 Reaction of 2-bromotoluene and $BPh(OH)_2$ (**6**) using $Ni(cod)_2$ and PPh_2Me 78
5.1.3 Reaction of 2-chlorotoluene and B(*p*-Tol)Pin (**5**) using $Ni(cod)_2$ and ProPhos 79
5.1.4 Reaction of 2-bromotoluene and B(*p*-Tol)Pin (**5**) using $Ni(cod)_2$ and ProPhos 81
5.1.5 Reaction of 2-bromotoluene and $BPh(OH)_2$ (**6**) using $Ni(cod)_2$ and ProPhos 82
5.2 Study of B,O-coordination between complex $Ni(PPh_2CH_2CH_2CH_2OH)_2Cl$(*o*-Tol) (**21**) with boronic acids and esters 82

5.2.1 Reaction of complex **21** with 4-OMe-PhB$(OH)_2$ (**22**)... 82

5.2.2 Reaction of complex **21** with BPh$(OH)_2$ (**6**) 87

5.2.3 Reactions of complex **21** with B(*p*-Tol)Pin (**5**) 89

5.2.4 Reactions of complex Ni$(PPh_2Me)_2$Cl(*o*-Tol) (**13**) with boronic acids and esters 90

5.2.5 Variable temperature NMR study of ProPhos (**9**) with 4-OMe-PhB$(OH)_2$ (**22**)..................... 92

5.3 Reactivity studies of complex Ni$(PPh_2CH_2CH_2CH_2OH)_2$Cl(*o*-Tol) (**21**) with KOH....................... 94

5.3.1 Reaction of complex **21** with KOH in THF/H_2O 94

5.3.2 Reactivity of the mixture containing Ni(κ^2-$PPh_2CH_2CH_2CH_2O$)(*o*-Tol)(ProPhos) (**24**) with B(*p*-Tol)Pin (**5**) or BPh$(OH)_2$ (**6**) 97

5.4 Transmetalation studies of Ni-PPh_2Me complexes (**13** and **16**) and Ni-ProPhos complex (**21**). 97

5.4.1 Synthesis of K[B(*p*-Tol)Pin(OH)] (**15**) 98

5.4.2 Reactions of complex Ni$(PPh_2Me)_2$Cl(*o*-Tol) (**13**) with B(*p*-Tol)Pin (**5**), BPh$(OH)_2$ (**6**) and K[B(*p*-Tol)Pin(OH)] (**15**)... 101

5.4.3 Reactions of complex [Ni(PPh_2Me)(μ-OH)(*o*-Tol)$]_2$ (**16**) with B(*p*-Tol)Pin (**5**), BPh$(OH)_2$ (**6**) and K[B(*p*-Tol)Pin(OH)] (**15**)... 102

5.4.4 The reaction of **13** with **6** and bases.. 106

5.4.5 Reactions of complexes **13** and **16** with K[B(*p*-Tol)Pin(OH)] (**15**) in the presence of 18-crown-6.. 108

5.4.6 Comparison of rates of relevant reactions in the Ni-PPh_2Me transmetalation.................. 109

5.4.7 Reactions of complex Ni$(PPh_2CH_2CH_2CH_2OH)_2$Cl(*o*-Tol) (**21**) with B(*p*-Tol)Pin (**5**), BPh$(OH)_2$ (**6**) and K[B(*p*-Tol)Pin(OH)] (**15**)... 112

5.4.8 The reaction of **21** with **6** and bases.. 114

5.4.9 Role of the base in the Ni-ProPhos catalytic system.. 117

6. Substrate study using ProPhos **9** 119

6.1 General procedure for determining the yield of the biaryl compounds.................................... 119

6.2 Characterization data for Ni-SMC products.. 121

6.3 NMR spectra for Ni-SMC products 129

7. Crystallographic data.. 164

1.General comments

All air- and moisture-sensitive reactions and manipulations were performed under nitrogen in a glovebox or using conventional Schlenk techniques. Common solvents (e.g., THF) were dried and deoxygenated by passing through alumina in a solvent purification system. Deuterated solvents were purchased from commercial sources. Deuterated solvents used in the glovebox (C_6D_6 and C_7D_8) were degassed using three freeze-pump-thaw cycles and stored over activated molecular sieve for 3 days before use. Unless otherwise specified, reagents were purchased from commercial sources. $Ni(acac)_2$, $Ni(cod)_2$, $NiCl_2•6H_2O$, and PPh_2Me (neat, 97%) were purchased from commercial sources. Other phosphine ligands and organometallic compounds were synthesized following the procedures presented in Section 2 and 4, respectively.

NMR spectra were recorded on a Bruker Avance 400 spectrometer (400.30 MHz for ^1H, 162.04 MHz for ^{31}P and 100.67 MHz for ^{13}C), and a Bruker Avance 500 spectrometer (500.20 MHz for ^1H and 202.49 MHz for ^{31}P). Unless otherwise noted, chemical shifts are reported in ppm at RT. ^1H chemical shifts are referenced to residual proteo-solvent peak at 7.26 ppm ($CHCl_3$), 7.16 ppm (C_6D_5H), 4.79 ppm (DHO), 2.50 ppm ($(CD_2H)S(O)(CD_3)$) and 2.08 ppm (C_7D_7H); ^{13}C chemical shifts are referenced to 128.1 ppm (C_6D_6),77.1 ppm ($CDCl_3$), 29.8 ppm ($(CD_3)_2SO$) and 20.4 ppm (C_7D_8). ^1H and ^{13}C chemical shifts are reported relative to tetramethylsilane (TMS), and ^{31}P chemical shifts are reported relative to 85% H_3PO_4 (aq).

High resolution mass spectra (HRMS) were recorded on an Agilent 6224 TOF LC/MS (APCI source). GC data were obtained using a Shimadzu GC-2010 with a Shimadzu SH-Rxi-5Sil MS column. GC data for kinetic experiments and %yield determination was obtained using a Shimadzu GC-2010 Plus with a Restek Rxi-5MS column (L 15 m, ID 0.25, DF 0.25).

2. Synthesis and catalytic activity of phosphine ligands

All phosphine syntheses were conducted using conventional Schlenk techniques. Characterization details for new phosphine ligands $PPh_2CH_2CH_2CH_2OC(O)Me$ (**S6**) and $PPh_2CH_2CH_2CH_2CH_2OSiMe_2Bu^t$ (**S8**) are provided in sections 2.8 & 2.9. All phosphines were dried under vacuum before use.

2.1 Synthesis of $PPh_2CH_2CH_2CH_2OH$ (ProPhos, 9)

To a solution of diphenylphosphine (2.00 g, 10.7 mmol, 1.0 equiv) and 3-chloropropanol (1.02 g, 10.7 mmol, 1.0 equiv) in THF (20 mL) was added *n*-butyl lithium (10.7 mL, 2.00 M in hexanes, 21.5 mmol, 2.0 equiv) dropwisely at 0 °C. The mixture was stirred for 1 h at room temperature (rt). Water (10 mL) was added, and the mixture was evaporated, and the residue was extracted in CH_2Cl_2 (2 x 10 mL). The suspension was filtered, and the solution evaporated. The remaining residue was further purified via column chromatography (hexane:CH_2Cl_2 1:1→CH_2Cl_2:EtOAc 7:3) to yield the product (2.06 g, 8.43 mmol, 78%) as a white solid. The NMR data is consistent with literature reports.[1]

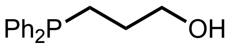

9

^{1}H NMR (400.30 MHz, $CDCl_3$) δ: 7.44 (ddt, *J* = 7.5, 5.4, 2.6 Hz, 4H, H_o), 7.37 – 7.31 (m, 6H, H_m&H_p), 3.72 (t, *J* = 6.4 Hz, 2H, CH_2O), 2.18 – 2.11 (m, 2H, PCH_2), 1.79 – 1.60 (m, 3H, CH_2 & OH, OH signal is broad and overlapped with CH_2 signals).

^{31}P{^{1}H} NMR (162.04 MHz, $CDCl_3$) δ: -15.5 (s).

^{13}C{^{1}H} NMR (100.67 MHz, $CDCl_3$) δ: 138.1 (d, *J* = 11Hz, C_{ipso}), 132.9 (d, *J* = 18 Hz, C_o), 128.9 (s, C_p), 128.6 (d, *J* = 7 Hz, C_m), 63.7 (d, *J* = 13 Hz, CH_2O), 29.1 (d, *J* = 15 Hz, PCH_2), 24.3 (d, *J* = 10 Hz, CH_2).

MS (ESI-TOF, CH_3CN) m/z: $[M + H]^+$ calculated for $C_{15}H_{18}OP$ 245.11, found 245.11.

2.2 Synthesis of $PPh_2CH_2CH_2OH$ (10)

To a solution of diphenylphosphine (1.00 g, 5.37 mmol, 1.0 equiv) and 2-chloroethanol (0.432 g, 5.37 mmol, 1.0 equiv) in THF (20 mL) was added *n*-butyl lithium (5.91 mL, 2.00 M in hexanes, 11.8 mmol, 2.2 equiv). The mixture was stirred for 3 h at 0 °C. The THF was evaporated, and the residue was extracted in CH_2Cl_2 (2 x 20 mL). The resulting solution was treated with an excess of solid NH_4Cl in CH_2Cl_2. The suspension was filtered, and the solution was evaporated. The remaining residue was extracted with Et_2O (2 x 15 mL), and the volatiles were removed. The crude residue was further purified via column chromatography (hexane:CH_2Cl_2 1:1→CH_2Cl_2:EtOAc 7:3) to yield the product (0.605 g, 2.63 mmol, 24%) as a colorless oil. The NMR data is consistent with literature reports.[1]

---

[1] Grasset, F. L.; Welter, R.; Braunstein, P.; Olivier-Bourbigou, H.; Magna, L. Titanium Complexes with Functional Alkoxido Ligands for Selective Ethylene Dimerization – A High Throughput Experimentation Approach. *ChemCatChem* **2021**, *13* (9), 2167–2178.

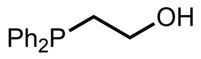

10

^{1}H NMR (400.30 MHz, $CDCl_3$) δ: 7.44 (ddt, J = 7.5, 5.3, 2.4 Hz, 4H, H_o), 7.37 – 7.30 (m, 6H, H_m&H_p), 3.80 (dtd, J = 9.6, 7.2, 5.5 Hz, 2H, CH_2O), 2.40 (t, J = 7.2 Hz, 2H, PCH_2), 1.54 (s, 1H, OH).

$^{31}P\{^{1}H\}$ NMR (162.04 MHz, $CDCl_3$) δ: -24.1 (s).

$^{13}C\{^{1}H\}$ NMR (100.67 MHz, $CDCl_3$) δ: 138.1 (d, J = 12 Hz, C_{ipso}), 132.9 (d, J = 19 Hz, C_o), 128.9 (s, C_p), 128.7 (d, J = 7 Hz, C_m), 60.4 (d, J = 22 Hz, CH_2O), 32.4 (d, J = 13 Hz, PCH_2).

MS (ESI-TOF, CH_3OH) m/z: $[M + H]^+$ calculated for $C_{14}H_{15}OP$ 231.09, found 231.09.

2.3 Synthesis of $PPh_2CH_2CH_2CH_2CH_2OH$ (S1)

To a solution of diphenylphosphine (0.943 g, 5.07 mmol, 1.0 equiv) and 4-chlorobutanol (0.550 g, 5.07 mmol, 85% mixed with HCl, 1.0 equiv) in THF (20 mL) was added *n*-butyl lithium (5.57 mL, 2.00 M in hexanes, 11.1 mmol, 2.2 equiv) dropwisely at 0 °C. The mixture was stirred for 3 h at rt. Water (10 mL) was added, and the mixture was evaporated, and the residue was extracted in CH_2Cl_2 (2 x 10 mL). The suspension was filtered, and the solution evaporated. The remaining residue was further purified via column chromatography (hexane:CH_2Cl_2 1:1→CH_2Cl_2:EtOAc 7:3) to yield the product (0.811 g, 3.14 mmol, 62%) as a colourless oil. The NMR data is consistent with literature reports.[1]

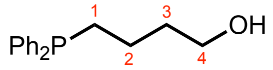

S1

^{1}H NMR (400.30 MHz, $CDCl_3$) δ: 7.43 (tt, J = 7.2, 2.0 Hz, 4H, H_o), 7.37 – 7.28 (m, 6H, H_m&H_p), 3.61 (t, J = 6.5 Hz, 2H, H4), 2.11 – 2.05 (m, 2H, H1), 1.79 – 1.65 (m, 3H, H3&OH, OH signal is broad and overlapped with CH_2 signals), 1.58 – 1.47 (m, 2H, H2).

$^{31}P\{^{1}H\}$ NMR (162.04 MHz, $CDCl_3$) δ: -16.3 (s).

$^{13}C\{^{1}H\}$ NMR (100.67 MHz, $CDCl_3$) δ: 138.8 (d, J = 13 Hz, C_{ipso}), 132.8 (d, J = 18 Hz, C_o), 128.6 (s, C_p), 128.5 (d, J = 7 Hz, C_m), 62.4 (s, C4), 34.2 (d, J = 12 Hz, C1), 27.9 (d, J = 11 Hz, C2), 22.4 (d, J = 17 Hz, C3).

MS (ESI-TOF, CH_3CN) m/z: $[M + H]^+$ calculated for $C_{16}H_{20}OP$ 259.12, found 259.12.

2.4 Synthesis of $PPh_2CH_2CH_2C(O)Me$ (S2)

To a mixture of neat diphenylphosphine (0.833 g, 4.48 mmol, 1.0 equiv) and but-3-en-2-one (0.377 g, 5.37 mmol, 1.2 equiv), 2-MeTHF (1.81 mL, 17.9 mmol, 4.0 equiv) was added. The mixture was stirred for 16 h at rt. The mixture was evaporated, and the residue was extracted in EtOAc (2 x 2 mL). The suspension was filtered, and the solution evaporated. The remaining residue was then purified by

flash silica gel chromatography using a gradient from hexane to hexane:EtOAc 8:2 to obtain the product (0.850 g, 3.32 mmol, 74%) as a colorless oil. The NMR data is consistent with literature reports.[2]

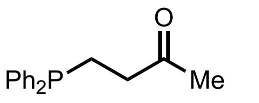

S2

^{1}H NMR (400.30 MHz, C_6D_6) δ: 7.46 – 7.32 (m, 4H, H_o), 7.13 – 6.98 (m, 6H, H_m&H_p), 2.32 – 2.23 (m, 2H, CH_2CO), 2.19 – 2.07 (m, 2H, PCH_2), 1.50 (s, 3H, $COCH_3$).

^{31}P{^{1}H} NMR (162.04 MHz, C_6D_6) δ: -15.7 (s).

^{13}C{^{1}H} NMR (100.67 MHz, C_6D_6) δ: 205.2 (d, *J* = 12 Hz, C=O), 139.2 (d, *J* = 14 Hz, C_{ipso}), 133.1 (d, *J* = 19 Hz, C_o), 128.9 (s, C_p), 128.8 (d, *J* = 7 Hz, C_m), 39.6 (d, *J* = 18 Hz, $\underline{C}H_2CO$), 29.1 (s, $CO\underline{C}H_3$), 21.8 (d, *J* = 12 Hz, PCH_2).

MS (ESI-TOF, CH_3CN) m/z: $[M + H]^+$ calculated for $C_{16}H_{18}OP$ 257.11, found 257.11.

2.5 Synthesis of $PPh_2CH_2CH_2C(O)OMe$ (S3)

To neat diphenylphosphine (0.909 g, 4.88 mmol, 1.0 equiv) and methyl acrylate (0.462 g, 5.37 mmol, 1.1 equiv) was added. The mixture was stirred for 16 h at rt. The mixture was evaporated under vacuum to remove extra methyl acrylate. The crude product was obtained (1.12 g, 4.08 mmol, 84%) as a colorless oil, with 94% purity determined by NMR (6% consisting of the branched product **S3'**). It was used without further purification. The crude product could be purified by flash silica gel chromatography using a gradient from hexane to hexane:EtOAc 8:2, yielding the pure linear product. The NMR data is consistent with literature reports.[2]

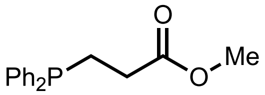

S3

^{1}H NMR (400.30 MHz, C_6D_6) δ: 7.40 – 7.31 (m, 4H, H_o), 7.08 – 6.99 (m, 6H, H_m&H_p), 3.27 (s, 3H, CH_3), 2.34 – 2.26 (m, 4H, CH_2).

^{31}P{^{1}H} NMR (162.04 MHz, C_6D_6) δ: -15.9 (s).

^{13}C{^{1}H} NMR (100.67 MHz, C_6D_6) δ: 172.9 (d, *J* = 15 Hz, C=O), 138.8 (d, *J* = 14 Hz, C_{ipso}), 133.1 (d, *J* = 19 Hz, C_o), 128.9 (s, C_p), 128.8 (d, *J* = 7 Hz, C_m), 51.2 (s, CH_3), 30.8 (d, *J* = 20 Hz, $\underline{C}H_2CO$), 23.6 (d, *J* = 13 Hz, PCH_2).

MS (ESI-TOF, CH_3CN) m/z: $[M + H]^+$ calculated for $C_{16}H_{18}O_2P$ 273.10, found 273.11.

2.6 Synthesis of $PPh_2CH_2CH_2OC(O)Me$ (S4)

To a solution of diphenylphosphine (1.20 g, 6.46 mmol, 1.0 equiv) and 3-chloropropanol (0.520 g, 6.46 mmol, 1.0 equiv) in THF (20 mL) was added *n*-butyl lithium (6.5 mL, 2.0 M in hexanes, 12.9

[2] Bissessar, D.; Egly, J.; Achard, T.; Steffanut, P.; Bellemin-Laponnaz, S. Catalyst-Free Hydrophosphination of Alkenes in Presence of 2-Methyltetrahydrofuran: A Green and Easy Access to a Wide Range of Tertiary Phosphines. *RSC Adv.* **2019**, *9* (47), 27250–27256.

mmol, 2.0 equiv). The mixture was stirred for 3 h at 0 °C. The THF was evaporated, and the residue was treated with EtOAc and stirred for 1 h. The suspension was extracted with water. The organic layer was evaporated. The crude residue was further purified via column chromatography (hexane:EtOAc 9:1→ hexane:EtOAc 7/3) to yield the product (0.628 g, 2.31 mmol, 36%) as a colorless oil.

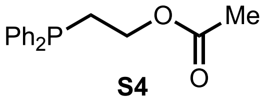

S4

^{1}H NMR (400.30 MHz, CDCl$_3$) δ: 7.45 (tt, *J* = 7.5, 2.2 Hz, 4H, H_o), 7.38 – 7.30 (m, 6H, H_m&H_p), 4.27 – 4.16 (m, 2H, CH_2O), 2.44 (dd, *J* = 8.0, 7.1 Hz, 2H, PCH_2), 1.95 (s, 3H, CH_3).

^{31}P{^{1}H} NMR (162.04 MHz, CDCl$_3$) δ: -22.4 (s).

^{13}C{^{1}H} NMR (100.67 MHz, CDCl$_3$) δ: 171.0 (s, C=O), 137.7 (d, *J* = 12 Hz, C_{ipso}), 132.8 (d, *J* = 19 Hz, C_o), 129.0 (s, C_p), 128.7 (d, *J* = 7 Hz, C_m), 62.2 (d, *J* = 24 Hz, CH_2O), 28.0 (d, *J* = 14 Hz, PCH_2), 21.0 (s, CH_3).

MS (ESI-TOF, CH$_3$CN) m/z: $[M + H]^+$ calcd for $C_{16}H_{18}O_2P$ 273.10, found 273.10.

This compound can be synthesized using another literature procedure, and our NMR data for **S4** is consistent with the literature reports.[3] The literature also reports the thermal decomposition of this phosphine to produce *s*-ethylenebis(diphenylphosphine). A proposed mechanism suggests that these decompositions occur through the initial formation of a 1,1-diphenylphosphoniacyclopropane salt intermediate, which formed due to the anchimeric effect of the trivalent phosphorus atom. We observed the rapid formation of a brown solid from a large amount of neat phosphine at rt. Thus, a small amount of phosphine is placed in a small vial and stored in the freezer, which slows the thermal decomposition.

2.7 Isolation of PPh$_2$CH$_2$CH$_2$OCH$_2$CH$_2$OH (S5)

This compound is isolated from the reaction mixture of synthesis **S4**. The crude residue was further purified via column chromatography (hexane:EtOAc 3:1) to yield the product (50.0 mg) as a colorless oil. The NMR data is consistent with the literature reports.[4]

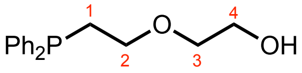

S5

^{1}H NMR (400.30 MHz, CDCl$_3$) δ: 7.44 (ddt, *J* = 7.6, 5.5, 2.8 Hz, 4H, H_o), 7.34 (ddt, *J* = 5.6, 2.3, 1.4 Hz, 6H, H_m&H_p), 3.89 – 3.84 (m, 2H, H3), 3.80 (dt, *J* = 9.7, 7.2 Hz, 2H, H2), 3.69 – 3.64 (m, 2H, H4), 2.41 (t, *J* = 7.2 Hz, 2H, H1), 2.02 (br s, 1H, OH).

^{31}P{^{1}H} NMR (162.04 MHz, CDCl$_3$) δ: -23.6 (s).

---

[3] Turner, R. W.; Soloway, A. H. The Thermal Stability of (2-Substituted Ethyl)Diphenylphosphines. The Potent Neighboring Group Effect of the Trivalent Phosphorus Atom1. *J. Org. Chem.* **1965**, *30* (12), 4031–4034.

[4] Ipaktschi, J.; Sulzbach, W. Synthese Und Charakterisierung von Hydridotrispyrazolylborat-Heterobimetall-Komplexen Mit Titan Und Molybdän. *J. Organomet. Chem.* **1992**, *434* (3), 287–302.

$^{13}C\{^1H\}$ NMR (100.67 MHz, $CDCl_3$) δ: 137.6 (d, J = 12 Hz, C_{ipso}), 132.9 (d, J = 19 Hz, C_o), 129.0 (s, C_p), 128.7 (d, J = 7 Hz, C_m), 63.1 (s, C3), 60.2 (d, J = 21.6 Hz, C2), 47.1 (s, C4), 32.3 (d, J = 12 Hz, C1).

MS (ESI-TOF, CH_3OH) m/z: $[M + H]^+$ calcd for $C_{16}H_{20}O_2P$ 275.12, found 275.11.

2.8 Synthesis of $PPh_2CH_2CH_2CH_2OC(O)Me$ (S6)

To a solution of diphenylphosphine (2.00 g, 10.7 mmol, 1.0 equiv) and 3-chloropropanol (1.02 g, 10.7 mmol, 1.0 equiv) in THF (20 mL) was added *n*-butyl lithium (10.7 mL, 2.0 M in hexanes, 21.5 mmol, 2.0 equiv). The mixture was stirred for 3 h at 0 °C. The THF was evaporated, and the residue was treated with EtOAc and then stirred for 1 h. The suspension was extracted by H_2O. The organic layer was evaporated. The crude residue was further purified via column chromatography (hexane:EtOAc 9:1→ hexane:EtOAc 7:3) to yield the product (1.80 g, 6.30 mmol, 59%) as a colorless oil.

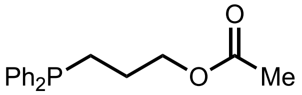

S6

^1H NMR (400.30 MHz, $CDCl_3$) δ: 7.46 – 7.40 (m, 4H, H_o), 7.39 – 7.29 (m, 6H, H_m&H_p), 4.12 (t, J = 6.5 Hz, 2H, CH_2O), 2.13 – 2.07 (m, 2H, PCH_2), 2.04 (s, 3H, CH_3), 1.82 – 1.70 (m, 2H, CH_2).

$^{31}P\{^1H\}$ NMR (162.04 MHz, $CDCl_3$) δ: -16.5 (s).

$^{13}C\{^1H\}$ NMR (100.67 MHz, $CDCl_3$) δ: 171.1 (s, C=O), 138.4 (d, J = 13 Hz, C_{ipso}), 132.8 (d, J = 19 Hz, C_o), 128.8 (s, C_p), 128.6 (d, J = 7 Hz, C_m), 65.0 (d, J = 14 Hz, CH_2O), 25.4 (d, J = 17 Hz, PCH_2), 24.5 (d, J = 12 Hz, CH_2), 21.1 (s, CH_3).

HRMS (ESI-TOF, CH_3CN) m/z: $[M + H]^+$ calcd for $C_{17}H_{19}O_2P$ 287.1195, found 287.1171.

2.9 Synthesis of $PPh_2CH_2CH_2CH_2CH_2OSiMe_2Bu^t$ (S8)

*Synthesis of $ClCH_2CH_2CH_2CH_2OSiMe_2Bu^t$ (**S7**)*

In a round-bottomed flask, solid *tert*-butyldimethylsilyl chloride (2.15 g, 14.3 mmol, 1.0 equiv) was dissolved in dry CH_2Cl_2 (50 mL) at 0 °C using an ice bath. 4-Chlorobutanol (1.55 g, 14.3 mmol, 85% mixed with HCl, 1.0 equiv) was added dropwise to the reaction mixture, and stirred at 0 °C for 30 min. After that, solid imidazole (1.12 g, 16.4 mmol, 1.2 equiv) was added to the flask and further stirred at 0 °C for 1.5 h. The reaction mixture was filtered through a frit, and the filtrate was concentrated under vacuum, and the resulting translucent oil was stored at -35 °C overnight. Hexanes (30 mL) was then added, and the suspension was passed through a Celite/$MgSO_4$ plug over a fine frit. The filtrate was concentrated under reduced pressure to give a colorless, transparent oil (2.65 g, 11.9 mmol, 83%). This compound is commercially available, and can be prepared by the literature procedure.[5]

[5] Roach, J. J.; Sasano, Y.; Schmid, C. L.; Zaidi, S.; Katritch, V.; Stevens, R. C.; Bohn, L. M.; Shenvi, R. A. Dynamic Strategic Bond Analysis Yields a Ten-Step Synthesis of 20-nor-Salvinorin A, a Potent κ-OR Agonist. *ACS Cent. Sci.* **2017**, *3* (12), 1329–1336.

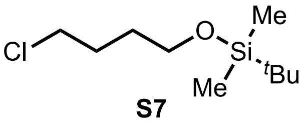

S7

^{1}H NMR (400.30 MHz, $CDCl_3$) δ: 3.64 (t, *J* = 6.1 Hz, 2H, OCH_2), 3.57 (t, *J* = 6.7 Hz, 2H, $ClCH_2$), 1.85 (ddt, *J* = 9.0, 8.2, 6.5 Hz, 2H, CH_2), 1.70 – 1.62 (m, 2H, CH_2), 0.89 (s, 9H, Bu^t), 0.05 (s, 6H, CH_3). The ^{1}H data is comparable with the literature report.[5]

To a solution of diphenylphosphine (2.03 g, 10.9 mmol, 1.0 equiv) and (4-chlorobutoxy)(1,1-dimethylethyl)dimethyl-silane (**S7**, 2.42 g, 10.9 mmol, 1.0 equiv) in THF (20 mL) was added *n*-butyl lithium (5.5 mL, 2.00 M in hexanes, 11.0 mmol, 1 equiv) dropwisely at 0 °C. The mixture was stirred for 3 h at rt. Water (10 mL) was added, and the mixture was evaporated, and the residue was extracted in CH_2Cl_2 (2 x 10 mL). The suspension was filtered, and the solution was evaporated. The remaining residue was further purified via column chromatography (hexane:CH_2Cl_2 1:1→CH_2Cl_2:EtOAc 7:3) to yield the product (1.81 g, 4.83 mmol, 45%) as a colorless oil.

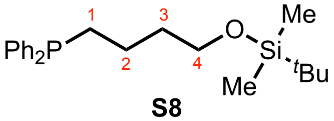

S8

^{1}H NMR (400.30 MHz, $CDCl_3$) δ: 7.43 (ddt, *J* = 7.3, 5.2, 2.8 Hz, 4H, H_o), 7.38 – 7.29 (m, 6H, H_m&H_p), 3.60 (t, *J* = 6.3 Hz, 2H, H4), 2.12 – 2.01 (m, 2H, H1), 1.65 (p, *J* = 6.6 Hz, 2H, H3), 1.56 – 1.45 (m, 2H, H2), 0.86 (s, 9H, Bu^t), 0.02 (s, 6H, CH_3).

^{31}P{^{1}H} NMR (162.04 MHz, $CDCl_3$) δ: -16.0 (s).

^{13}C{^{1}H} NMR (100.67 MHz, $CDCl_3$) δ: 139.1 (d, *J* = 13 Hz, C_{ipso}), 132.9 (d, *J* = 18 Hz, C_o), 128.6 (s, C_p), 128.5 (d, *J* = 7 Hz, C_m), 62.8 (s, C4), 34.4 (d, *J* = 13 Hz, d, C1), 28.0 (d, *J* = 11 Hz, C2), 26.1 (s, $SiC(CH_3)_3$), 22.5 (d, *J* = 17 Hz, C3), 18.4 (s, $Si\mathit{C}(CH_3)_3$), -5.2 (s, $SiCH_3$).

HRMS (ESI-TOF, CH_3CN) m/z: $[M + H]^+$ calculated for $C_{22}H_{34}OPSi$ 373.2111, found 373.2134.

2.10 NMR spectra of isolated phosphines

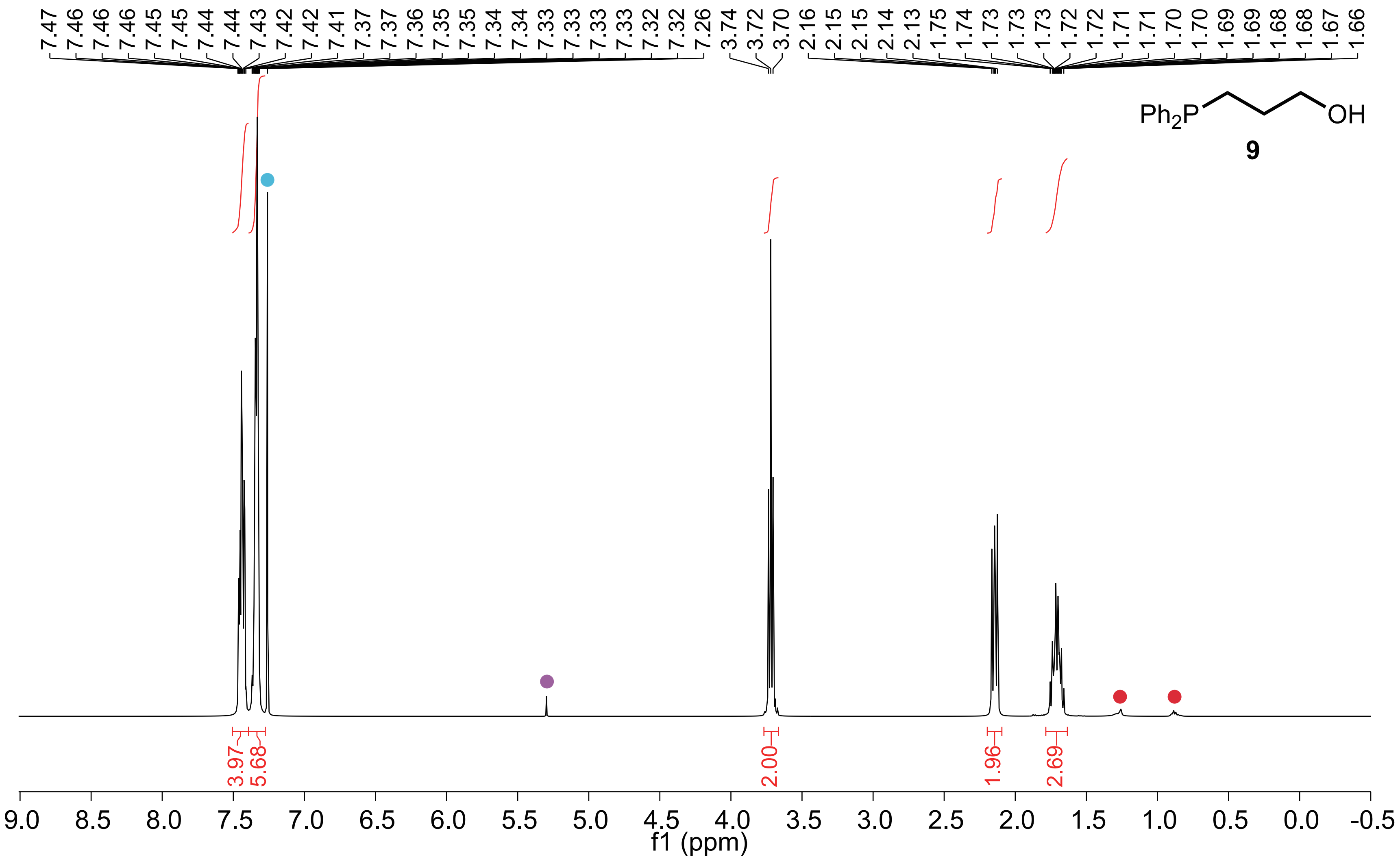

Figure S1 ^{1}H NMR (400.30 MHz, $CDCl_3$) spectrum of $PPh_2CH_2CH_2CH_2OH$ (**9**). Residual proteo-solvent (•), CH_2Cl_2 (•) and hexane (•).

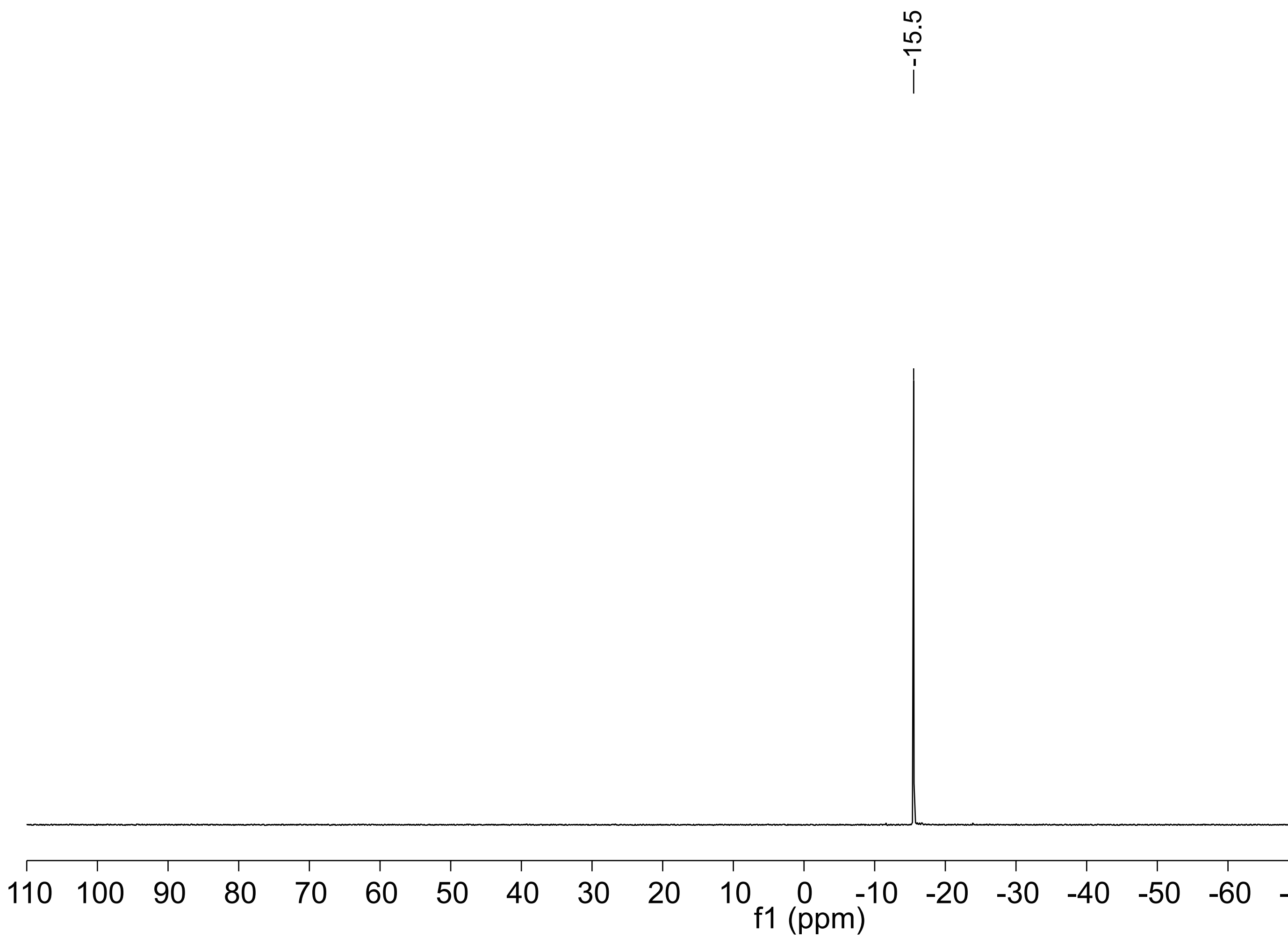

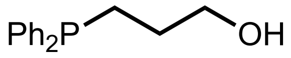

9

Figure S2 $^{31}P\{^{1}H\}$ NMR (162.04 MHz, $CDCl_3$) spectrum of $PPh_2CH_2CH_2CH_2OH$ (**9**).

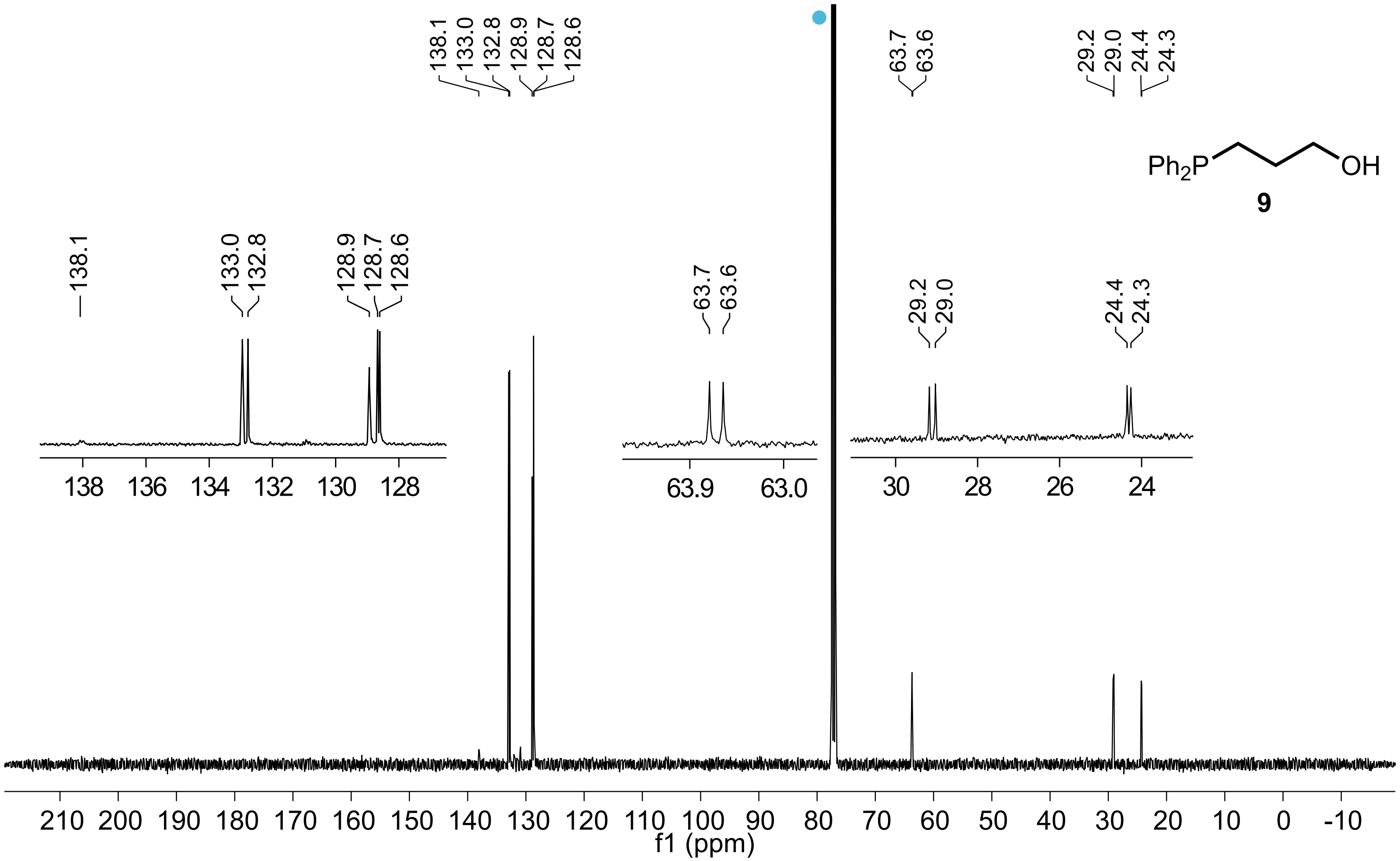

Figure S3 $^{13}C\{^1H\}$ NMR (100.67 MHz, $CDCl_3$) spectrum of $PPh_2CH_2CH_2CH_2OH$ (**9**). Deuterated solvent (•).

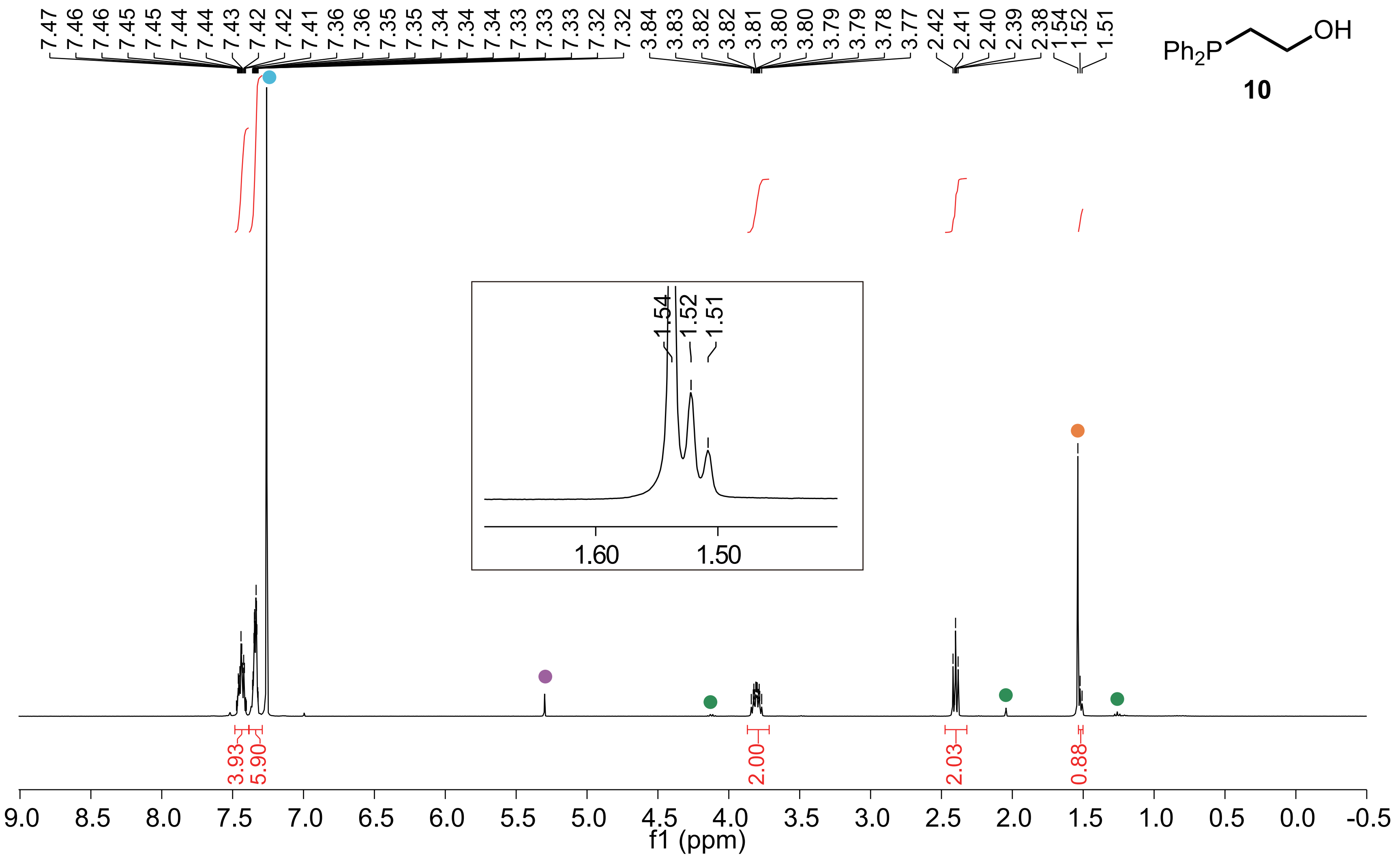

Figure S4 ^{1}H NMR (400.30 MHz, $CDCl_3$) spectrum of $PPh_2CH_2CH_2OH$ (**10**). Residual proteo-solvent (●), CH_2Cl_2 (●), H_2O (●) and EtOAc (●). Inset shows the signal due to OH, which is overlapped with H_2O (●).

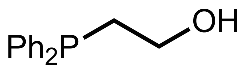

10

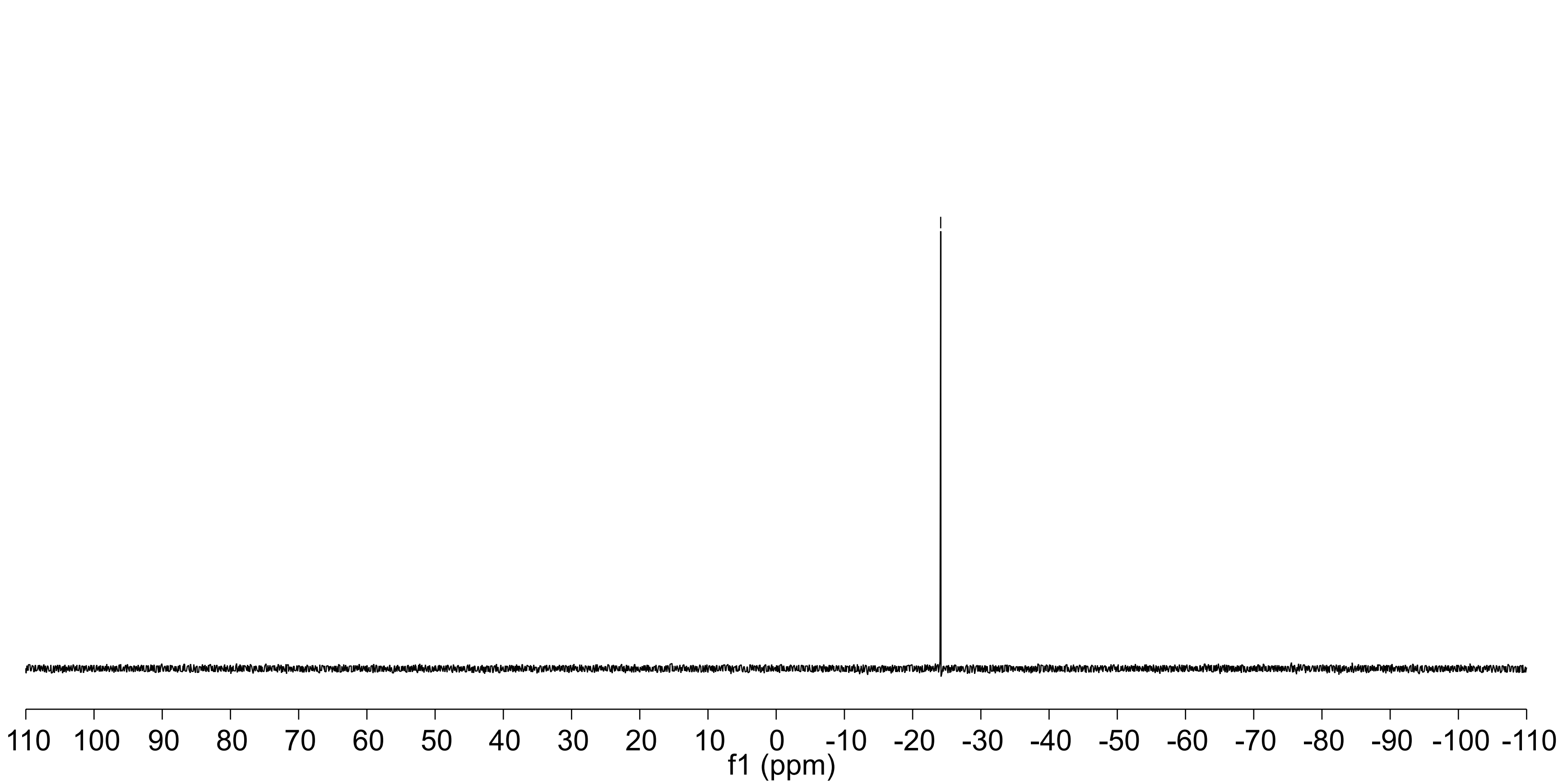

Figure S5 $^{31}P\{^{1}H\}$ NMR (162.04 MHz, $CDCl_3$) spectrum of $PPh_2CH_2CH_2OH$ (**10**).

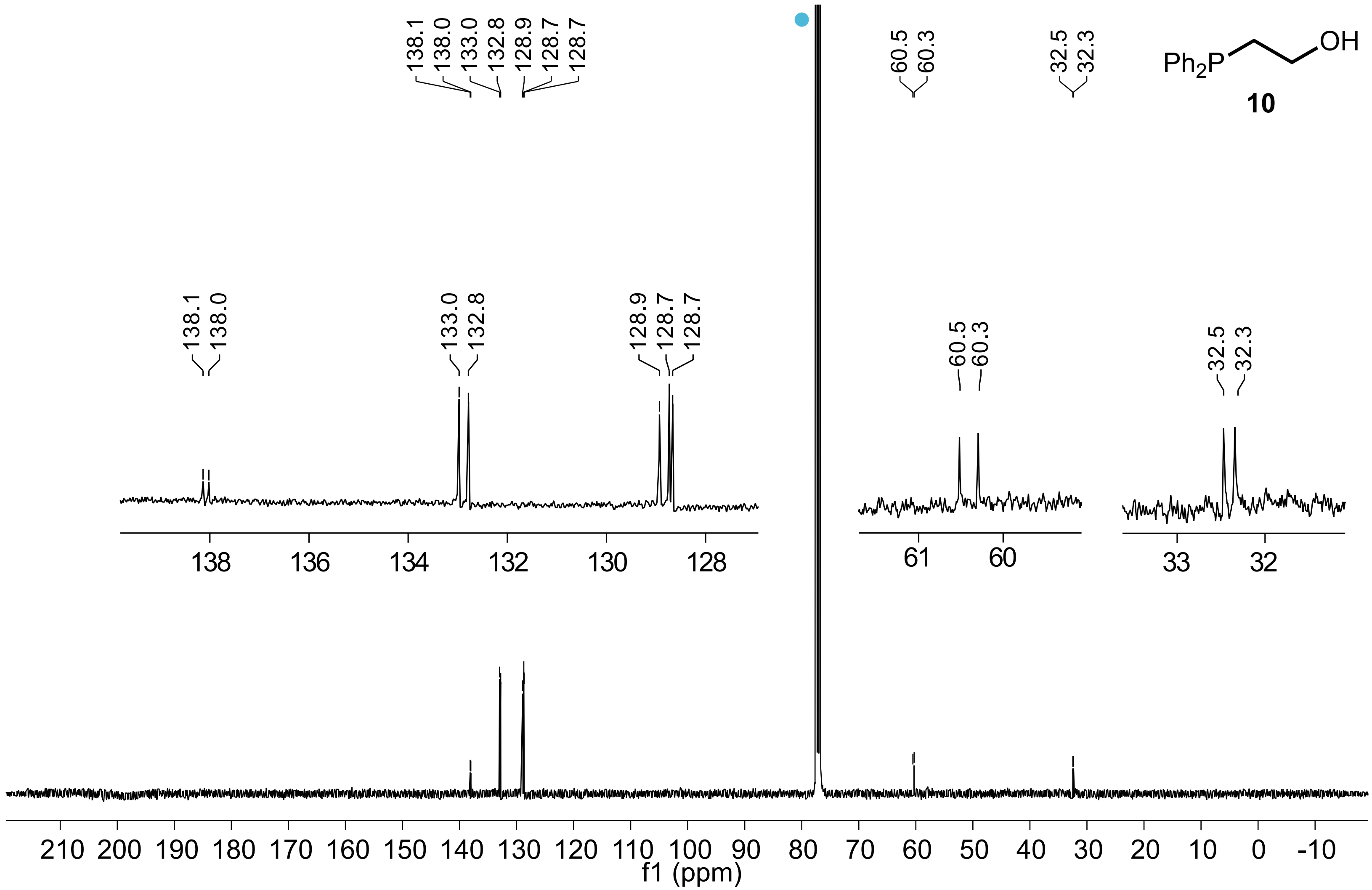

Figure S6 $^{13}C\{^1H\}$ NMR (100.67 MHz, $CDCl_3$) spectrum of $PPh_2CH_2CH_2OH$ (**10**). Deuterated solvent (•).

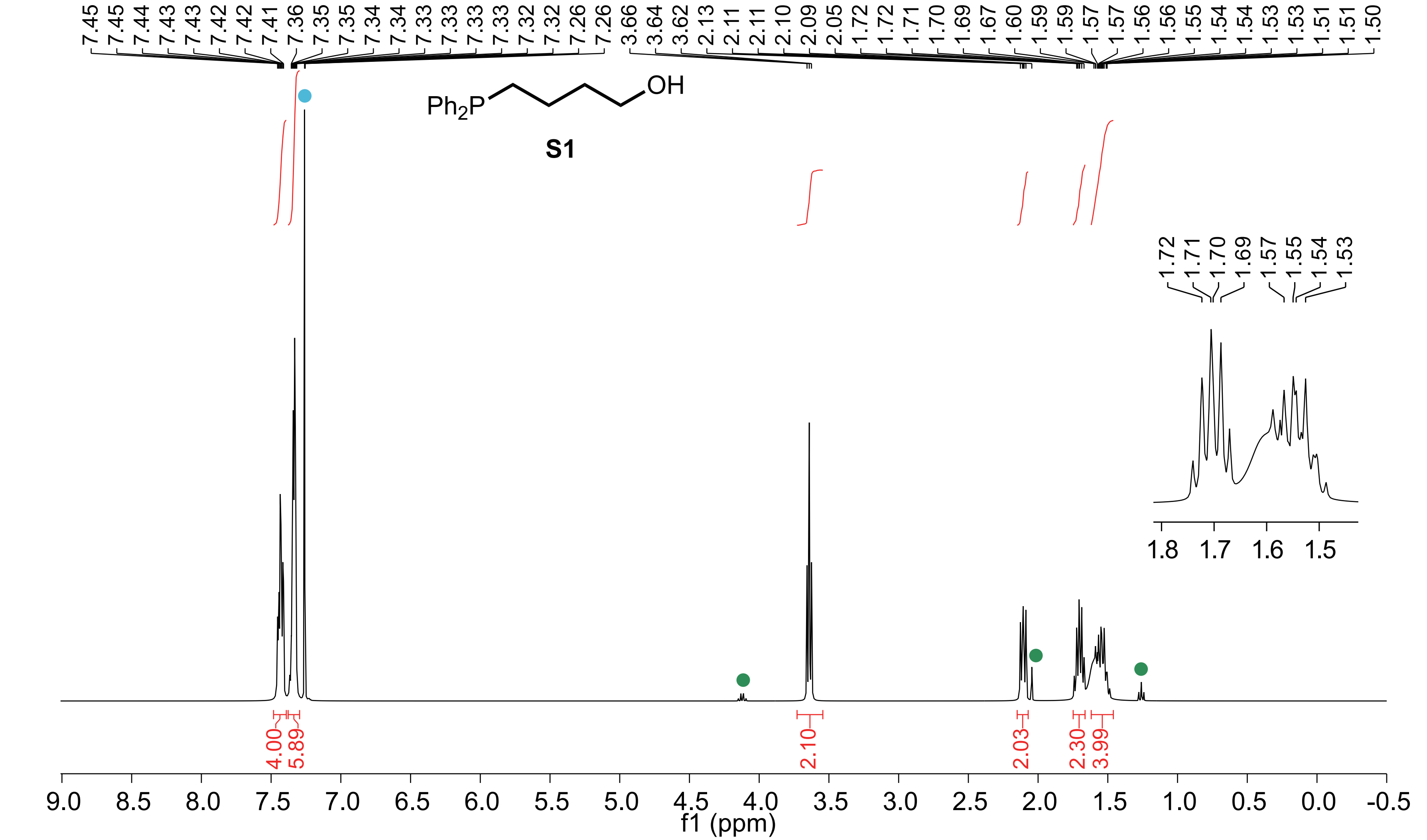

Figure S7 ^{1}H NMR (400.30 MHz, $CDCl_3$) spectrum of $PPh_2CH_2CH_2CH_2CH_2OH$ (**S1**). Residual proteo-solvent (•) and EtOAc (•). Inset shows the overlapping signals, including CH_2, OH and free H_2O.

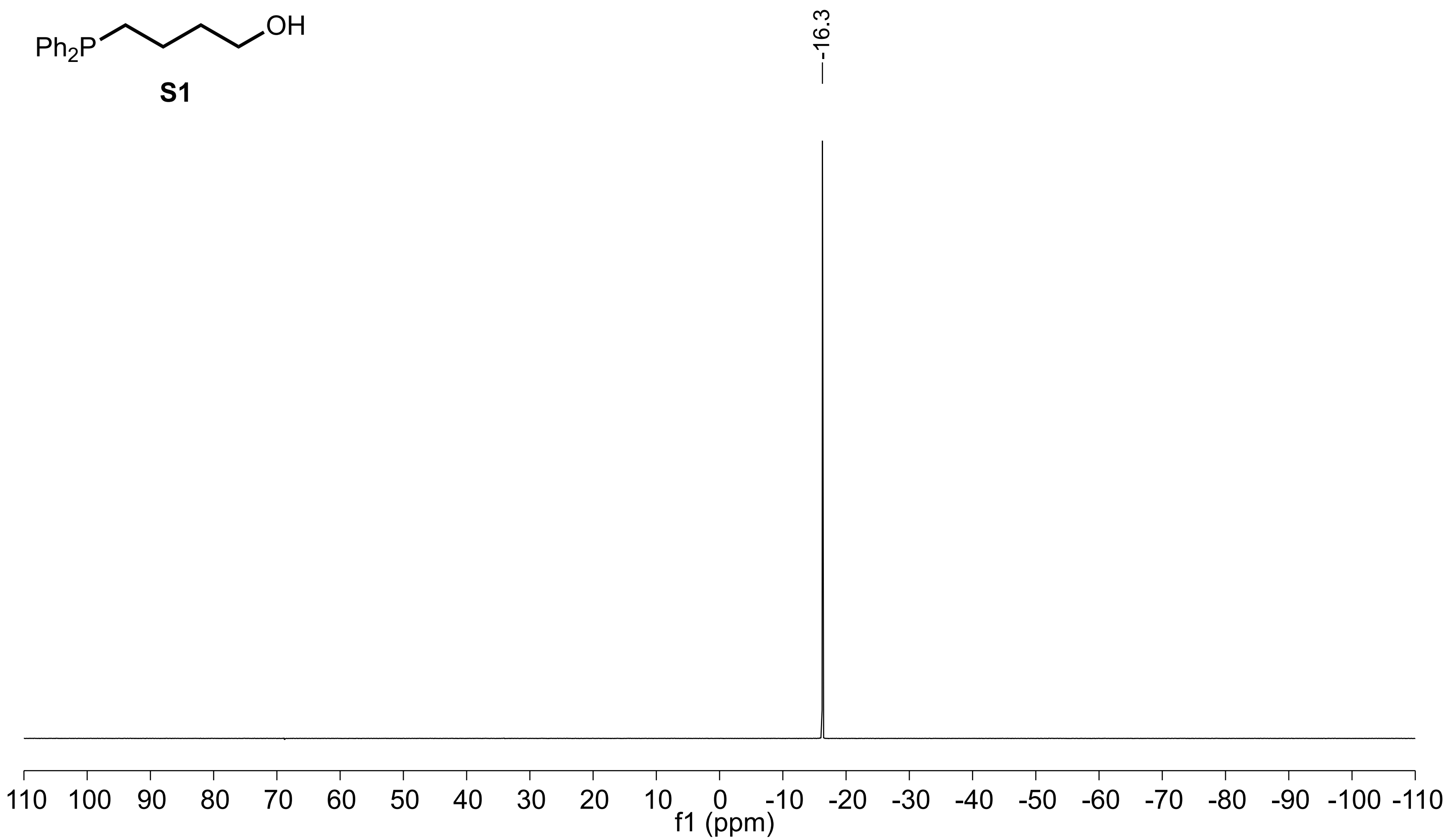

Figure S8 $^{31}P\{^{1}H\}$ NMR (162.04 MHz, $CDCl_3$) spectrum of $PPh_2CH_2CH_2CH_2CH_2OH$ (**S1**).

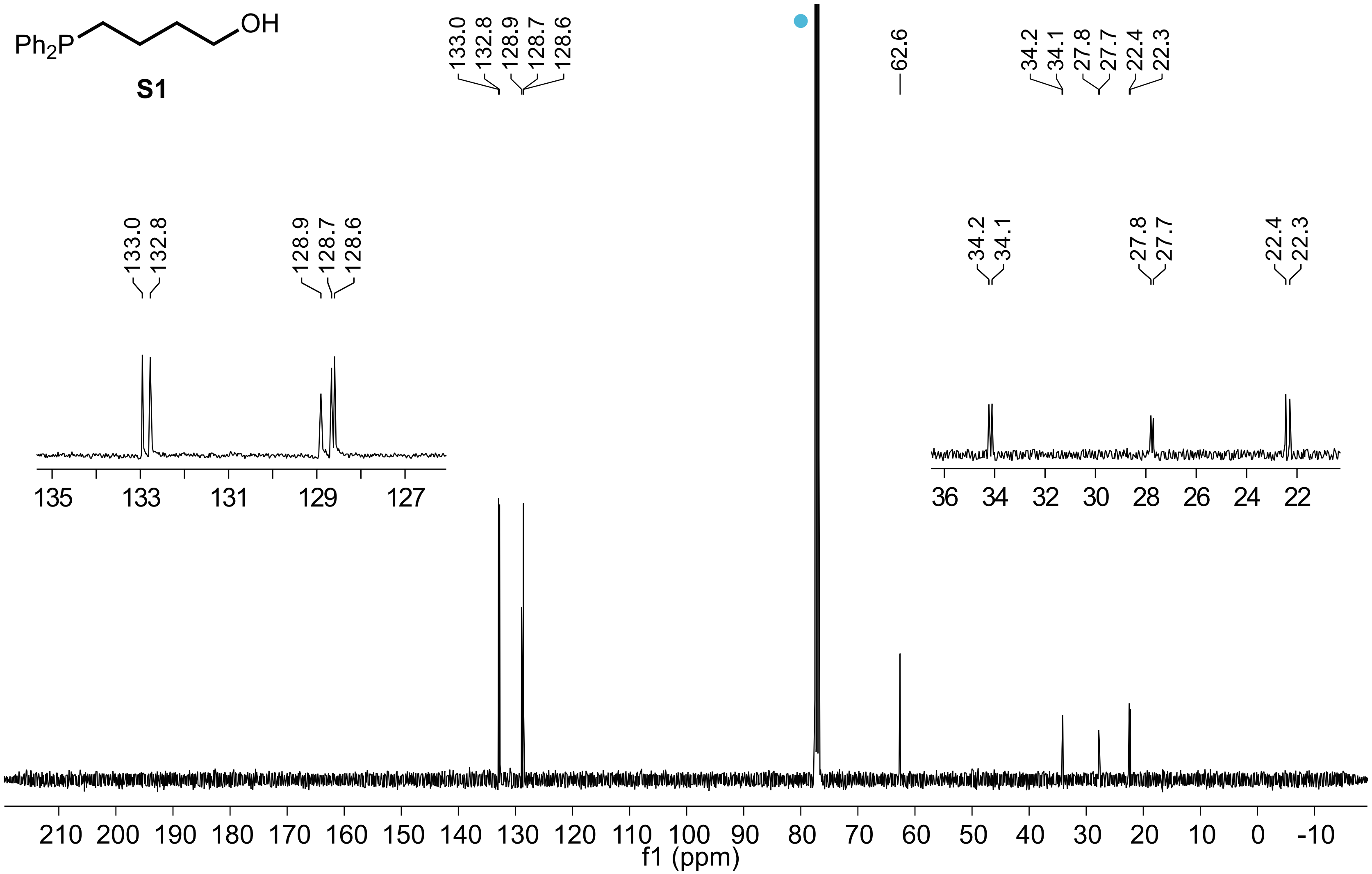

Figure S9 $^{13}C\{^{1}H\}$ NMR (100.67 MHz, $CDCl_3$) spectrum of $PPh_2CH_2CH_2CH_2CH_2OH$ (**S1**). Deuterated solvent (•).

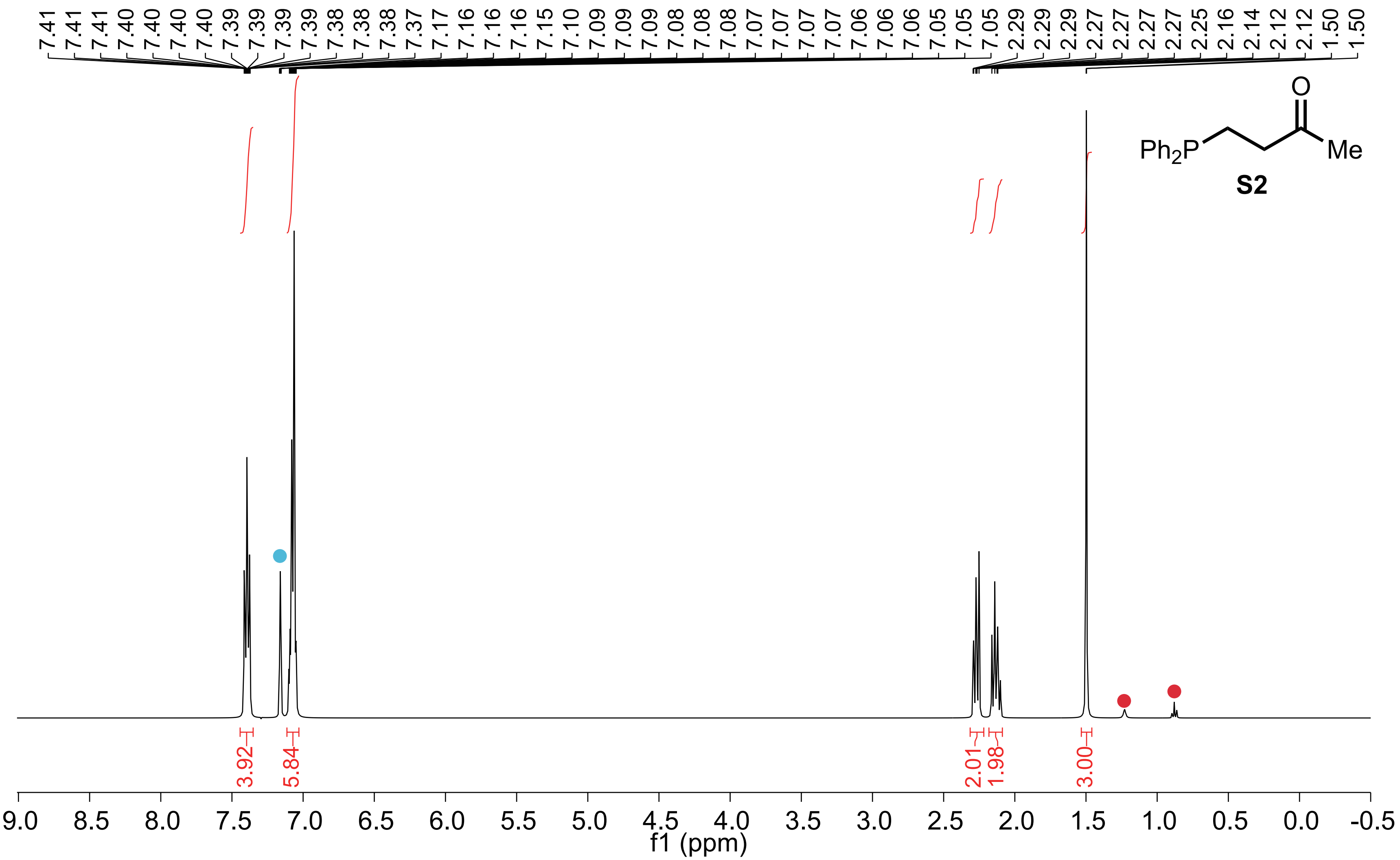

Figure S10 ^{1}H NMR (400.30 MHz, C_6D_6) spectrum of $PPh_2CH_2CH_2C(O)Me$ (**S2**). Residual proteo-solvent (●) and hexane (●).

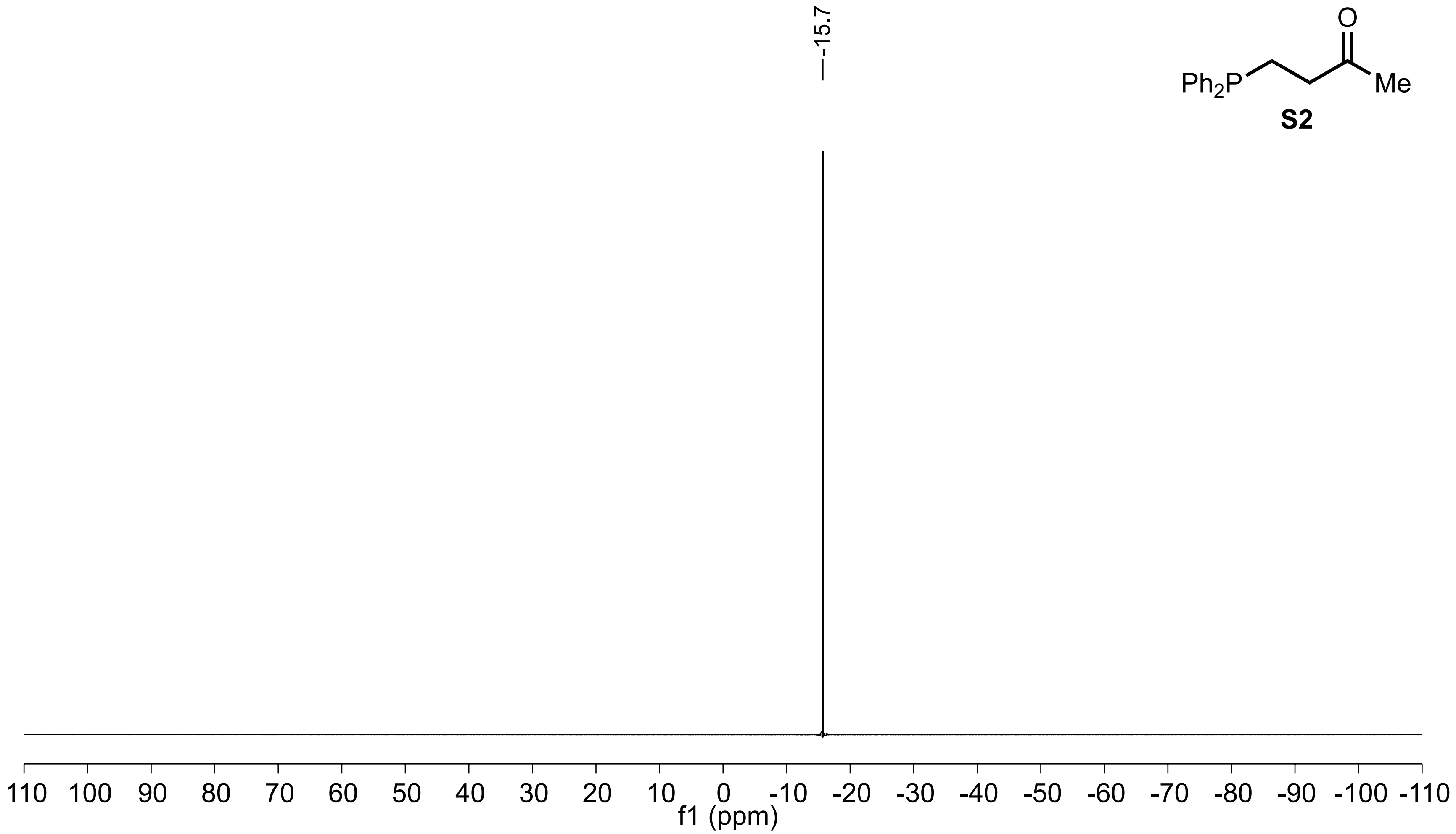

Figure S11 $^{31}P\{^{1}H\}$ NMR (162.04 MHz, C_6D_6) spectrum of $PPh_2CH_2CH_2C(O)Me$ (**S2**).

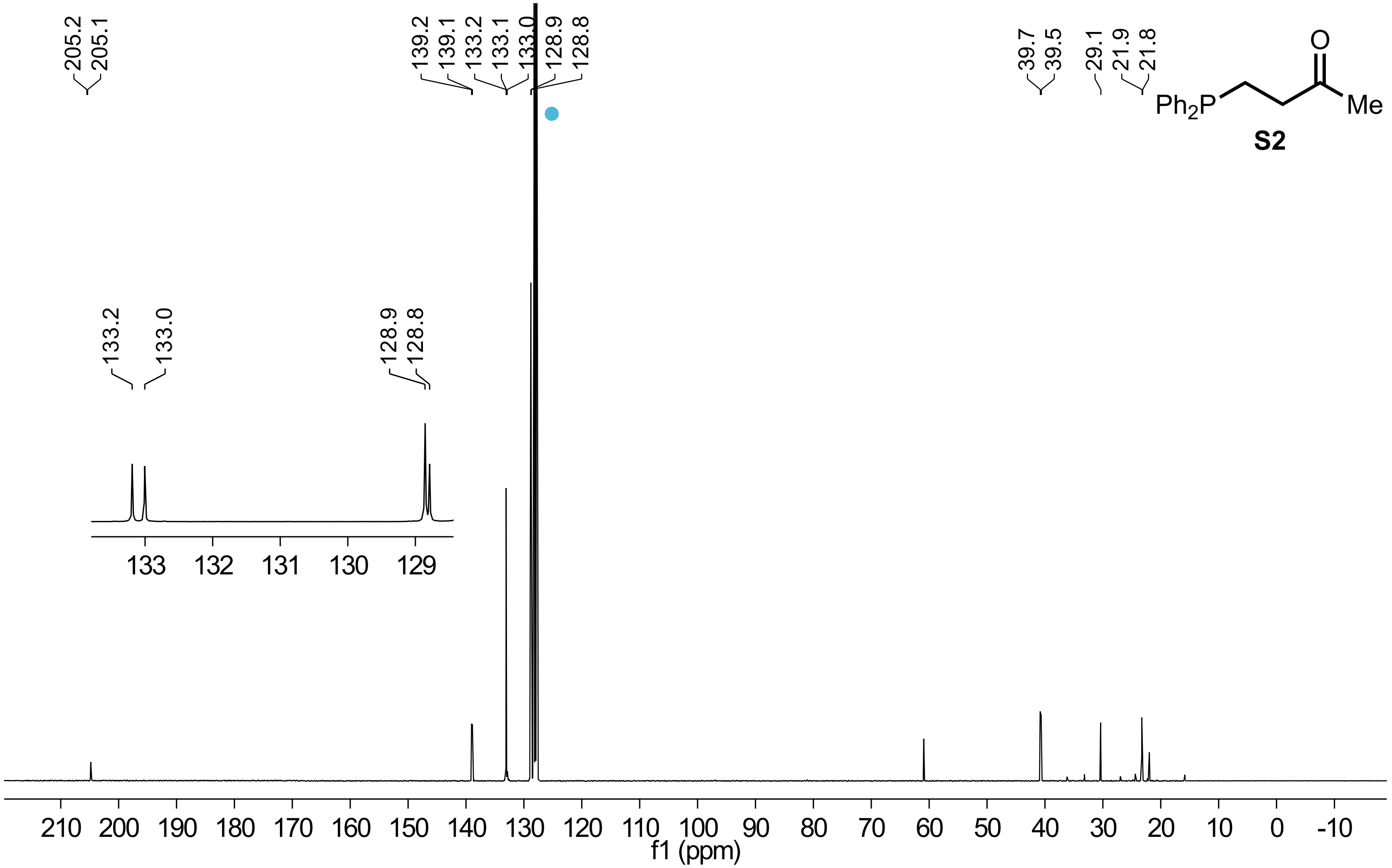

Figure S12 $^{13}C\{^{1}H\}$ NMR (100.67 MHz, C_6D_6) spectrum of $PPh_2CH_2CH_2C(O)Me$ (**S2**). Deuterated solvent (•). Signals near the baseline of the alkyl region due to solvents, pentane, and hexane.

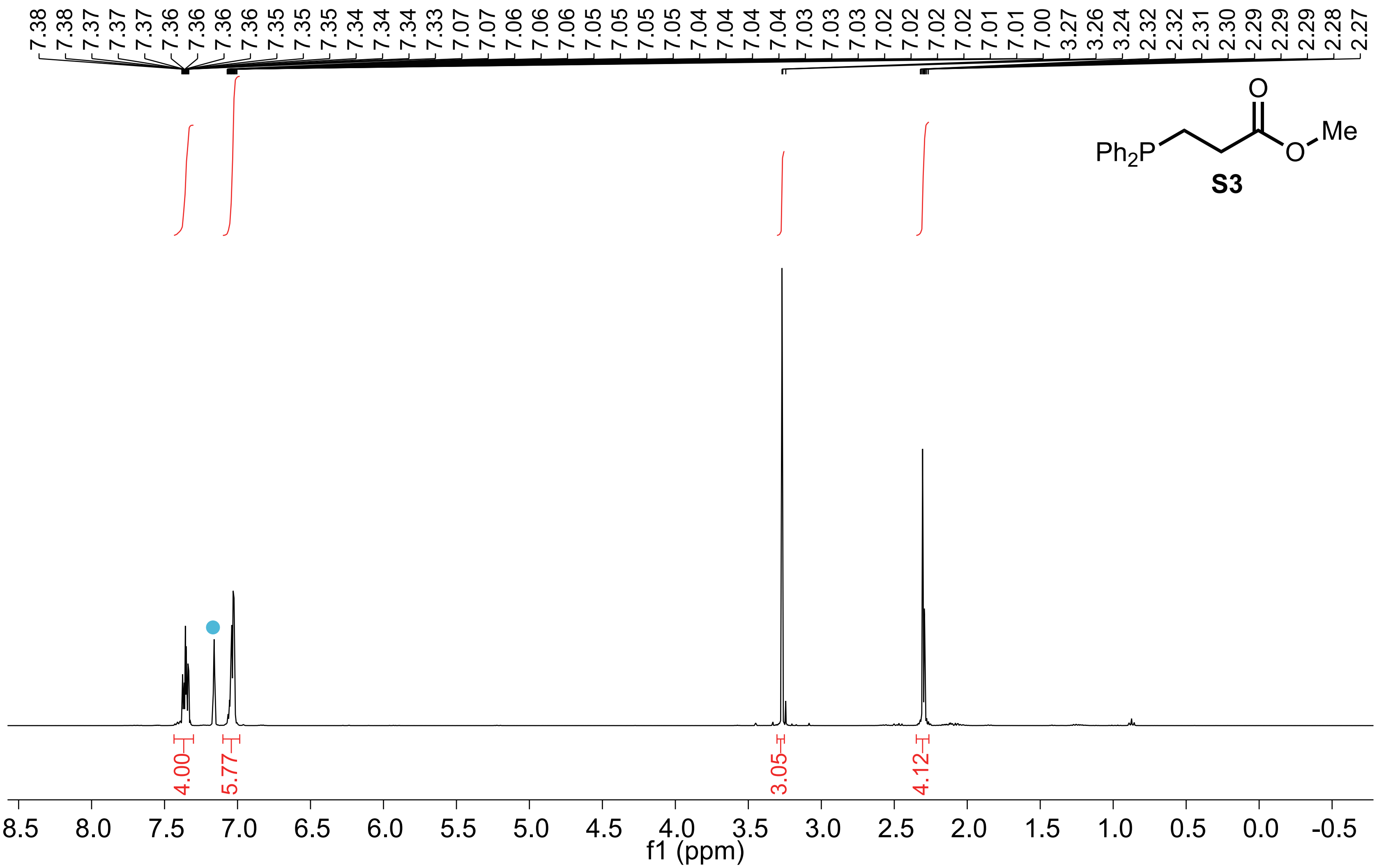

Figure S13 ^{1}H NMR (400.30 MHz, C_6D_6) spectrum of $PPh_2CH_2CH_2C(O)OMe$ (**S3**). Residual proteo-solvent (•)

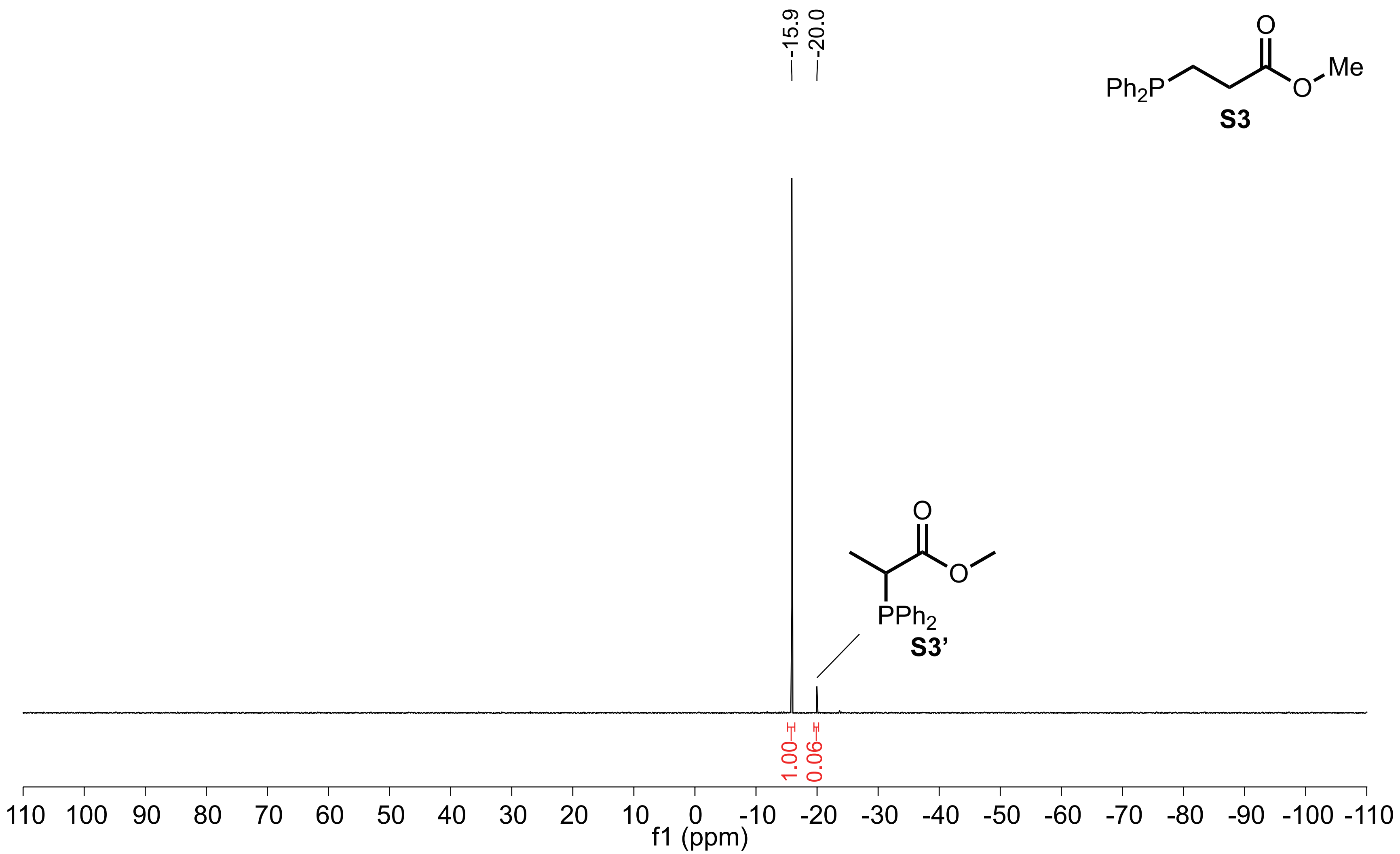

Figure S14 $^{31}P\{^1H\}$ NMR (162.04 MHz, C_6D_6) spectrum of $PPh_2CH_2CH_2C(O)OMe$ (**S3**), which contains 6% of the branched isomer (**S3'**).

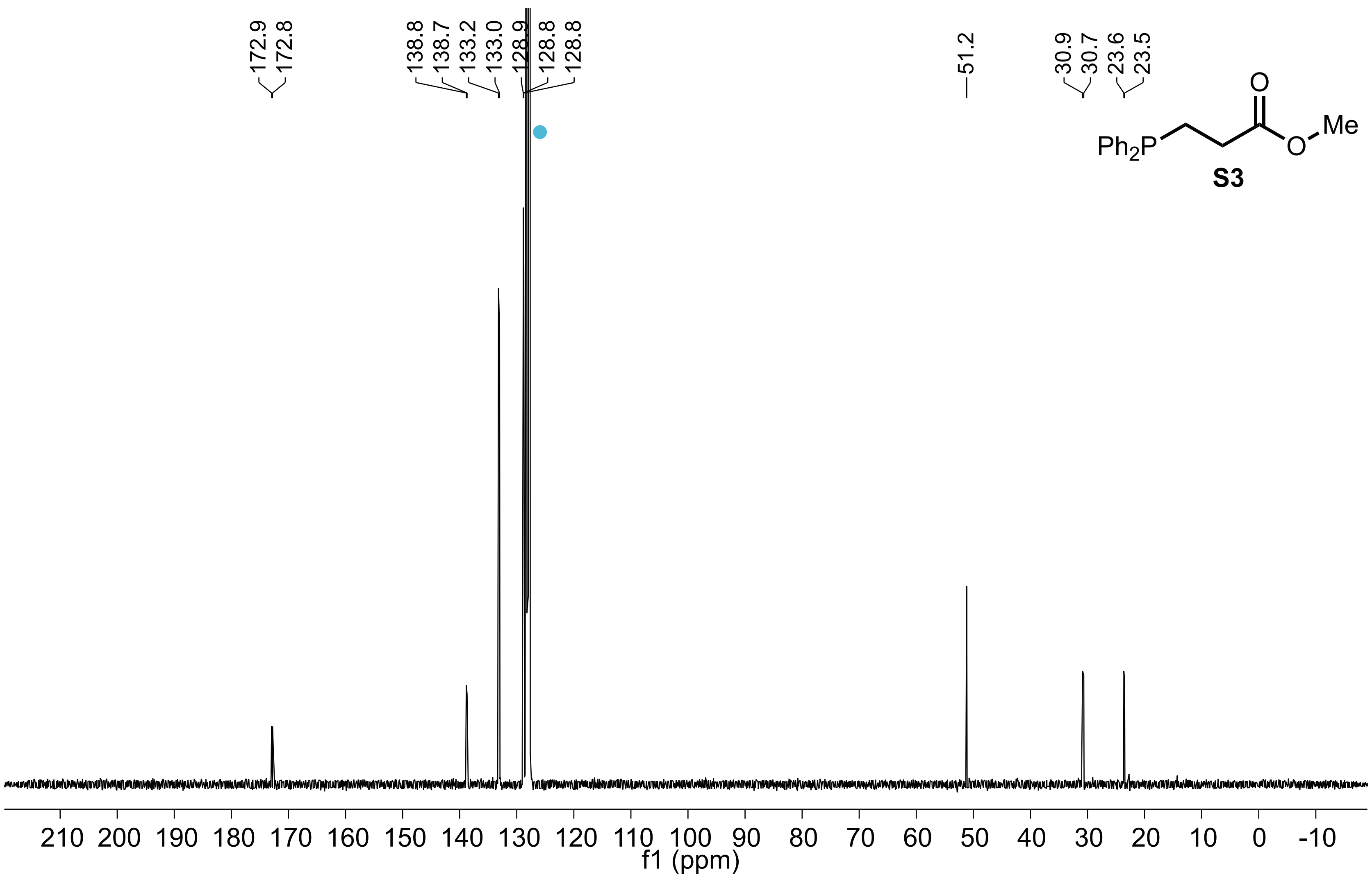

Figure S15 $^{13}C\{^{1}H\}$ NMR (100.67 MHz, C_6D_6) spectrum of $PPh_2CH_2CH_2C(O)OMe$ (**S3**). Deuterated solvent (•).

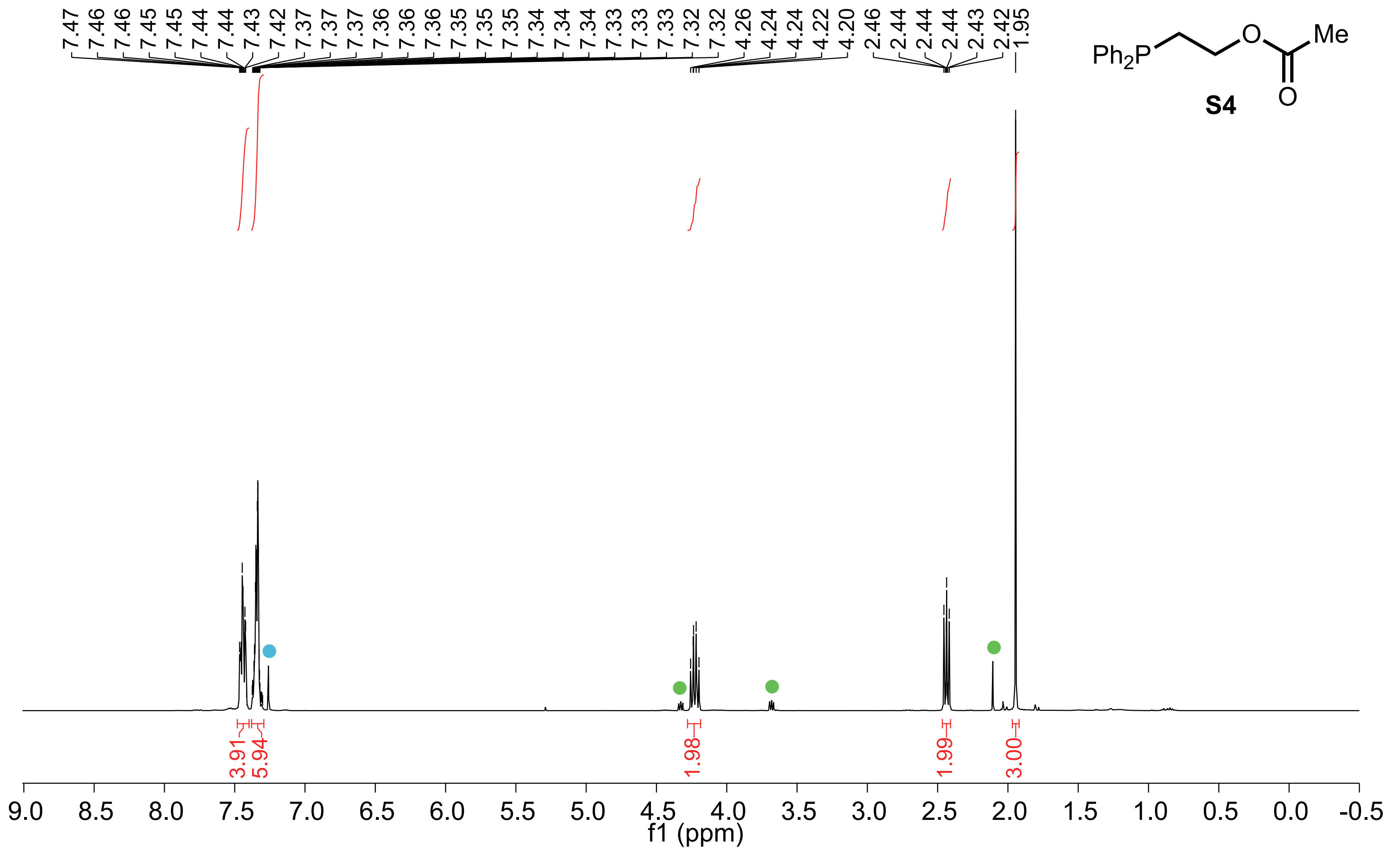

Figure S16 ^{1}H NMR (400.30 MHz, C_6D_6) spectrum of $PPh_2CH_2CH_2OC(O)Me$ (**S4**). Residual proteo-solvent (•) and unreacted starting material $ClCH_2CH_2OH$ (•).

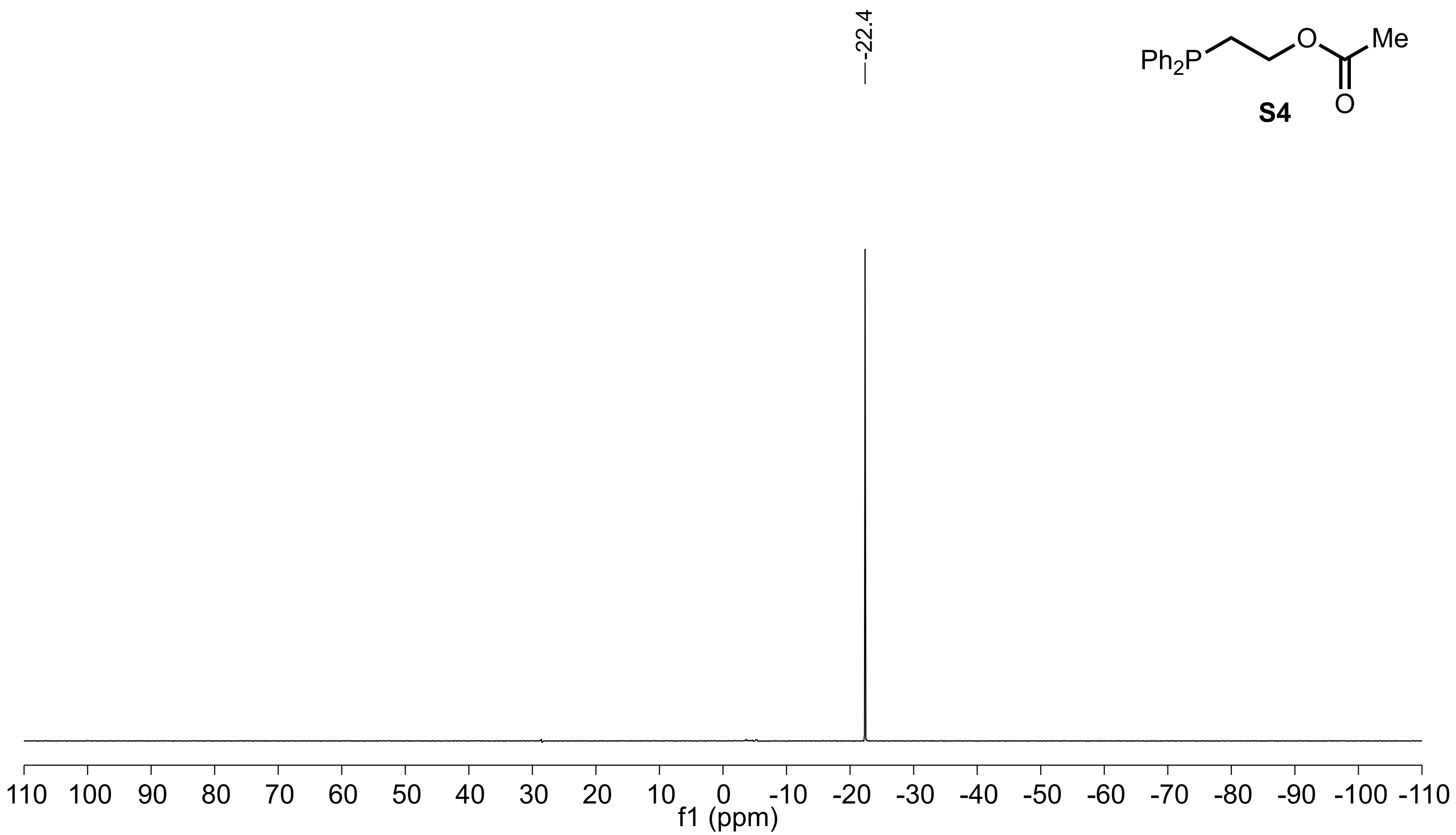

Figure S17 $^{31}P\{^1H\}$ NMR (162.04 MHz, C_6D_6) spectrum of $PPh_2CH_2CH_2OC(O)Me$ (**S4**).

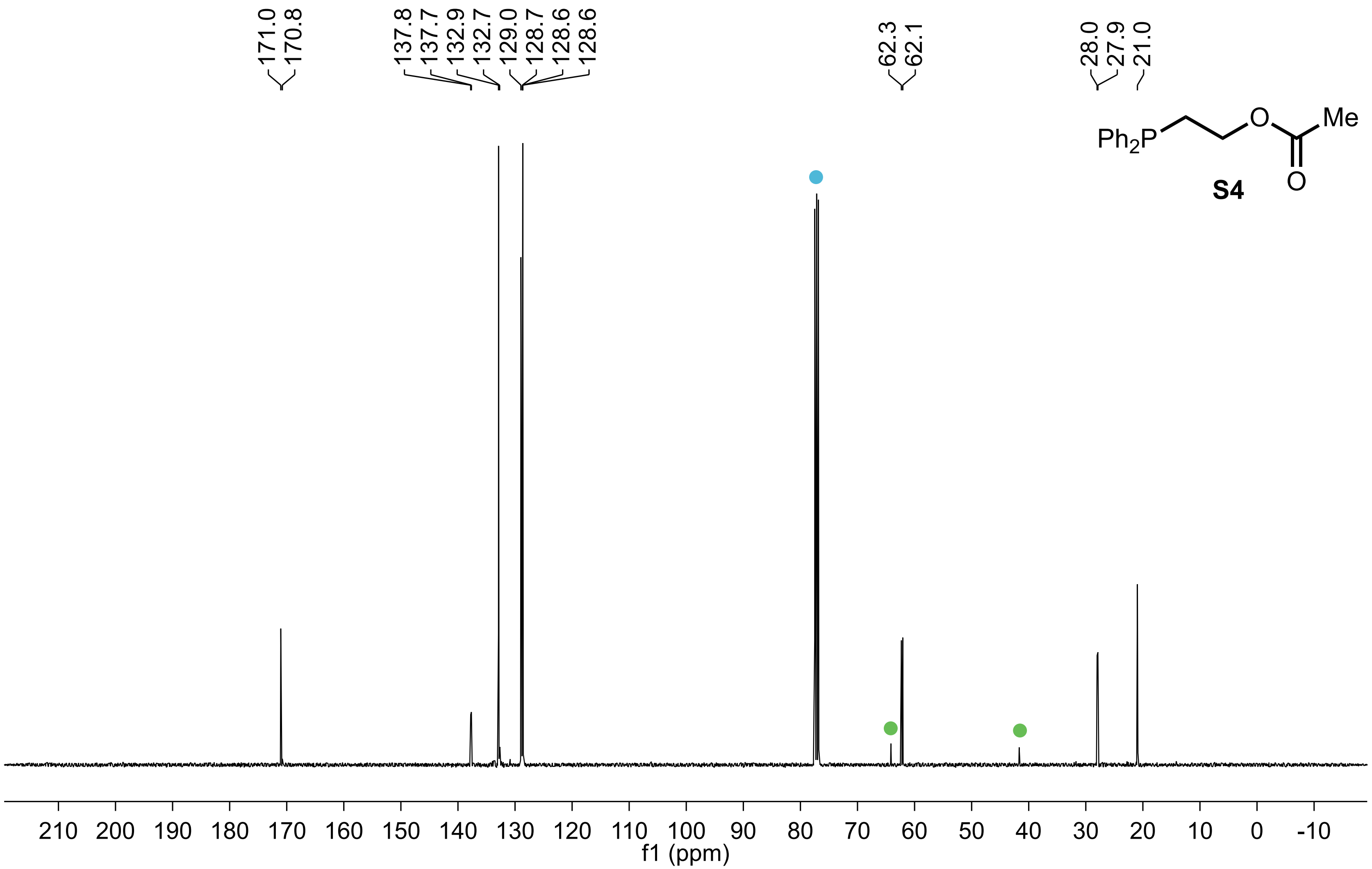

Figure S18 $^{13}C\{^1H\}$ NMR (100.67 MHz, C_6D_6) spectrum of $PPh_2CH_2CH_2OC(O)Me$ (**S4**). Deuterated solvent (•) and unreacted starting material $ClCH_2CH_2OH$ (•).

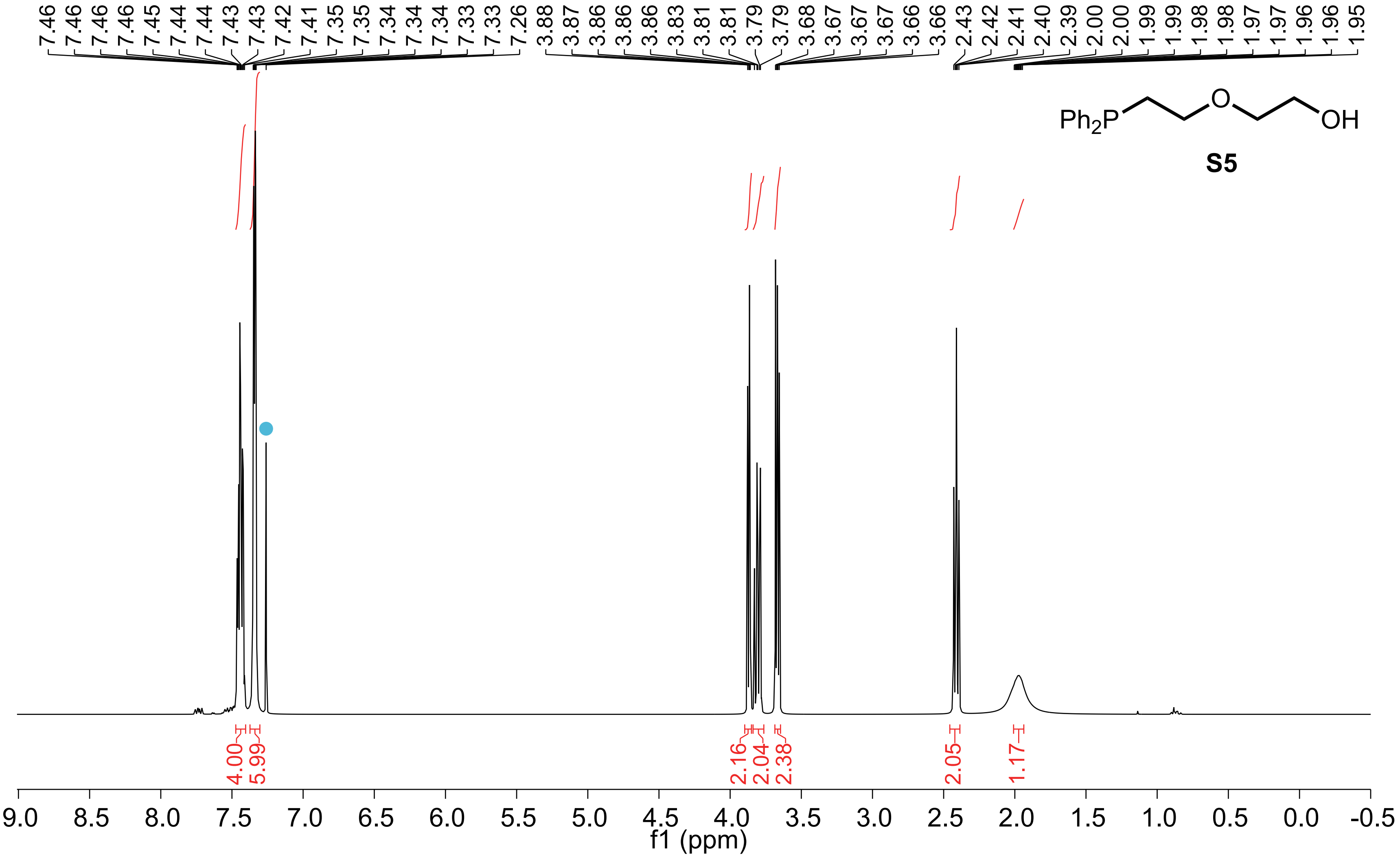

Figure S19 ^{1}H NMR (400.30 MHz, $CDCl_3$) spectrum of $PPh_2CH_2CH_2OCH_2CH_2OH$ (**S5**). Residual proteo-solvent (•).

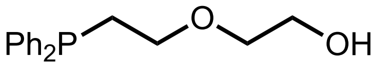

S5

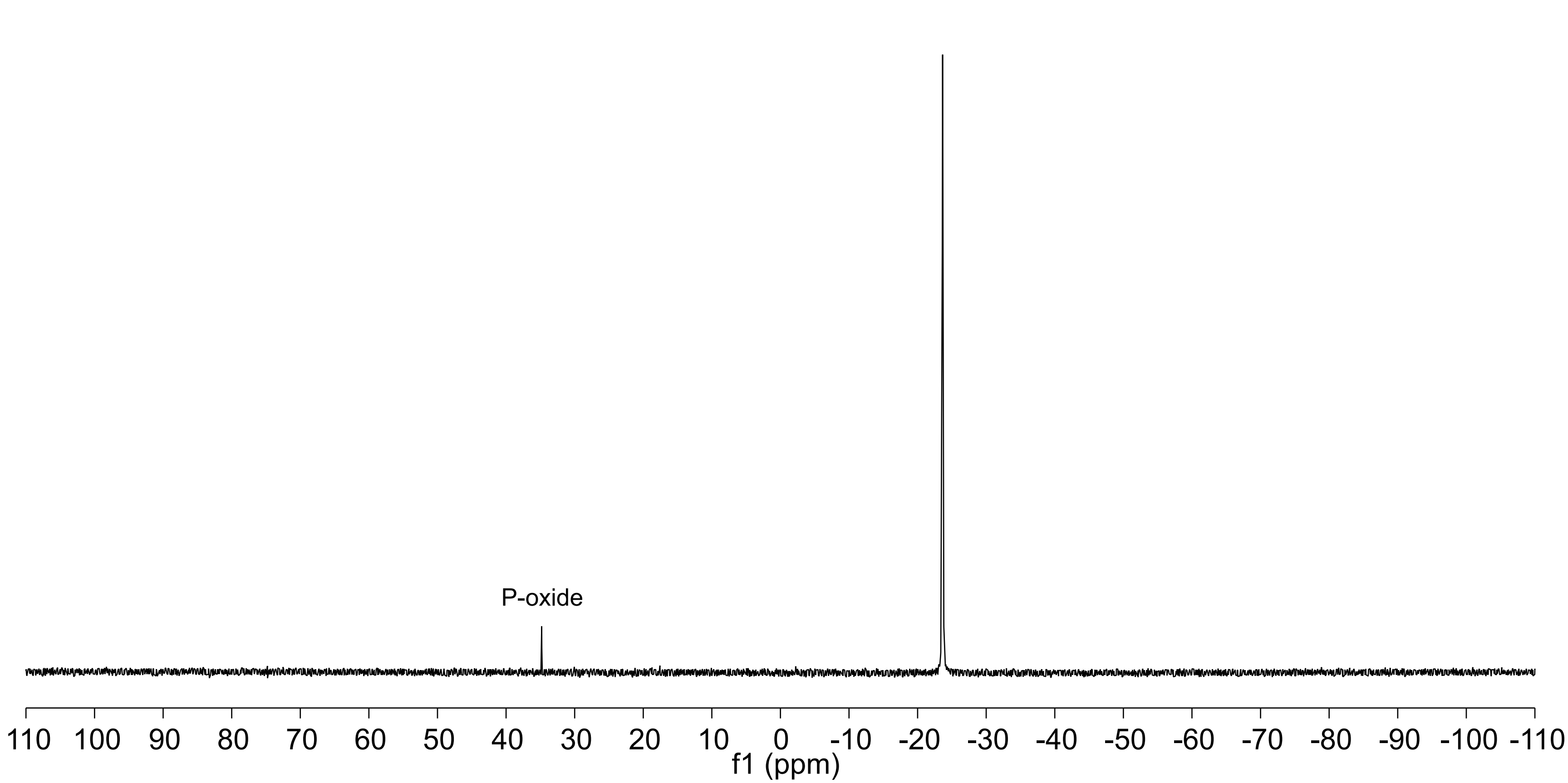

Figure S20 $^{31}P\{^{1}H\}$ NMR (162.04 MHz, C_6D_6) spectrum of $PPh_2CH_2CH_2OCH_2CH_2OH$ (**S5**).

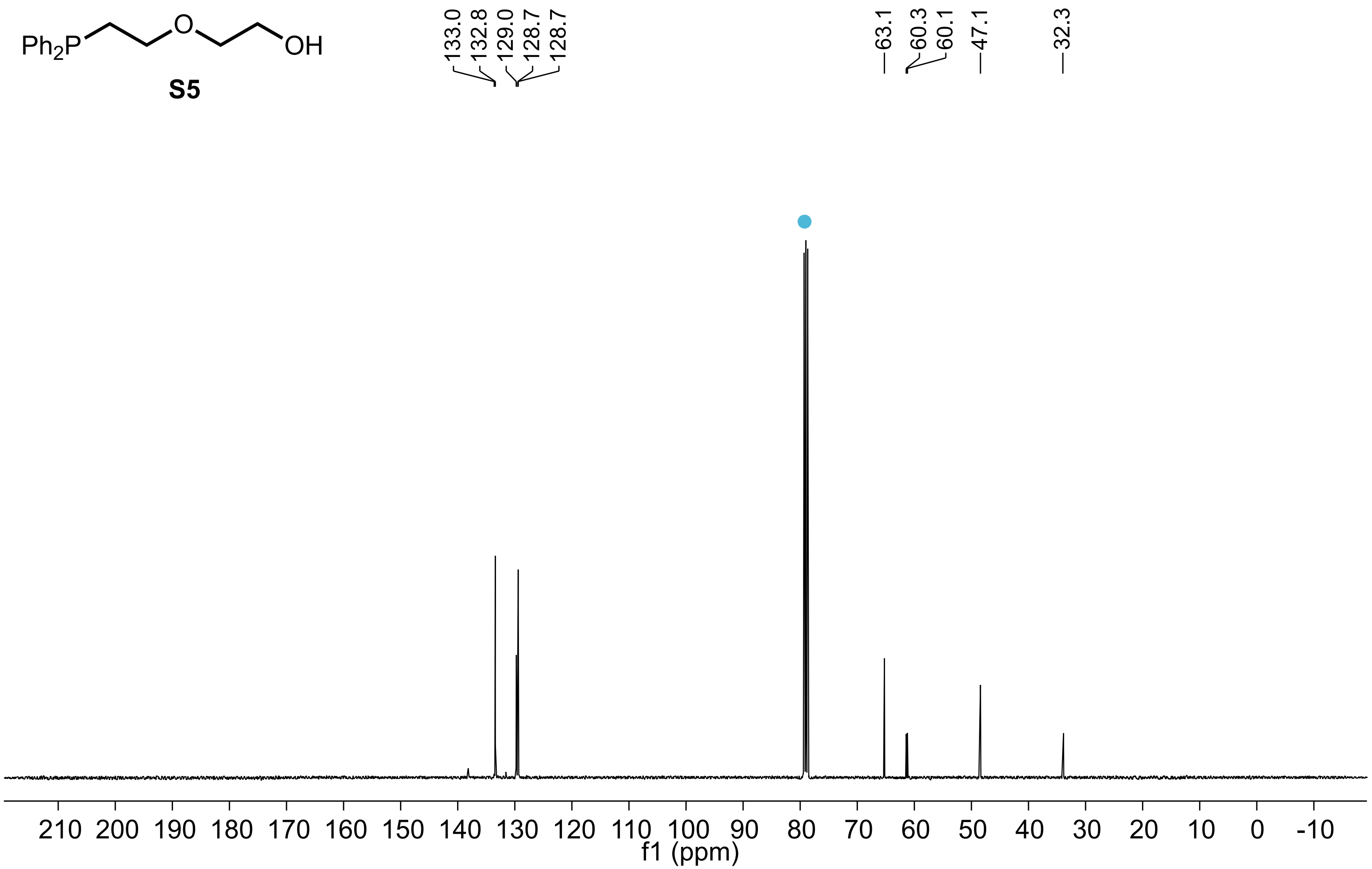

Figure S21 $^{13}C\{^{1}H\}$ NMR (100.67 MHz, C_6D_6) spectrum of $PPh_2CH_2CH_2OCH_2CH_2OH$ (**S5**). Deuterated solvent (•).

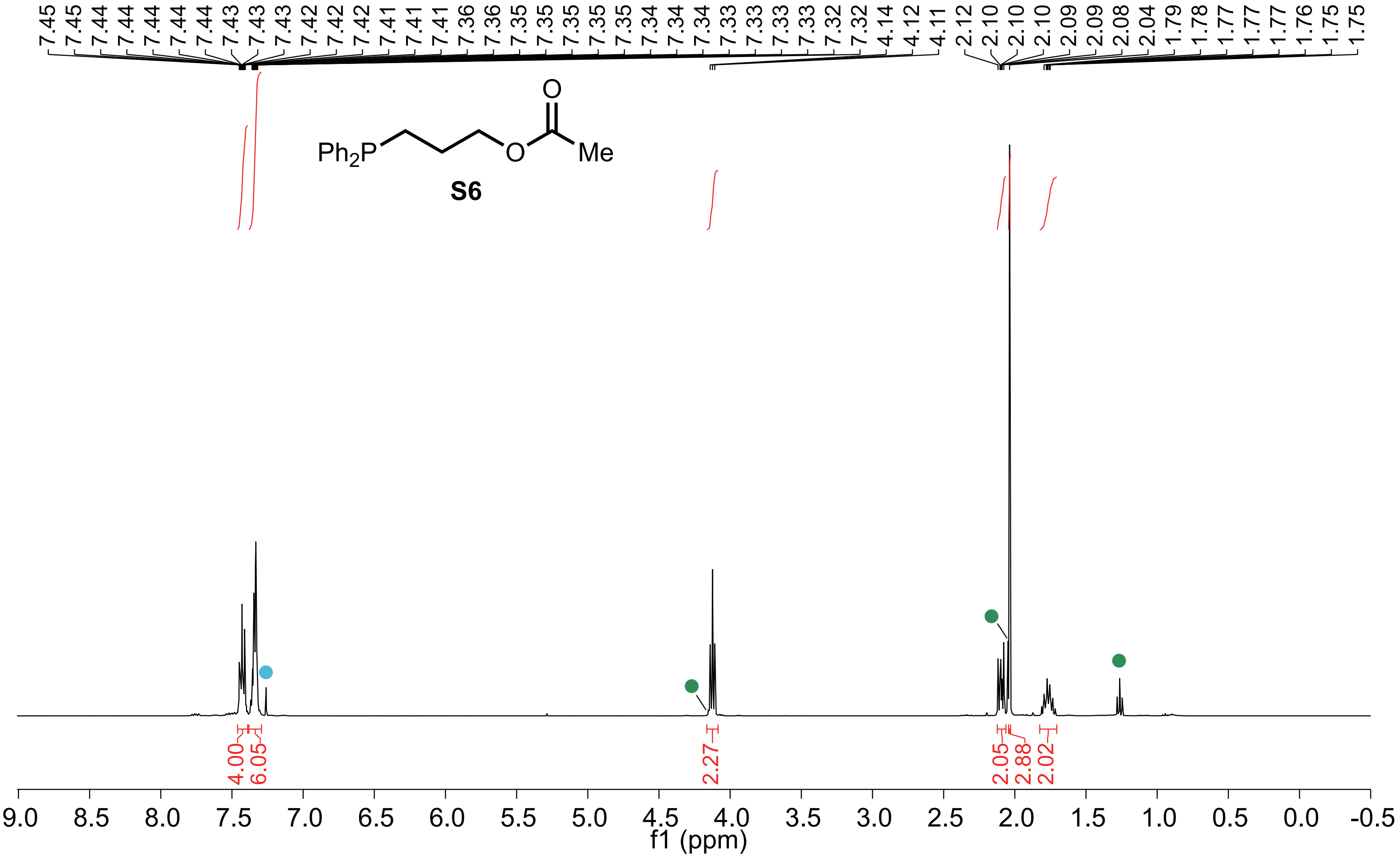

Figure S22 ^{1}H NMR (400.30 MHz, $CDCl_3$) spectrum of $PPh_2CH_2CH_2CH_2OC(O)Me$ (**S6**). Residual proteo-solvent (•) and EtOAc (•).

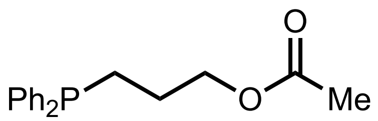

S6

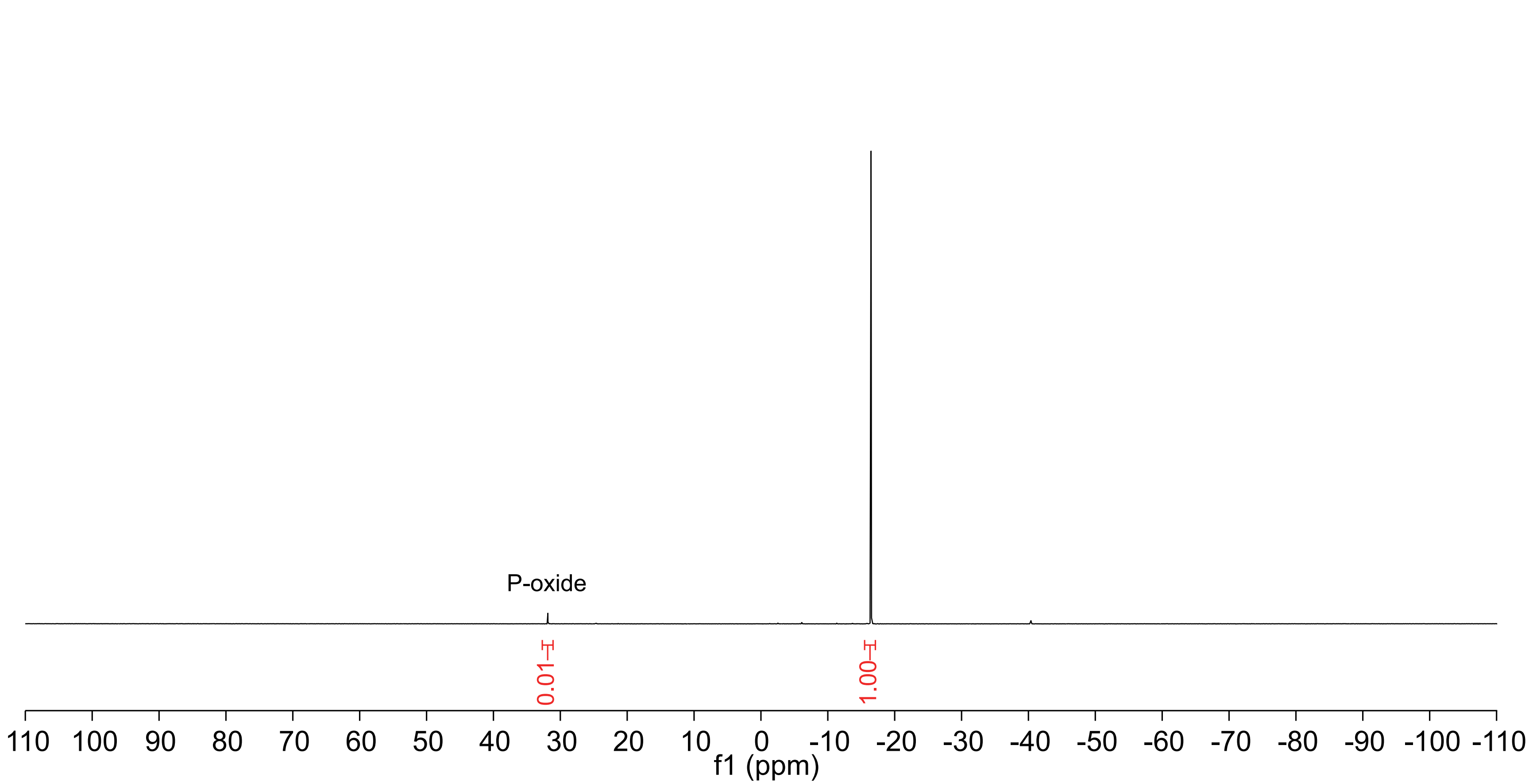

Figure S23 $^{31}P\{^1H\}$ NMR (162.04 MHz, C_6D_6) spectrum of $PPh_2CH_2CH_2CH_2OC(O)Me$ (**S6**).

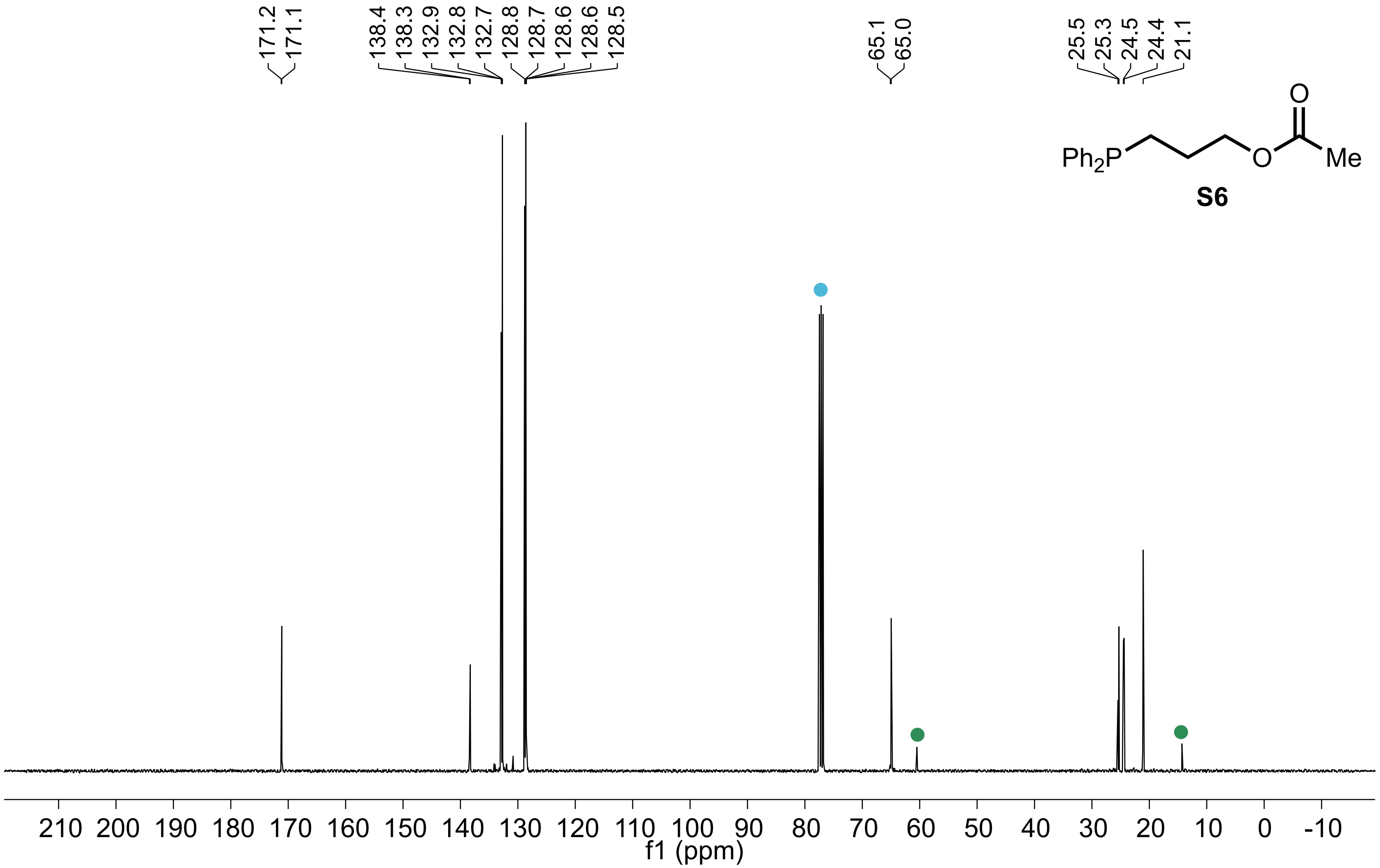

Figure S24 $^{13}C\{^{1}H\}$ NMR (100.67 MHz, C_6D_6) spectrum of $PPh_2CH_2CH_2CH_2OC(O)Me$ (**S6**). Deuterated solvent (•) and EtOAc (•).

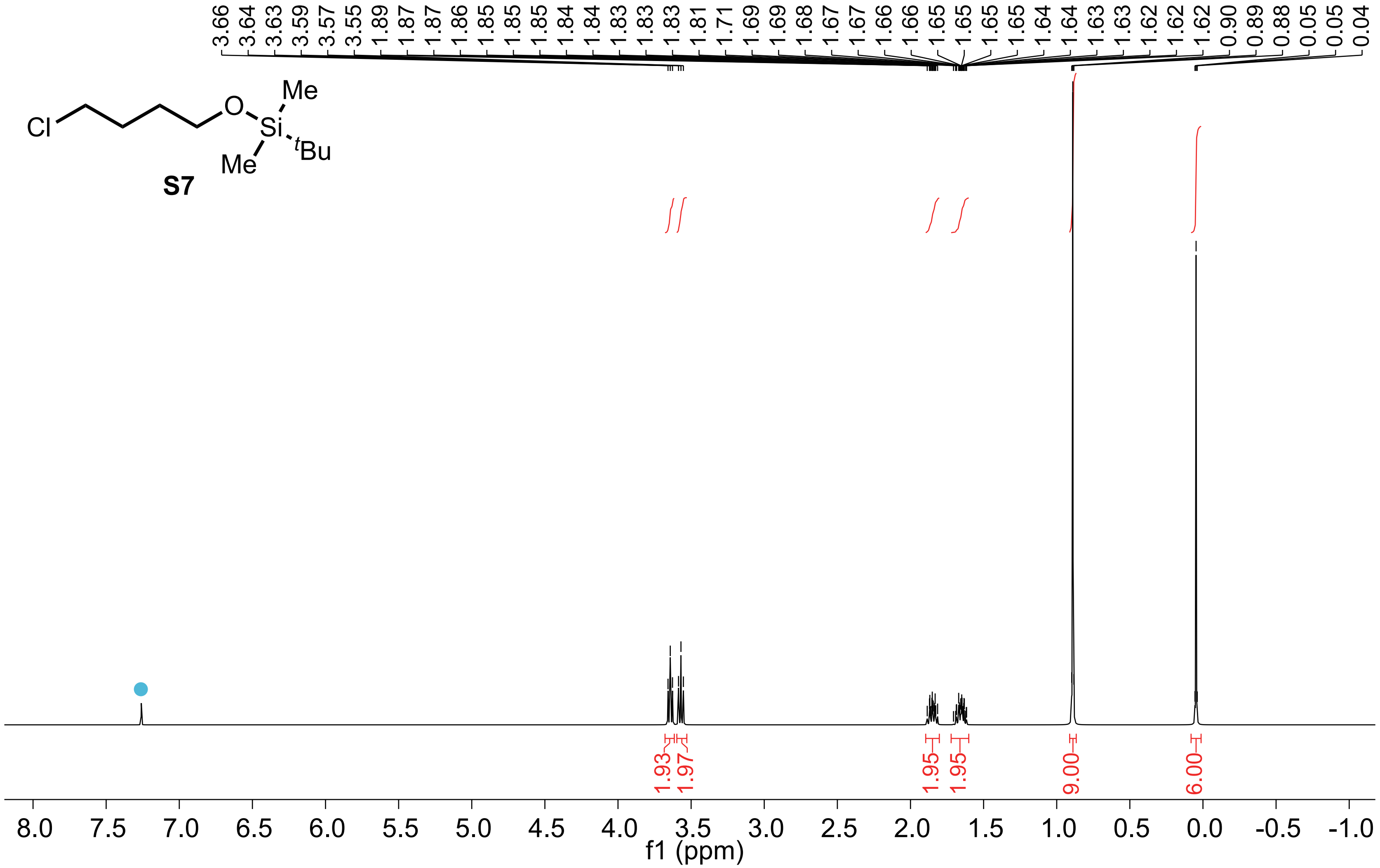

Figure S25 ^{1}H NMR (400.30 MHz, $CDCl_3$) spectrum of $ClCH_2CH_2CH_2CH_2OSiMe_2Bu^t$ (**S7**). Residual proteo-solvent (•)

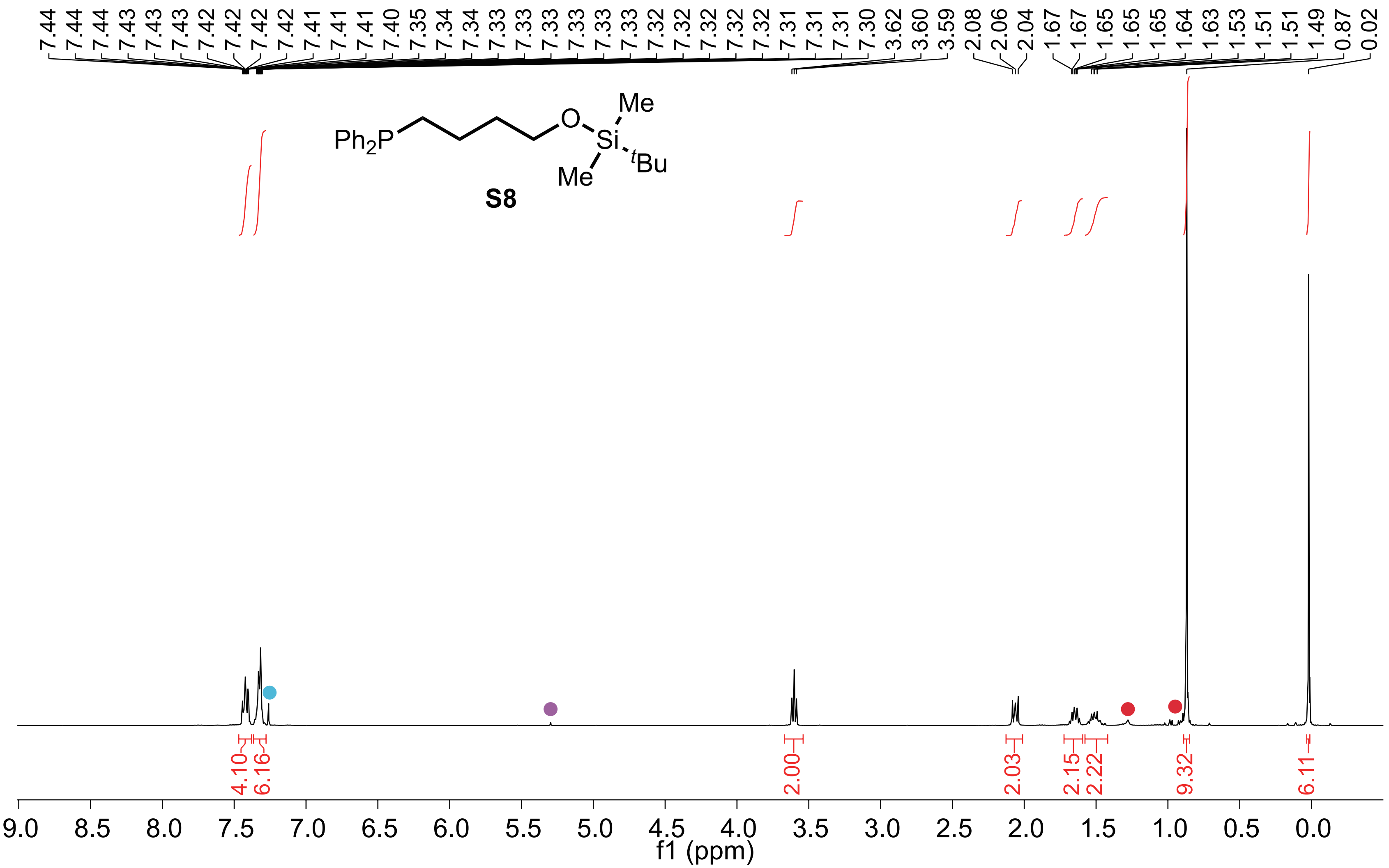

Figure S26 ^{1}H NMR (400.30 MHz, $CDCl_3$) spectrum of $PPh_2CH_2CH_2CH_2OSiMe_2Bu^t$ (**S8**). Residual proteo-solvent (●), CH_2Cl_2 (●) and hexane (●).

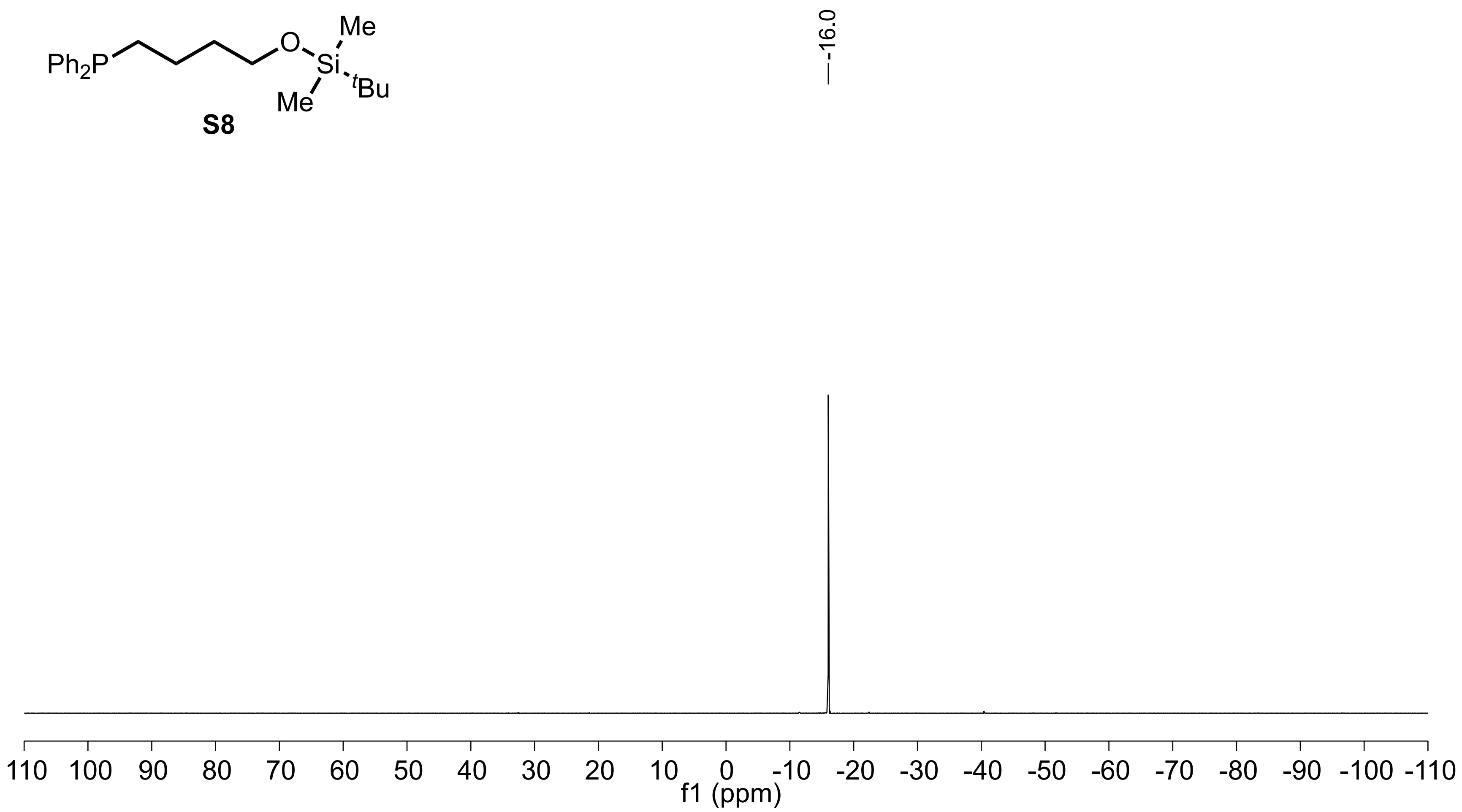

Figure S27 $^{31}P\{^1H\}$ NMR (162.04 MHz, $CDCl_3$) spectrum of $PPh_2CH_2CH_2CH_2OSiMe_2Bu^t$ (**S8**).

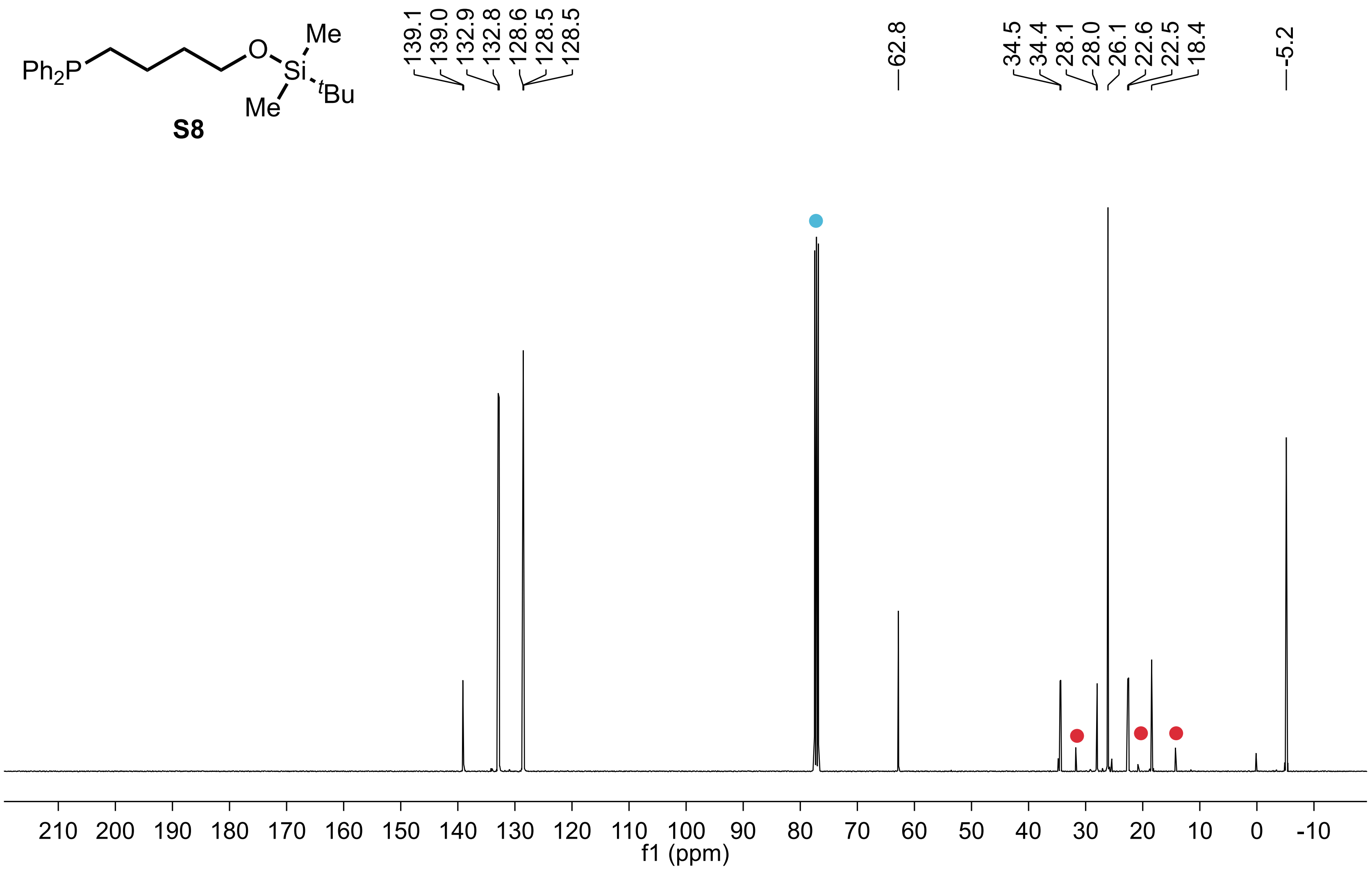

Figure S28 $^{13}C\{^{1}H\}$ NMR (100.67 MHz, $CDCl_3$) spectrum of $PPh_2CH_2CH_2CH_2OSiMe_2Bu^t$ (**S8**). Deuterated solvent (•) and hexane (•).

2.11 Evaluation of phosphine ligands on Ni-SMC

General procedure for the optimized ligand and catalyst screening

In a nitrogen-filled glove box, $Ni(cod)_2$ (with 4.0 equiv of phosphine ligand) or a Ni-PPh_2Me catalyst (with 2.0-3.0 equiv of PPh_2Me), 4-bromoacetophenone (**4**), and *p*-tolylboronic acid pinacol ester (B(*p*-Tol)Pin **5**) were separately dissolved in 2-MeTHF to prepare stock solutions. Solid K_3PO_4 (2.5 equiv) was weighed into a 2 mL crimp-top vial. The catalyst solution (0.2 mL, 0.5-3 mol%), **4** (0.1 mL, 0.2 mmol, 1.0 equiv), and **5** (0.1 mL, 1.1 equiv) were sequentially added to the vial. The vial was then sealed, removed from the glove box, and added with deionized water (80 μL). The vial was placed in a shaker and heated to 60-80 °C with an agitation speed of 800 rpm. To the reaction mixture, *n*-decane (0.1 mmol) was added as the internal standard. Samples were extracted at specific time intervals and extracted with ethyl acetate. The resultant organic layer was separated, and subsequently washed with water. An aliquot of the organic layer was analyzed by GC. %Yields in duplicate runs displayed an error of ±2%.

Table S1 Evaluation of Ligands with Various Catalyst Loading.[a]

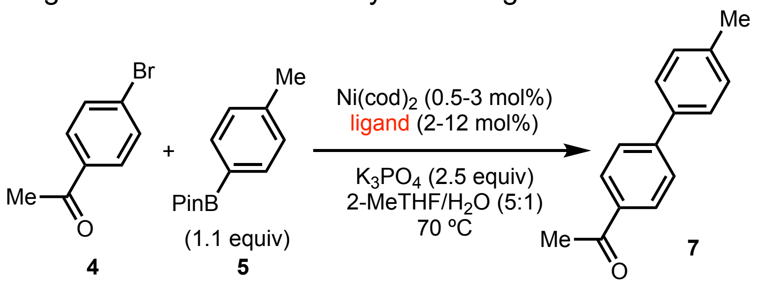

Entry	Ligands	%Yield at 3 mol% Ni	%Yield at 1 mol% Ni	%Yield at 0.5 mol% Ni
1	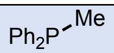	98(58)	96(37)	81
2	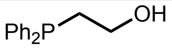	46(9)	46(8)	29
**3**	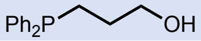	**99(78)**	**98(75)**	**98**
4	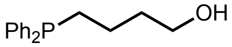	98(94)	93(67)	70
5	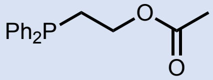	99(85)	99(45)	72
6	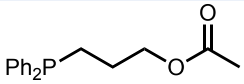	99(95)	97(51)	82
7	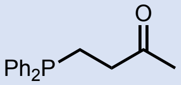	99(88)	97(52)	51
8	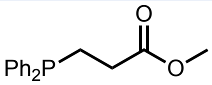	69(14)	38(38)	21
9	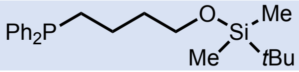	98(94)	92(54)	39
10	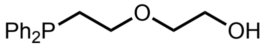	54(9)	51(8)	33

[a]%Yields determined by GC after 24 h and %yields at 2 h in the parentheses.

Table S2 Evaluation of Temperature Effect.[a]

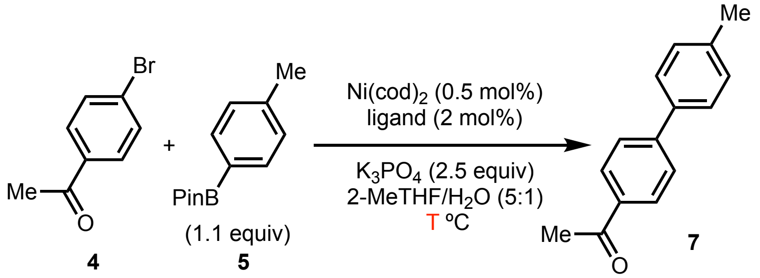

Entry	Ligands	%Yield (60 °C)	%Yield (70 °C)	%Yield (80 °C)
1	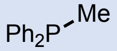	42	81	71
**2**	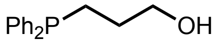	**98**	**98**	**99**
3	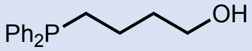	78	70	70
4	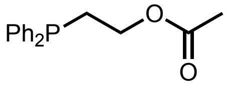	70	72	55
5	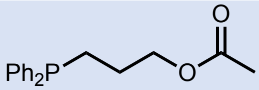	83	82	63

[a]%Yields determined by GC after 24 h.

Table S3 Evaluation of Base and Boronate Effect for Ni-PPh_2Me Catalysis.[a]

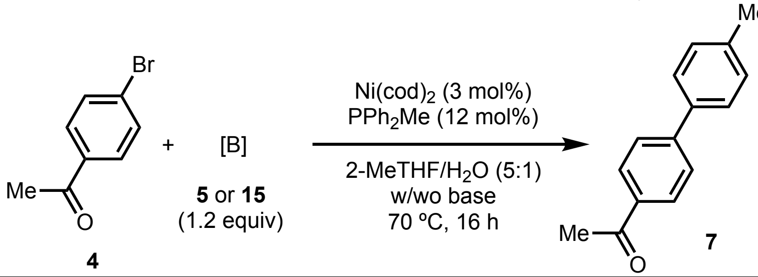

Entry	[B]	Base (equiv)		%Yield
1	K[B(*p*-Tol)Pin(OH)] (**15**)	K_3PO_4	2.5	72
2	K[B(*p*-Tol)Pin(OH)] (**15**)	-	-	76
3	B(*p*-Tol)Pin (**5**)	K_3PO_4	2.5	98
4	B(*p*-Tol)Pin (**5**)	KOH	1.2	66
5	B(*p*-Tol)Pin (**5**)	KOH	2.5	99

[a] Condition: [**4**] = 0.5 M, %yields determined by GC after 16 h.

Table S4 Evaluation of Base Effect for Ni-ProPhos Catalysis.[a]

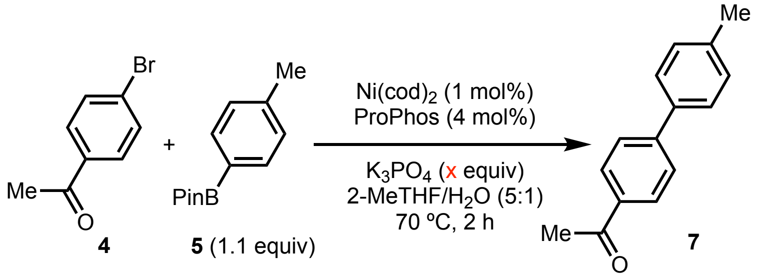

Entry	Base equiv	%Yield
1	0.3	28
2	0.9	30
3	1.5	32
4	2.1	38
**5**	**2.5**	**48**
6	2.7	40
7	4.0	35
8	5.0	31

[a] Condition: [**4**] = 0.5 M, %yields determined by GC after 2 h.

Table S5 Evaluation of Activity for Ni-PPh_2Me Catalysts.[a]

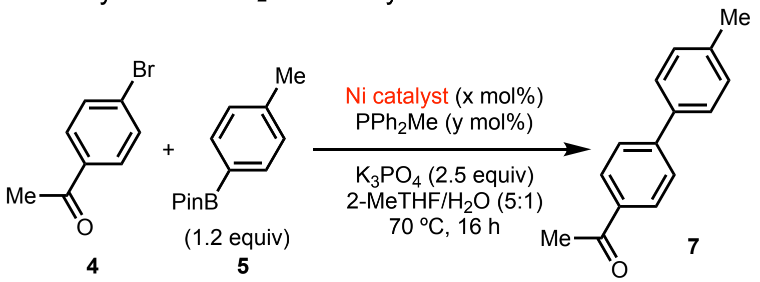

Entry	Ni catalyst (mol%)	mol% of PPh_2Me	%Yield
1	$Ni(cod)_2$ (3)	12	98
2	$Ni(PPh_2Me)_4$ (3)	-	98
3	$Ni(PPh_2Me)_2Cl$(*o*-Tol) (3)	6	80
4	$Ni(PPh_2Me)_2Br$(*o*-Tol) (3)	6	81
5	$[Ni(PPh_2Me)(\mu\text{-}OH)(o\text{-}Tol)]_2$ (1.5)	9	75

[a] Condition: [**4**] = 0.5 M, %yields determined by GC after 16 h.

3. Kinetics experiments

3.1 General procedure for kinetic experiments

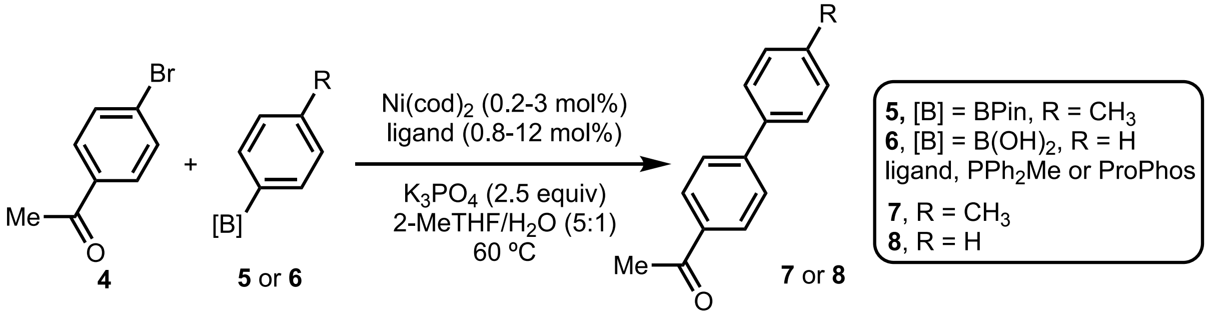

In a nitrogen-filled glove box, Ni(cod)$_2$ (with 4.0 equiv of PPh$_2$Me or ProPhos **9**), 4-bromoacetophenone (**4**) and a boronic substrate (B(*p*-Tol)Pin **5** or BPh(OH)$_2$ **6**) were separately dissolved in 2-MeTHF to form stock solutions in various concentrations, respectively. In most cases, solid K_3PO_4 (2.5 equiv) was weighed into a 2 mL crimp-top vial. The solutions of Ni phosphine (0.20 mL, 0.4–6.0 μmol), the aryl bromide (0.10 mL, 0.20 mmol, 1.0 equiv) and the boronic substrate (0.10 mL, 0.22 mmol, 1.1 equiv) were then added to the vial. After that, the vial was sealed and removed from the glove box, then deionized water (80 μL) was added to the vial via a syringe. The reaction was placed in a shaker and heated to 60 °C. The agitation speed was set to 800 rpm. The reaction progress was monitored by preparing a series of identical samples (#s = 10). At the specific time point, a sample was removed from the shaker. Each sample was extracted by ethyl acetate, the resulting organic layer was collected, washed with water. An aliquot of the organic layer was analyzed by GC. Reactions were conducted in duplicates, and determined concentrations have a ±2% error bar in replicate runs.

3.2 Variable Time Normalization Analysis (VTNA) results

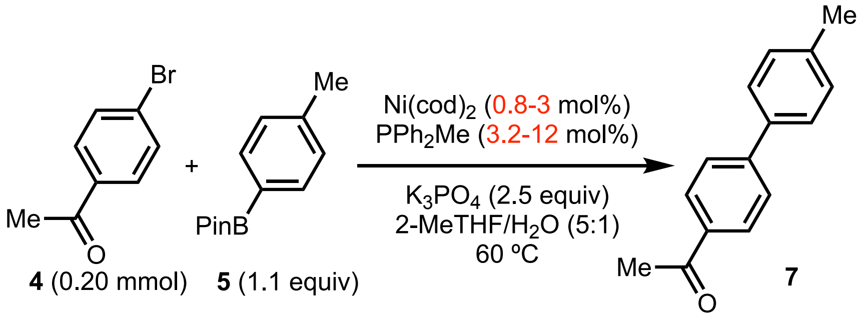

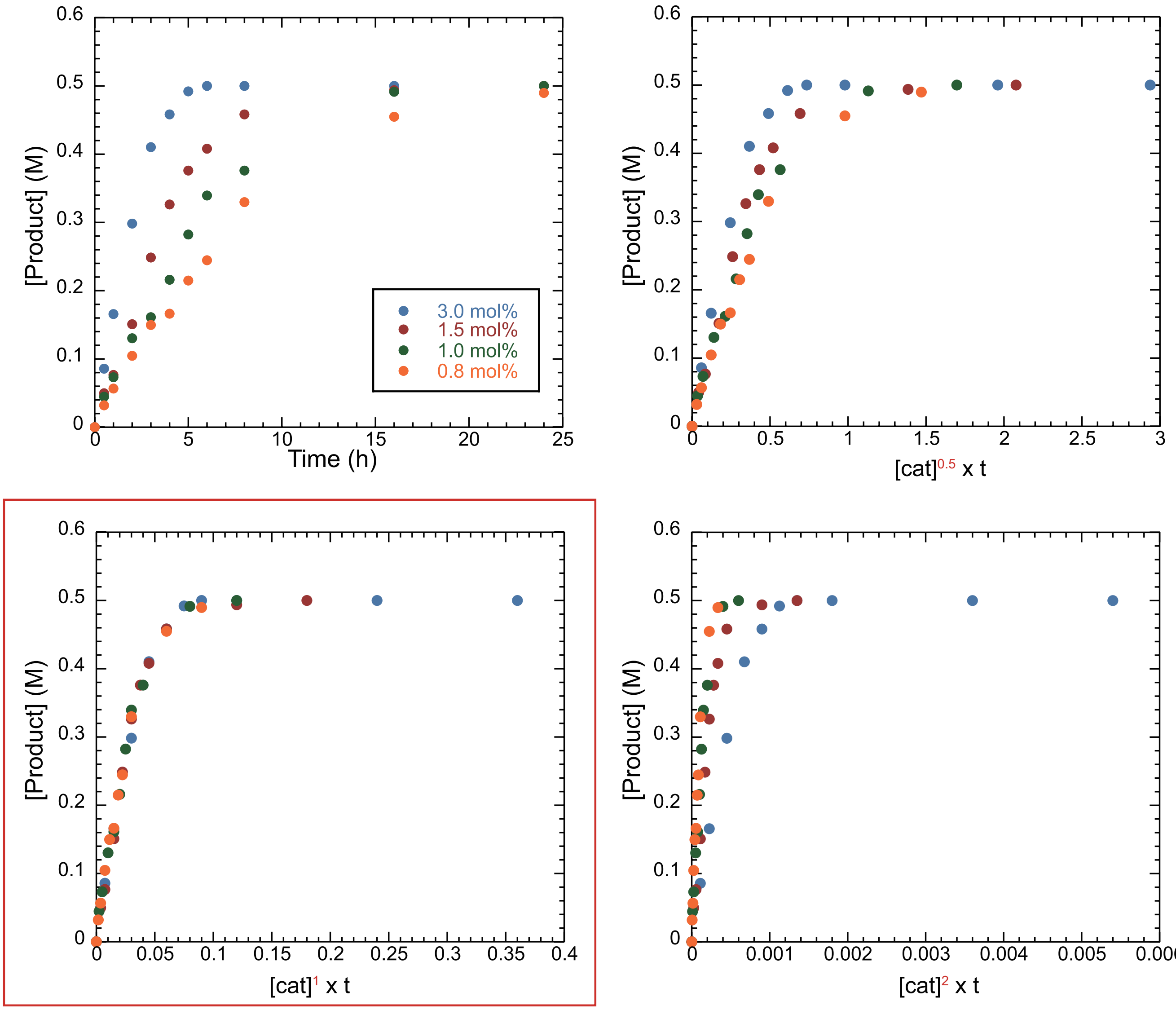

Figure S29 VTNA for the reaction rate dependence on [Ni] for the cross-coupling of 4-bromoacetophenone (**4**) and B(*p*-Tol)Pin (**5**) using $Ni(cod)_2$ and PPh_2Me. Original plot is top-left, time normalization plots show that there is a first-order dependence on [Ni] (red square). Condition: [**4**] = 0.5 M, [**5**] = 0.55 M, 60 °C.

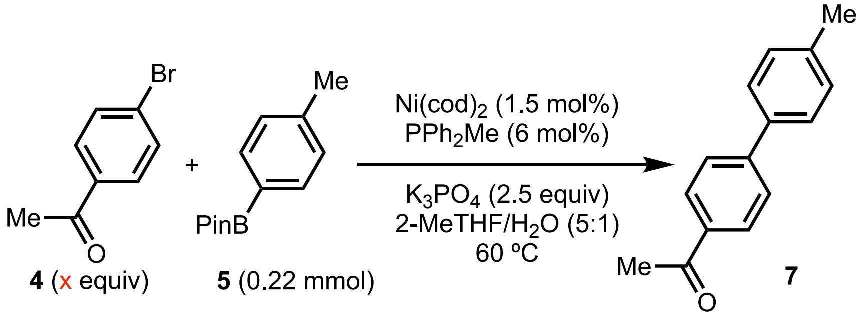

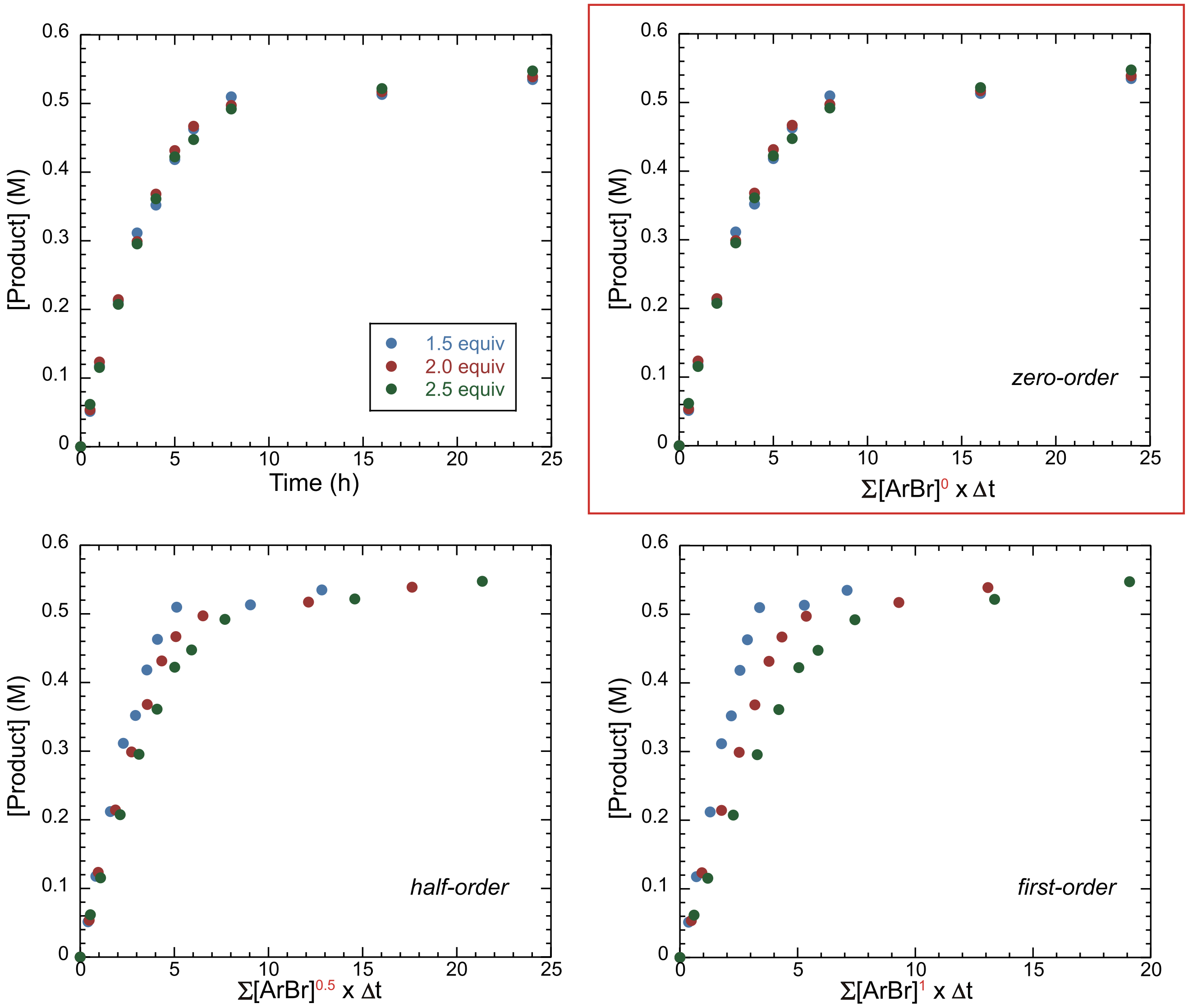

Figure S30 VTNA for the reaction rate dependence on [4-bromoacetophenone] for the cross-coupling of 4-bromoacetophenone (**4**) and B(*p*-Tol)Pin (**5**) using 1.5 mol% Ni(cod)$_2$ and 6 mol% PPh$_2$Me. Original plot is top-left, time normalization plots show that there is a zero-order dependence on [**4**] (red square). Condition: [**5**] = 0.55 M, 60 °C.

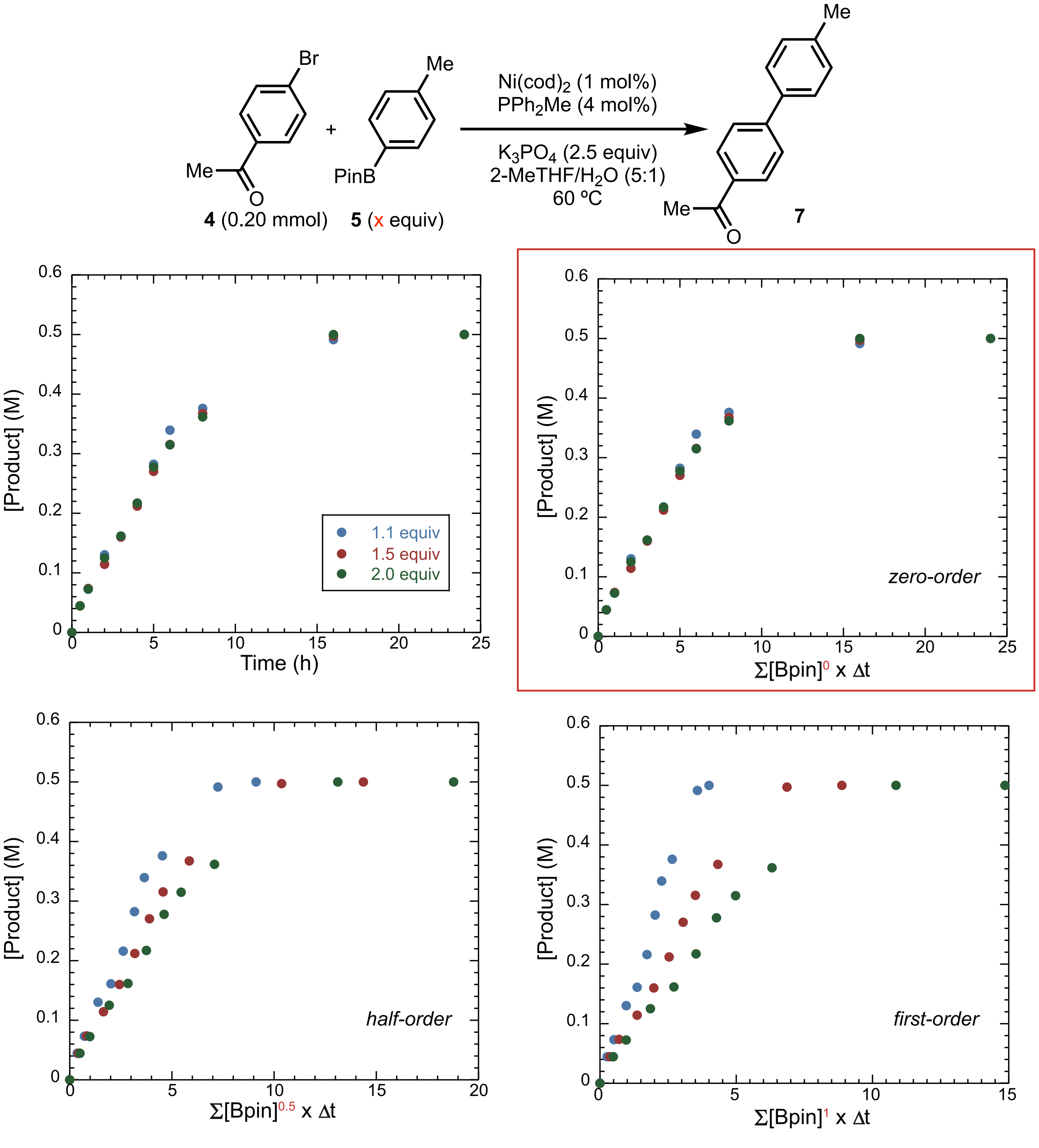

Figure S31 VTNA for the reaction rate dependence on [B(p-Tol)Pin] for the cross-coupling of 4-bromoacetophenone (**4**) and B(p-Tol)Pin (**5**) using 1 mol% Ni(cod)$_2$ and 4 mol% PPh$_2$Me. Original plot is top-left, time normalization plots show that there is a zero-order dependence on [**5**] (red square). Condition: [**4**] = 0.5 M, 60 °C.

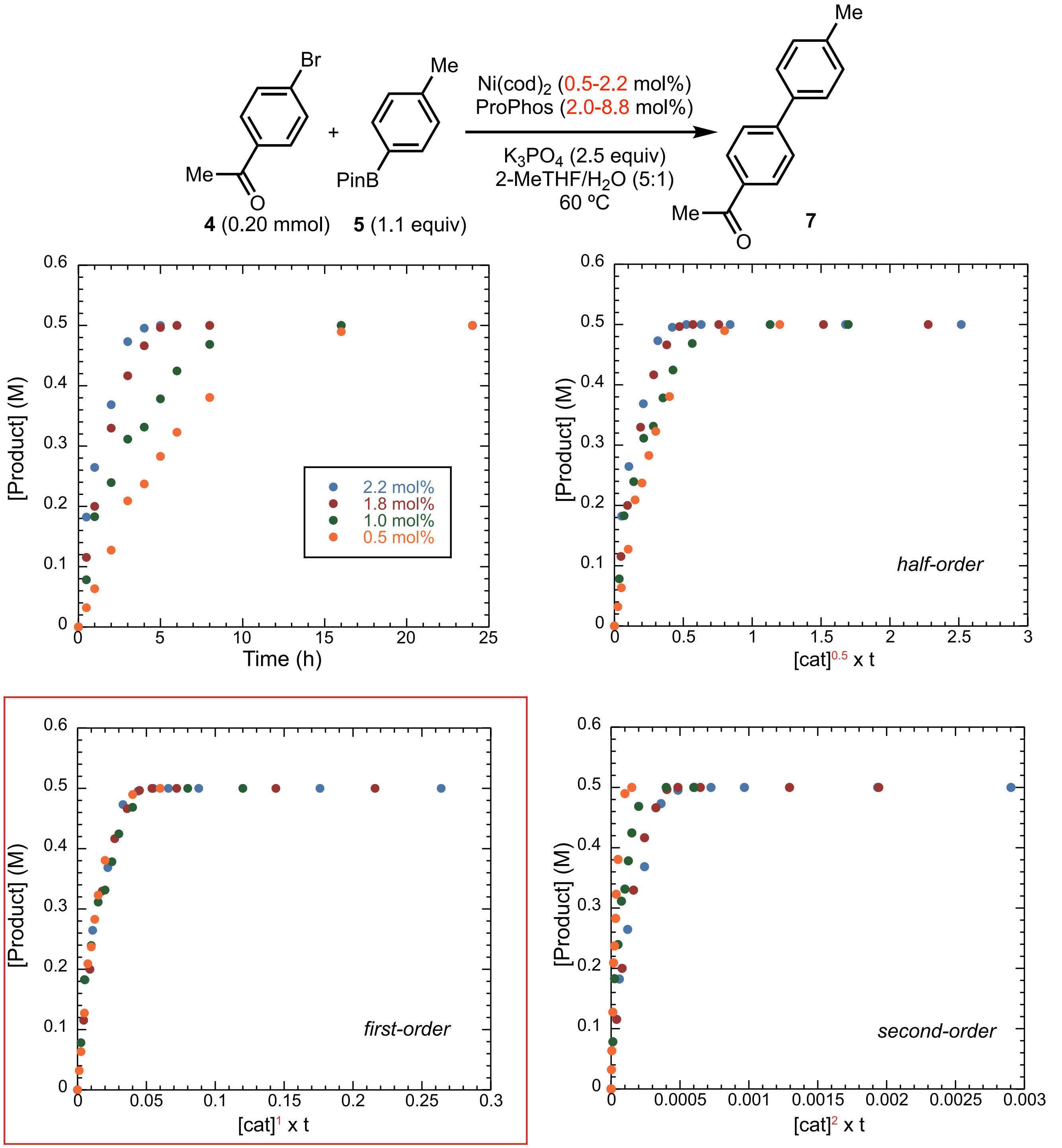

Figure S32 VTNA for the reaction rate dependence on [Ni] for the cross-coupling of 4-bromoacetophenone (**4**) and B(*p*-Tol)Pin (**5**) using Ni(cod)$_2$ and ProPhos. Original plot is top-left, time normalization plots show that there is a first-order dependence on [Ni] (red square). Condition: [**4**] = 0.5 M, [**5**] = 0.55 M, 60 °C.

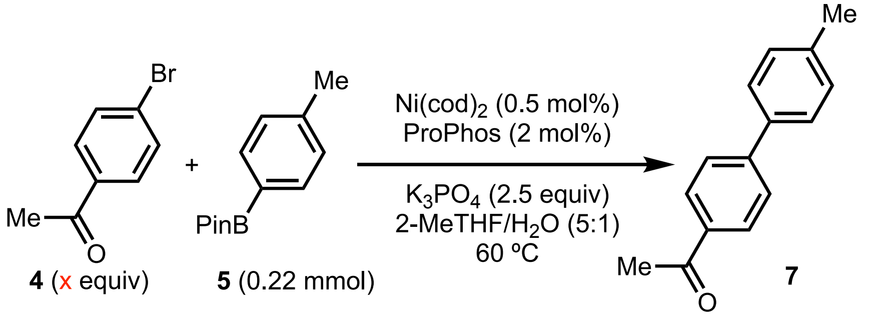

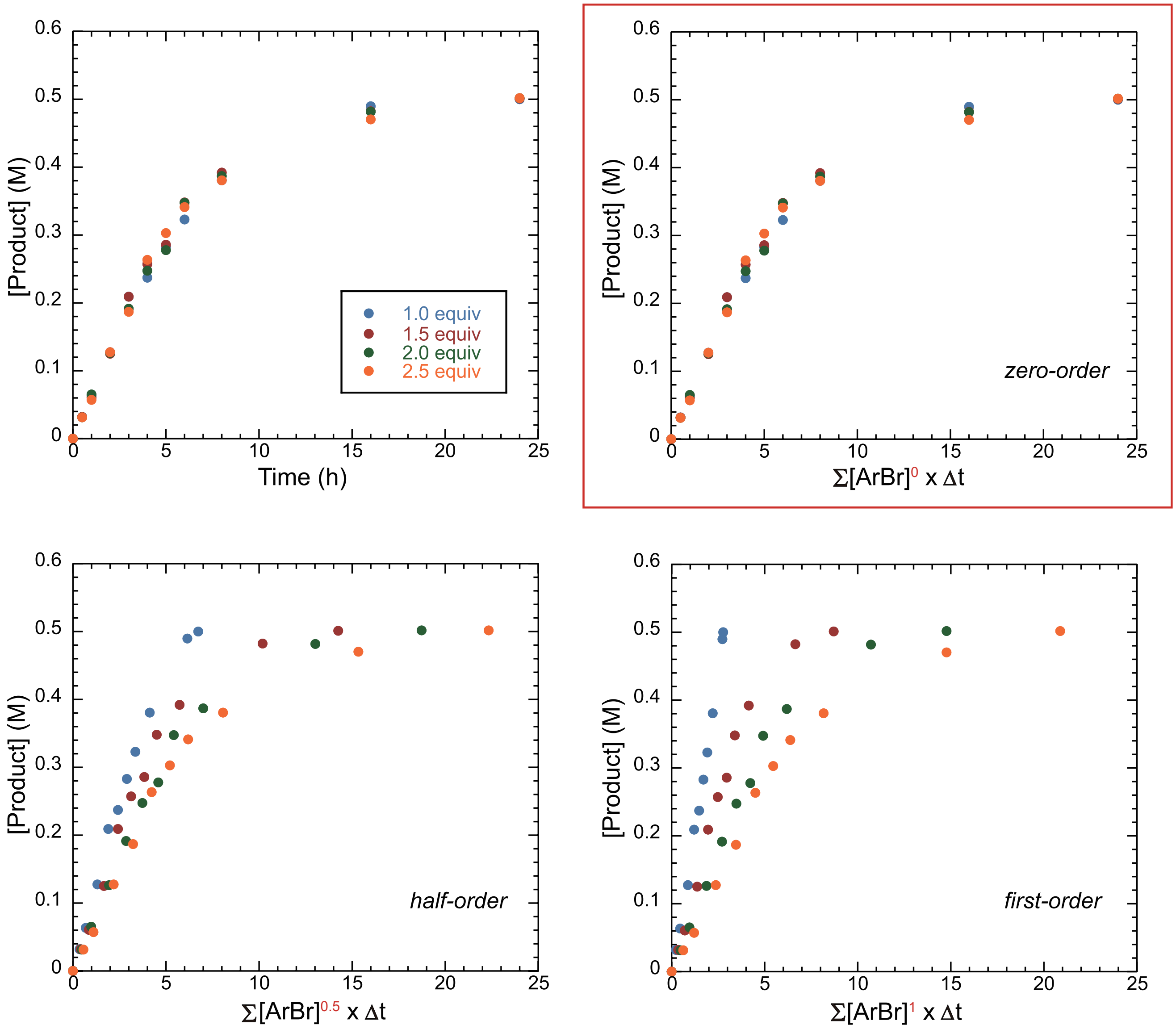

Figure S33 VTNA for the reaction rate dependence on [4-bromoacetophenone] for the cross-coupling of 4-bromoacetophenone (**4**) and B(*p*-Tol)Pin (**5**) using 0.5 mol% Ni(cod)$_2$ and 2 mol% ProPhos. Original plot is top-left, time normalization plots show that there is a zero-order dependence on [**4**] (red square). Condition: [**5**] = 0.55 M, 60 °C.

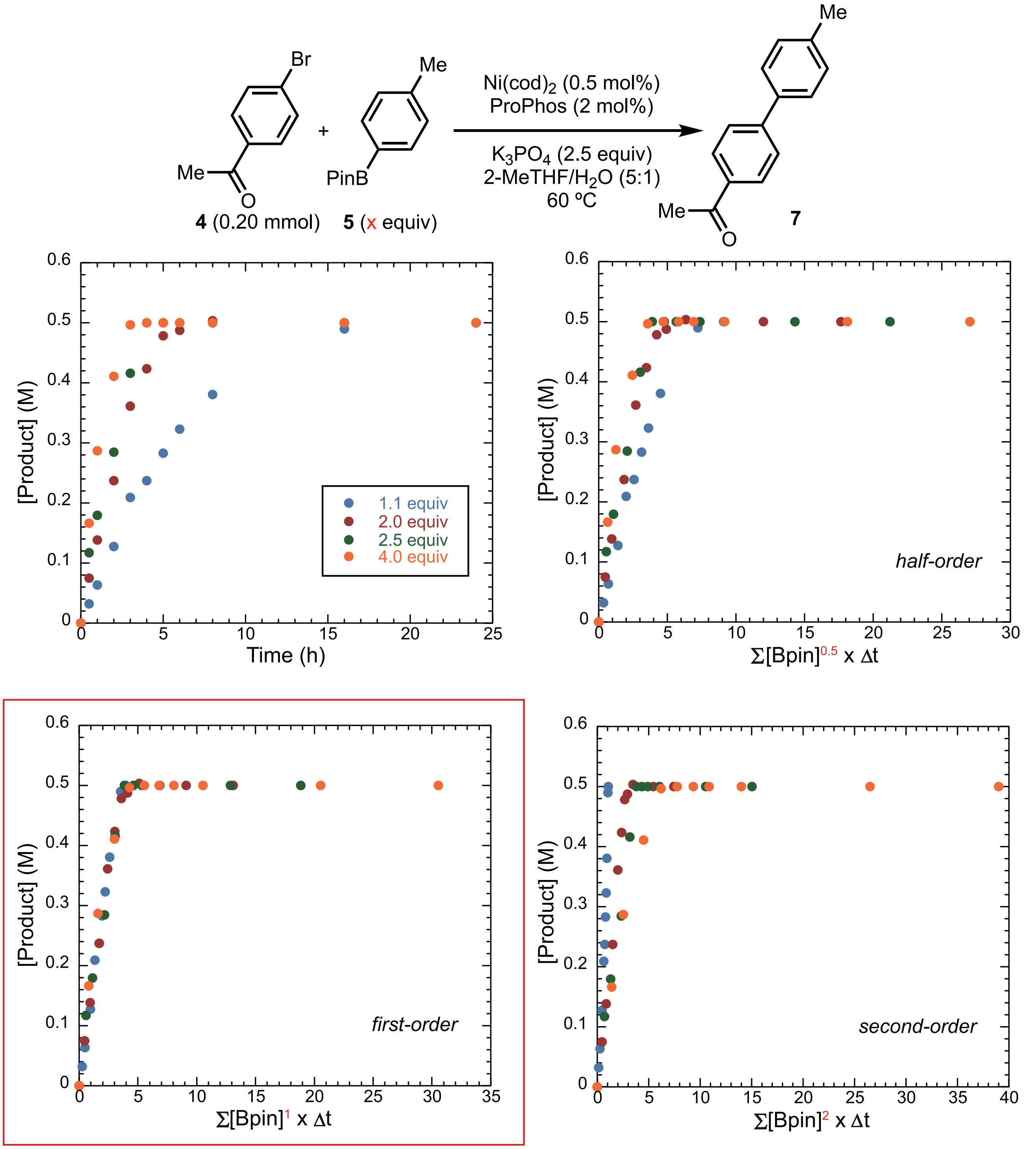

Figure S34 VTNA for the reaction rate dependence on [B(*p*-Tol)Pin] for the cross-coupling of 4-bromoacetophenone (**4**) and B(*p*-Tol)Pin (**5**) using 0.5 mol% $Ni(cod)_2$ and 2 mol% ProPhos. Original plot is top-left, time normalization plots show that there is a first-order dependence on [**5**] (red square). Condition: [**4**] = 0.5 M, 60 °C.

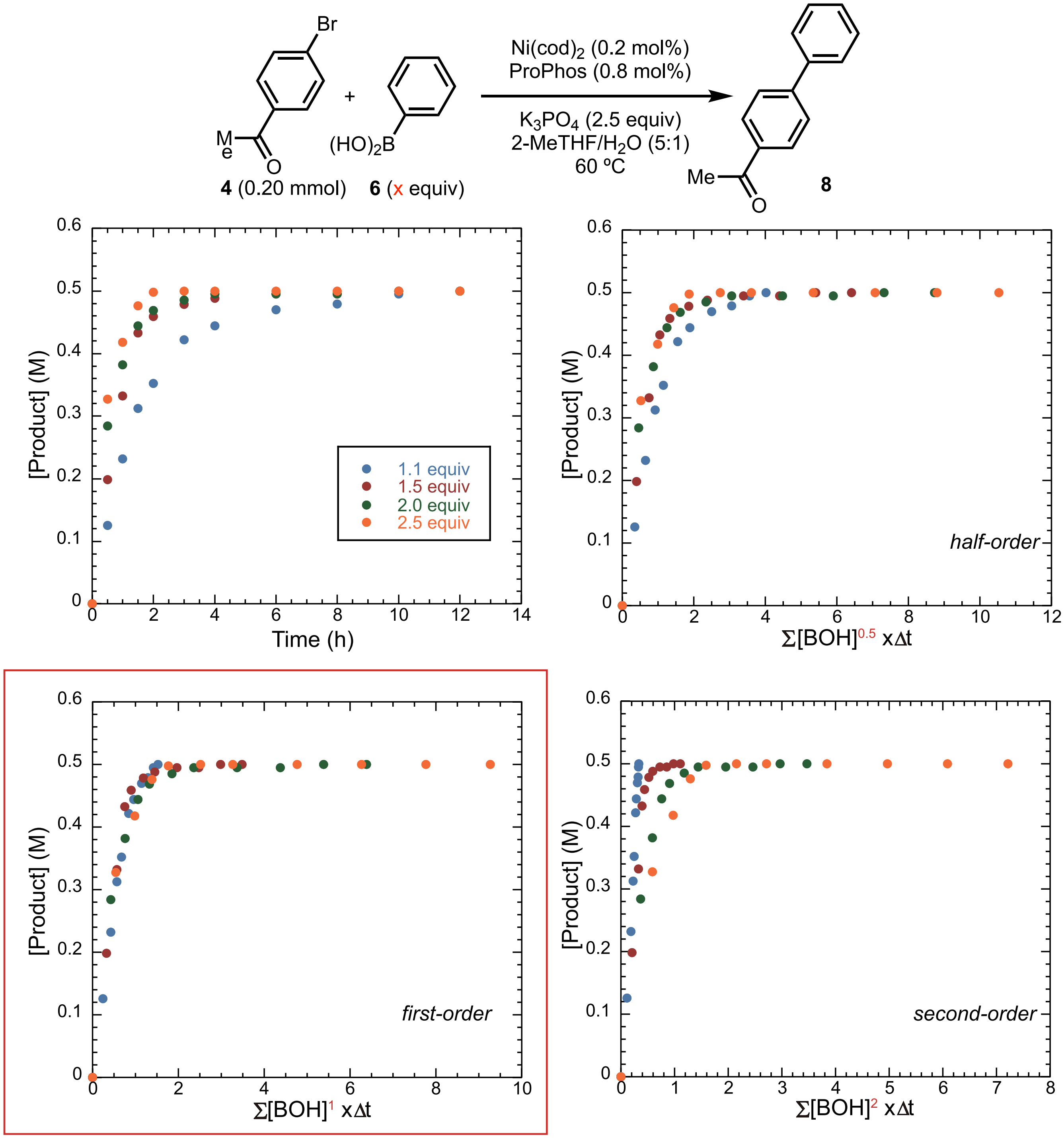

Figure S35 VTNA for the reaction rate dependence on [phenylboronic acid] for the cross-coupling of 4-bromoacetophenone (**4**) and $BPh(OH)_2$ (**6**) using 0.2 mol% $Ni(cod)_2$ and 0.8 mol% ProPhos. Original plot is top-left, time normalization plots show that there is a first-order dependence on [**6**] (red square). Condition: [**4**] = 0.5 M, 60 °C.

3.3 Effect of potential hydrolysis of B(*p*-Tol)Pin (5) during catalysis

We compared the rate of hydrolysis of B(*p*-Tol)Pin (**5**) with the catalytic conversion of **5** (Figure S36). The hydrolysis of B(*p*-Tol)Pin (**5**) in the catalytic solvents and temperature is slower (e.g. 40% at 24 h) than the catalytic conversion. At the 24-hour mark, we examined the boron speciation in both the organic (2-MeTHF) and aqueous phases. In the organic phase, toluene is identified via GCMS. Boron species isolated from the organic phase reveals only the presence of **5**, with no detection of B(*p*-Tol)$(OH)_2$ by NMR. Isolation of the boron product in the aqueous phase indicates a trace amount of KB[(*p*-Tol)Pin(OH)] (**15**). Overall, the hydrolysis of the boronic ester under basic conditions appears to be complex, involving protodeboronation, aligning with recent findings by Lloyd-Jones and colleagues.[6] While we cannot rule out the potential transient formation of B(*p*-Tol)$(OH)_2$ under catalytic conditions, the major boron species in the organic phase remains the boronic ester **5**.

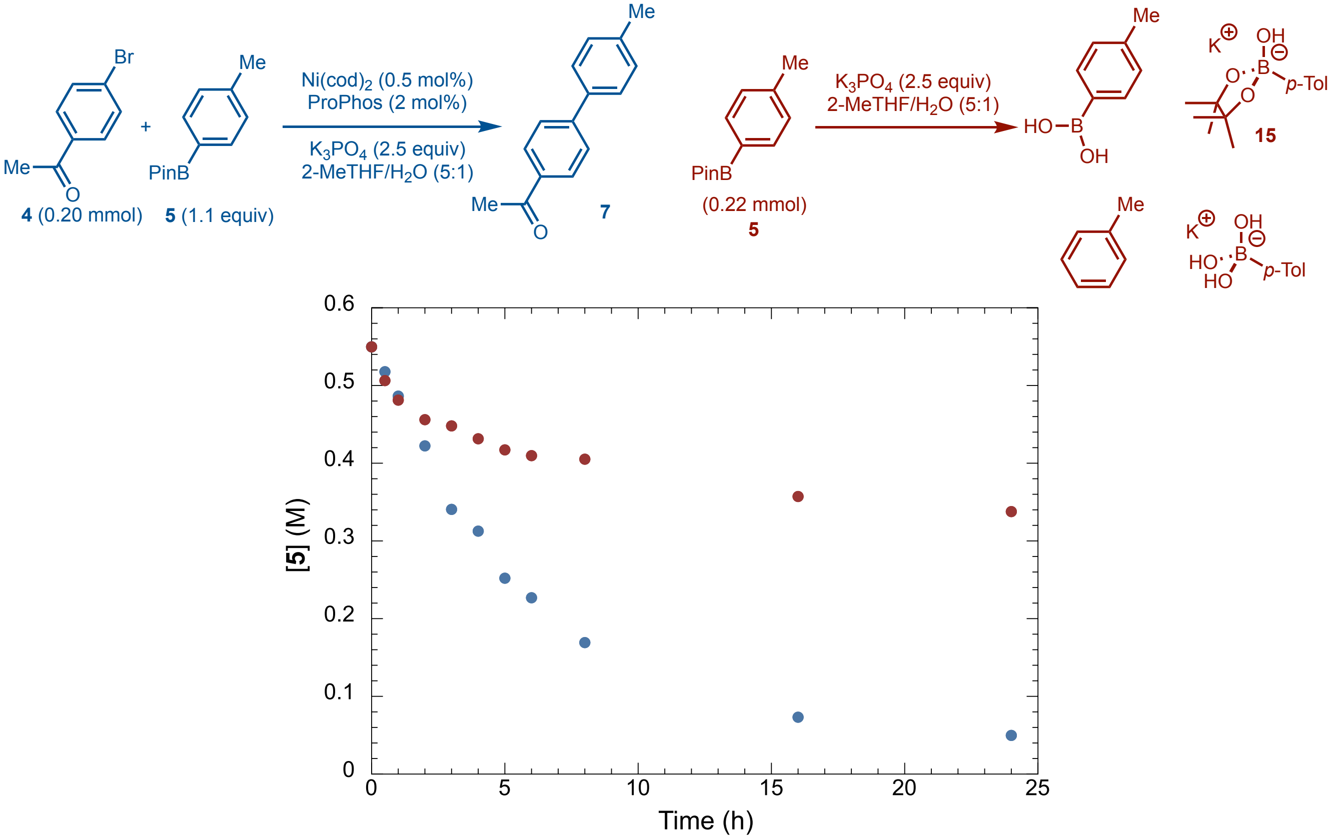

Figure S36 Comparison between the SMC of 4-bromoacetophenone (**4**) and B(*p*-Tol)Pin (**5**) using 0.5 mol% Ni(cod)$_2$ and 2 mol% ProPhos and the "hydrolysis" of B(*p*-Tol)Pin (**5**) without the electrophile **4** and the catalyst. Condition: [**4**] = 0.5 M, 60 °C.

[6] Hayes, H. L. D.; Wei, R.; Assante, M.; Geogheghan, K. J.; Jin, N.; Tomasi, S.; Noonan, G.; Leach, A. G.; Lloyd-Jones, G. C. Protodeboronation of (Hetero)Arylboronic Esters: Direct versus Prehydrolytic Pathways and Self-/Auto-Catalysis. *J. Am. Chem. Soc.* **2021**, *143* (36), 14814–14826.

4. Synthesis of Ni complexes

4.1 Synthesis of $Ni(PPh_2CH_2CH_2CH_2OH)_2(cod)$ (11)

A vial was charged with $Ni(cod)_2$ (50.0 mg, 0.182 mmol, 1.0 equiv), $PPh_2CH_2CH_2CH_2OH$ (88.8 mg, 0.364 mmol, 2.0 equiv), THF (4 mL). The mixture was stirred at 20 °C overnight. The red solution was filtered through a Celite. The filtrate was concentrated under vacuum, layered with pentane (5 mL), and stored at -35 °C overnight to give an orange crystal (74.0 mg, 0.113 mmol, 62%). The complex contained two equivalents of co-crystallized THF, as detected by both X-ray and NMR analyses.

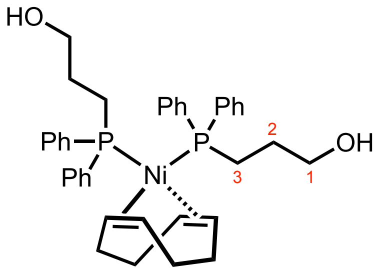

11

^{1}H NMR (400.30 MHz, C_6D_6) δ: 7.44 (ddt, *J* = 7.9, 5.2, 2.3 Hz, 8H, H_o), 7.07 (qd, *J* = 4.3, 2.0 Hz, 12H, H_m & H_p), 4.57 – 4.45 (m, 4H, CH at cod), 3.13 (t, *J* = 6.2 Hz, 4H, H1), 2.23 (dt, *J* = 12.0, 3.0 Hz, 4H, H3), 2.03 – 1.80 (m, 8H, CH_2 at cod), 1.48 – 1.42 (m, 4H, H2 overlapping with THF signals), 0.92 (br s, 2H, OH overlapping with pentane signals).

^{31}P{^{1}H} NMR (162.04 MHz, C_6D_6) δ: 31.7 (s).

^{13}C{^{1}H} NMR (100.67 MHz, C_6D_6) δ: 140.1 (dd, *J* = 23, 4 Hz, C_{ipso}), 133.7 (d, *J* = 12 Hz, C_o), 128.6 (s, C_p, overlapping with C_6D_6 signals and detected by ^{13}C DEPT 135), 128.5 (d, *J* = 8 Hz, C_m, overlapping with C_6D_6 signals and detected by ^{13}C DEPT 135), 83.4 (t, *J* = 6 Hz, CH at cod), 83.3 (t, *J* = 6 Hz, CH at cod), 64.2 (d, *J* = 11 Hz, C1), 31.1 – 30.9 (m, CH_2 at cod), 30.2 – 29.9 (m, C2), 29.6 (s, C3).

HRMS (ESI-TOF, CH_3CN) m/z: $[M + K]^+$ calculated for $C_{38}H_{46}NiO_2P_2K$ 693.1958, found 693.1989.

4.2 Attempted synthesis of $Ni(PPh_2CH_2CH_2CH_2OH)_4$ (12)

A vial was charged with $Ni(cod)_2$ (40.0 mg, 0.145 mmol, 1.0 equiv), $PPh_2CH_2CH_2CH_2OH$ (142 mg, 0.582 mmol, 4.0 equiv), THF (4 mL). The mixture was stirred at 20 °C overnight. The red suspension was filtered through a Celite. To the filtrate, pentane (5 mL) was added to precipitate a dark orange solid. The solid was washed with pentane (3 x 3 mL) and further dried under vacuum for 2 h to afford an orange solid (65 mg), which contained a mixture of $Ni(PPh_2CH_2CH_2CH_2OH)_4$ (**12**, 80%) and $Ni(PPh_2CH_2CH_2CH_2OH)_2(cod)$ (**11**, 20%), as determined by ^{31}P{^{1}H} NMR (Figure S36).

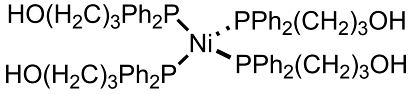

12

*Partial characterization data of complex **12***

$^{31}P\{^1H\}$ NMR (162.04 MHz, C_6D_6) δ: 12.7 (br s, $\omega_{1/2}$ = 724 Hz).

$^{31}P\{^1H\}$ NMR (162.04 MHz, C_6D_6:THF = 1:4) δ: 13.2 (br s, $\omega_{1/2}$ = 410 Hz).

HRMS (ESI-TOF, CH_3OH) m/z:

$[M + O + 2K]^{2+}$ calculated for $C_{60}H_{68}NiO_5P_4K_2$ 564.1317, found 564.1284;

$[M – 1\ ligand + O + Na]^{+}$ calculated for $C_{45}H_{51}NaNiO_4P_3$ 829.2246, found 829.2248;

$[M – 2\ ligands + O + NH_4]^{+}$ calculated for $C_{30}H_{38}NniO_3P_2$ 580.1675, found 580.1671;

$[M – 3\ ligands + O + Na]^{+}$ calculated for $C_{15}H_{17}NaNiO_2P$ 341.0212, found 340.0242.

Only Ni complex containing one phosphine oxide is detected by HRMS due to the highly air-sensitive nature of this complex.

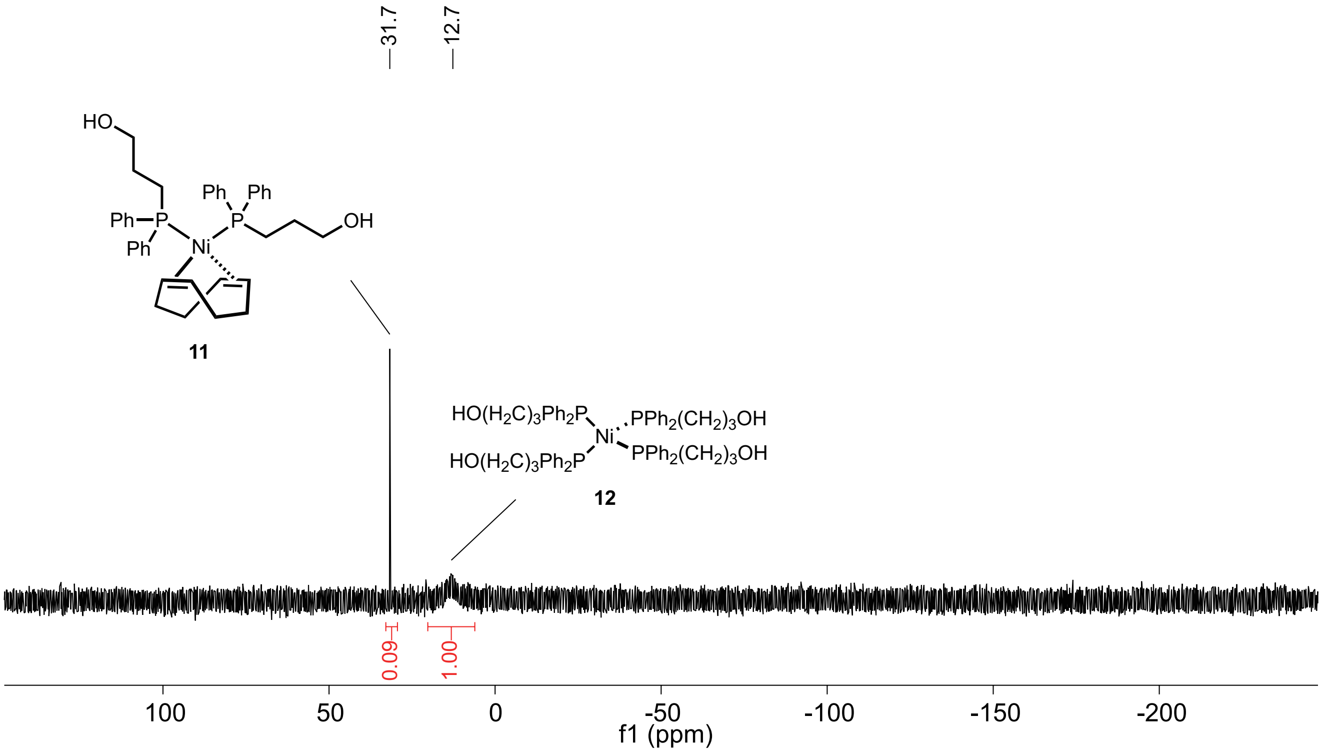

Figure S37 $^{31}P\{^1H\}$ NMR (162.04 MHz, C_6D_6) spectrum of the crude mixture containing complexes $Ni(PPh_2CH_2CH_2CH_2OH)_4$ (**12**) and $Ni(PPh_2CH_2CH_2CH_2OH)_2(cod)$ (**11**).

The NMR signals of ^1H, $^{31}P\{^1H\}$, and $^{13}C\{^1H\}$ associated with complex **12** exhibit significant broadening, leading us to exclusively report the $^{31}P\{^1H\}$ NMR data. Plausible rationales include the limited solubility of this complex in C_6D_6 and/or the occurrence of dynamic processes in solution, such as ligand dissociation and re-coordination. To address the solubility concern, we employed 0.4 mL of THF and 0.1 mL of C_6D_6 (locking purposes) for dissolve this complex (Figure S38), but the broad signal attributed to **12** persisted, hinting at the potential validity of the latter hypothesis.

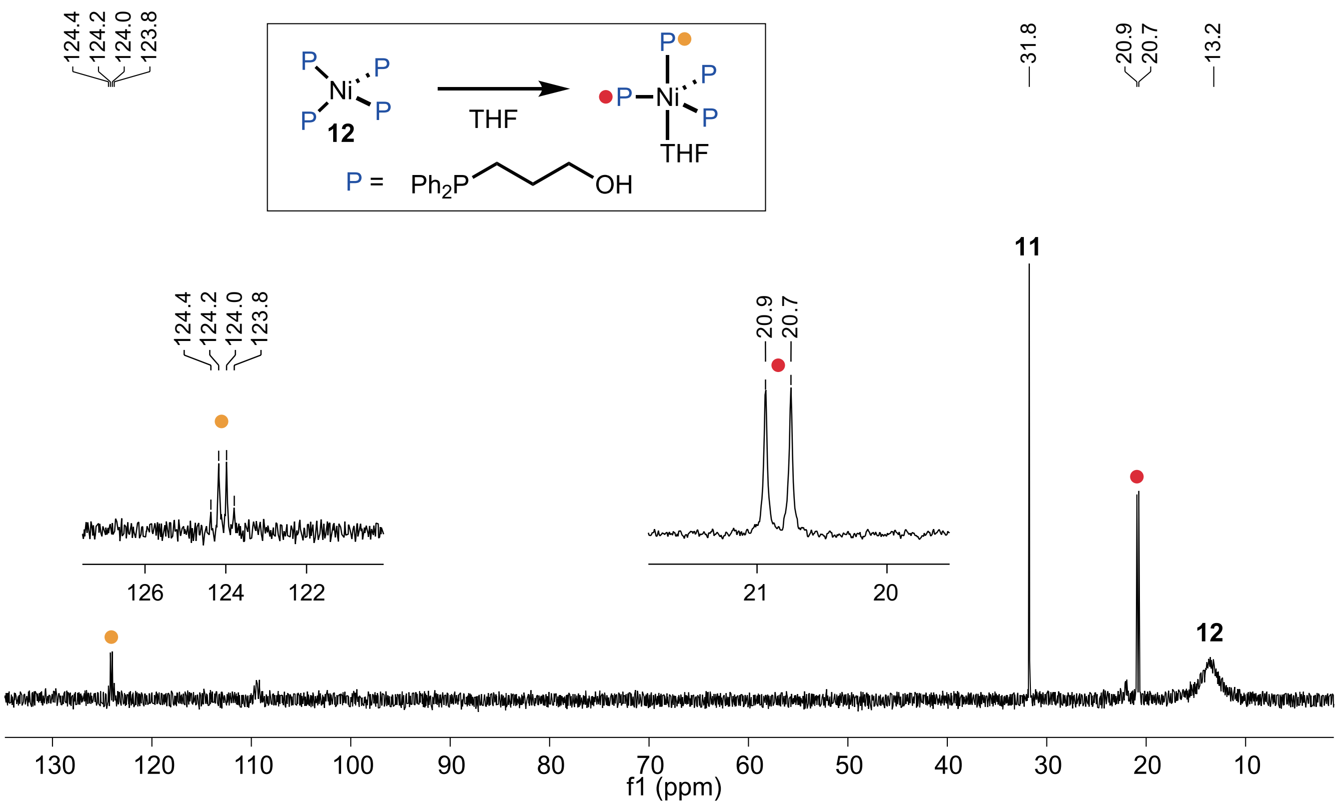

Figure S38 $^{31}P\{^{1}H\}$ NMR (162.04 MHz, C_6D_6:THF = 1:4) spectrum of the crude mixture containing $Ni(PPh_2CH_2CH_2CH_2OH)_4$ (**12**), $Ni(PPh_2CH_2CH_2CH_2OH)_2(cod)$ (**11**), $Ni(PPh_2CH_2CH_2CH_2OH)_4(THF)$ and other unidentified species.

Furthermore, when complex **12** was in the presence of excess THF (close to catalytic conditions), a new Ni species emerged. This species is tentatively identified as the bipyramidal complex $Ni(PPh_2CH_2CH_2CH_2OH)_4(THF)$. The basis for this assignment lies in the diagnostic multiplicity observed in the $^{31}P\{^{1}H\}$ signals (δ: 124.1 ppm (q, J = 31 Hz, integration = 1), 20.8 ppm (d, J = 31 Hz, integration = 3)), providing further support for our proposed characterization.

4.3 Synthesis of Ni(PPh$_2$Me)$_2$Cl(*o*-Tol) (13)

A Schlenk flask was charged with $Ni(cod)_2$ (100 mg, 0.364 mmol, 1.0 equiv), THF (5 mL), 2-chlorotoluene (460 mg, 3.64 mmol, 10 equiv), and PPh_2Me (291 mg, 1.45 mmol, 4.0 equiv). The reaction mixture was stirred at 60 °C for 5 days, then cooled to rt and concentrated under vacuum to give an orange oil. The orange oil was dissolved in a minimal MeOH and layered with hexane to give a dark red crystal (102 mg, 0.174 mmol, 48%). Spectroscopic data are consistent with the literature reports.[7]

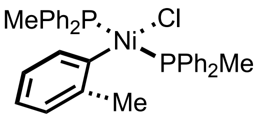

13

[7] Standley, E. A.; Smith, S. J.; Müller, P.; Jamison, T. F. A Broadly Applicable Strategy for Entry into Homogeneous Nickel(0) Catalysts from Air-Stable Nickel(II) Complexes. *Organometallics* **2014**, *33* (8), 2012–2018.

^{1}H NMR (400.30 MHz, C_6D_6) δ: 7.84 (ddt, *J* = 7.4, 5.4, 3.4 Hz, 4H, H_o at Ph), 7.62 (qd, *J* = 4.9, 2.5 Hz, 4H, H_o at Ph), 7.13 – 6.92 (m, 13H, H_m, H_p at Ph & *o*-Tol), 6.75 – 6.64 (m, 1H, *o*-Tol), 6.59 (d, *J* = 7.8 Hz, 2H, *o*-Tol), 2.75 (s, 3H, CH_3 at *o*-Tol), 1.08 (t, *J* = 3.6 Hz, 6H, CH_3 at PPh_2Me).
$^{31}P\{^1H\}$ NMR (162.04 MHz, C_6D_6) δ: 7.6 (s).
$^{13}C\{^1H\}$ NMR (100.67 MHz, C_6D_6) δ: 153.1 (t, *J* = 34 Hz, *o*-Tol), 143.3 (d, *J* = 7 Hz, *o*-Tol), 136.1, (t, *J* = 5 Hz, *o*-Tol), 135.01 (t, *J* = 20 Hz, C_{ipso} at Ph), 133.9 (t, *J* = 6 Hz, C_o at Ph), 133.5 (t, J = 21 Hz, C_{ipso} at Ph), 133.3 (t, *J* = 5 Hz, C_o at Ph), 129.9 (s, C_p at Ph), 129.5 (s, C_p at Ph), 128.3 (t, *J* = 5 Hz, C_m at Ph), 128.1 (t, *J* = 5 Hz, C_m at Ph), 124.0 (t, *J* = 3 Hz, *o*-Tol), 122.6 (t, *J* = 3 Hz, *o*-Tol), 26.6 (CH_3 at *o*-Tol), 12.9 (t, *J* = 16 Hz, CH_3 at PPh_2Me).
HRMS (ESI-TOF, CH_3CN) m/z: $[M - Cl]^+$ calculated for $C_{33}H_{33}NiP_2$ 549.1405, found 549.1414. No HRMS was reported in literature.

4.4 Synthesis of Ni(PPh_2Me)$_2$Br(*o*-Tol) (14)

A Schlenk flask was charged with $Ni(acac)_2$ (400 mg, 1.56 mmol, 1.0 equiv), THF (10 mL), and PPh_2Me (686 mg, 3.42 mmol, 2.2 equiv). The suspension was cooled to 0 °C with an ice bath and o-tolylmagnesium bromide (1.56 mmol, 0.68 M in THF, 2.3 mL) was added dropwise with vigorous stirring. The solution began to turn orange at the end of the addition. The solution was stirred for 30 min at 0 °C. After that, the solvent was removed under vacuum and MeOH (5 mL) was added. The mixture was sonicated and stored at -35 °C for 2 h to give a yellow suspension. The yellow precipitate was collected by vacuum filtration, washed with cold MeOH (3 x 10 mL), and dried under vacuum to give an orange-yellow solid (447 mg, 0.709 mmol, 46%). Spectroscopic data are consistent with the literature reports.[8] Note: MeOH is important to remove $Mg(acac)_2$ and precipitate out the complex. However, the complex is partially soluble in MeOH, leading to the low yield.

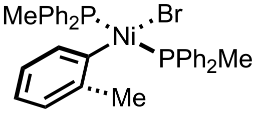

14

^{1}H NMR (400.30 MHz, C_6D_6) δ: 7.81 (qt, *J* = 5.3, 2.4 Hz, 4H, H_o at Ph), 7.63 (ddt, *J* = 9.5, 4.8, 2.5 Hz, 4H, H_o at Ph), 7.13 – 6.96 (m, 13H, H_m, H_p at Ph & *o*-Tol), 6.67 (td, *J* = 7.2, 1.6 Hz, 1H, *o*-Tol), 6.62 – 6.54 (m, 2H, *o*-Tol), 2.76 (s, 3H, CH_3 at *o*-Tol), 1.13 (t, *J* = 3.7 Hz, 6H, CH_3 at PPh_2Me).
$^{31}P\{^1H\}$ NMR (162.04 MHz, C_6D_6) δ: 8.6 (s).
HRMS (ESI-TOF, CH_3CN) m/z: $[M - Br]^+$ calculated for $C_{33}H_{33}NiP_2$ 549.1405, found 549.1397. No HRMS was reported in literature.

[8] Haibach, M. C.; Ickes, A. R.; Tcyrulnikov, S.; Shekhar, S.; Monfette, S.; Swiatowiec, R.; Kotecki, B. J.; Wang, J.; Wall, A. L.; Henry, R. F.; Hansen, E. C. Enabling Suzuki–Miyaura Coupling of Lewis-Basic Arylboronic Esters with a Nonprecious Metal Catalyst. *Chem. Sci.* **2022**, *13* (43), 12906–12912.

4.5 Synthesis of [Ni(PPh_2Me)(μ-OH)(*o*-Tol)]$_2$ (16)

A vial was charged with Ni(PPh_2Me)$_2$X(*o*-Tol) (**13**, X = Cl: 50.0 mg or **14**, X = Br: 53.8 mg, 0.0854 mmol, 1.0 equiv), KOH (95.8 mg, 1.71 mmol, 20 equiv), THF (4 mL), and deionized H_2O (20 mL). The mixture was stirred at 20 °C overnight, then solvent was removed under vacuum to give a yellow oil containing a white solid. The mixture was extracted with pentane (3 x 5 mL), and further filtered through a Celite. The resulting yellow solution was concentrated under vacuum (~2 mL), and stored at -35 °C overnight to give a yellow solid. The solid was collected by filtration, and further dried under vacuum to afford a yellow solid (21.8 mg, 0.0297 mmol, 70%). This compound is extremely soluble in pentane, which makes it difficult to isolate it free of the free PPh_2Me. A trace amount of free PPh_2Me and its oxide are detected by ^{31}P NMR in the isolated mixture.

<u>*Characterization of the isomeric Ni hydroxo-bridged dinuclear complexes*</u>

The identity of the isolated mixture containing four isomeric Ni bridging OH complexes is supported by the correlations in the $^{1}H/^{31}P\{^{1}H\}$ HMBC spectrum (Figure S39). Two most intense signals at 17.2 and 17.9 ppm are assigned to two *trans*-isomers, which show the correlations with the bridging OH signals at -3.34 and -3.35 ppm (Figure S39a). These ^{1}H shifts are consistent with the literature reported for *trans*-analogues.[9] For two *cis*-isomers, we observed two sets of the bridging OH signals (four signals total) due to the symmetry. Two upfield signals at -5.31 and -5.51 ppm are assigned to the two OH *cis* to the PPh_2Me (Figure S39b). According to the $^{1}H/^{31}P\{^{1}H\}$ HMBC, we are able to assign the ^{31}P signals at 17.9 and 16.8 ppm due to the two *cis*-isomers. The two most downfield bridging OH signals at -1.75 and -1.87 ppm are assigned to the signals due to the OH *trans* to the PPh_2Me ligand for the *cis*-Ni complexes (*syn*- and *anti*-). In addition, four signals between 3.20 and 3.59 ppm are assigned to four CH_3 signals at the Ni-Tol fragment based on the $^{1}H/^{31}P\{^{1}H\}$ HMBC correlations (Figure S39c). However, we lack the spectroscopic evidence to distinguish the *syn*- and *anti*-isomers. Nevertheless, the *trans*-isomers are major species, which is consistent with the reported DFT calculation for their high stability.[10] Since the resting signals in these four isomers are close, we are not able to assign every ^{1}H and ^{13}C NMR signals.

[9] Christian, A. H.; Müller, P.; Monfette, S. Nickel Hydroxo Complexes as Intermediates in Nickel-Catalyzed Suzuki–Miyaura Cross-Coupling. *Organometallics* **2014**, *33* (9), 2134–2137.

[10] Payard, P.-A.; Perego, L. A.; Ciofini, I.; Grimaud, L. Taming Nickel-Catalyzed Suzuki-Miyaura Coupling: A Mechanistic Focus on Boron-to-Nickel Transmetalation. *ACS Catal.* **2018**, *8* (6), 4812–4823.

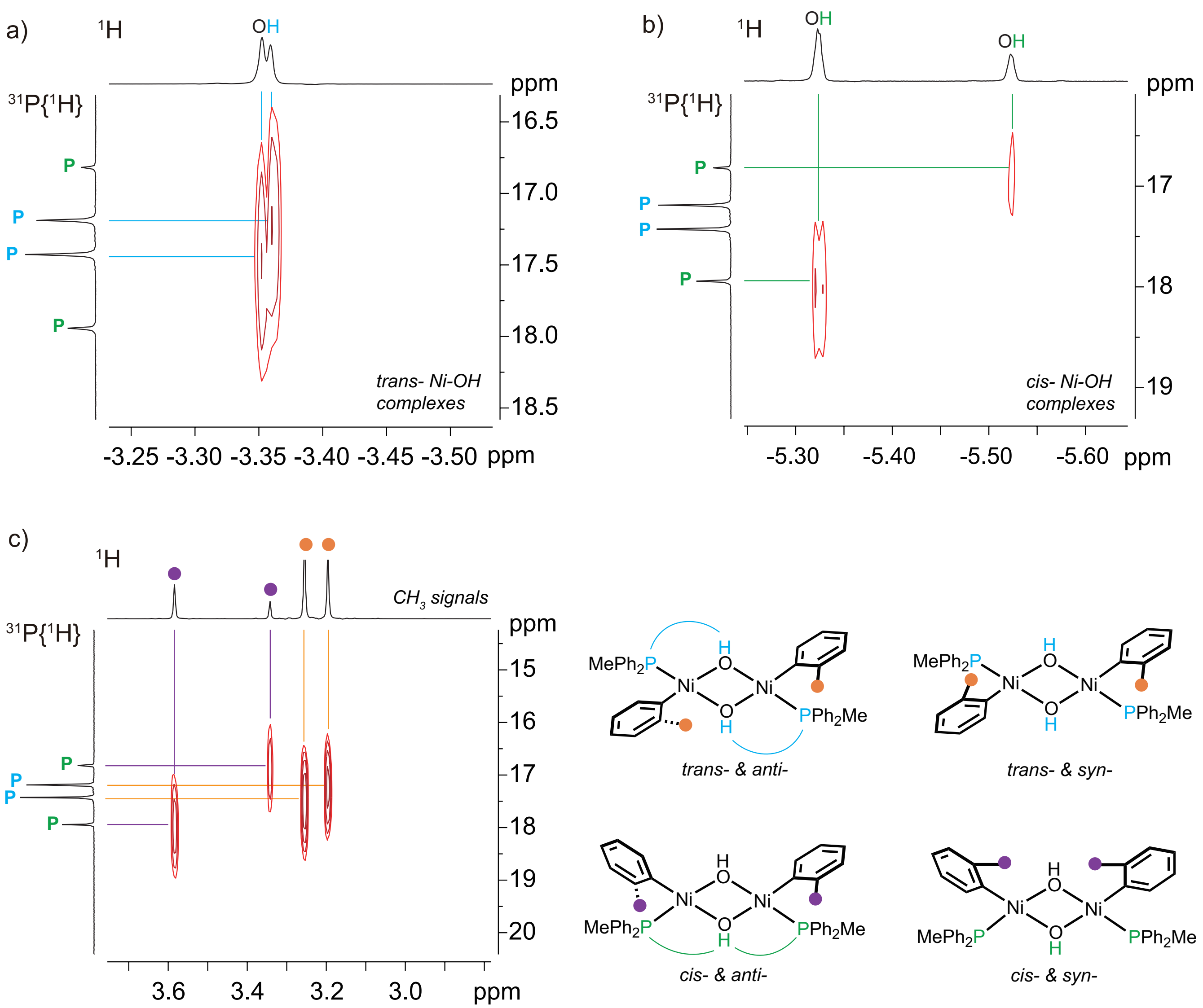

Figure S39 Partial $^{1}H/^{31}P\{^{1}H\}$-HMBC NMR (500.20 MHz, C_6D_6) spectra for the isolated [Ni(PPh_2Me)(μ-OH)(*o*-Tol)]$_2$ (**16**), showing the correlations in four isomeric Ni hydroxo-bridged dinuclear complexes.

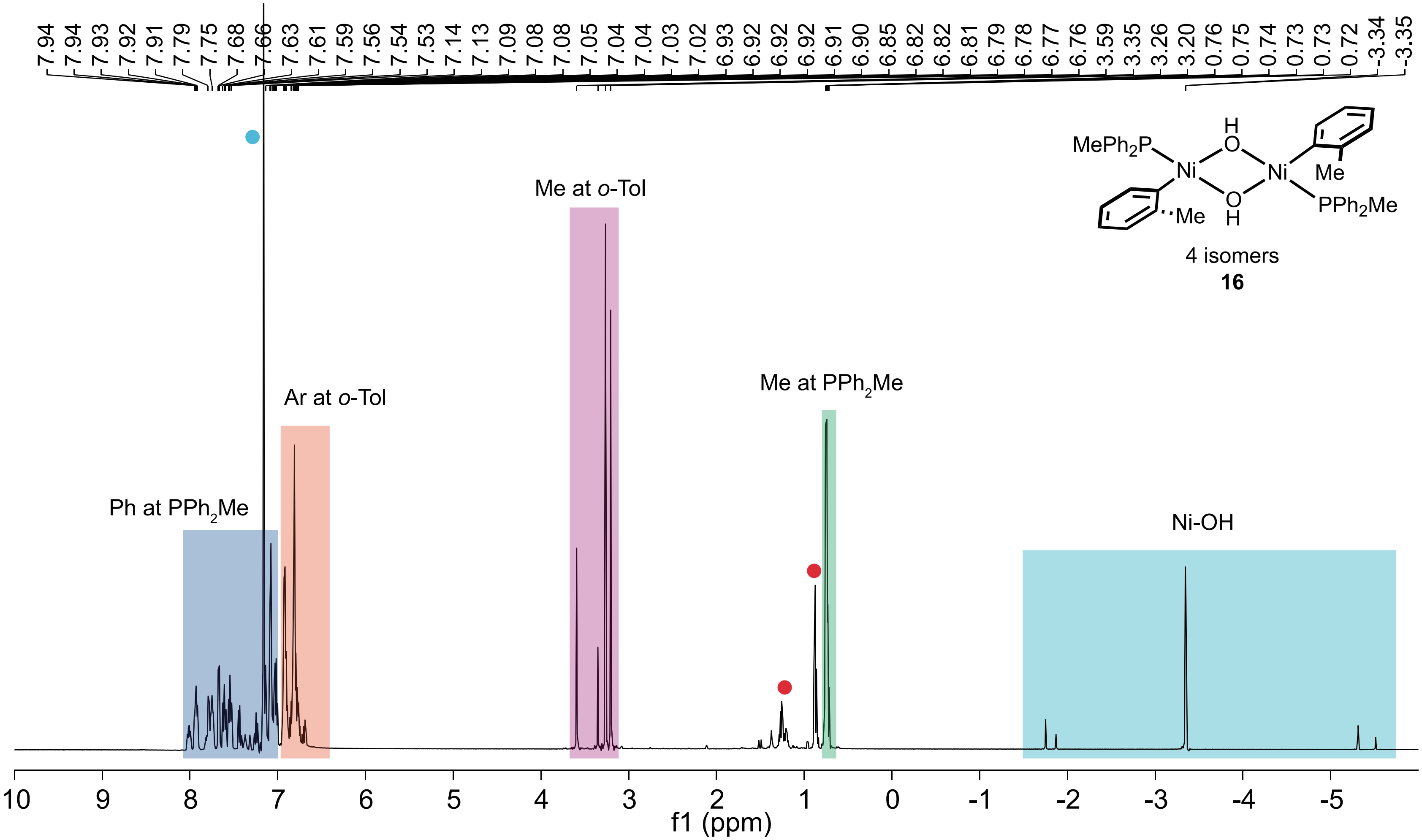

Figure S40 ^{1}H NMR (500.20 MHz, C_6D_6) spectrum of complex [Ni(PPh_2Me)(μ-OH)(*o*-Tol)]$_2$ (**16**). Residual proteo-solvent (•) and pentane (•).

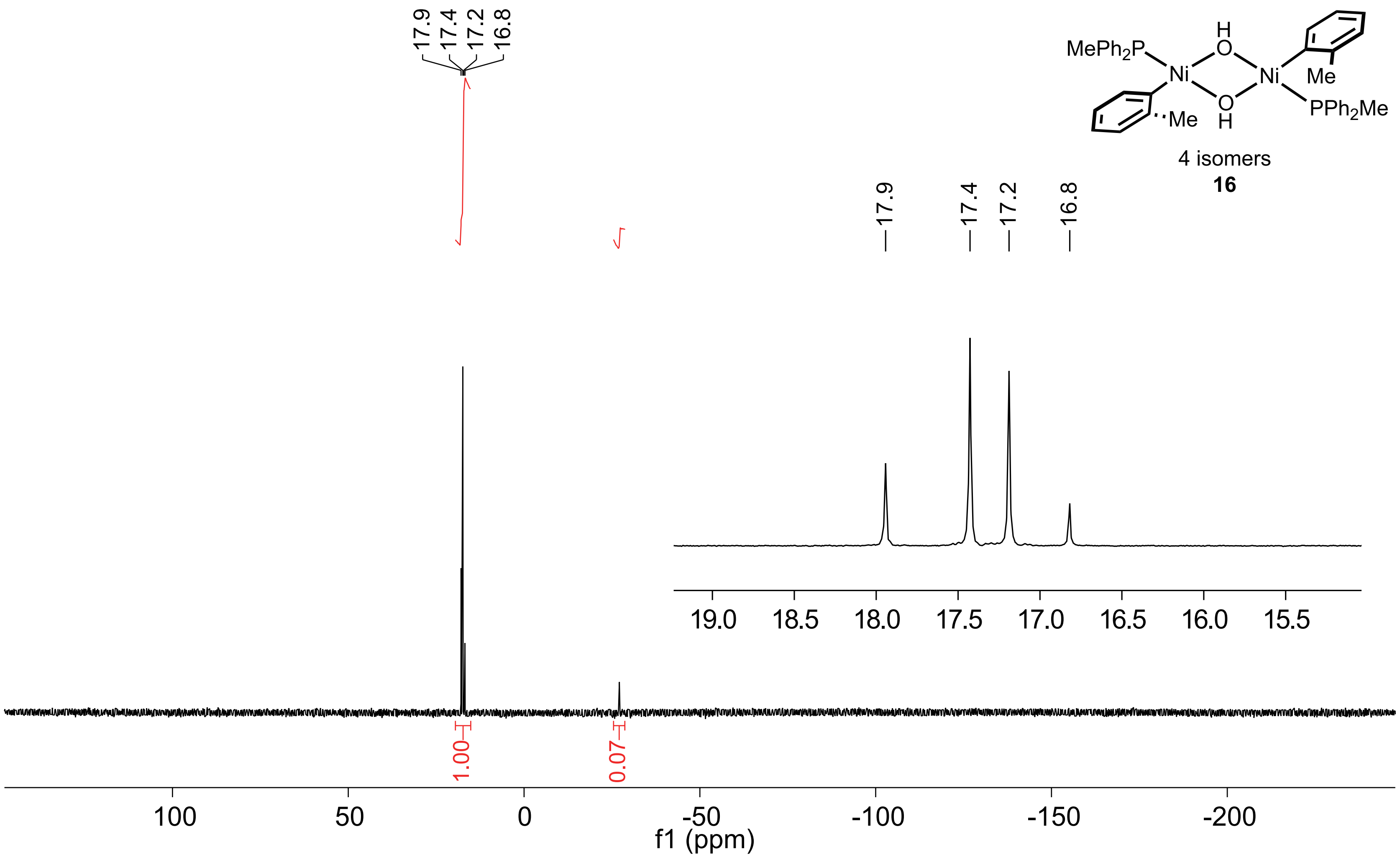

Figure S41 $^{31}P\{^1H\}$ NMR (202.48 MHz, C_6D_6) spectrum of complex [Ni(PPh_2Me)(μ-OH)(*o*-Tol)]$_2$ (**16**). Free PPh_2Me at -24 ppm.

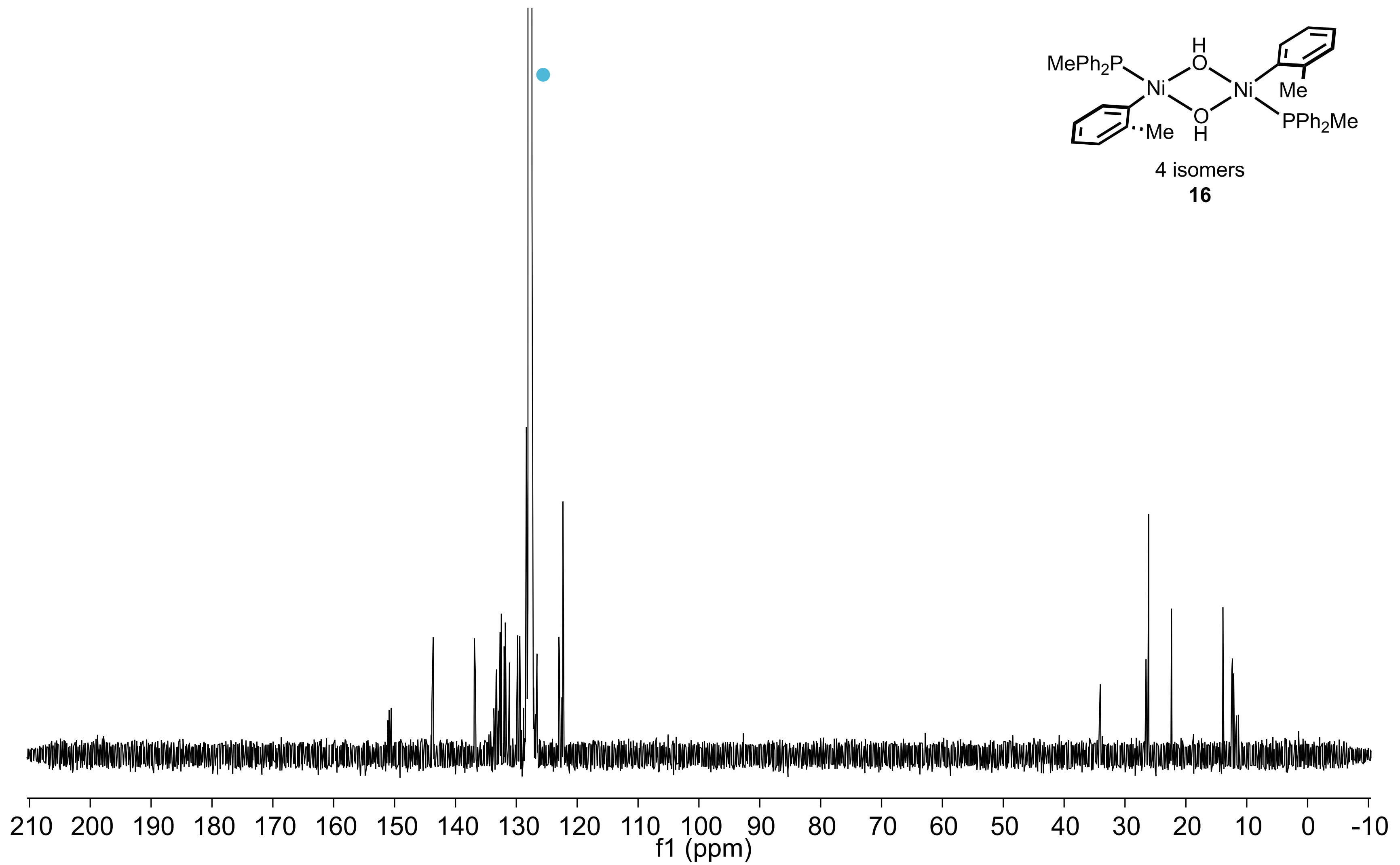

Figure S42 $^{13}C\{^{1}H\}$ NMR (125.79 MHz, C_6D_6) spectrum of complex $[Ni(PPh_2Me)(\mu\text{-}OH)(o\text{-}Tol)]_2$ (**16**). Deuterated solvent (●).

*Diagnostic NMR signals for complex **16**:*

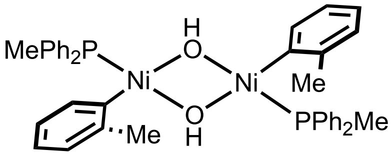

4 isomers
16

^{1}H NMR (500.20 MHz, C_6D_6) δ: 8.06 – 7.19 (m, Ar at PPh_2Me), 7.16 – 6.64 (m, Ar at PPh_2Me & Ar at *o*-Tol), 3.59 (s, CH_3 at *cis*-isomer), 3.35 (s, CH_3 at *cis*-isomer), 3.26 (s, CH_3 at *trans*-isomer), 3.20 (s, CH_3 at *trans*-isomer), 0.81 – 0.69 (m, CH_3 at PPh_2Me), -1.75 (s, OH at *cis*-isomer), -1.87 (s, OH at *cis*-isomer), -3.34 (s, OH at *trans*-isomer), -3.35 (s, *trans*-isomer), -5.31 (s, *cis*-isomer), -5.51 (s, *cis*-isomer).

^{31}P{^{1}H} NMR (202.50 MHz, C_6D_6) δ: 17.9 (s, *cis*-isomer), 17.4 (s, *trans*-isomer), 17.2 (s, *trans*-isomer), 16.8 (s, *cis*-isomer).

^{13}C{^{1}H} NMR spectrum was obtained but not assigned (see Figure S42).

HRMS (ESI-TOF, CH_3CN) m/z:

$[M + O + H]^+$ calculated for $C_{40}H_{43}Ni_2O_3P_2$ 749.1389, found 749.1410; presumably, one of the PPh_2Me ligands at the Ni complex is oxidized during the MS analysis.

$[M - 1 \text{ ligand} + H]^+$ calculated for $C_{27}H_{30}Ni_2O_2P$ 533.0685, found 533.0716.

4.6 Synthesis of Ni(PPh_2Me)$_4$ (18)

A vial was charged with Ni(cod)$_2$ (50.0 mg, 0.181 mmol, 1.0 equiv), PPh_2Me (145 mg, 0.727 mmol, 4.0 equiv) and THF (4 mL). The mixture was stirred at 20 °C overnight, then solvent was removed under vacuum to give an orange solid. The solid was washed with pentane (3 x 5 mL), and further dried under vacuum for 2 h to afford an orange solid (121 mg, 0.141 mmol, 77%). Spectroscopic data are consistent with the literature reports.[8]

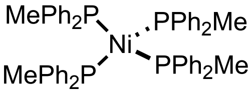

18

^{1}H NMR (500.20 MHz, C_6D_6) δ: 7.28 – 7.20 (m, 16H, H_o), 7.04 (d, *J* = 7.3 Hz, 8H, H_p), 6.97 (t, *J* = 7.4 Hz, 16H, H_m), 1.71 (s, 12H, CH_3).

^{31}P{^{1}H} NMR (202.50 MHz, C_6D_6) δ: 3.9 (s).

4.7 Synthesis of Ni(TMEDA)Cl(*o*-Tol) (S9)

The synthesis was slightly modified from the literature.[11] A vial was charged with Ni(cod)$_2$ (300 mg, 1.09 mmol, 1.0 equiv), *N,N,N',N'*-tetramethylethylenediamine (152 mg, 1.31 mmol, 1.2 equiv) and 2-chlorotoluene (4 mL). The mixture was stirred at 20 °C for 3 days. To the dark orange suspension,

[11] Magano, J.; Monfette, S. Development of an Air-Stable, Broadly Applicable Nickel Source for Nickel-Catalyzed Cross-Coupling. *ACS Catal.* **2015**, *5* (5), 3120–3123.

pentane (5 mL) was added, and the resulting suspension was stirred for 30 min at 20 °C. The solid was vacuum-filtered and washed with pentane (3 x 3 mL). The resulting solid was further dried under vacuum for 2 h to afford an orange solid (300 mg, 0.995 mmol, 91%). NMR data is consistent with the literature.[11] The crude of this compound was directly used for the further synthesis of the corresponding Ni phosphine complex without purification. The purification of **S9** was reported in the literature.[11]

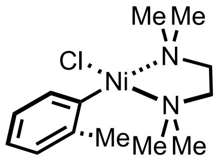

S9

^{1}H NMR (400 MHz, C_6D_6) δ: 7.69 (dd, *J* = 7.4, 1.3 Hz, 1H, H_o at o-Tol), 7.04 – 6.89 (m, 3H, Hs at o-Tol), 3.67 (s, 3H, CH_3 at o-Tol), 2.22 (s, 3H, N-CH_3), 2.10 – 2.01 (m, 4H, N-CH_3 & N-CH_2), 1.82 (s, 3H,N-CH_3), 1.55 – 1.40 (m, 1H, N-CH_2), 1.32 – 1.15 (m, 4H, N-CH_3 & N-CH_2), 1.06 – 0.93 (m, 1H, N-CH_2).

4.8 Synthesis of Ni($PPh_2CH_2CH_2CH_2OH)_2$Cl(*o*-Tol) (21)

A vial was charged with Ni(TMEDA)Cl(*o*-Tol) (**S9**, 100 mg, 0.332 mmol, 1.0 equiv), $PPh_2CH_2CH_2CH_2OH$ (172 mg, 0.730 mmol, 2.2 equiv) and THF (4 mL). The mixture was stirred at 20 °C for 2 days, then solvent was removed under vacuum to give an orange oil. To the orange oil, toluene (2 mL) was added, and the suspension was filtrated through a celite. The resulting orange solution was concentrated under vacuum, layered with pentane (5 mL), and stored at -35 °C overnight to give a yellow solid. The solid was further dried under vacuum for 2 h to afford an orange solid (197 mg, 0.292 mmol, 88%).

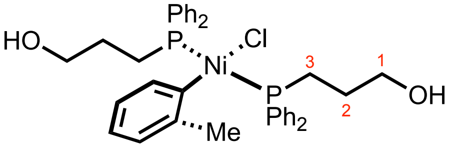

21

^{1}H NMR (400.30 MHz, C_6D_6) δ: 7.83 (tdd, *J* = 7.1, 5.4, 3.6 Hz, 4H, H_o at Ph), 7.45 (dtd, *J* = 8.5, 4.5, 2.4 Hz, 4H, H_o at Ph), 7.10 – 6.97 (m, 13H, H_m, H_p at Ph & *o*-Tol), 6.63 – 6.48 (m, 2H, *o*-Tol), 6.39 (dd, *J* = 7.3, 1.7 Hz, 1H, *o*-Tol), 3.16 (q, *J* = 5.9 Hz, 4H, H1), 2.75 (s, 3H, CH_3 at *o*-Tol), 2.10 – 1.90 (m, 4H, H3), 1.56 – 1.36 (m, 4H, H2), 0.79 (t, *J* = 5.6 Hz, 2H, OH).

^{31}P{^{1}H} NMR (162.04 MHz, C_6D_6) δ: 13.4 (s).

^{13}C{^{1}H} NMR (100.67 MHz, C_6D_6) δ: 150.0 (t, *J* = 33 Hz, *o*-Tol), 143.8 (t, *J* = 3 Hz, *o*-Tol), 137.0 (t, *J* = 5 Hz, *o*-Tol), 134.6 (t, *J* = 6 Hz, C_o at Ph), 133.5 (t, *J* = 5 Hz, C_o at Ph), 132.7 (t, *J* = 20 Hz, C_{ipso} at Ph), 132.4 (t, *J* = 20 Hz, C_{ipso} at Ph), 129.9 (s, C_p at Ph), 129.3 (s, C_p at Ph), 124.0 (t, *J* = 3 Hz, *o*-Tol), 122.6 (t, *J* = 3 Hz, *o*-Tol), 63.0 (t, *J* = 7 Hz, C1), 28.3 (s, C3), 26.5 (s, CH_3 at *o*-Tol), 23.0 (t, *J* = 14 Hz, C2). Signals due to C_m at Ph are not observed because of their overlapping with the signal due to C_6D_6.

HRMS (ESI-TOF, CH_3CN) m/z: [M - Cl]$^+$ calculated for $C_{37}H_{41}NiO_2P_2$ 637.1935, found 637.1929.

4.9 NMR spectra of isolatable Ni complexes

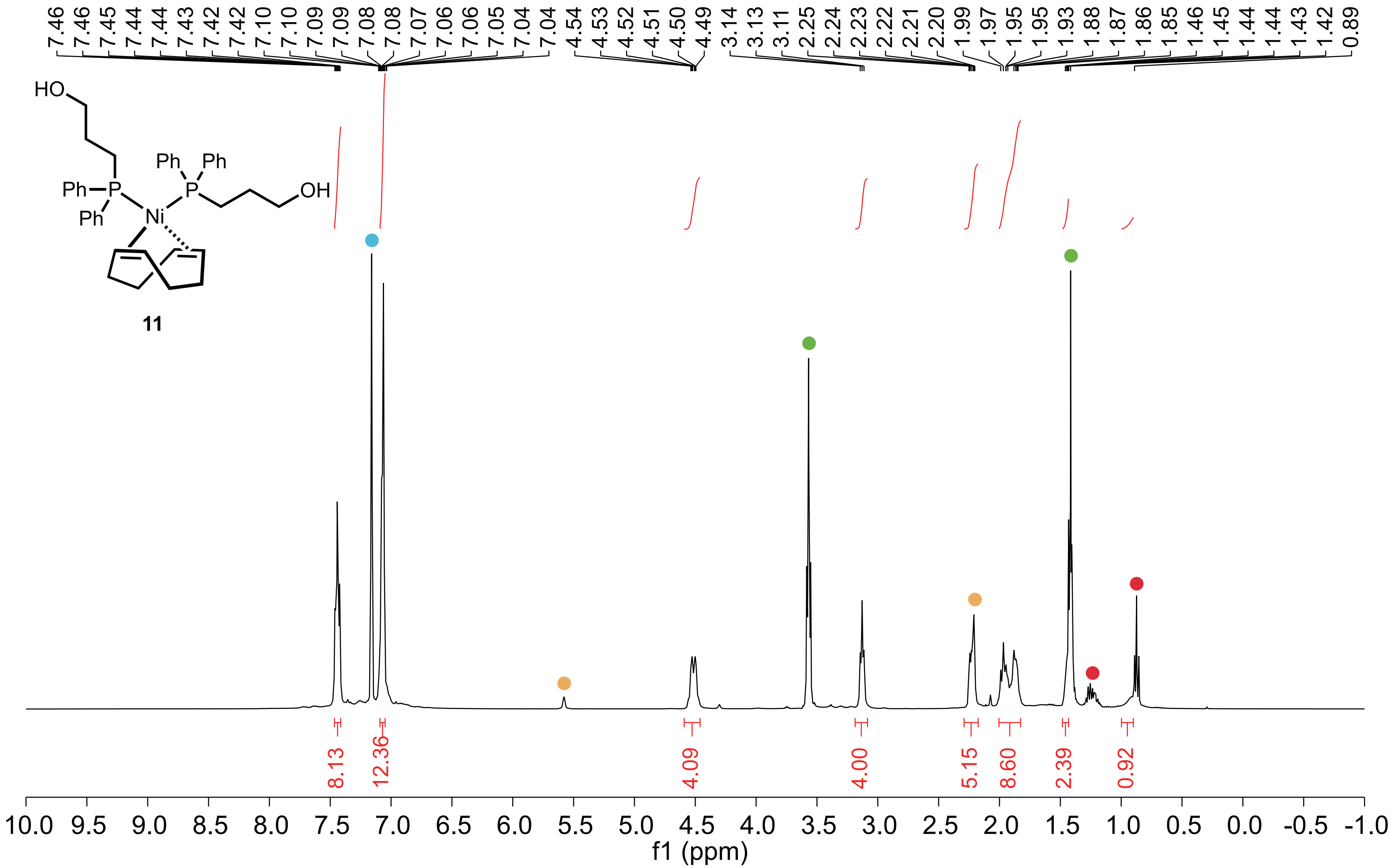

Figure S43 ^{1}H NMR (400.30 MHz, C_6D_6) spectrum of complex Ni($PPh_2CH_2CH_2CH_2OH)_2$(cod) (**11**). Residual proteo-solvent (•), free cod (•), co-crystallized THF (•) and pentane (•).

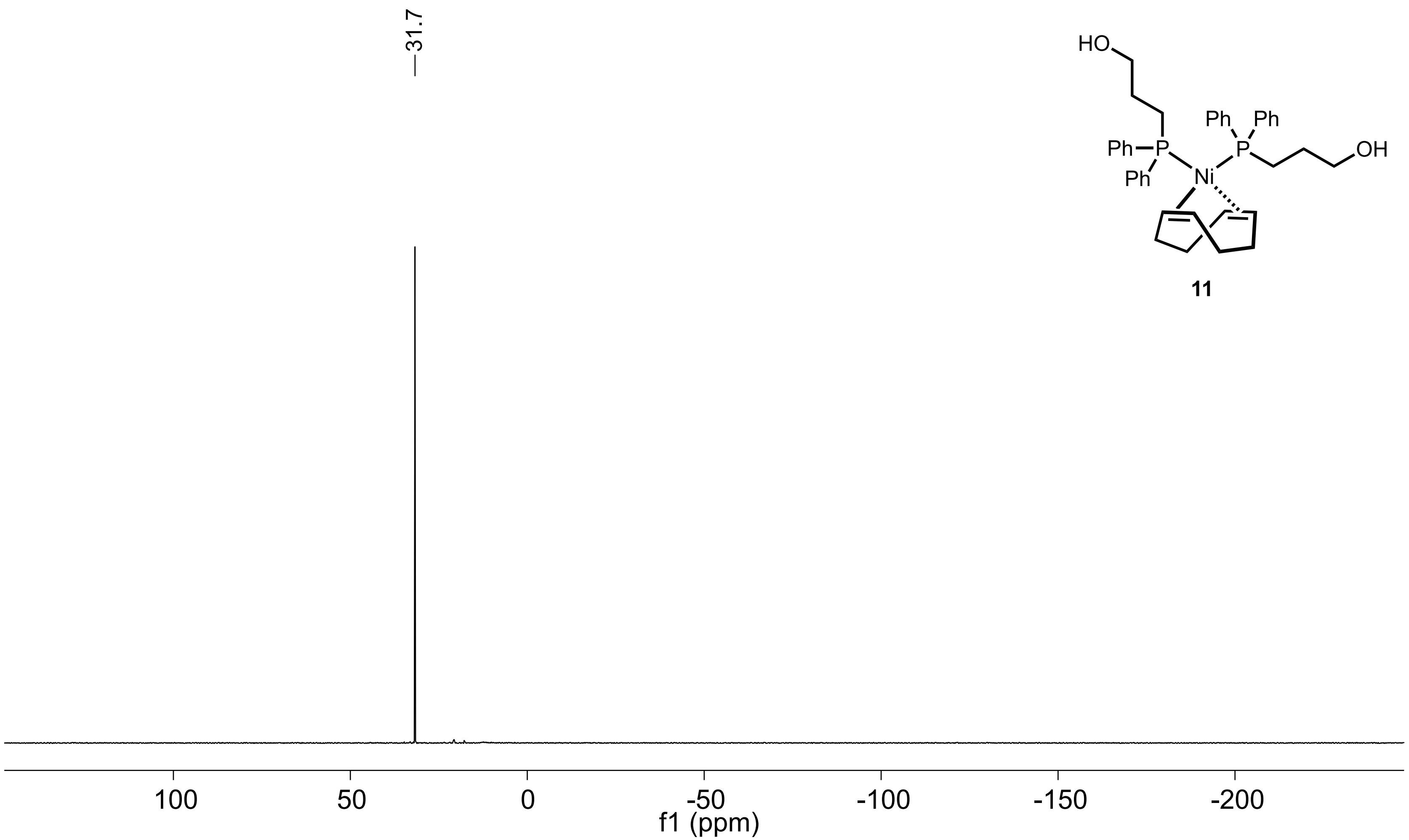

Figure S44 $^{31}P\{^{1}H\}$ NMR (162.04 MHz, C_6D_6) spectrum of complex $Ni(PPh_2CH_2CH_2CH_2OH)_2(cod)$ (**11**).

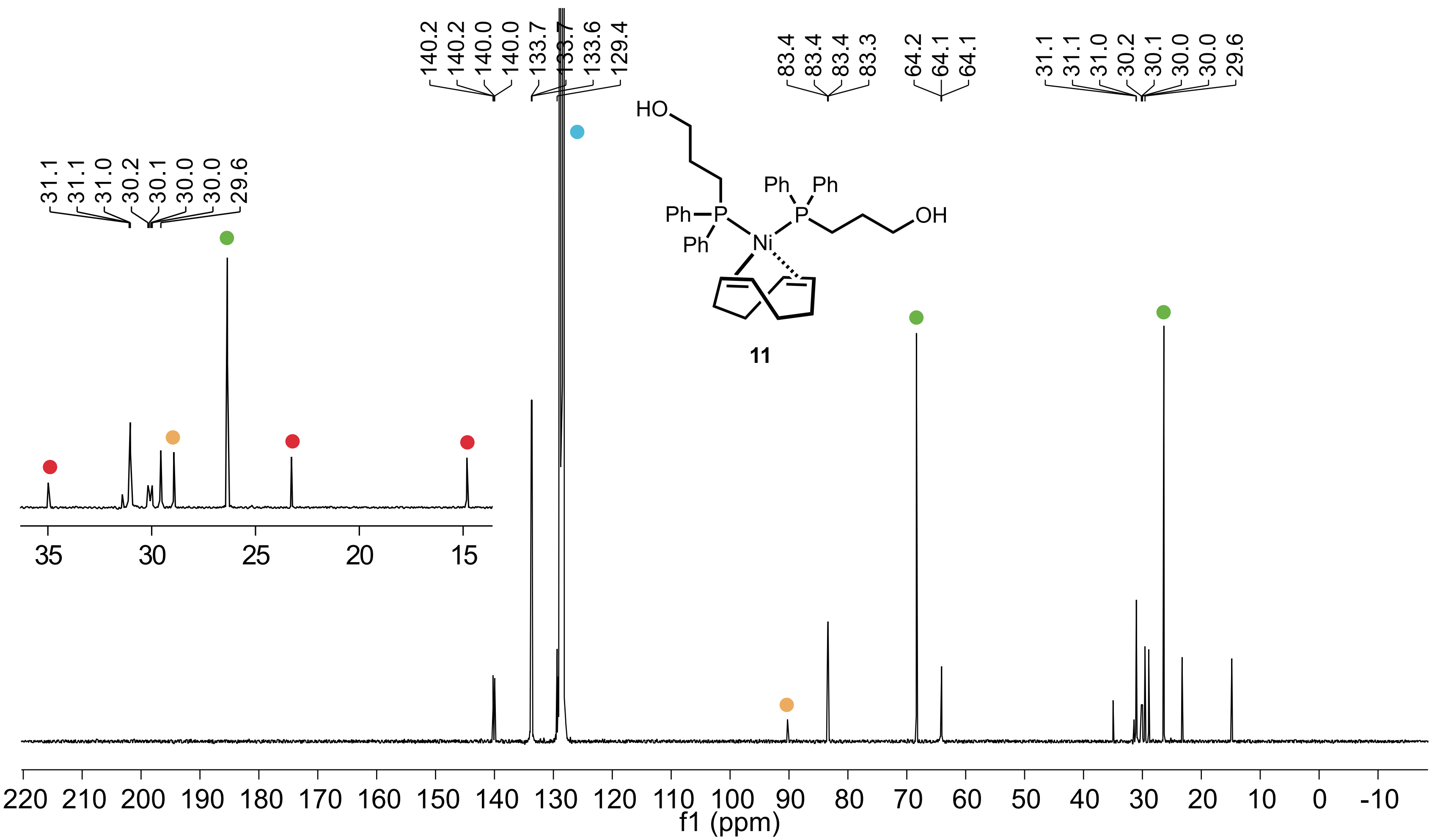

Figure S45 $^{13}C\{^{1}H\}$ NMR (100.67 MHz, C_6D_6) spectrum of complex $Ni(PPh_2CH_2CH_2CH_2OH)_2(cod)$ (**11**). Deuterated solvent (•), free cod (•), co-crystallized THF (•) and pentane (•).

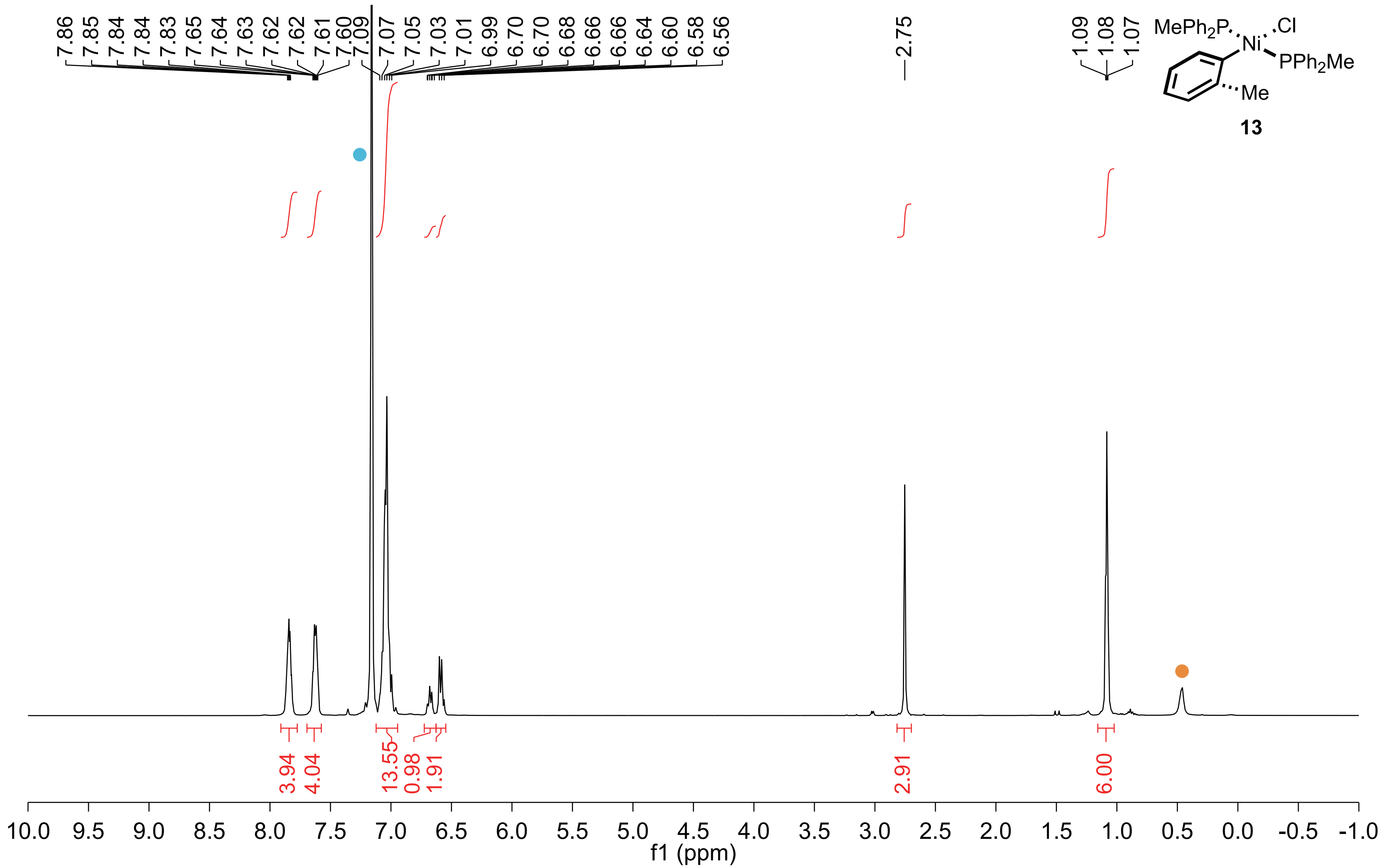

Figure S46 ^{1}H NMR (400.30 MHz, C_6D_6) spectrum of complex $Ni(PPh_2Me)_2Cl$(*o*-Tol) (**13**). Residual proteo-solvent (●) and H_2O (●).

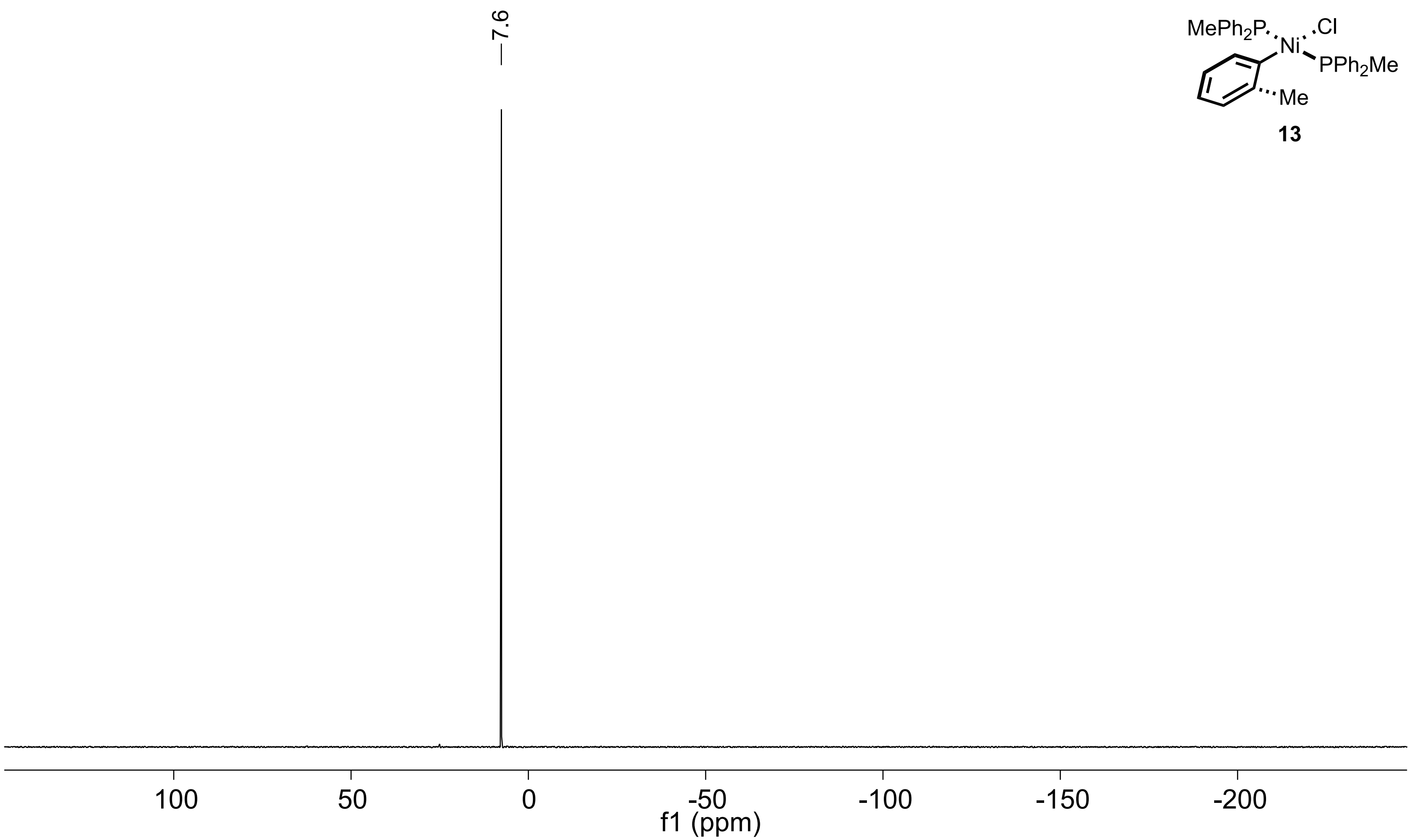

13

Figure S47 $^{31}P\{^1H\}$ NMR (162.04 MHz, C_6D_6) spectrum of complex $Ni(PPh_2Me)_2Cl$(*o*-Tol) (**13**).

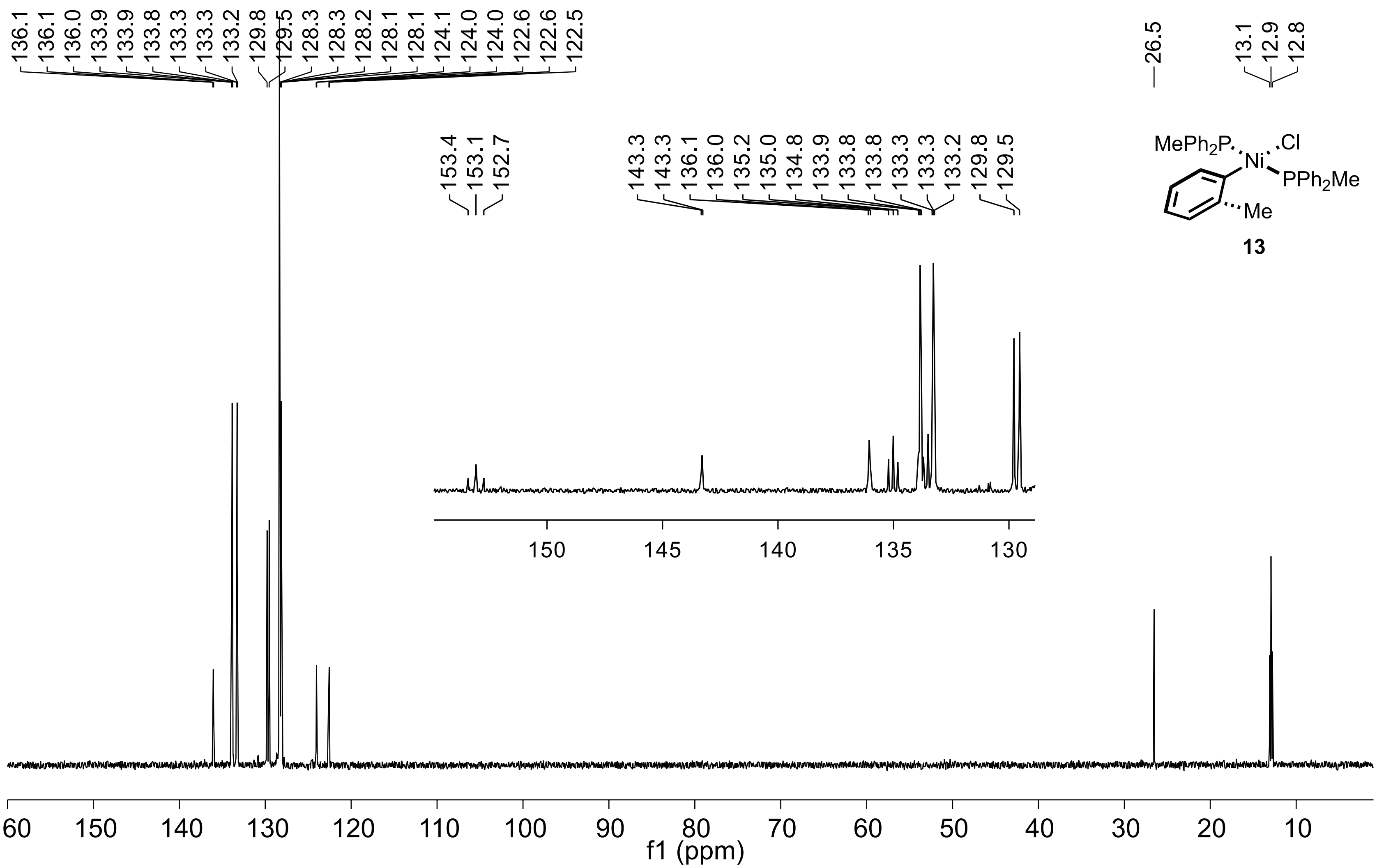

Figure S48 ^{13}C DEPT 135 NMR (100.67 MHz, C_6D_6) spectrum of complex $Ni(PPh_2Me)_2Cl$(*o*-Tol) (**13**). Inset shows $^{13}C\{^1H\}$ NMR of all quaternary carbons.

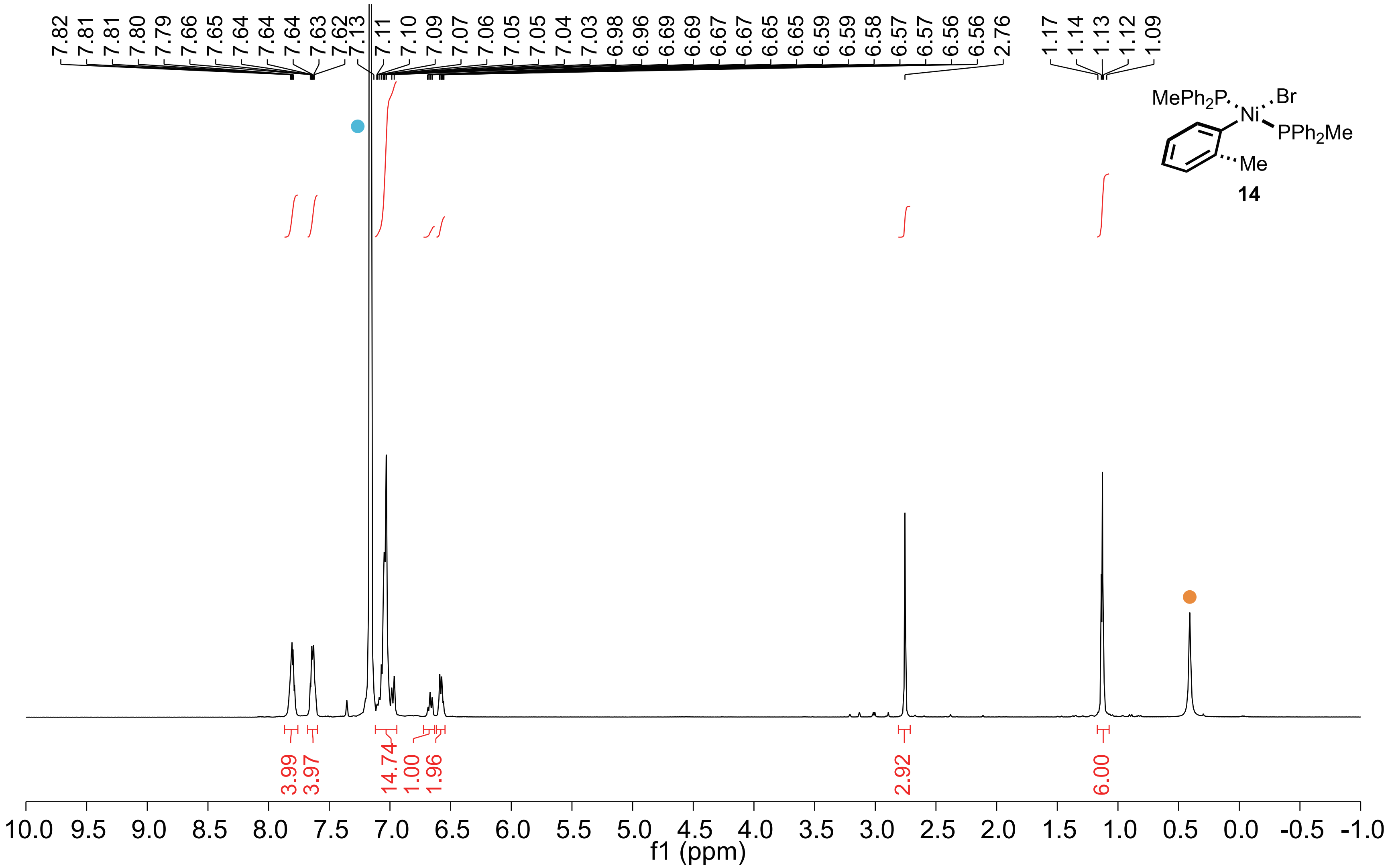

Figure S49 ^{1}H NMR (400.30 MHz, C_6D_6) spectrum of complex $Ni(PPh_2Me)_2Br$(*o*-Tol) (**14**). Residual proteo-solvent (•) and H_2O(•).

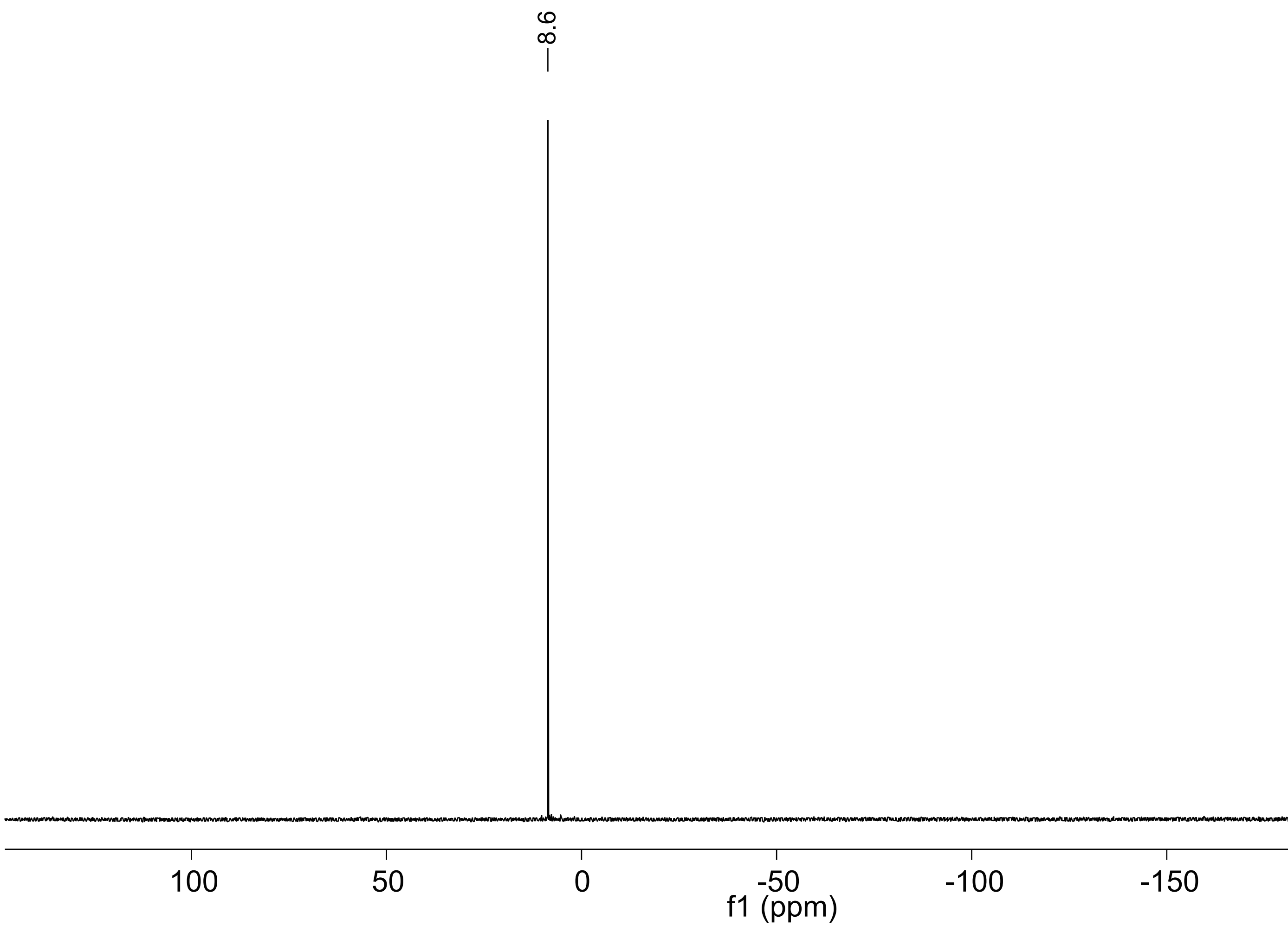

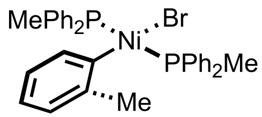

14

Figure S50 $^{31}P\{^1H\}$ NMR (162.04 MHz, C_6D_6) spectrum of complex $Ni(PPh_2Me)_2Br$(*o*-Tol) (**14**).

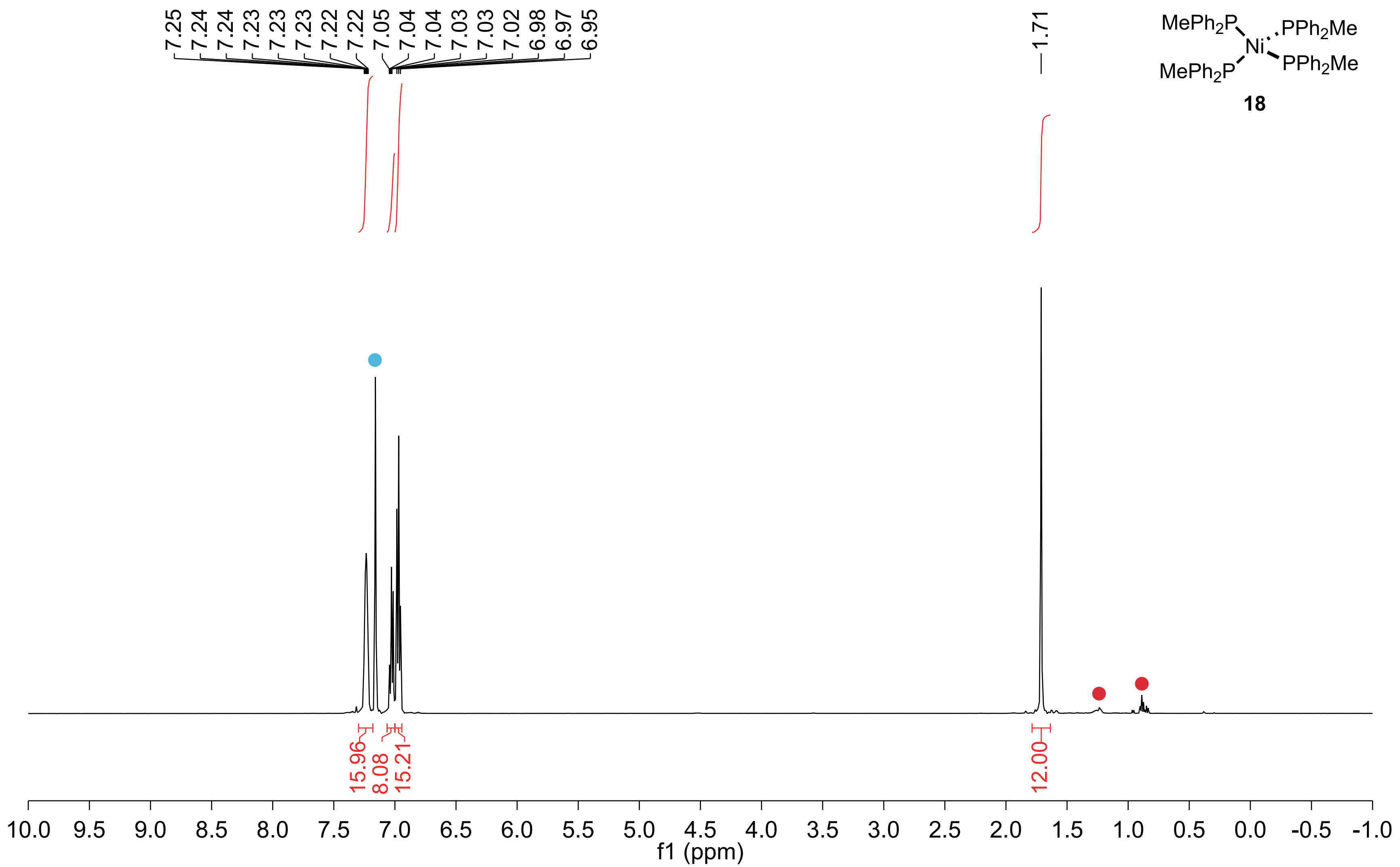

Figure S51 ^{1}H NMR (400.30 MHz, C_6D_6) spectrum of complex $Ni(PPh_2Me)_4$ (**18**). Residual proteo-solvent (•) and pentane (•).

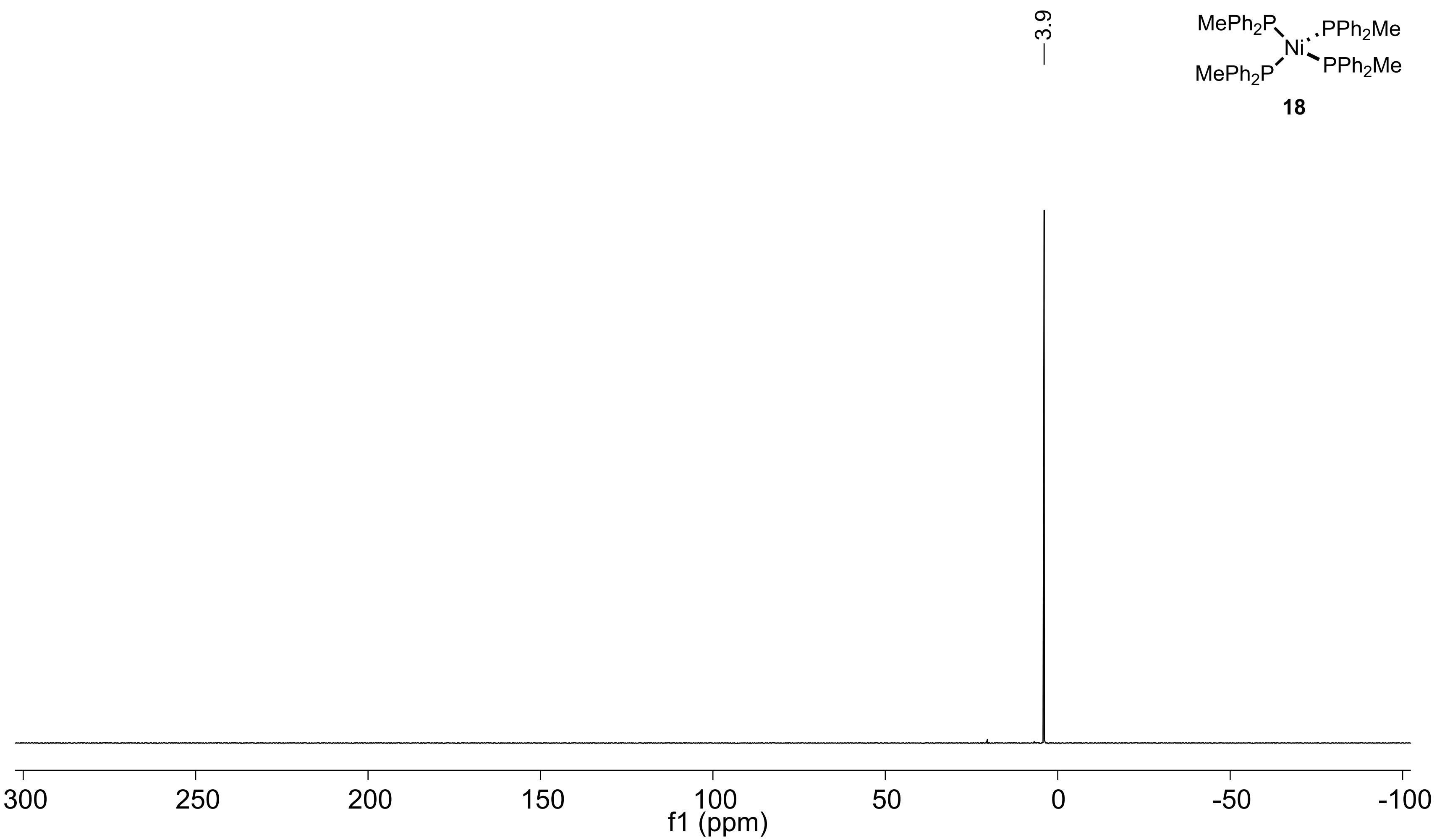

18

Figure S52 $^{31}P\{^{1}H\}$ NMR (162.04 MHz, C_6D_6) spectrum of complex $Ni(PPh_2Me)_4$ (**18**).

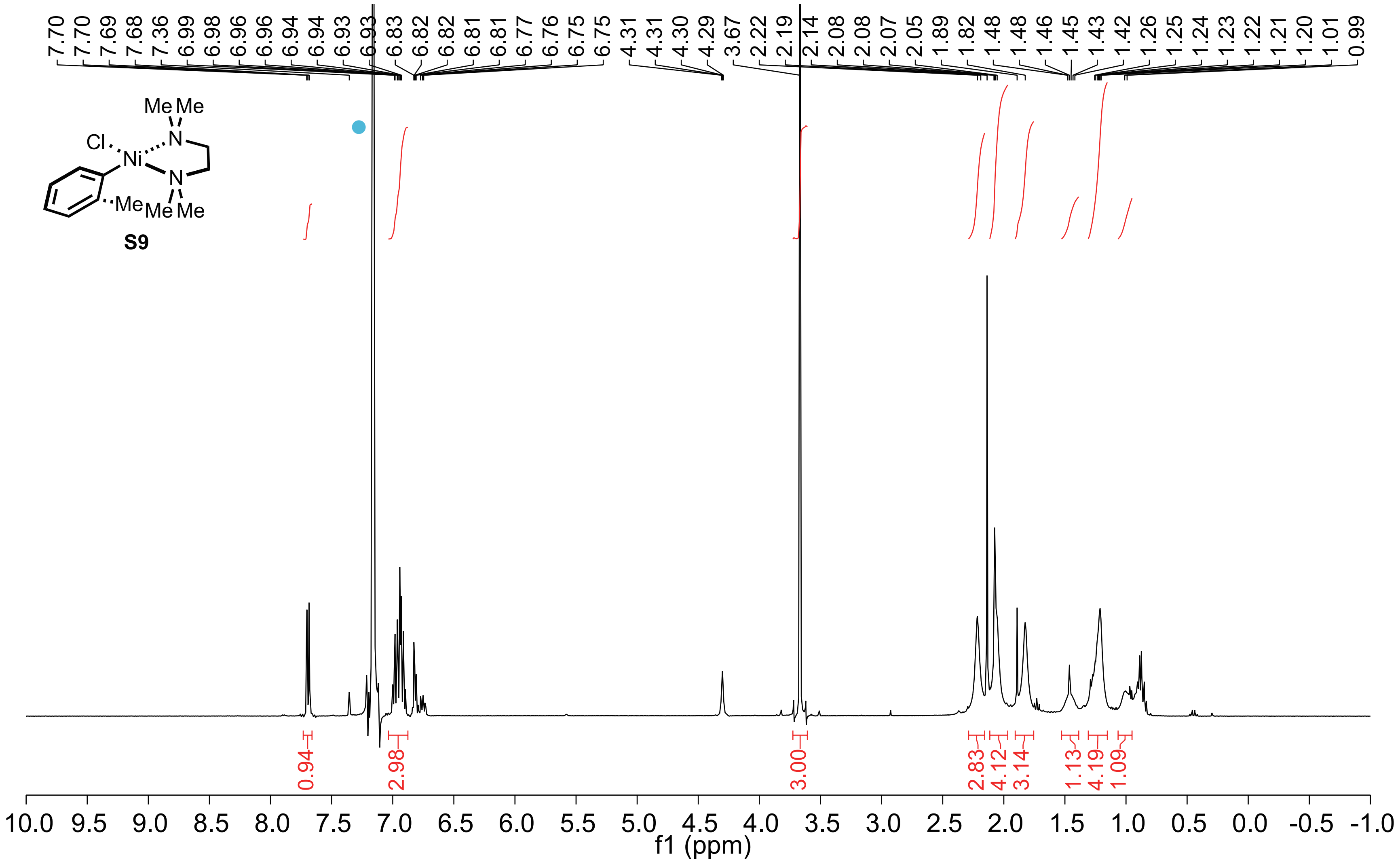

Figure S53 ^{1}H NMR (400.30 MHz, C_6D_6) spectrum of crude Ni(TMEDA)Cl(*o*-Tol) (**S9**). Residual proteo-solvent (•).

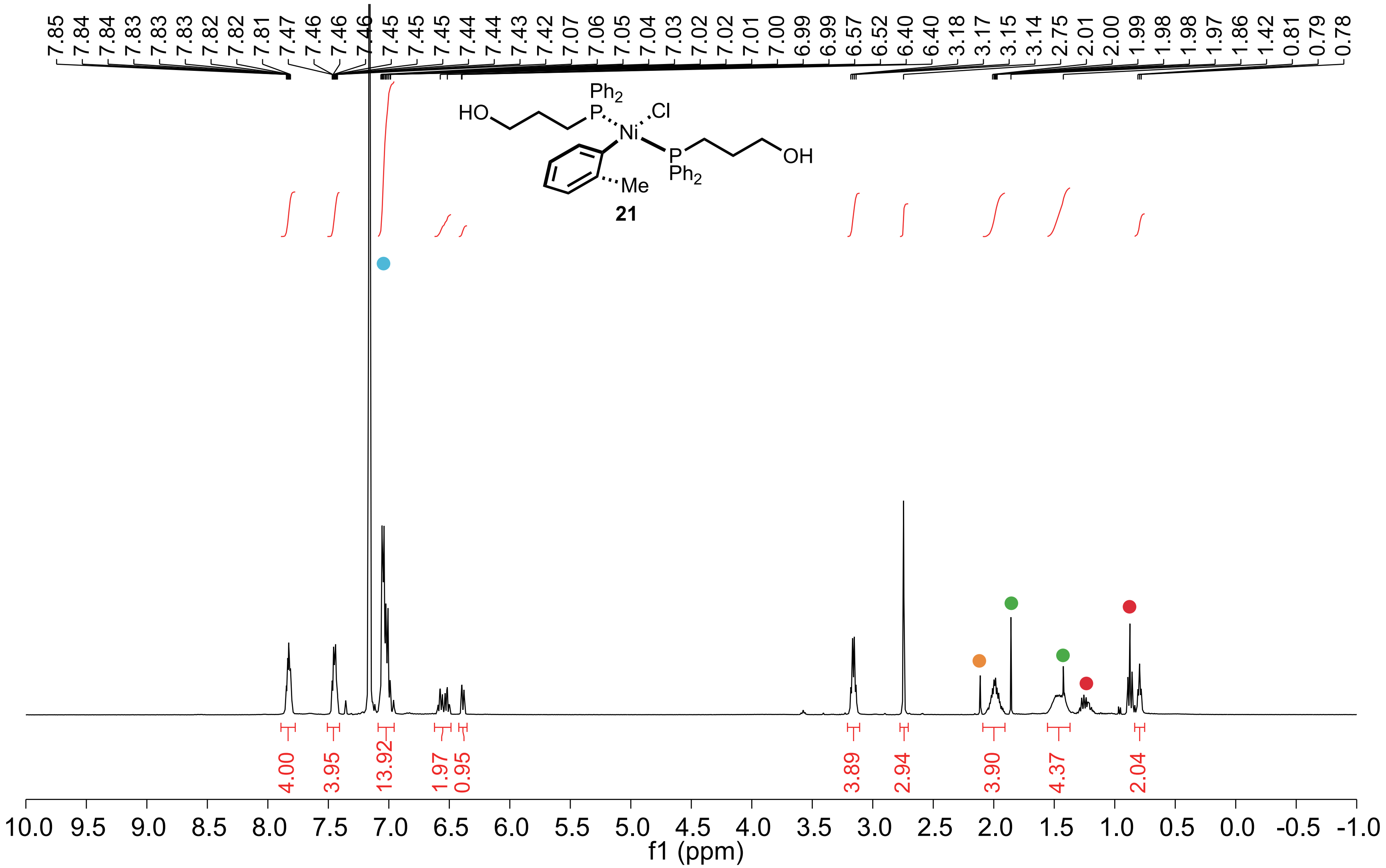

Figure S54 ^{1}H NMR (400.30 MHz, C_6D_6) spectrum of complex $Ni(PPh_2CH_2CH_2CH_2OH)_2Cl(o\text{-}Tol)$ (**21**). Residual proteo-solvent (●), toluene (●), unidentified non-Ni species (●) and pentane (●).

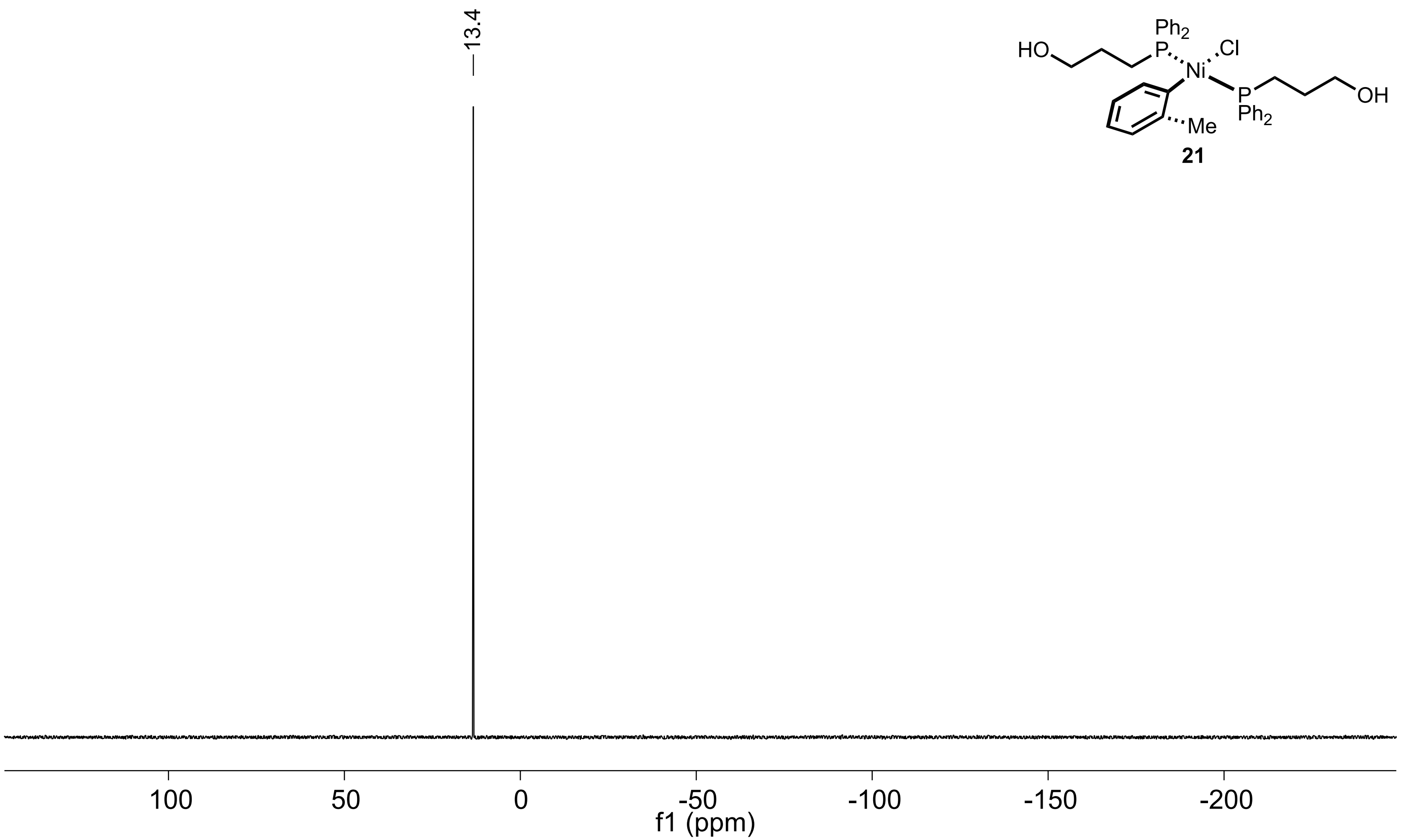

21

Figure S55 $^{31}P\{^{1}H\}$ NMR (162.04 MHz, C_6D_6) spectrum of complex $Ni(PPh_2CH_2CH_2CH_2OH)_2Cl$(*o*-Tol) (**21**).

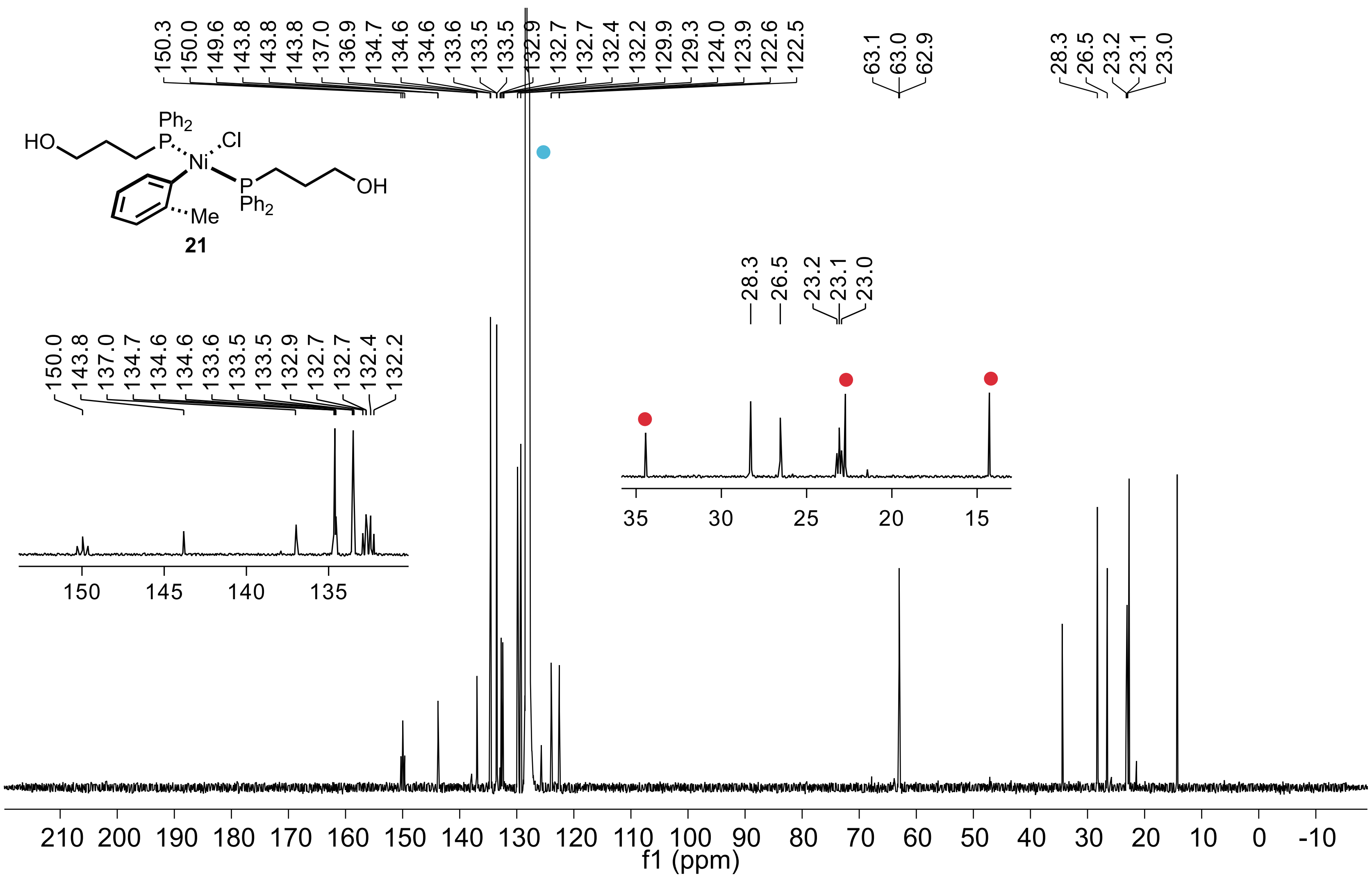

Figure S56 $^{13}C\{^{1}H\}$ NMR (100.67 MHz, C_6D_6) spectrum of complex $Ni(PPh_2CH_2CH_2CH_2OH)_2Cl$(*o*-Tol) (**21**). Deuterated solvent (●) and pentane (●).

5. Organometallic study of Ni phosphine complexes

5.1 Resting state analysis of the catalytic systems using PPh_2Me or ProPhos as a ligand

General procedure for detecting the catalyst resting state

Ni(cod)$_2$ (3.0 mg, 0.011 mmol, 1.0 equiv) and a phosphine ligand (ProPhos **9**: 11 mg, 0.044 mmol, 4.0 equiv; PPh_2Me: 8.1 mL, 0.044 mmol, 4.0 equiv) were dissolved in 2-MeTHF (0.4 mL) for 30 min. An electrophile (2-chlorotoluene: 13 mL, 0.109 mmol, 10 equiv; 2-bromotoluene: 13 mL, 0.109 mmol, 10 equiv) and B(*p*-Tol)Pin **5** (26 mg, 0.120 mmol, 11 equiv)/BPh(OH)$_3$ **6** (15 mg, 0.120 mmol, 11 equiv) were separately added to the Ni solution. The solution was transferred to a J. Young tube. After that, the K_3PO_4 (23 mg, 0.109 mmol, 10 equiv) and C_6D_6 (0.1 mL, for locking purpose) were added to the NMR tube and removed from the glove box, then deionized water (2 μL) was added to the tube via a syringe. The sample was initially monitored by $^{31}P\{^1H\}$ NMR prior to the heating (obtained at 15 min), and then placed in an oil bath and heated to 70 °C. The reaction progress was monitored by $^{31}P\{^1H\}$ NMR (run at rt).

Note: 2-chlorotoluene or 2-bromotoluene was selected since we were able to isolate the corresponding Ni aryl halide complexes (e.g. Ni(PPh_2Me)$_2$Br(*o*-Tol) **14**). Using established ^{31}P chemical shift values for these complexes helped assign reaction intermediates. Less K_3PO_4 and water were added to the sample in order to decelerate the reaction to facilitate kinetic measurements and improve shimming. Parallel experiments were performed in the NMR tubes to ensure the catalytic conversion. The reaction mixture after 1 h heating was extracted with ethyl acetate and washed with water. An aliquot of organic layer was analyzed by GCMS, revealing the formation of the product. The reaction with **6** resulted in a heterogeneous solution with poor shimming. Thus, the reaction mixtures at the specific time point were filtrated in the glovebox, and the filtrates were analyzed by $^{31}P\{^1H\}$ NMR.

5.1.1 Reaction of 2-bromotoluene and B(*p*-Tol)Pin (5) using Ni(cod)$_2$ and PPh$_2$Me

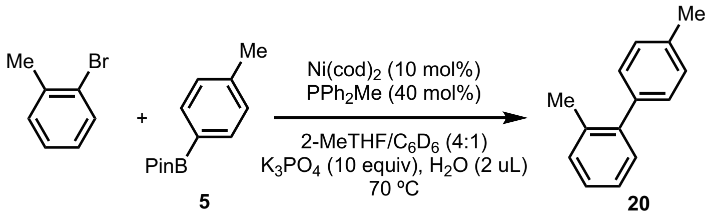

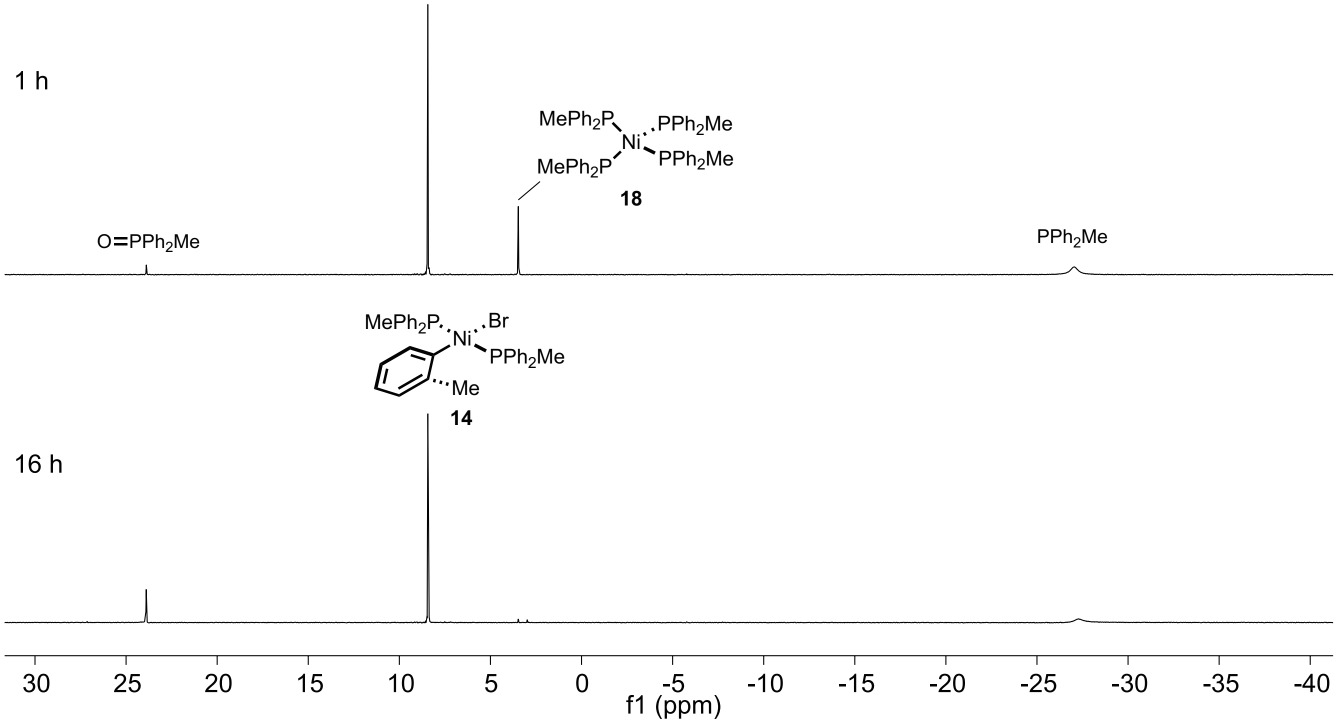

Figure S57 $^{31}P\{^{1}H\}$ NMR (162.04 MHz, C_6D_6:2-MeTHF = 1:4) spectrum of the catalytic cross-coupling of 2-bromotoluene and B(*p*-Tol)Pin (**5**) using 10 mol % Ni(cod)$_2$ and 40 mol% PPh$_2$Me at 70 °C, showing the observed Ni species overtime.

5.1.2 Reaction of 2-bromotoluene and $BPh(OH)_2$ (6) using $Ni(cod)_2$ and PPh_2Me

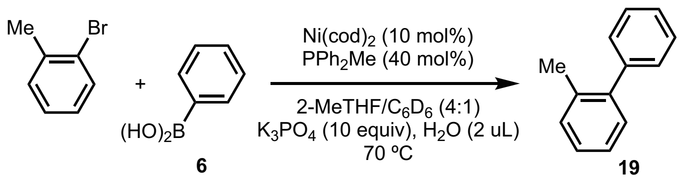

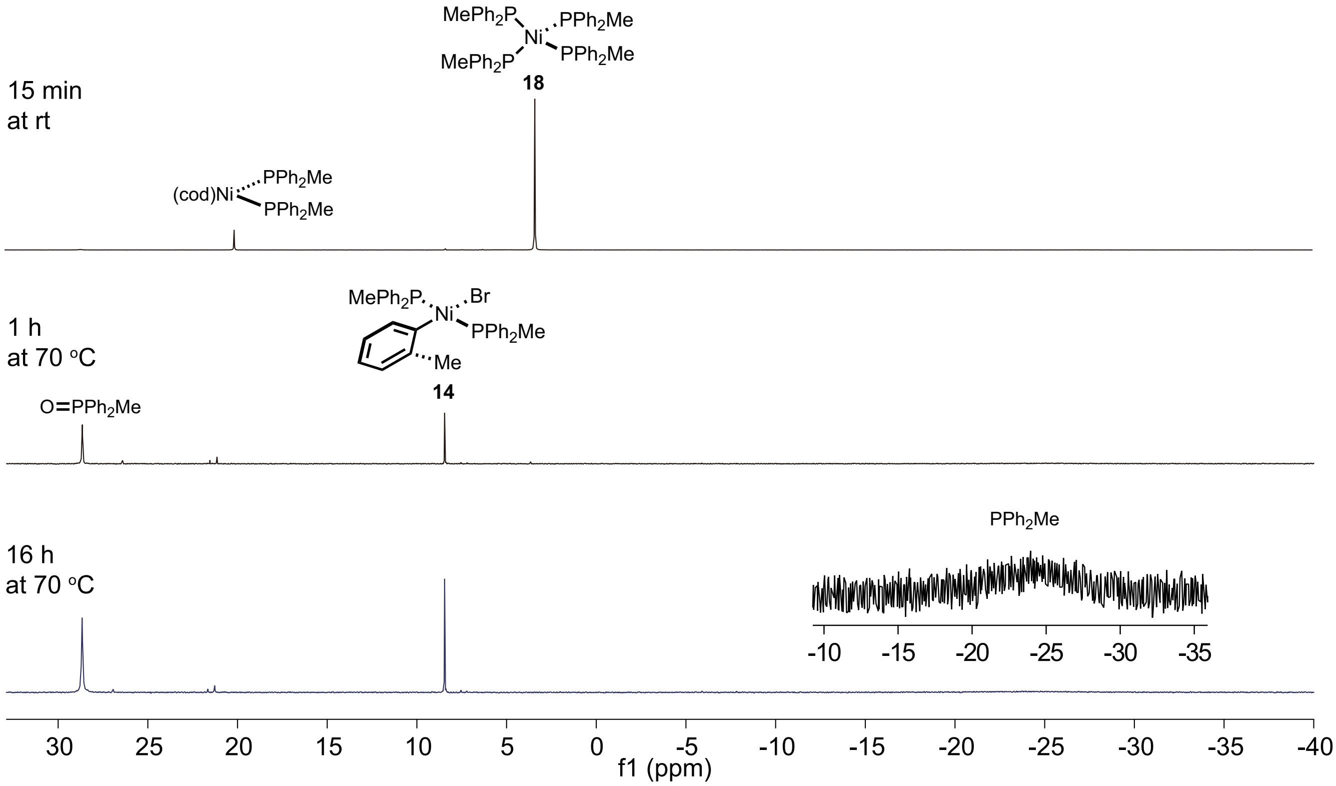

Figure S58 $^{31}P\{^1H\}$ NMR (162.04 MHz, C_6D_6:2-MeTHF = 1:4) spectrum of the catalytic cross-coupling of 2-bromotoluene and $BPh(OH)_2$ (**6**) using 10 mol % $Ni(cod)_2$ and 40 mol% PPh_2Me at 70 °C, showing the observed Ni species overtime.

5.1.3 Reaction of 2-chlorotoluene and B(*p*-Tol)Pin (5) using Ni(cod)$_2$ and ProPhos

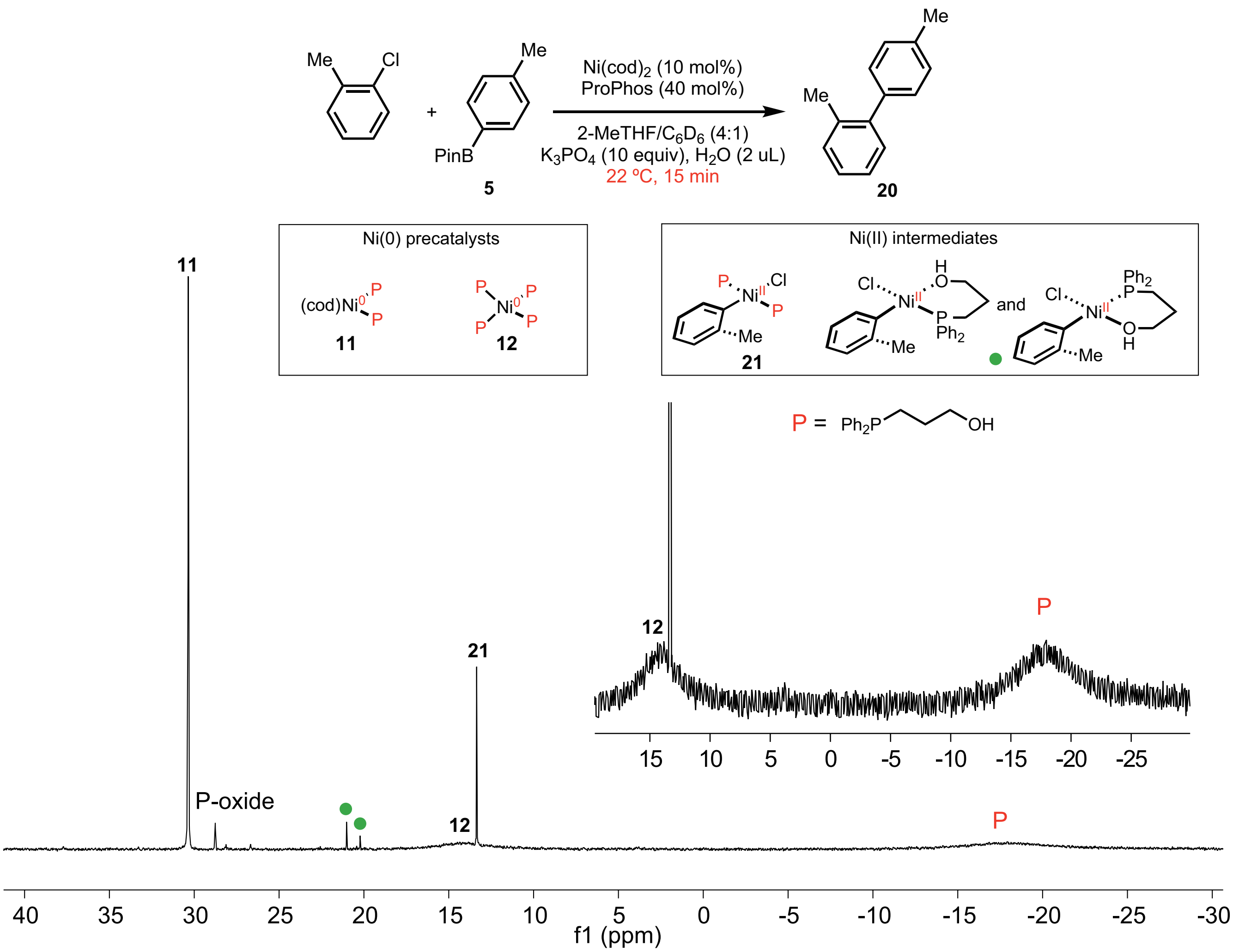

Figure S59 $^{31}P\{^1H\}$ NMR (162.04 MHz, C_6D_6:2-MeTHF = 1:4) spectrum of the catalytic cross-coupling of 2-chlorotoluene and B(*p*-Tol)Pin (**5**) using 10 mol % Ni(cod)$_2$ and 40 mol% ProPhos at rt for 15 min.

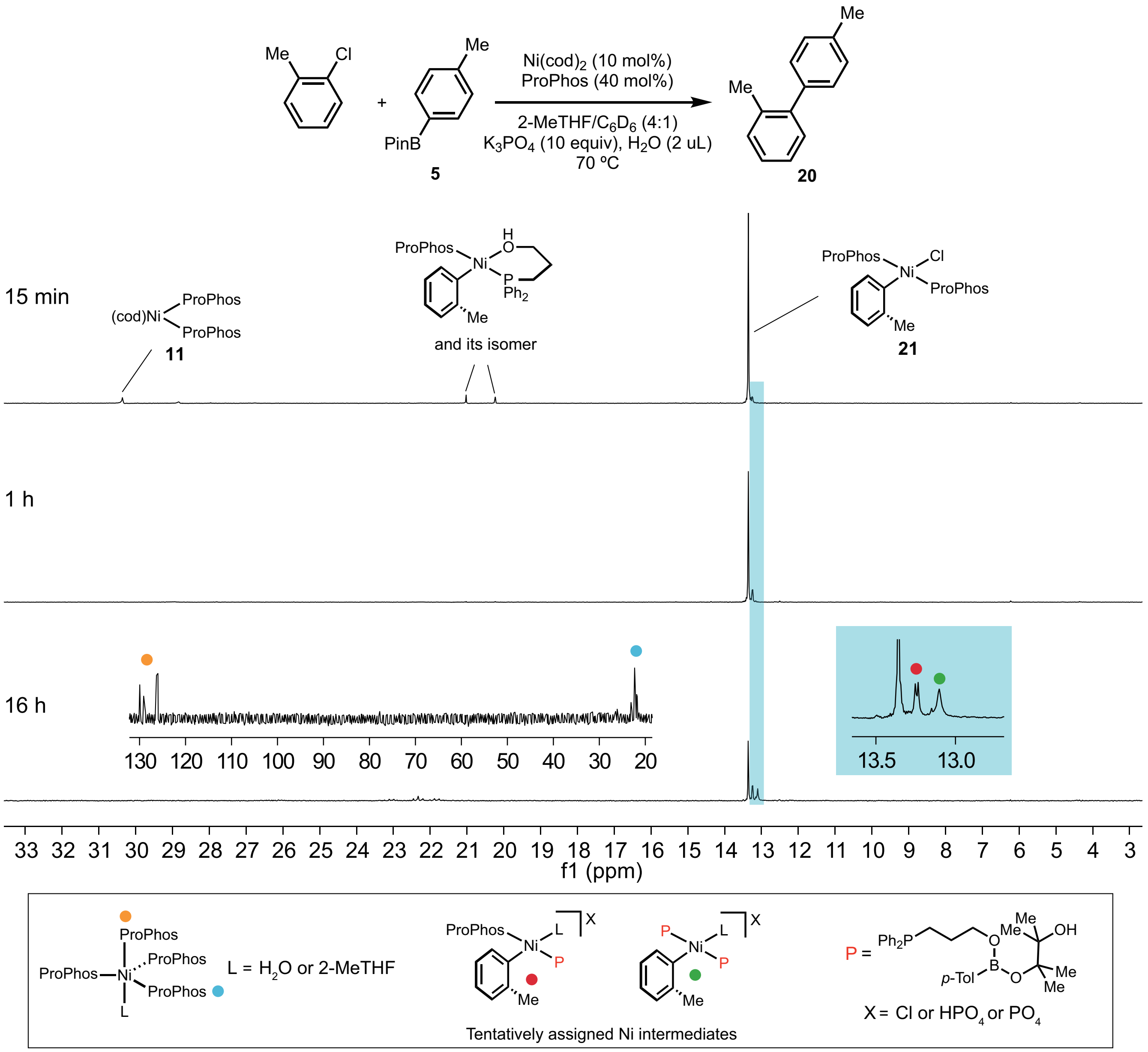

Figure S60 $^{31}P\{^{1}H\}$ NMR (162.04 MHz, C_6D_6:2-MeTHF = 1:4) spectrum of the catalytic cross-coupling of 2-chlorotoluene and B(*p*-Tol)Pin (**5**) using 10 mol % $Ni(cod)_2$ and 40 mol% ProPhos at 70 °C, showing the observed Ni species overtime. The broad signal due to the Ni boron adduct (e.g. green dot) could be attributed to the reversible chelation of the phosphine ligands to the cationic Ni center.

5.1.4 Reaction of 2-bromotoluene and B(*p*-Tol)Pin (5) using Ni(cod)$_2$ and ProPhos

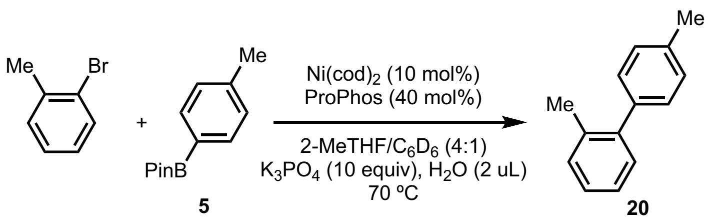

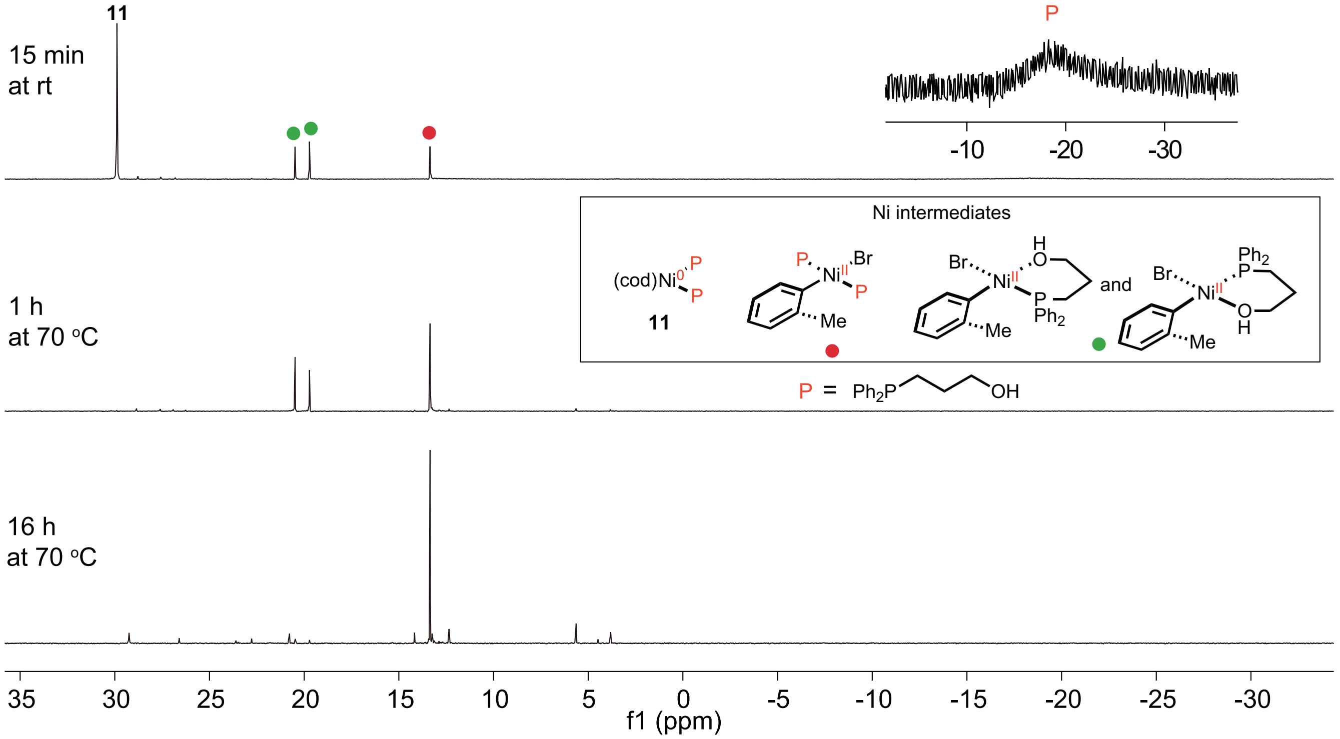

Figure S61 $^{31}P\{^1H\}$ NMR (162.04 MHz, C_6D_6:2-MeTHF = 1:4) spectrum of the catalytic cross-coupling of 2-bromotoluene and B(*p*-Tol)Pin (**5**) using 10 mol % Ni(cod)$_2$ and 40 mol% ProPhos, showing the observed Ni species overtime at rt and heating at 70 °C. Note: the assigned complex Ni($PPh_2CH_2CH_2CH_2OH$)$_2$Br(*o*-Tol) shows an identical ^{31}P shift to its analogue **21**.

5.1.5 Reaction of 2-bromotoluene and $BPh(OH)_2$ (6) using $Ni(cod)_2$ and ProPhos

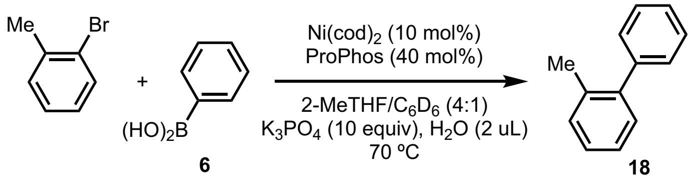

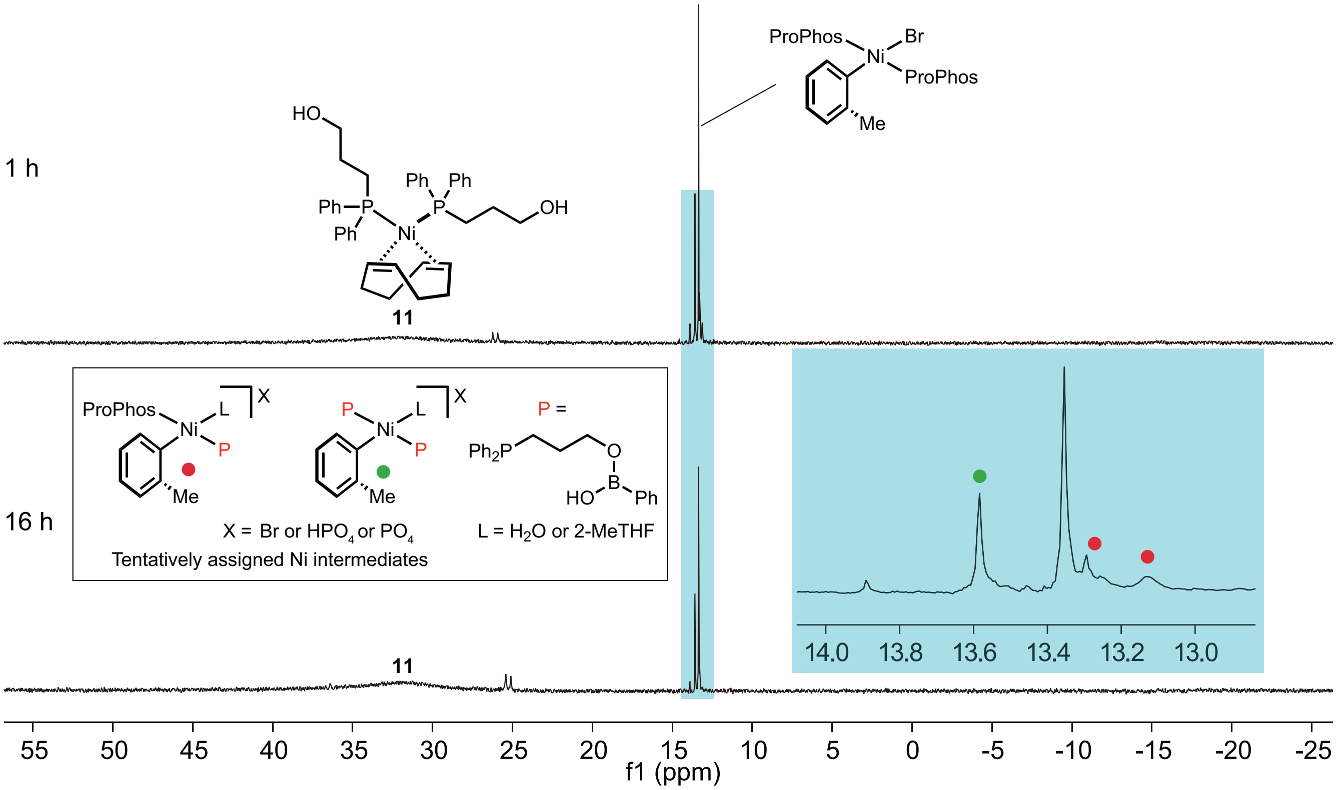

Figure S62 $^{31}P\{^1H\}$ NMR (162.04 MHz, C_6D_6:2-MeTHF = 1:4) spectrum of the catalytic cross-coupling of 2-bromotoluene and $BPh(OH)_2$ (**6**) using 10 mol % $Ni(cod)_2$ and 40 mol% ProPhos at 70 °C, showing the observed Ni species overtime. A noticeable broadening is observed in most signals due to Ni species (e.g. **11**), which is likely caused by exchanges occurring between phosphine ligands and the solvent.

5.2 Study of B,O-coordination between complex $Ni(PPh_2CH_2CH_2CH_2OH)_2Cl(o\text{-}Tol)$ (21) with boronic acids and esters

5.2.1 Reaction of complex 21 with 4-OMe-$PhB(OH)_2$ (22)

We added 4-methoxyphenylboronic acid (4-OMe-$PhB(OH)_2$ **22**, 3.4 mg, 22 μmol, 5.0 equiv) to a C_6D_6 solution of the isolated complex $Ni(PPh_2CH_2CH_2CH_2OH)_2Cl(o\text{-}Tol)$ (**21**, 3.0 mg, 4.5 μmol, 1.0 equiv) at rt. Over a 15-minute period, there was no observable change in color. The $^{31}P\{^1H\}$ NMR spectrum shows three new major signals at 14.9, 14.2 and 13.8 ppm, along with other trace signals (Figure S63). We performed $^1H/^{31}P\{^1H\}$-HMBC NMR experiments on these ^{31}P signals (Figure S64), revealing that both ^{31}P signals at 13.8 and 14.2 ppm (green dots) exhibits a correlation with the ^1H signals ranging from 2.08 to 1.91 ppm (green). Despite the overlap of all three resonances (**21** and **23**) corresponding to the methylene group (green) attached to P at approximately 2.0 ppm, the suggested coordination involving B and O results in a minor shift in the other methylene protons (blue and orange)

tentatively assigned as **23**, as depicted in the ^{1}H-COSY spectrum of this blend (Figure S65). The ^{1}H signals falling within 2.08 and 1.91 ppm (green) show a ^{1}H-COSY correlation with the ^{1}H signals from 1.91 to 1.58 ppm (blue), and the later (blue) shows a correlation with a ^{1}H multiplet signal around 3.75 ppm (orange). Additionally, the ^{11}B NMR spectrum of this reaction mixture shows a broad singlet at 29.3 ppm, closely resembling the signal observed for boronic acid **22** (Figure S67). Both ^{11}B chemical shifts are characteristic of three-coordinated boronic acids and esters (^{11}B δ: 25-33 ppm).[12]

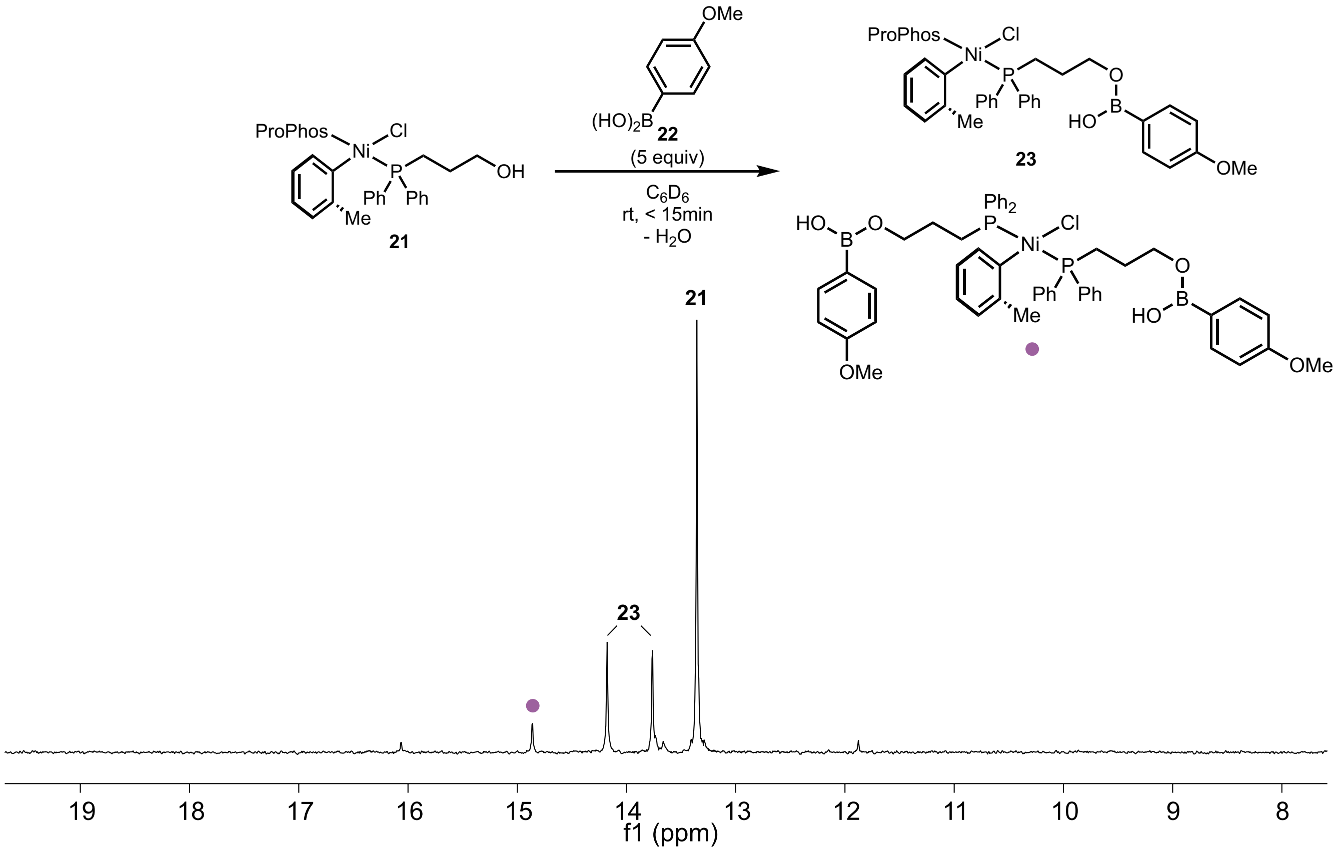

Figure S63 $^{31}P\{^{1}H\}$ NMR (162.04 MHz, C_6D_6) spectrum of the reaction of complex $Ni(PPh_2CH_2CH_2CH_2OH)_2Cl$(*o*-Tol) (**21**) with 4-OMe-$PhB(OH)_2$ (**22**, 5.0 equiv) at rt.

[12] Lennox, A. J. J.; Lloyd-Jones, G. C. Selection of Boron Reagents for Suzuki–Miyaura Coupling. *Chem. Soc. Rev.* **2014**, *43* (1), 412–443.

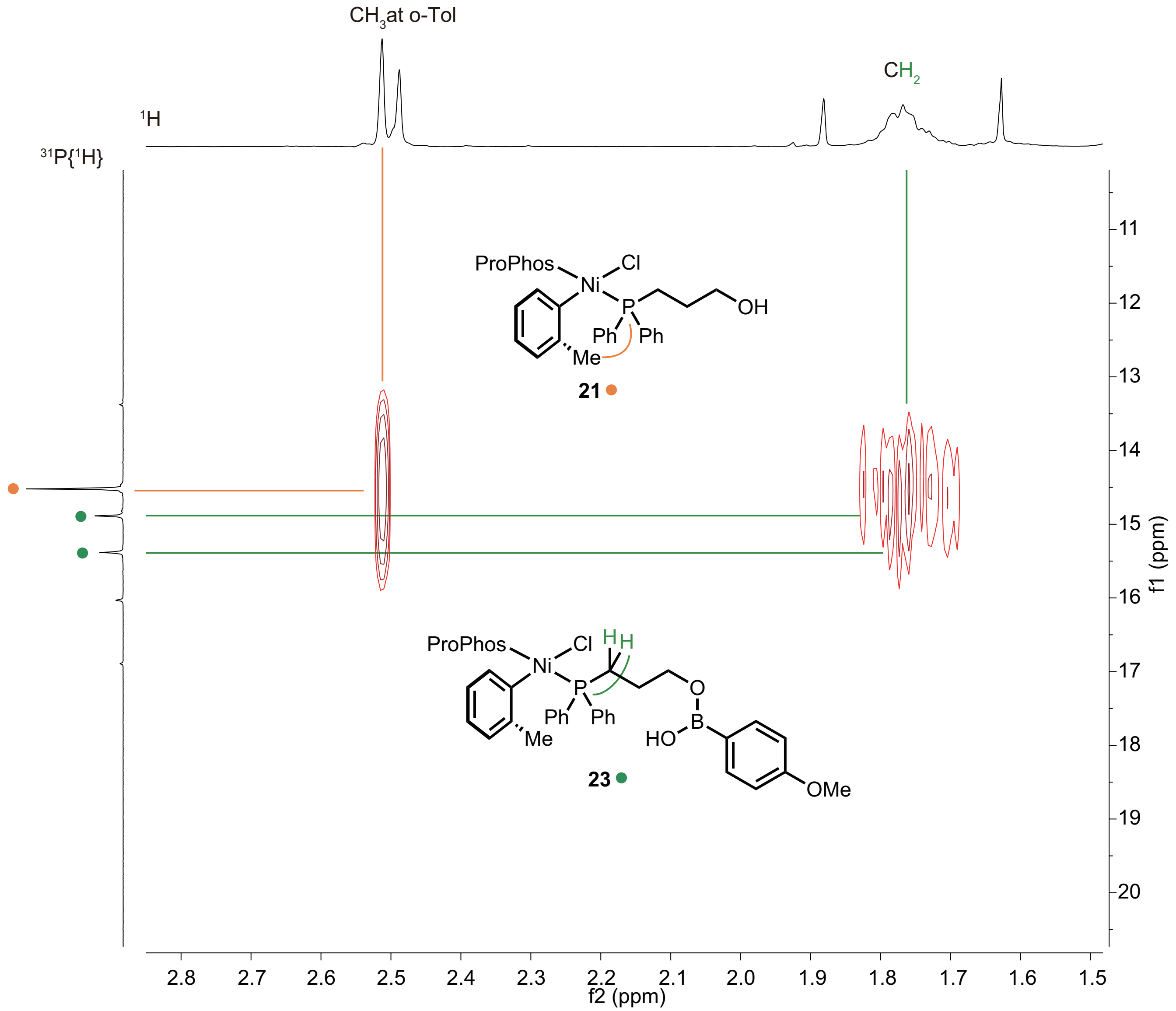

Figure S64 Partial $^{1}H/^{31}P\{^{1}H\}$-HMBC NMR (500.20 MHz, C_6D_6) spectrum for the reaction complex $Ni(PPh_2CH_2CH_2CH_2OH)_2Cl$(*o*-Tol) (**21**) with 4-OMe-PhB$(OH)_2$ (**22**, 5.0 equiv) at rt, showing the correlations between the P nuclei and its adjacent CH_2 protons on the proposed P-C-O-B ligand. The correlations due to complex **23** are heavily overlapped with those due to complex **21**.

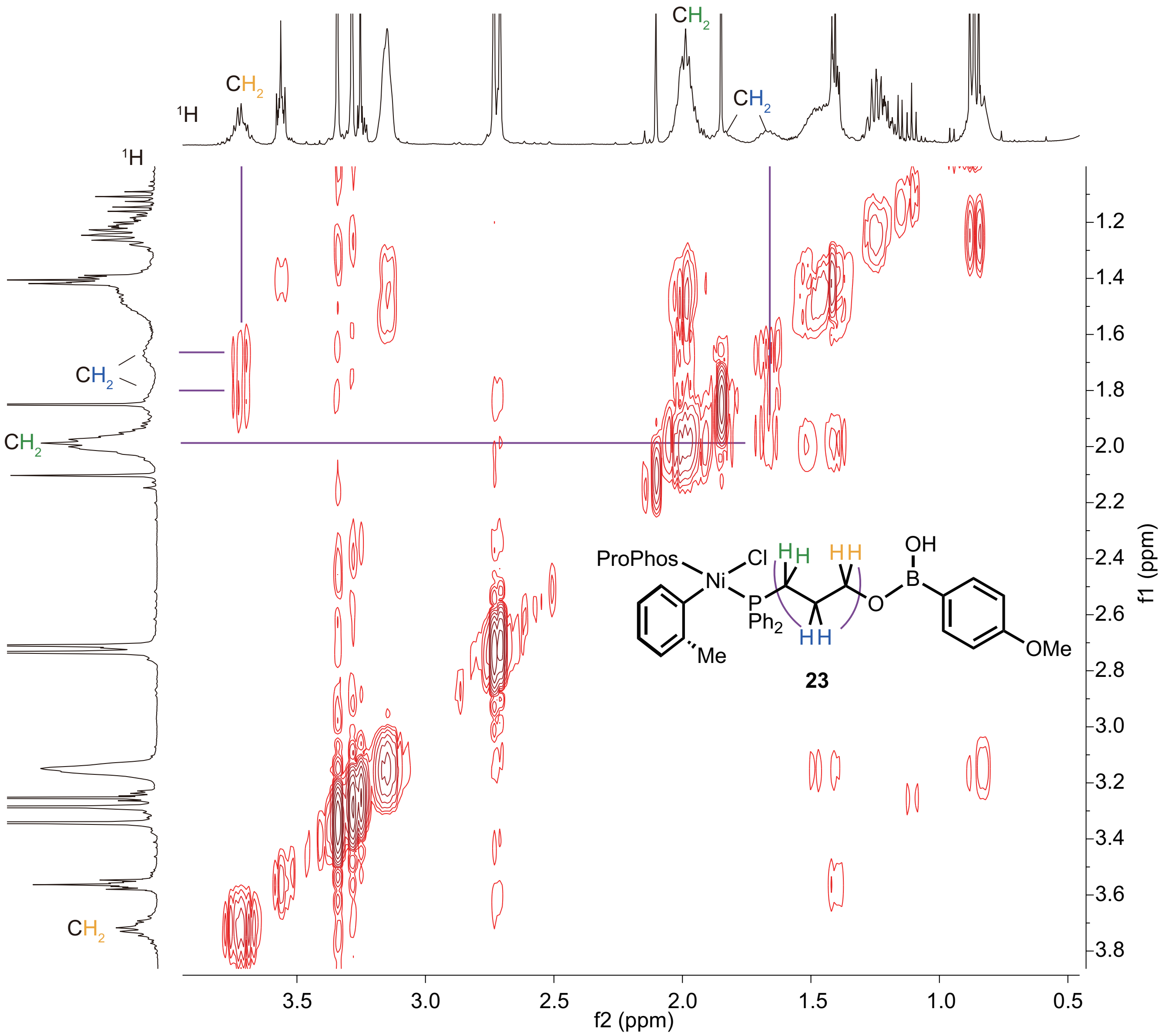

Figure S65 Partial ^{1}H COSY (400.30 MHz, C_6D_6) spectrum for the reaction of complex $Ni(PPh_2CH_2CH_2CH_2OH)_2Cl$(*o*-Tol) (**21**) with **22** (5.0 equiv) at rt, showing the correlations of CH_3 protons on the phosphine ligand.

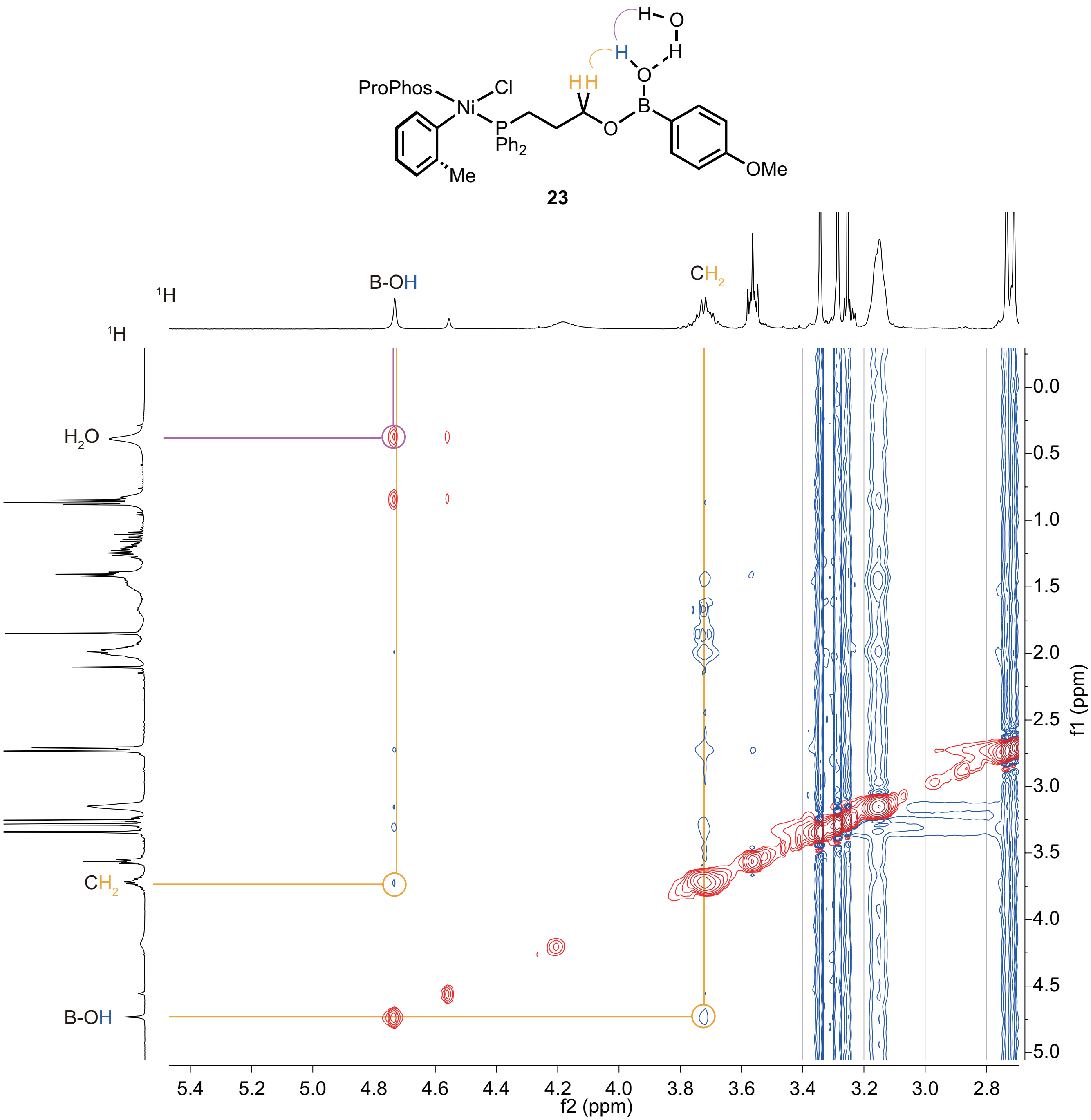

Figure S66 Partial ^{1}H NOESY (400.30 MHz, C_6D_6) spectrum for the reaction of complex $Ni(PPh_2CH_2CH_2CH_2OH)_2Cl$(*o*-Tol) (**21**) with **22** (5.0 equiv) at rt, showing a weak NOE (orange —) between CH_2 protons in the phosphine ligand and the OH proton in the boronic acid, as well as a strong EXSY (purple —) between the OH proton in the boronic acid and H_2O. (Note: The 2D EXSY technique is the same as the 2D NOESY technique. To differentiate between them, a straightforward approach is to observe the phase: in EXSY, off-diagonal peaks share the same phase as the diagonal peaks, whereas in NOESY, the off-diagonal peaks exhibit a different phase from the diagonal ones.)

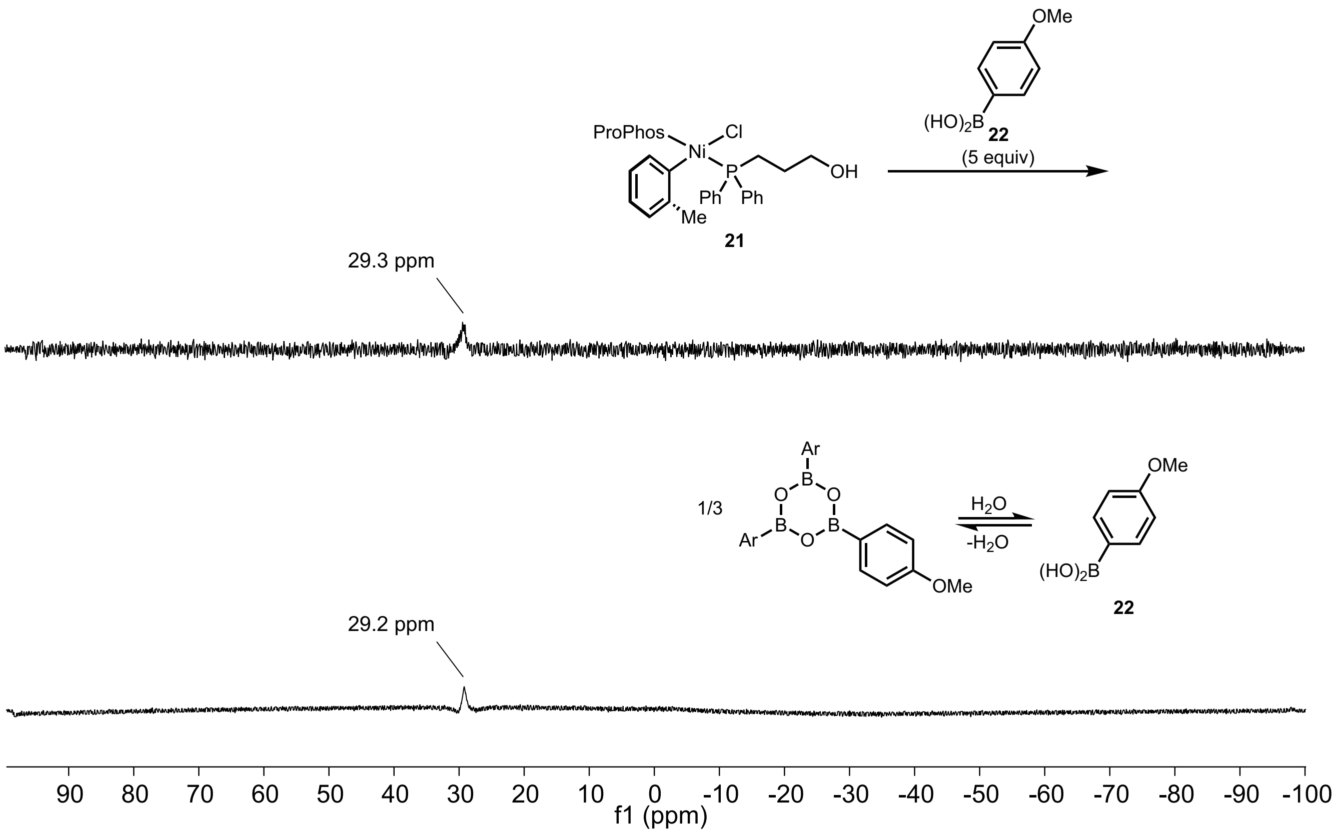

Figure S67 ^{11}B NMR (160.48 MHz, C_6D_6) spectra for the reaction mixture of complex $Ni(PPh_2CH_2CH_2CH_2OH)_2Cl$(*o*-Tol) (**21**) with **22** at rt (top) and the free boronic acid **22** (bottom).

5.2.2 Reaction of complex 21 with $BPh(OH)_2$ (6)

The addition of $BPh(OH)_2$ (**6**, 4.3 mg, 36 μmol, 5.0 equiv) to a C_6D_6 solution of complex **21** (4.8 mg, 7.1 μmol, 1.0 equiv) at rt produces a series of new complexes, analogous to the reaction observed with 4-methoxyphenylboronic acid. In the $^{31}P\{^1H\}$ NMR spectrum (Figure S68), we observed three major new signals at 14.9, 14.2 and 13.8 ppm, which shows identical chemical shifts to that observed in the reaction using 4-OMe-PhB$(OH)_2$ **22** (forming the analogous complex **S10**) However, the reaction with **6** shows more other unidentified product than that with **22**, as detected by $^{31}P\{^1H\}$ NMR (Figures S68 vs S63).

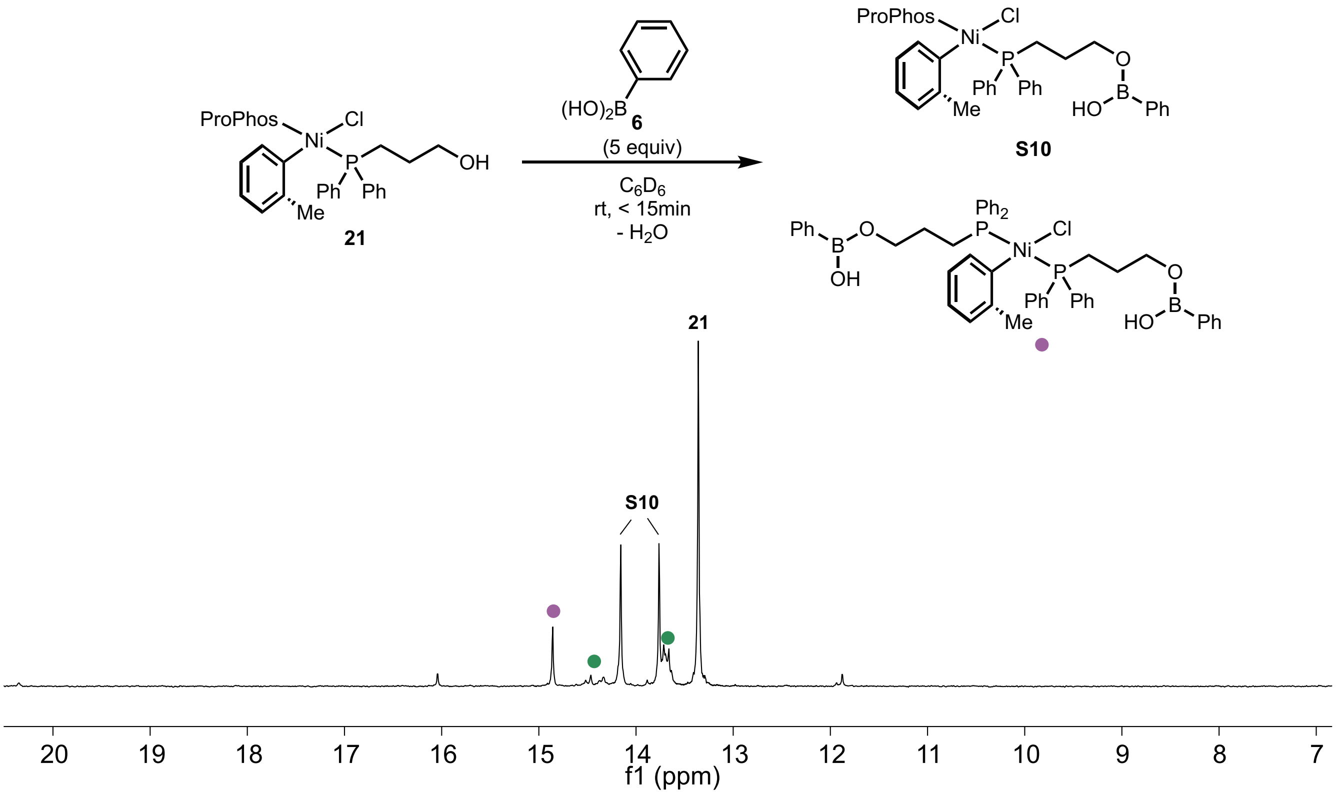

Figure S68 ${}^{31}P\{{}^{1}H\}$ NMR (162.04 MHz, C_6D_6) spectrum of the reaction of complex $Ni(PPh_2CH_2CH_2CH_2OH)_2Cl(o\text{-Tol})$ (**21**) with $BPh(OH)_2$ (**6**, 5.0 equiv) at rt. This reaction mixture is more complicated than the reaction observed with 4-methoxyphenylboronic acid **22**, unidentified species is marked as green dots.

In Figure S69a, the ${}^{1}H$ NMR spectrum of $BPh(OH)_2$ (**6**) shows an OH signal at approximately 3.99 ppm and aromatic ${}^{1}H$ signals at 7.65 and 7.21 ppm (H_a and $H_{b,c}$). However, in the ${}^{1}H$ NMR spectrum of the mixture containing complex **S10** (Figure S69b), the OH signal due to **6** is shifted further downfield to 5.05 ppm (blue). The aromatic ${}^{1}H$ signals of **6** are shifted in the ${}^{1}H$ NMR spectrum (Figure S69b) compared to that of free **6**, but these shifted signals are heavily overlapped with the signals due to the Ph at the phosphine ligand. Thus, we are unable to locate them in the ${}^{1}H$ spectrum. By comparing the spectrum (Figure S69b) to the ${}^{1}H$ NMR spectrum of the isolated **21** (Figure S69c), we found that: 1) the CH_2 signal at 3.16 ppm ($H_{d'}$, Figure S69c) shifts downfield to 3.72 ppm (H_d); 2) a signal due to CH_3 in the *o*-Tol group emerges at 2.71 ppm (H_e), which is slightly shifted more upfield compared to that in the isolated **21** ($H_{e'}$ at 2.74ppm).

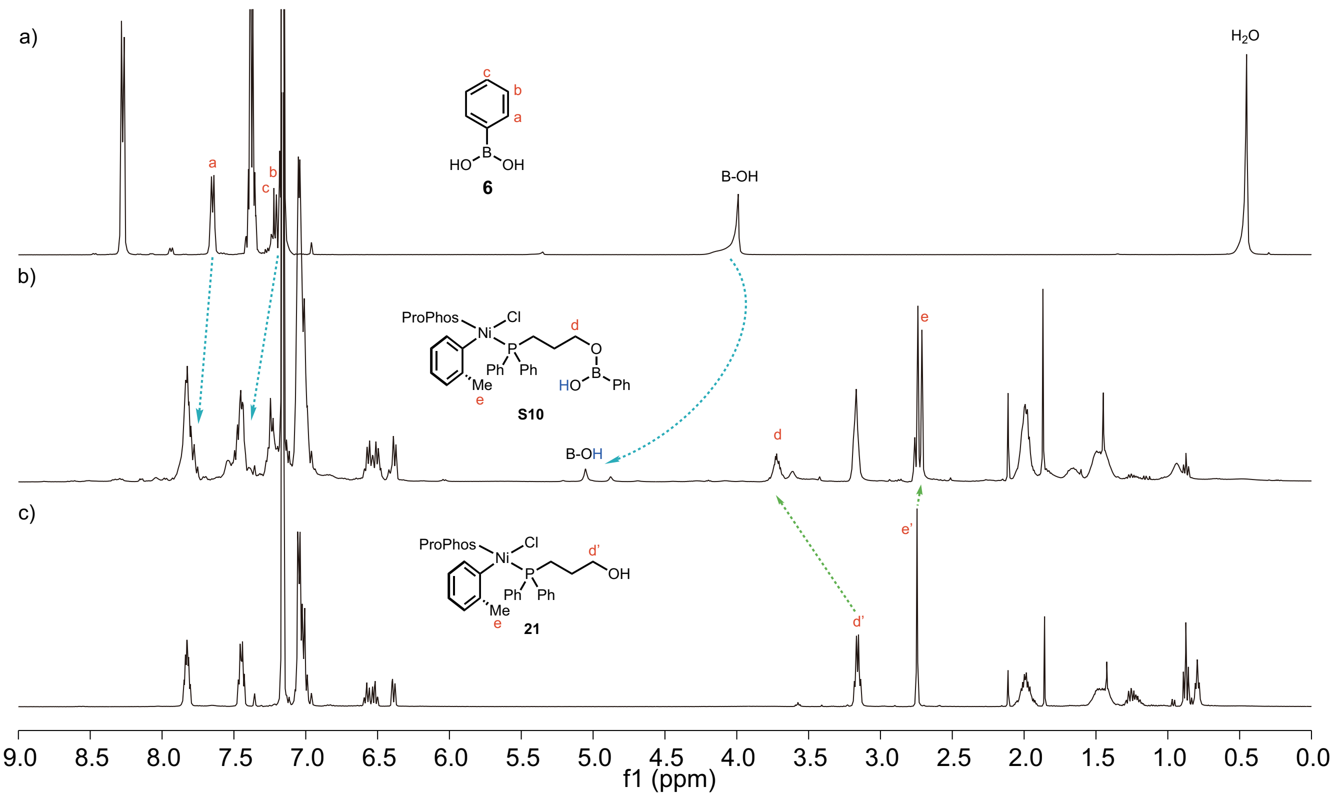

Figure S69 ^{1}H NMR (400.30 MHz, C_6D_6) spectra of: a) $BPh(OH)_2$ (**6**), which undergoes an equilibrium with its corresponding boroxine and H_2O in C_6D_6 [13]; b) the reaction of complex $Ni(PPh_2CH_2CH_2CH_2OH)_2Cl$(*o*-Tol) (**21**) with **6** (5.0 equiv) at rt; c) isolated complex $Ni(PPh_2CH_2CH_2CH_2OH)_2Cl$(*o*-Tol) (**21**) . The blue arrows indicate the peak shifts of the boronic acid **6**, and the green arrow indicates the peak shift of **21**.

5.2.3 Reactions of complex 21 with B(*p*-Tol)Pin (5)

We performed similar reactions of complex **21** (3.0 mg, 4.5 μmol, 1.0 equiv) with *p*-Tol boronic acid pinacol esters (**5**, 1.2 mg, 5.3 μmol, 1.2 equiv). As shown in Figure S70, both reactions show no new species even after 24 h.

[13] Antoft-Finch, A.; Blackburn, T.; Snieckus, V. *N,N*-Diethyl O-Carbamate: Directed Metalation Group and Orthogonal Suzuki−Miyaura Cross-Coupling Partner. *J. Am. Chem. Soc.* **2009**, *131* (49), 17750–17752.

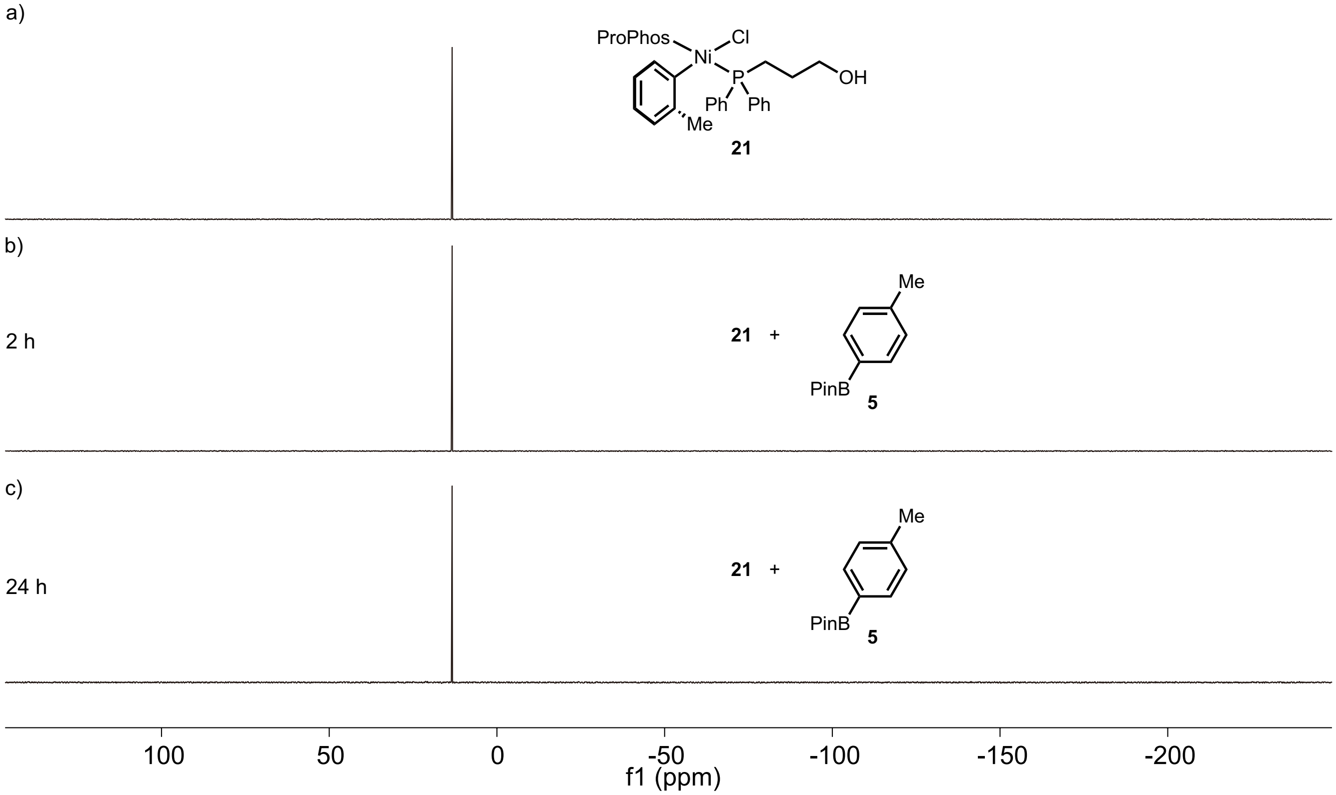

Figure S70 $^{31}P\{^1H\}$ NMR (162.04 MHz, C_6D_6) spectra of: a) isolated of complex Ni($PPh_2CH_2CH_2CH_2OH)_2$Cl(*o*-Tol) (**21**); b) the reaction of complex **21** with B(*p*-Tol)Pin (**5**, 1.2 equiv) at rt after 2 h; c) the reaction of complex **21** with B(*p*-Tol)Pin (**5**, 1.2 equiv) at rt after 24 h.

5.2.4 Reactions of complex Ni($PPh_2Me)_2$Cl(*o*-Tol) (13) with boronic acids and esters

We performed control experiments with Ni($PPh_2Me)_2$Cl(*o*-Tol) (**13**, 4.0 mg, 6.8 μmol, 1.0 equiv) and two boronic acids, 4-methoxyphenylboronic acid (**22**, 5.2 mg, 34 μmol, 5.0 equiv) and BPh$(OH)_2$ (**6**, 4.2 mg, 34 μmol, 5.0 equiv), respectively. Beside some unknown decomposition of the Ni starting material, both reactions show no significant change after 3 days (Figure S71-72). A similar reaction with B(*p*-Tol)Pin (**5**, 1.8 mg, 8.2 μmol, 1.2 equiv) also shows no significant change after several days.

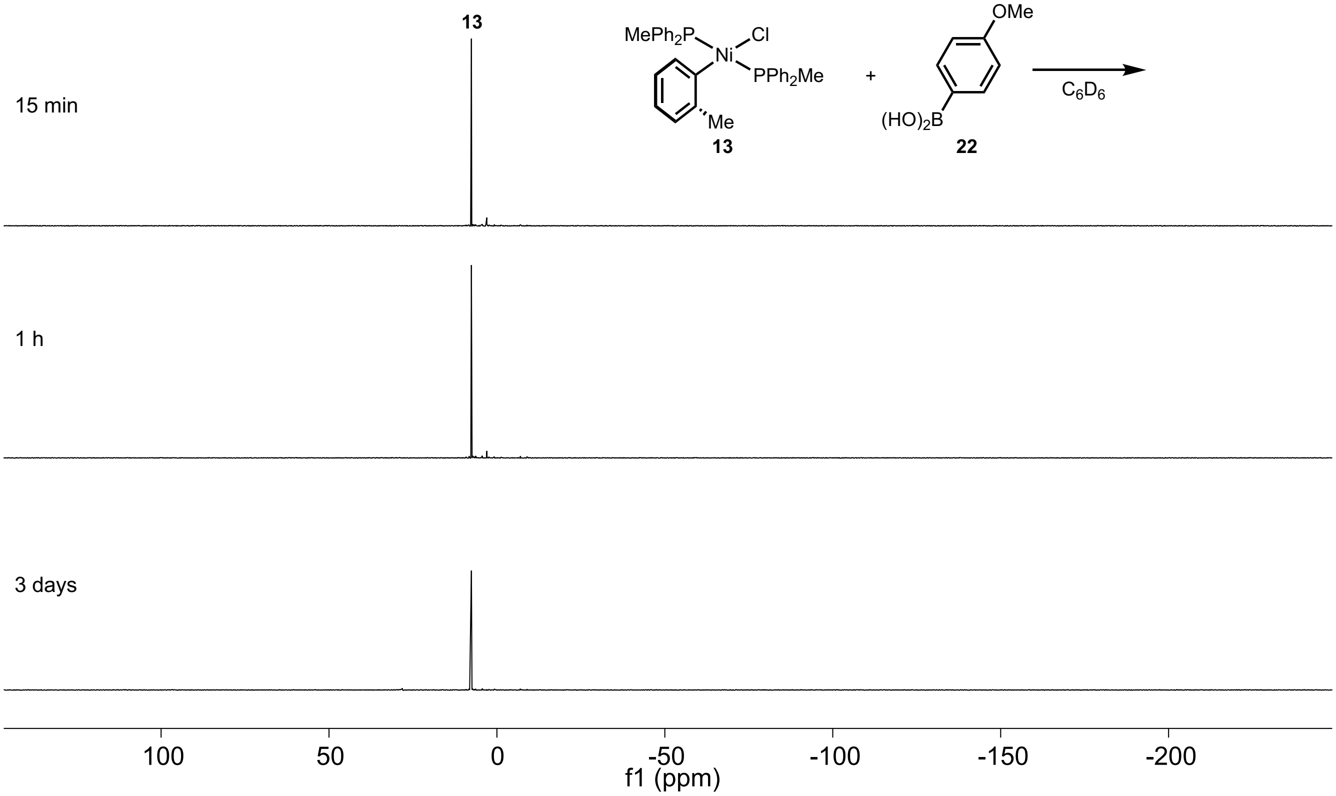

Figure S71 $^{31}P\{^{1}H\}$ NMR (162.04 MHz, C_6D_6) spectra of the reaction of complex $Ni(PPh_2Me)_2Cl$(*o*-Tol) (**13**) with 4-OMe-$PhB(OH)_2$ (**22**, 5.0 equiv) at rt, showing some decomposition of the Ni complex overtime.

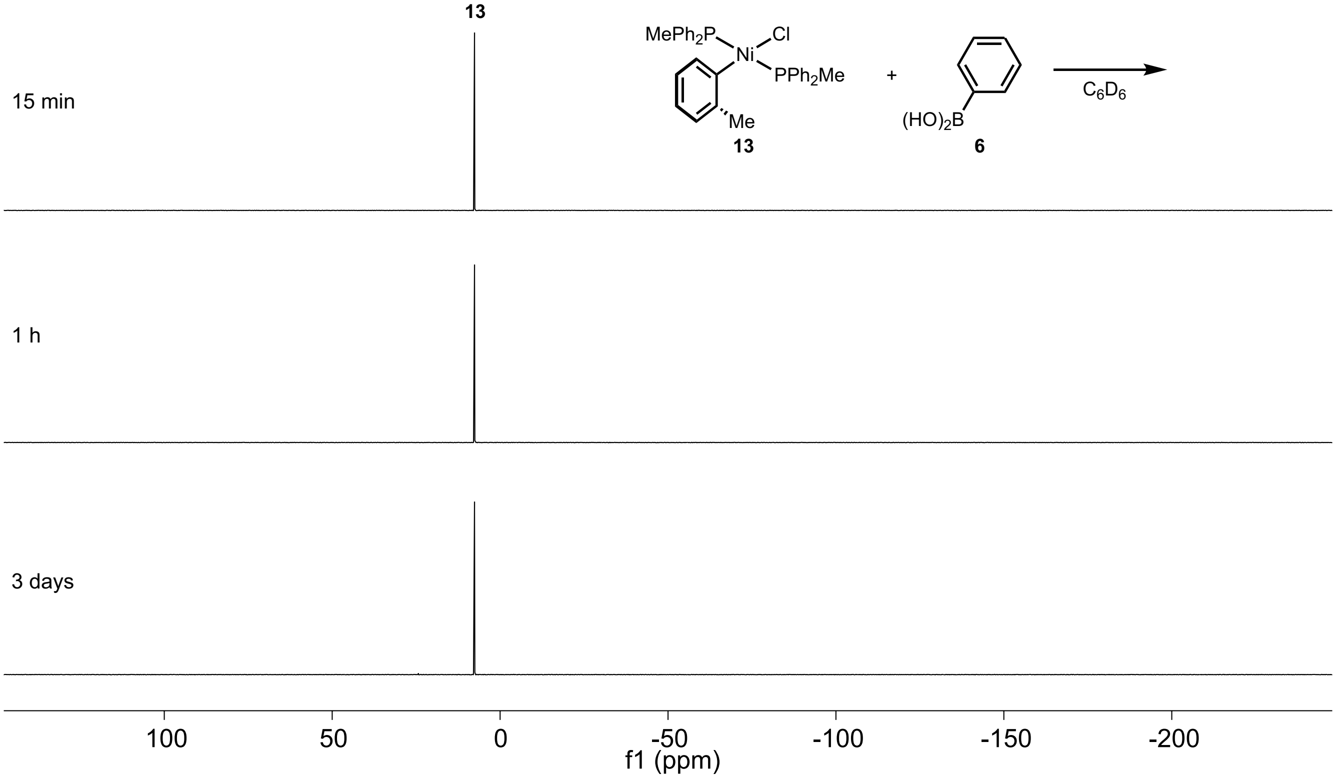

Figure S72 $^{31}P\{^{1}H\}$ NMR (162.04 MHz, C_6D_6) spectra of the reaction of $Ni(PPh_2Me)_2Cl$(*o*-Tol) (**13**) with $BPh(OH)_2$ (**6**, 5.0 equiv) at rt, showing minor decomposition of the Ni complex overtime.

5.2.5 Variable temperature NMR study of ProPhos (9) with 4-OMe-PhB(OH)$_2$ (22)

We conducted NMR experiments at various temperatures for the reaction mixture of free ligand **9** and boronic acid **22** to explore possible equilibria involving the B,O-coordination. It is important to highlight that: 1) high-temperature NMR studies with complex **21** and **22** were not carried out due to the rapid "base-free" transmetalation between **21** and **22** (vide infra); 2) analyzing the resulting B,O-adduct from the reaction mixture of **9** and **22** is considerably less intricate compared to that of **21** and **22**.

Room temperature experiments. Addition of 4-OMe-PhB(OH)$_2$ (**22**, 12 mg, 0.082 mmol, 4.0 equiv) to the isolated PPh$_2$CH$_2$CH$_2$CH$_2$OH (**9**, 5.0 mg, 0.020 mmol, 1.0 equiv) in C$_6$D$_6$ leads to an immediate formation of the phosphine-borane adduct PPh$_2$CH$_2$CH$_2$CH$_2$OB(4-OMe-Ph)(OH), as detected by ^{1}H (Figure S73b) and ^{31}P{^{1}H} NMR (Figure S74). All ^{1}H shifts for this phosphine-borane adduct are downfield shift compared to PPh$_2$CH$_2$CH$_2$CH$_2$OH (Figure S73b,c), which is consistent with previously identified Ni-P-O-B adducts **23** (Section 5.2.1). The ^{31}P{^{1}H} NMR spectrum (Figure S74) also shows a new signal at -16.3 ppm due to PPh$_2$CH$_2$CH$_2$CH$_2$OBPh(OH)$_2$, which is slightly shifted compared to that due to **9** (-16.1 ppm). ESI-MS analysis of this mixture shows a signal at m/z = 379.15, presumably matching the calculated m/z for C$_{22}$H$_{24}$BO$_3$P (379.16, *i.e.* PPh$_2$CH$_2$CH$_2$CH$_2$OB(4-OMe-Ph)(OH) + H$^+$). Furthermore, the ^{11}B NMR spectrum of this reaction mixture shows a broad singlet at 29.2 ppm.

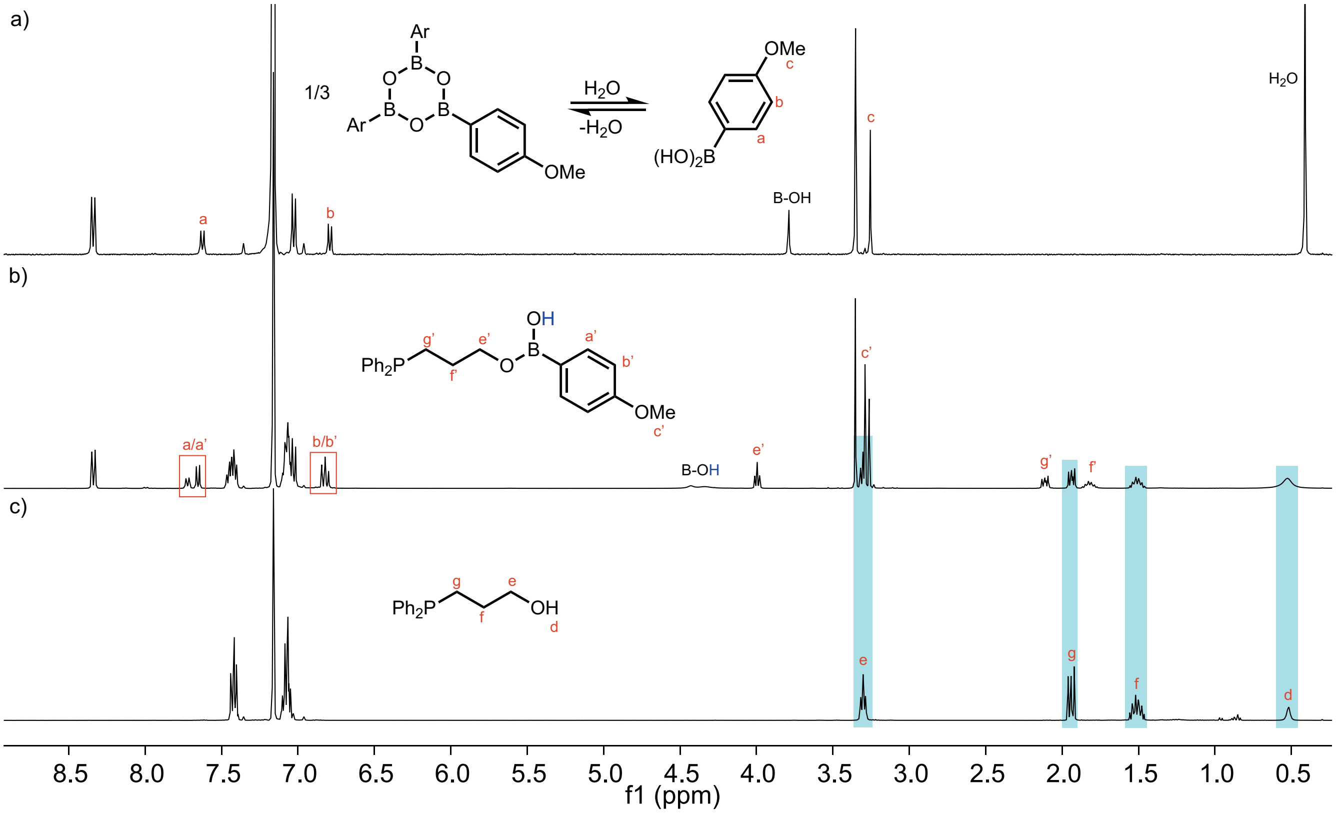

Figure S73 ^{1}H NMR (400.30 MHz, C$_6$D$_6$) spectra of: a) 4-OMe-PhB(OH)$_2$ **22**; b) the reaction of **9** with **6** at rt; c) isolated ProPhos **9**.

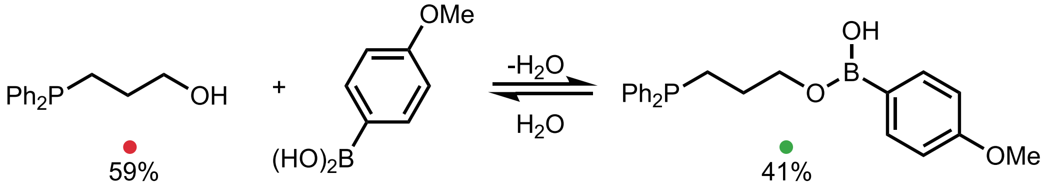

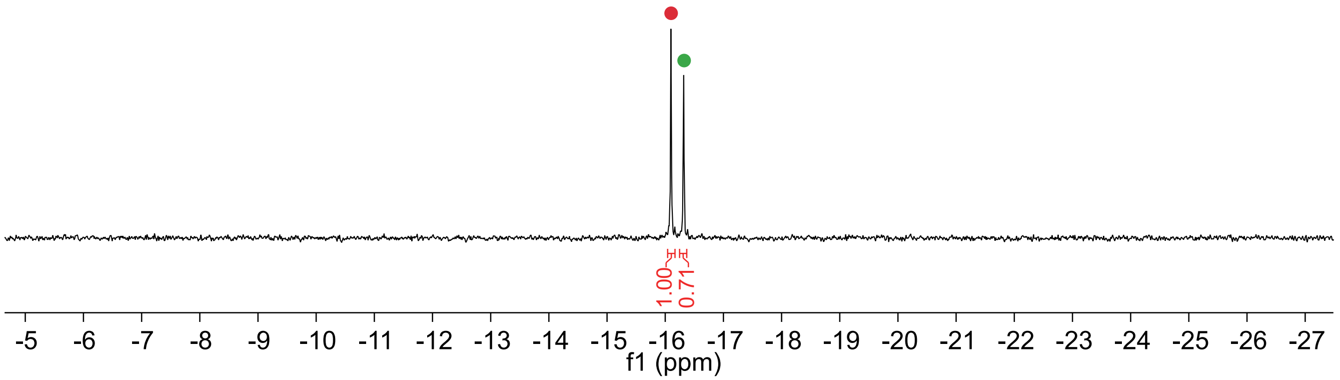

Figure S74 $^{31}P\{^{1}H\}$ NMR (162.04 MHz, C_6D_6) spectra of the reaction of **9** (red) with **22** at rt.

High temperature experiments. Heating the reaction mixture of ProPhos **9** and **22** gradually causes both ^{31}P signals due to **9** and its B,O-adduct shifted more downfield (Figure S75 right). Notably, the signal intensity of the B,O-adduct diminishes compared to that of **9**, suggesting a shift in equilibrium toward the side of **9** and boronic acid at higher temperatures. The VT-^{1}H NMR (Figure S75 left) spectra reveal the gradual merging of H_a (**22**) and H_c (B,O-adduct) signals with the temperature increasing, accompanied by a reduction in their intensities compared to the boroxine signal (H_b). These findings indicate an overall shift in equilibrium toward the boroxine at elevated temperatures, consistent with computational studies predicting the endothermic nature of boronic acid dehydration.[14] Cooling the sample back to 25°C restores the original spectrum, providing evidence for the reversibility of the B,O-coordination between ligand **9** and boronic acid **22**. It's worth noting that such equilibrium dynamics could be more intricate under catalytic conditions involving the presence of H_2O and a base.

[14] Bhat, K. L.; Markham, G. D.; Larkin, J. D.; Bock, C. W. Thermodynamics of Boroxine Formation from the Aliphatic Boronic Acid Monomers R–B(OH)$_2$ (R = H, H_3C, H_2N, HO, and F): A Computational Investigation. *J. Phys. Chem. A* **2011**, *115* (26), 7785–7793.

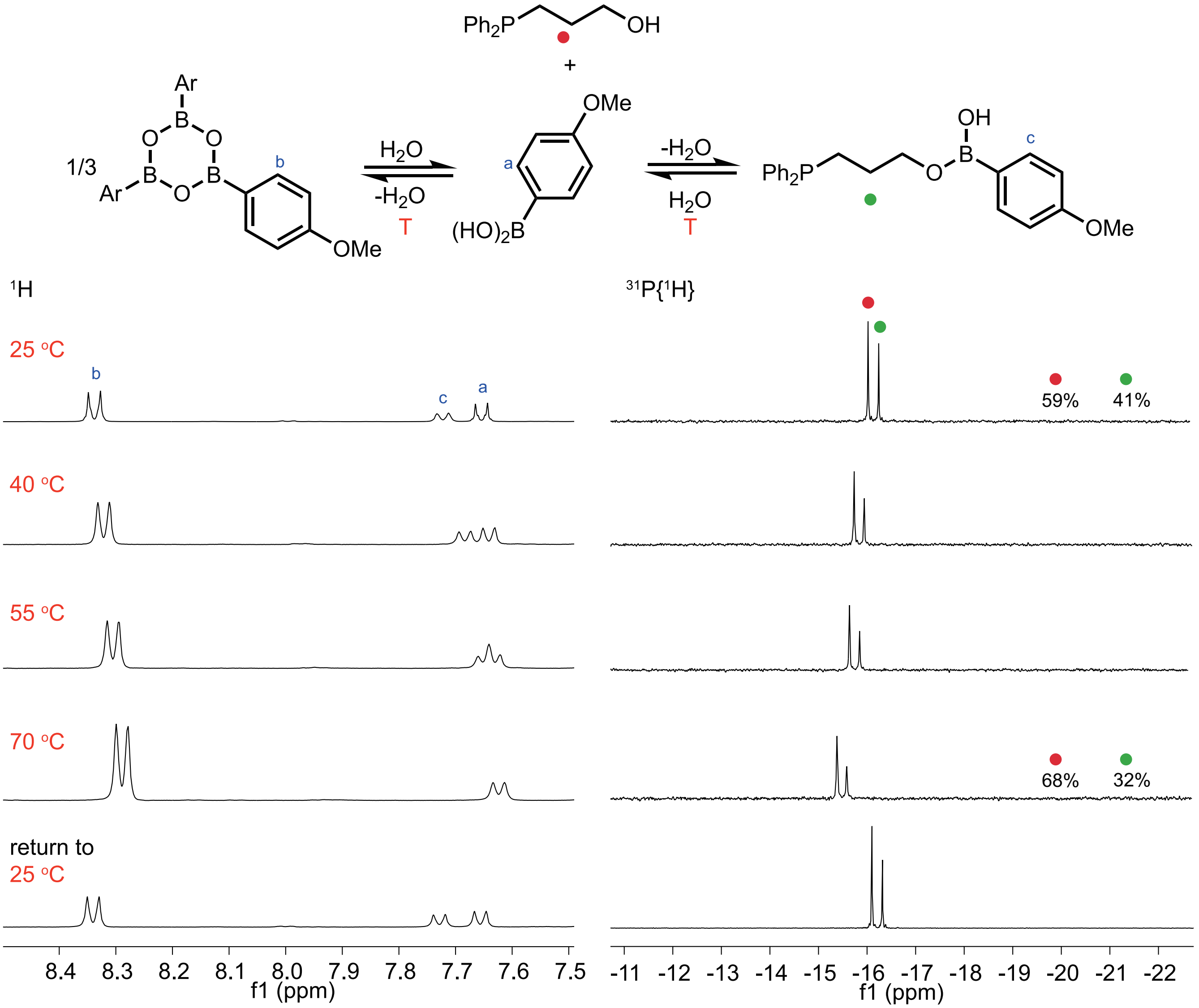

Figure S75 ^{1}H NMR (400.30 MHz, C_6D_6) and $^{31}P\{^{1}H\}$ NMR (162.04 MHz, C_6D_6) spectra of the reaction of **9** (red) with **22** at variable temperatures.

5.3 Reactivity studies of complex Ni($PPh_2CH_2CH_2CH_2OH)_2$Cl(*o*-Tol) (21) with KOH

5.3.1 Reaction of complex 21 with KOH in THF/H_2O

A vial was loaded with Ni($PPh_2CH_2CH_2CH_2OH)_2$Cl(*o*-Tol) (**21**, 22 mg, 0.032 mmol, 1.0 equiv) and KOH (26 mg, 0.64 mmol, 20 equiv). A mixture of THF:H_2O (20:1, 2 mL) was then added and the yellow solution was stirred at rt overnight. After that, the solvent was removed under vacuum and the residue was redissolved in pentane (2 mL), filtered through a Celite and the filtrate was stored was stored at -35 °C for 3 h. The yellow solid was suspended in pentane, collected by filtration to afford a yellow solid (12 mg, %yield is not determined due to the mixture, Scheme S1).

Scheme S1 Reaction of Complex **21** with KOH in THF/H_2O, Showing the Tentatively Assigned Products **24**.

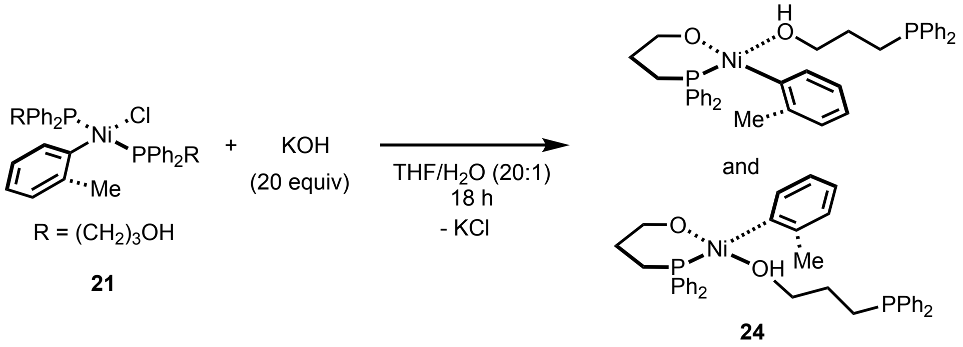

$^{31}P\{^1H\}$ NMR shows the mixture contains three signals (Figure S76c). The two signals at 21.8 and 22.4 ppm are assigned to the *trans*- and *cis*-isomers of Ni(κ^2-$\underline{P}Ph_2CH_2CH_2CH_2\underline{O}$)(*o*-Tol)(L) (**24**, L = $PPh_2CH_2CH_2CH_2OH$), based on analogous P,O-coordinated Ni complexes observed during the preparation of complex **21** (Figure S76b). The other signal at -16.3 ppm is raised by the coordination of $PPh_2CH_2CH_2CH_2OH$ through the OH group, supported by the comparison of ^1H signals of the OH group (H_a) and the H_b and that of free $PPh_2CH_2CH_2CH_2OH$ (Figure S77). The ^1H NMR spectrum shows no signals corresponding to Ni-OH or Ni-$PPh_2CH_2CH_2CH_2OH$ (Figure S78). Although full assignment is difficult due to peak overlap in the alkyl region, we observed two new ^1H signals due to the CH_3 groups at the Ni-Tol fragments (3.41 and 3.23 ppm), which are more downfield compared to that in the isolated Ni($PPh_2CH_2CH_2CH_2OH$)$_2$Cl(*o*-Tol) (**21**) (2.75 ppm).

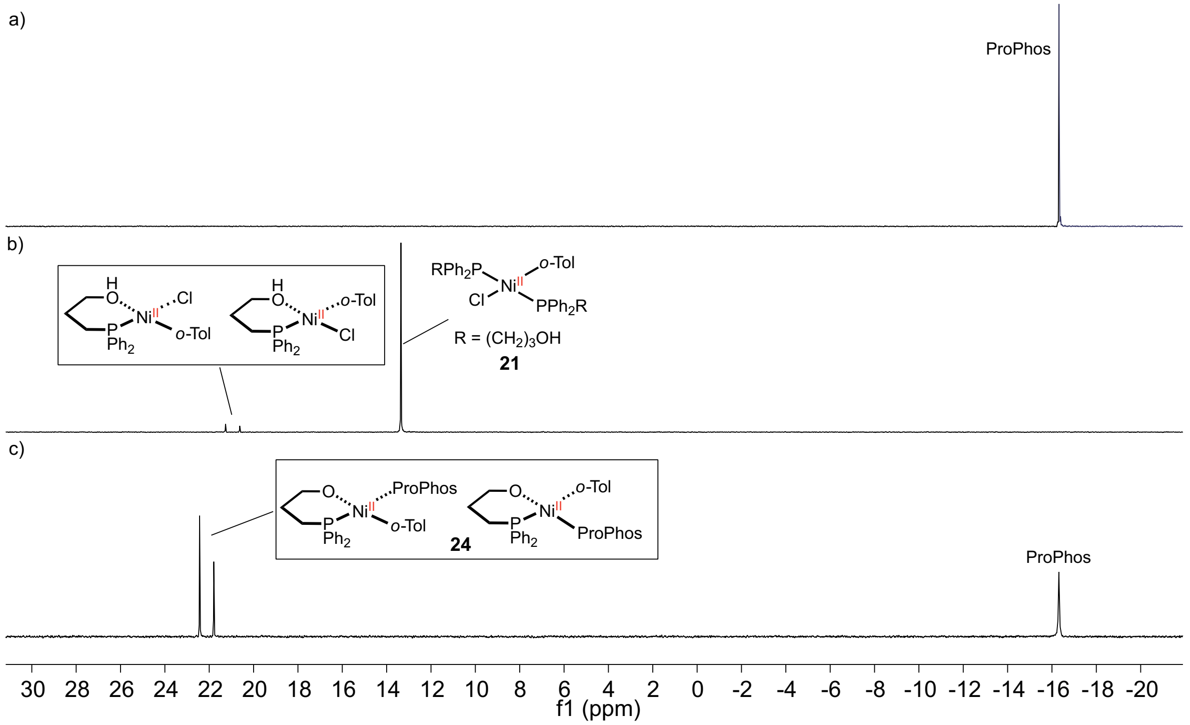

Figure S76 $^{31}P\{^1H\}$ NMR (162.04 MHz, C_6D_6) spectra of: a) isolated ProPhos **9**; b) the ligand substitution mixture of Ni(TMEDA)Cl(*o*-Tol) (**S9**) with ProPhos **9**; c) the reaction mixture of Ni($PPh_2CH_2CH_2CH_2OH$)$_2$Cl(*o*-Tol) with KOH in THF/H_2O.

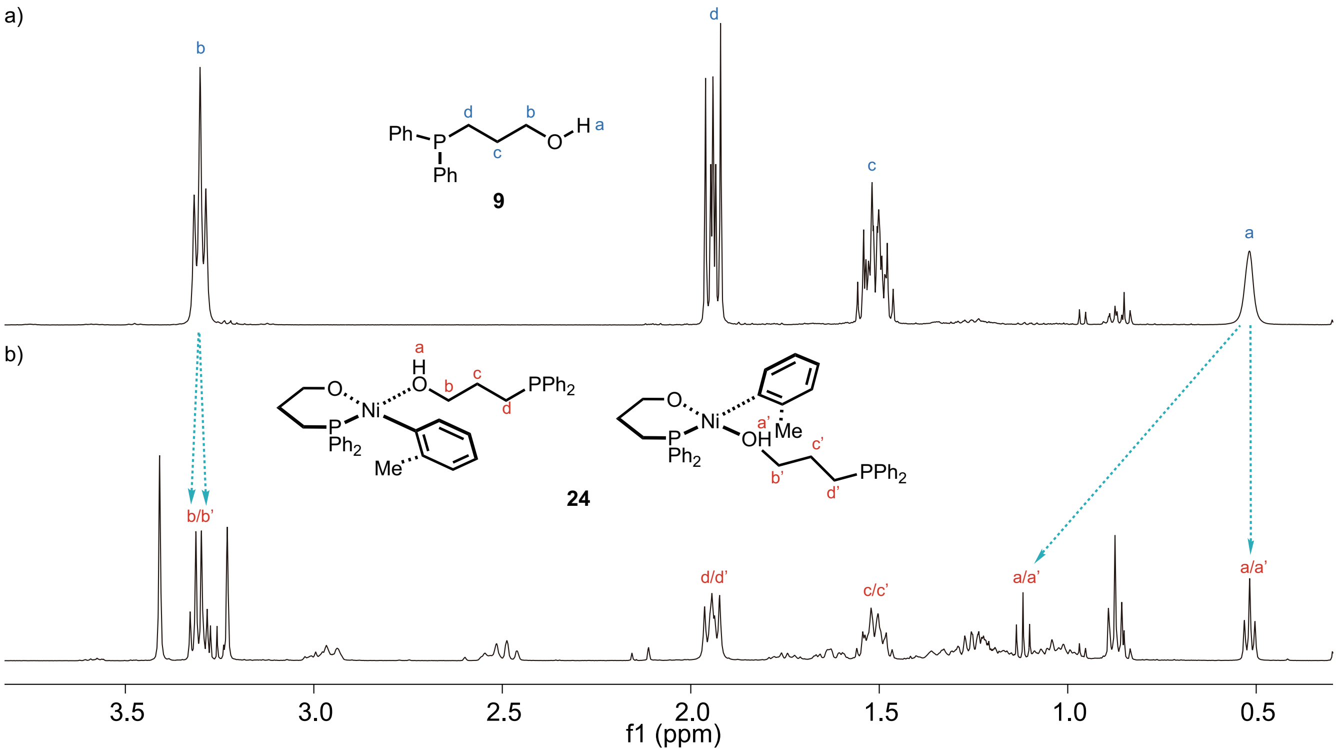

Figure S77 ^{1}H NMR (400.30 MHz, C_6D_6) spectrum of a) free ProPhos **9**; b) the reaction of the reaction mixture of $Ni(PPh_2CH_2CH_2CH_2OH)_2Cl$(*o*-Tol) (**21**) with KOH in THF/H_2O, showing the shift of the signals due to OH and its adjacent CH protons at **24** with respect to free **9**.

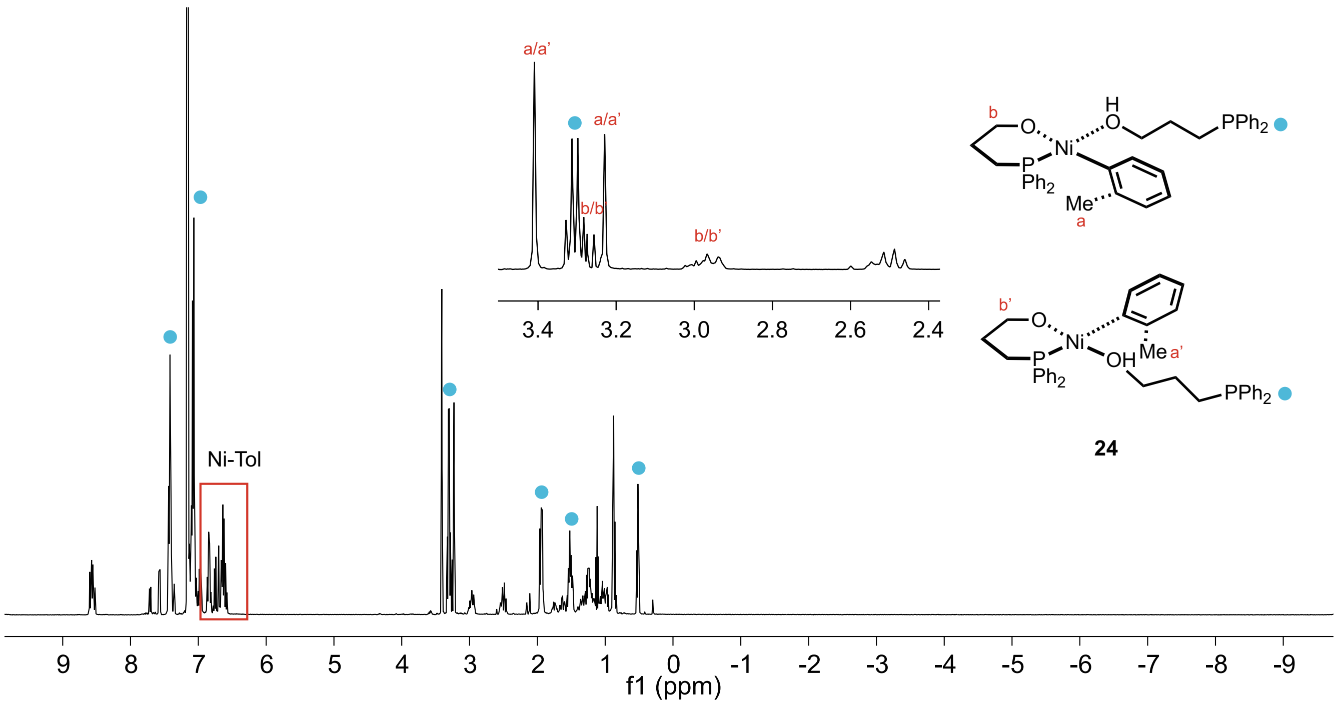

Figure S78 ^{1}H NMR (400.30 MHz, C_6D_6) spectrum of the mixture conation complex **24**, showing no Ni bridging OH formed (i.e., "empty" upfield region) and diagnostic signals in the tentatively assigned complexes. ProPhos ligand though O-coordination (•).

5.3.2 Reactivity of the mixture containing Ni(κ^2-<u>P</u>Ph$_2$CH$_2$CH$_2$CH$_2$<u>O</u>)(*o*-Tol)(ProPhos) (24) with B(*p*-Tol)Pin (5) or BPh(OH)$_2$ (6)

When we mixed Ni(κ^2-<u>P</u>Ph$_2$CH$_2$CH$_2$CH$_2$<u>O</u>)(*o*-Tol)(ProPhos) (**24**, 5.0 mg, 7.8 μmol, 1.0 equiv) and BPh(OH)$_2$ (**6**, 2.0 mg, 16 μmol, 2.0 equiv), a new Ni species formed within 15 min along with the disappearance of the two Ni isomers in the ^{31}P{^{1}H} NMR spectrum. This new species at 21.6 ppm is assigned to a B,O-coordinated adduct (Figure S79), based on the downfield shift and broadening of the ^{1}H signal due to boronic acid coordination (see detail in Section 5.2). The transmetalation products after 1 h were analyzed by GC, showing 22% of the desired product **19** and 22% of biphenyl (homocoupling product).

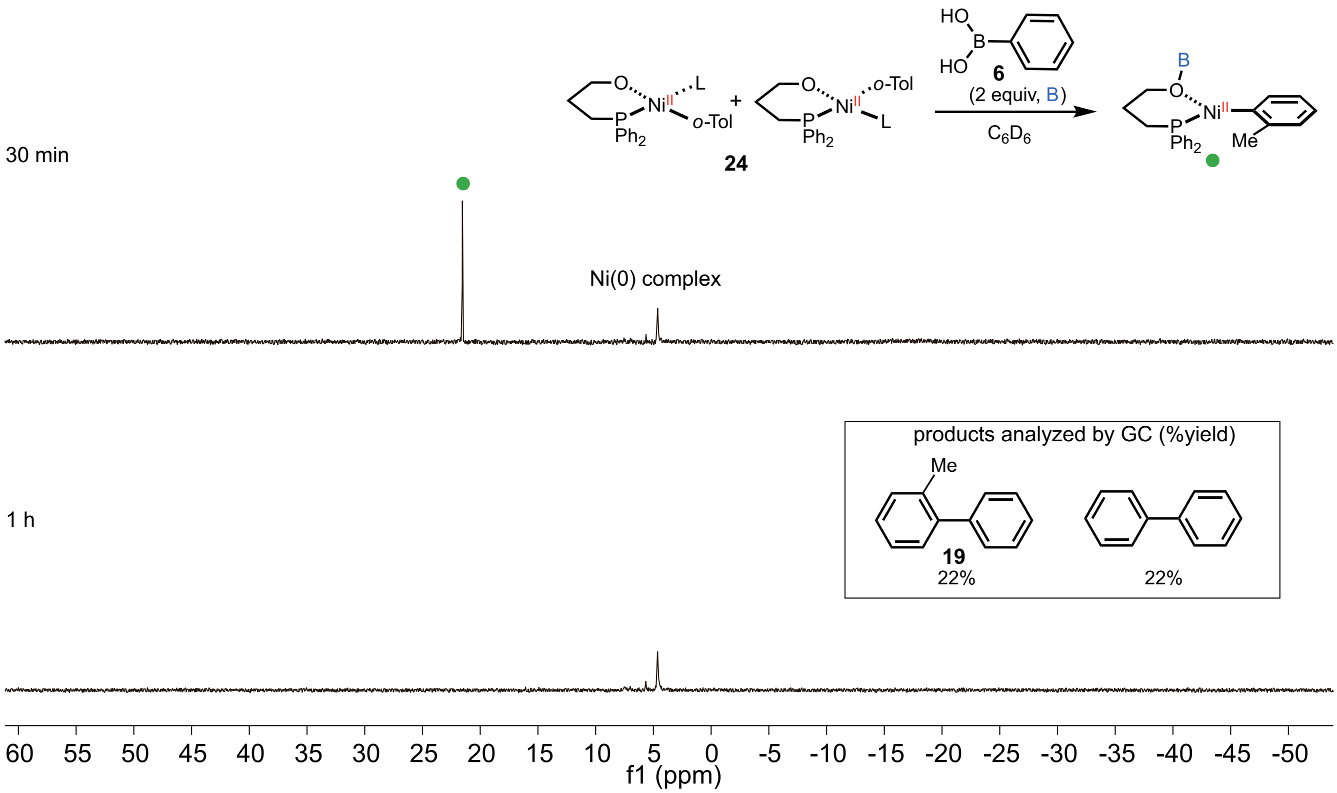

Figure S79 ^{31}P{^{1}H} NMR (162.04 MHz, C$_6$D$_6$) spectra of the reaction of the mixture containing Ni(κ^2-<u>P</u>Ph$_2$CH$_2$CH$_2$CH$_2$<u>O</u>)(*o*-Tol)(ProPhos) (**24**) with BPh(OH)$_2$ (**6**) and the resulting biaryl products. L = ProPhos.

5.4 Transmetalation studies of Ni-PPh$_2$Me complexes (13 and 16) and Ni-ProPhos complex (21)

General experimental procedure for the product analysis using GC

Ni(PPh$_2$Me)$_2$Cl(*o*-Tol) (**13**, 6.8–10 μmol, 1.0 equiv) or [Ni(PPh$_2$Me)(μ-OH)(*o*-Tol)]$_2$ (**16**, 3.0-4.1 μmol, 1.0 equiv) or Ni(PPh$_2$CH$_2$CH$_2$CH$_2$OH)$_2$Cl(*o*-Tol) (**21**, 5.2-10 μmol, 1.0 equiv) and PPh$_2$Me (if applicable, 2.0 equiv), were dissolved in THF (1 mL) for 30 min in a 4 mL vial with a stir bar. *p*-Tolylboronic acid pinacol ester (**5**, 2 equiv) or phenylboronic acid (**6**, 2–5.0 equiv) or the corresponding potassium aryl boronate (K[B(*p*-Tol)Pin(OH)] **15**, 2.0 equiv) was added to the Ni solution. For the reactions with boronate **15**, 18-crown-6 (1 equiv, if applicable) was added for facilitate its solubility in

THF. Dry K_3PO_4 (10 equiv) and H_2O (10 μL) were added (if applicable). An aliquot (50 μL) was analyzed by GC with an internal standard (*n*-decane).

General experimental procedure for the Ni speciation analysis by NMR

Subsequent to the general protocol above for product analysis, a minor adjustment was implemented to enhance shimming. The sample was then meticulously prepared in an NMR tube. Dry K_3PO_4 or KOH (10 equiv) and H_2O (1 μL) were used. Additionally, C_6D_6 or C_7D_8 (0.1 mL, for locking purpose) were added. After preparation, the sample was placed in an NMR tube shaker. Monitoring was carried out using $^{31}P\{^1H\}$ NMR.

5.4.1 Synthesis of K[B(*p*-Tol)Pin(OH)] (15)

The synthesis is slightly modified from the literature procedure.[15] To a 20 mL vial with a stir bar, **5** (400 mg, 1.83 mmol, 1.0 equiv), potassium hydroxide (103 mg, 1.83 mmol, 1.0 equiv), and MeCN (5 mL) were sequentially added. The reaction was rigorously stirred at 70 °C for 16 h. The solution was filtered by a frit, and the precipitate was washed with hexane (5 x 5 mL). The precipitate was dried under vacuum to yield the product as a white solid (355 mg, 1.29 mmol, 71%).

^{1}H NMR (400.30 MHz, D_2O) δ: 7.46 (d, *J* = 7.5 Hz, 2H, H_o), 7.11 (d, *J* = 7.5 Hz, 2H, H_m), 2.28 (s, 3H, CH_3 at *o*-Tol), 1.91 (s, 1H, OH), 1.22 (s, 6H, CH_3), 1.04 (s, 6H, CH_3).

$^{13}C\{^1H\}$ NMR (100.67 MHz, D_2O) δ: 135.3 (s, Ar at *o*-Tol), 131.4 (s, C_o at *o*-Tol), 127.8 (s, C_m at *o*-Tol), 75.6 (BO*C*(CH_3)$_2$), 23.7 (s, CH_3 at BPin), 20.1 (s, CH_3 at *o*-Tol). One quaternary ^{13}C signal at Tol group is not observed.

HRMS (ESI-TOF, CH_3OH) m/z: [M -KOH + Cl]$^-$ calculated for $C_{13}H_{19}BClO_2$ 253.1175, found 253.1188.

Note: The boronic ester **5** is barely soluble in D_2O, suggesting that NMR data we obtained is from the boronate **15**.

[15] Mills, L. R.; Gygi, D.; Simmons, E. M.; Wisniewski, S. R.; Kim, J.; Chirik, P. J. Mechanistic Investigations of Phenoxyimine–Cobalt(II)-Catalyzed C(Sp^2)–C(Sp^3) Suzuki–Miyaura Cross-Coupling. *J. Am. Chem. Soc.* **2023**, *145* (31), 17029–17041.

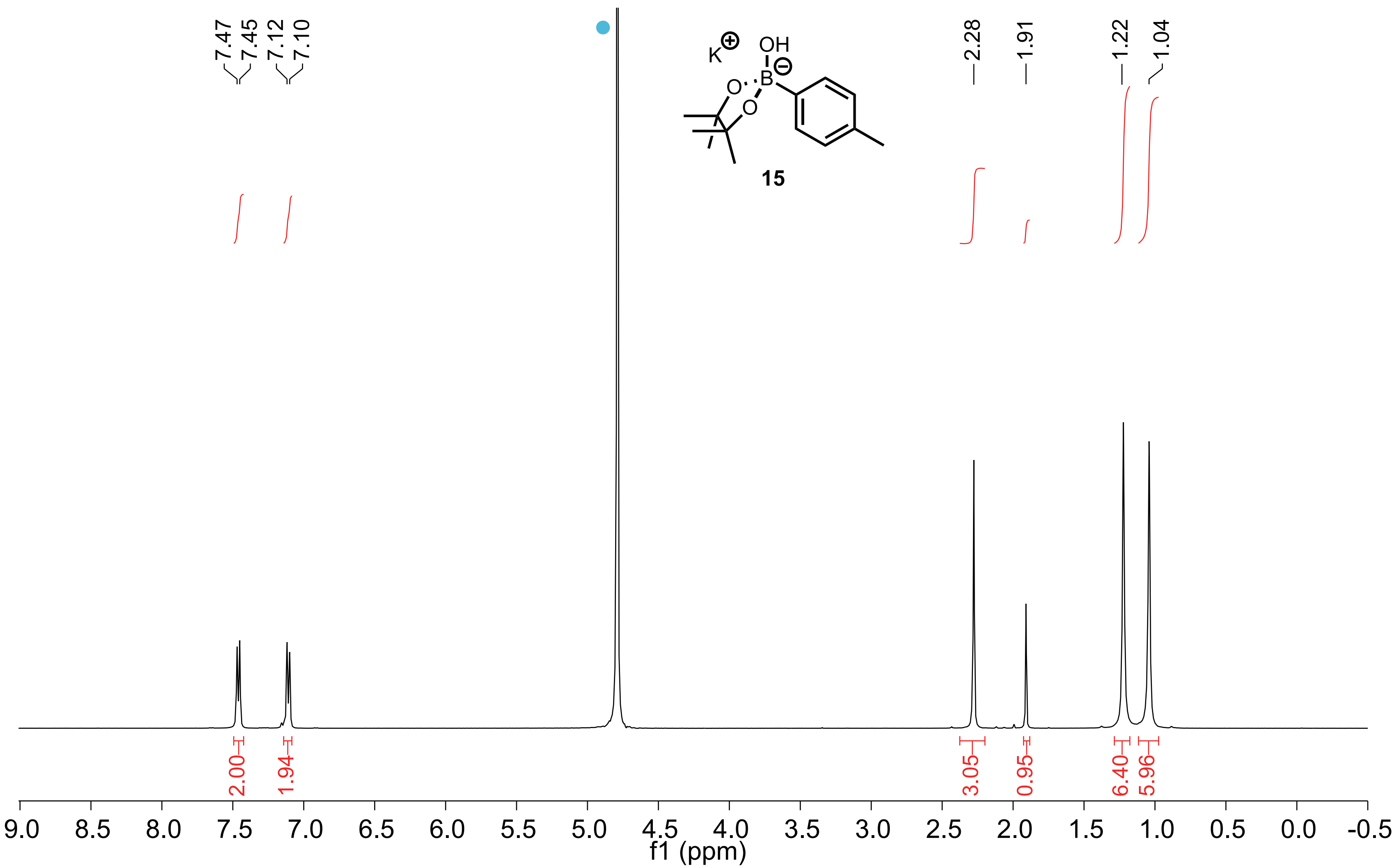

Figure S80 ^{1}H NMR (400.30 MHz, D_2O) spectrum of the isolated K[B(*p*-Tol)Pin(OH)] (**15**). Residual proteo-solvent (•).

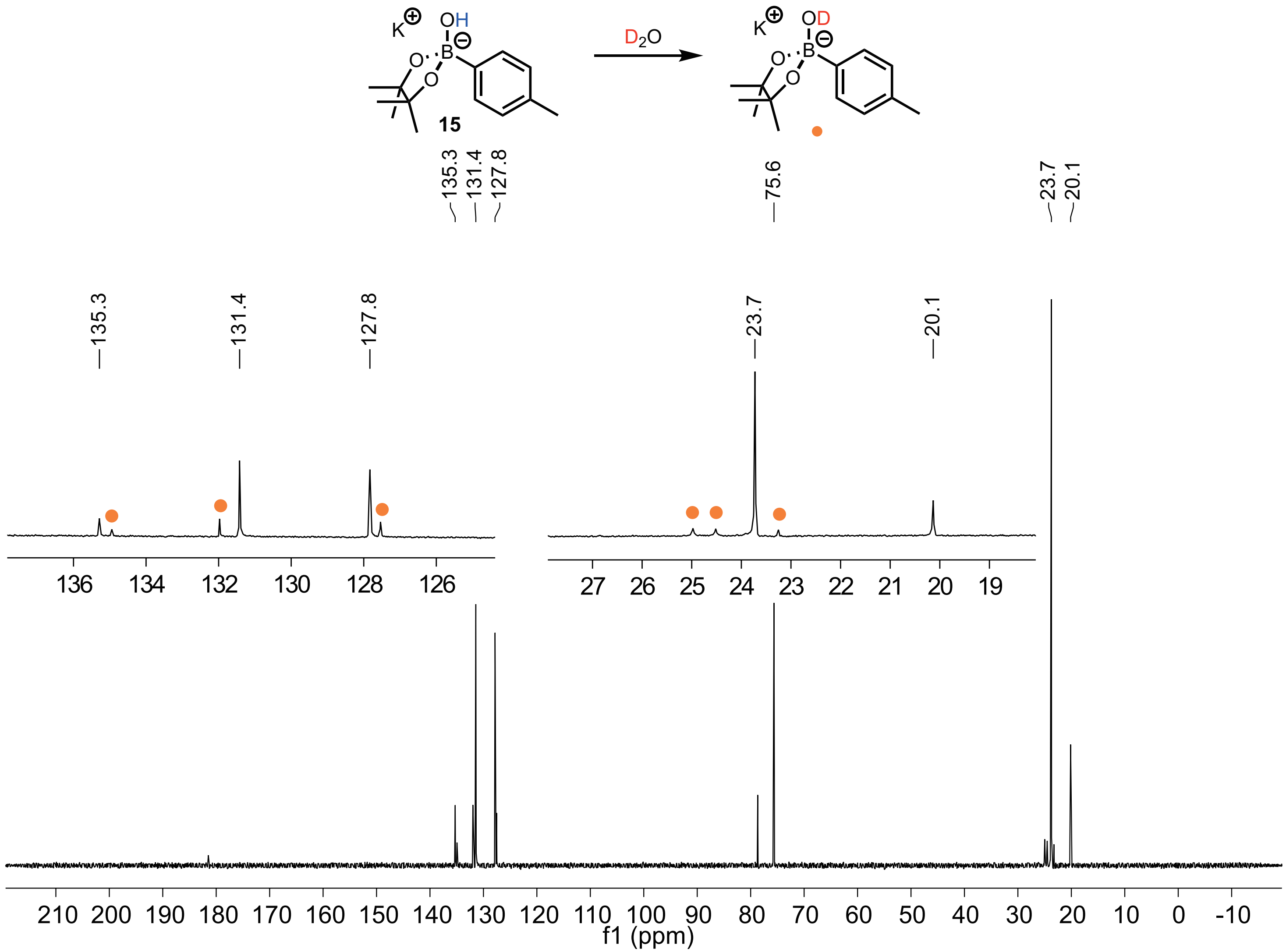

Figure S81 $^{13}C\{^1H\}$ NMR (100.67 MHz, D_2O) spectrum of the isolated K[B(*p*-Tol)Pin(OH)] (**15**), showing K[B(*p*-Tol)Pin(OD)] (●) is a possible minor species that likely comes from deprotonation of D_2O in the NMR solvent by **15**. A similar observation was reported in the literature.[16]

[16] Mills, L. R.; Gygi, D.; Ludwig, J. R.; Simmons, E. M.; Wisniewski, S. R.; Kim, J.; Chirik, P. J. Cobalt-Catalyzed C(Sp^2)–C(Sp^3) Suzuki–Miyaura Cross-Coupling Enabled by Well-Defined Precatalysts with L,X-Type Ligands. *ACS Catal.* **2022**, *12* (3), 1905–1918.

5.4.2 Reactions of complex Ni(PPh$_2$Me)$_2$Cl(*o*-Tol) (13) with B(*p*-Tol)Pin (5), BPh(OH)$_2$ (6) and K[B(*p*-Tol)Pin(OH)] (15)

As shown in Figure S82, the reactions of Ni(PPh$_2$Me)$_2$Cl(*o*-Tol) (**13**) with B(*p*-Tol)Pin (**5**) with or without K_3PO_4 show nearly no formation of the desired biaryl product even after 24 h (orange and pink). The reaction of **13** with K[B(*p*-Tol)Pin(OH)] (**15**) shows a gradient product formation up to 29% over 24 h (cyan). Additionally, transmetalation of **13** using boronic acid **6** (blue and red) is faster than those using boronic esters.

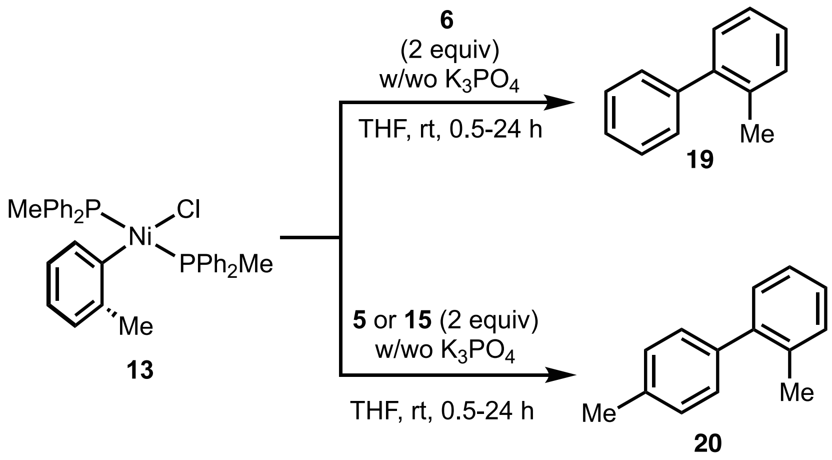

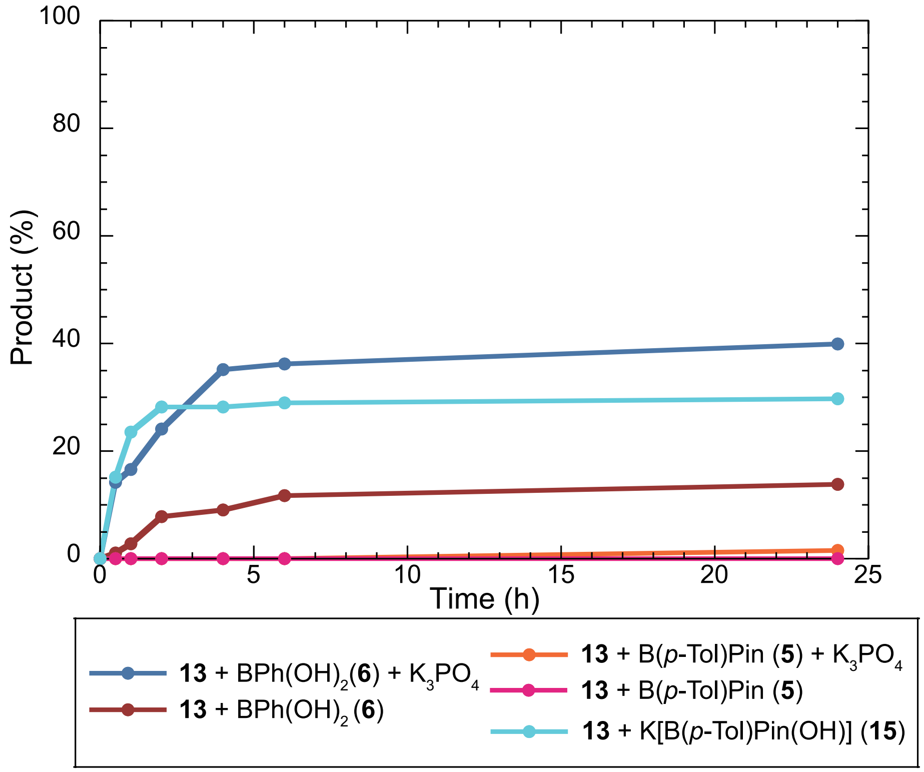

Figure S82 Stoichiometric transmetalation of complex Ni(PPh$_2$Me)$_2$Cl(*o*-Tol) (**13**) with B(*p*-Tol)Pin (**5**), BPh(OH)$_2$ (**6**) and KB[(*p*-Tol)Pin(OH)] (**15**). %Yields of products **19** and **20** determined by GC.

We attribute the faster transmetalation rate using boronate **15** to the boronate's better solubility in THF. Boronate serves as a base to facilitate the formation of Ni-OH. We measured the pH of K_3PO_4 and boronate solutions in THF. Under these transmetalation conditions, we found that the pH for the boronate reaction was around 9. In contrast, with an excess of K_3PO_4, even upon the addition of water, the concentration of OH anion remained too low to be detected by pH paper. Our findings align with a previous investigation involving a specific system (THF-water-Cs_2CO_3).[17] Under catalytic conditions, the discernible difference in pH values between the phases affects the speciation of boron species, resulting in a higher concentration of boronic acid or ester in the bulk phase (also see Section 3.3).

5.4.3 Reactions of complex [Ni(PPh$_2$Me)(μ-OH)(*o*-Tol)]$_2$ (16) with B(*p*-Tol)Pin (5), BPh(OH)$_2$ (6) and K[B(*p*-Tol)Pin(OH)] (15)

Scheme S2 Literature Precedents of Monomeric and Dimeric Ni-OH Complexes.[18,19]

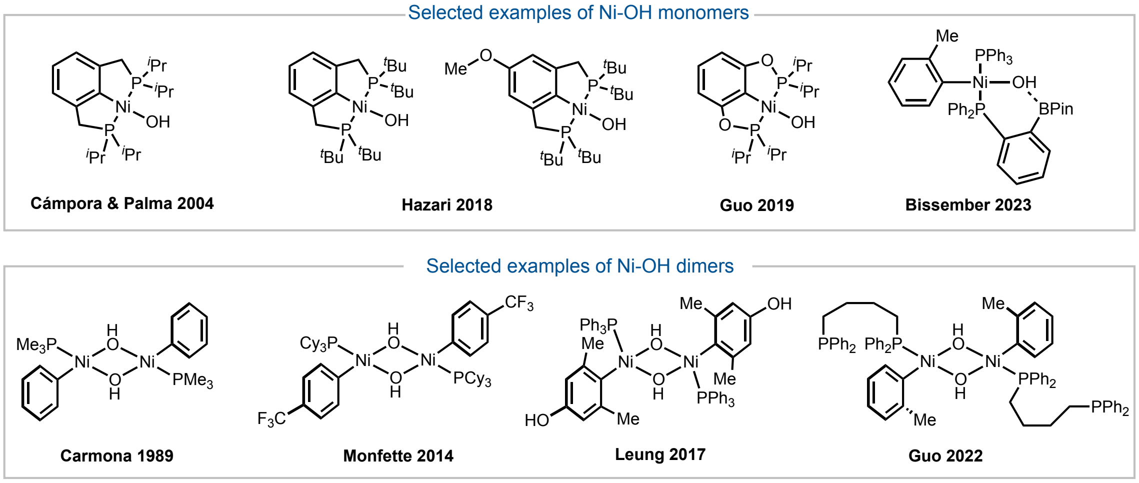

As illustrated in Figure S83, the reactivity of Ni-OH complex **16** towards both boronic acid **6** and ester **5** is higher than that of complex **13**. Notably, the transmetalation reaction of complex **16** with

[17] Lennox, A. J. J.; Lloyd-Jones, G. C. Organotrifluoroborate Hydrolysis: Boronic Acid Release Mechanism and an Acid–Base Paradox in Cross-Coupling. *J. Am. Chem. Soc.* **2012**, *134* (17), 7431–7441.
[18] Ni-OH monomers: 1) Cámpora, J.; Palma, P.; del Río, D.; Álvarez, E. CO Insertion Reactions into the M−OH Bonds of Monomeric Nickel and Palladium Hydroxides. Reversible Decarbonylation of a Hydroxycarbonyl Palladium Complex. *Organometallics* **2004**, *23* (8), 1652–1655. 2) Heimann, J. E.; Bernskoetter, W. H.; Guthrie, J. A.; Hazari, N.; Mayer, J. M. Effect of Nucleophilicity on the Kinetics of CO2 Insertion into Pincer-Supported Nickel Complexes. *Organometallics* **2018**, *37* (21), 3649–3653. 3) Eberhardt, N. A.; Wellala, N. P. N.; Li, Y.; Krause, J. A.; Guan, H. Dehydrogenative Coupling of Aldehydes with Alcohols Catalyzed by a Nickel Hydride Complex. *Organometallics* **2019**, *38* (7), 1468–1478. 4) Olding, A.; Ho, C. C.; Lucas, N. T.; Canty, A. J.; Bissember, A. C. Pretransmetalation Intermediates in Suzuki–Miyaura C–C and Carbonylative Cross-Couplings: Synthesis and Structural Authentication of Aryl- and Aroylnickel(II) Boronates. *ACS Catal.* **2023**, *13* (5), 3153–3157.
[19] Ni-OH dimers: refs. 9 & 17 and 1) Carmona, E.; Marin, J. M.; Palma, P.; Paneque, M.; Poveda, M. L. Pyrrolyl, Hydroxo, and Carbonate Organometallic Derivatives of Nickel(II). Crystal and Molecular Structure of [Ni(CH$_2$C$_6$H$_4$-*o*-Me)(PMe$_3$)(μ-OH)]2•2,5-HNC$_4$H$_2$Me$_2$. *Inorg. Chem.* **1989**, *28* (10), 1895–1900. 2) So, S.-C.; Cheung, W.-M.; Sung, H. H.-Y.; Williams, I. D.; Leung, W.-H. 4-Hydroxyaryl Complexes of Group 10 Metals. *J. Organomet. Chem.* **2017**, *853*, 1–4.

boronate **15** exhibits low reactivity, potentially attributable to the competition between Ni-OH and free OH anion for boron coordination. In summary, the fast transmetalation of dimeric Ni-OH complex **16** with a boronic acid and ester, leading to the liberation of biaryl product and the regeneration of highly active monomeric Ni(0) species, suggests a plausible pathway for **16** to re-enter the catalytic cycle.

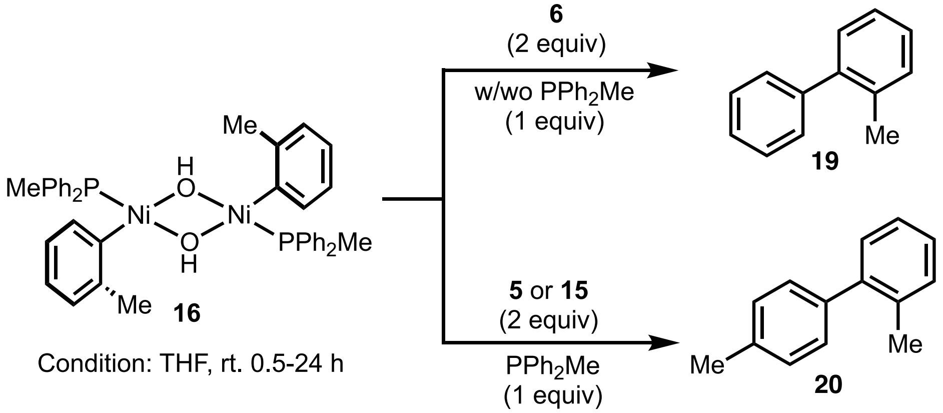

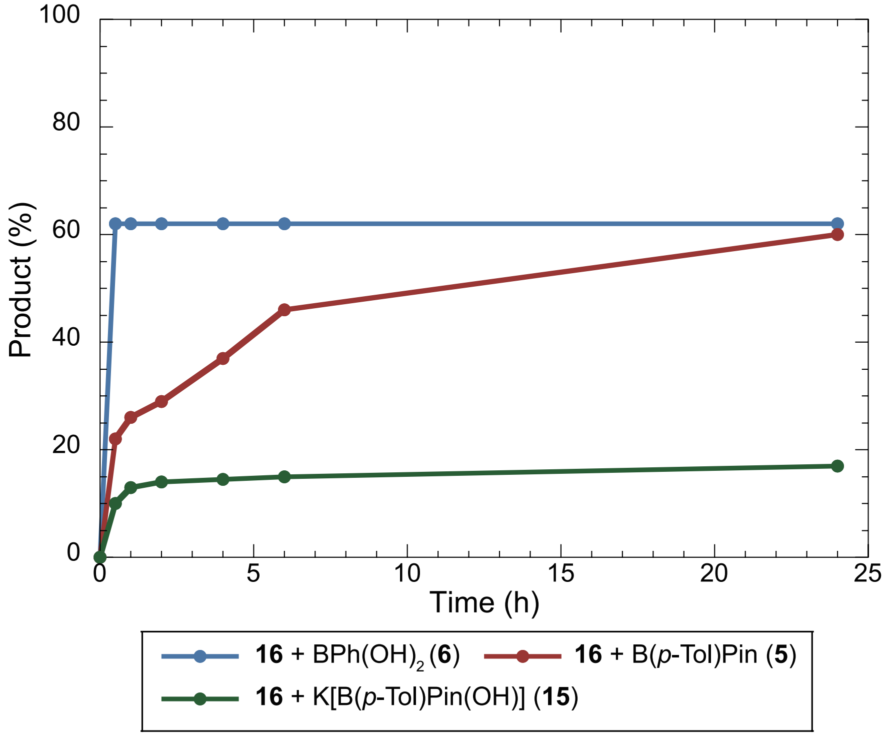

Figure S83 Stoichiometric transmetalation of complex [Ni(PPh$_2$Me)(μ-OH)(*o*-Tol)]$_2$ (**16**) with B(*p*-Tol)Pin (**5**), BPh(OH)$_2$ (**6**) and KB[(*p*-Tol)Pin(OH)] (**15**). %Yields of products **19** and **20** determined by GC.

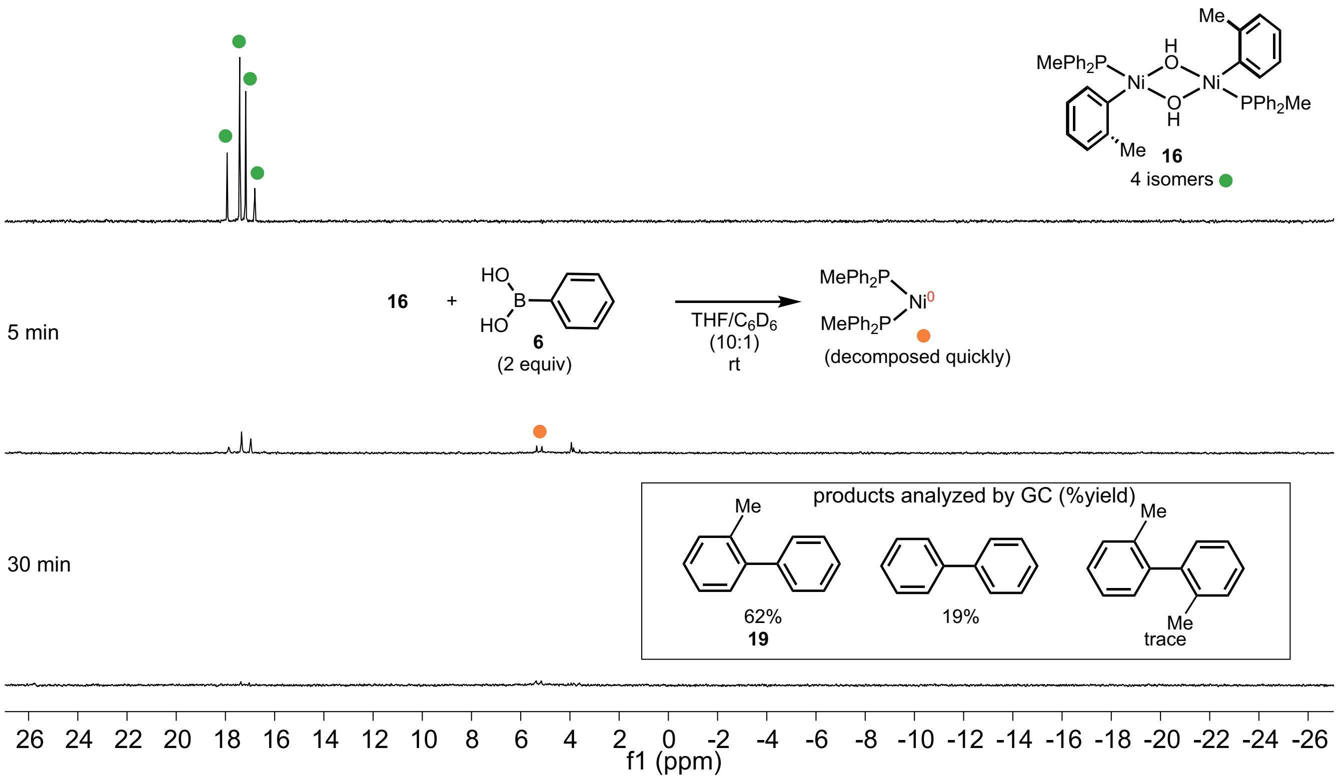

Figure S84 Representative $^{31}P\{^{1}H\}$ NMR (162.04 MHz, THF/C_6D_6) spectra of complex [Ni(PPh_2Me)(μ-OH)(*o*-Tol)]$_2$ (**16**) with $BPh(OH)_2$ (**6**) overtime, showing the formation of the Ni(0) species throughout the reaction and product distribution analyzed by GC.

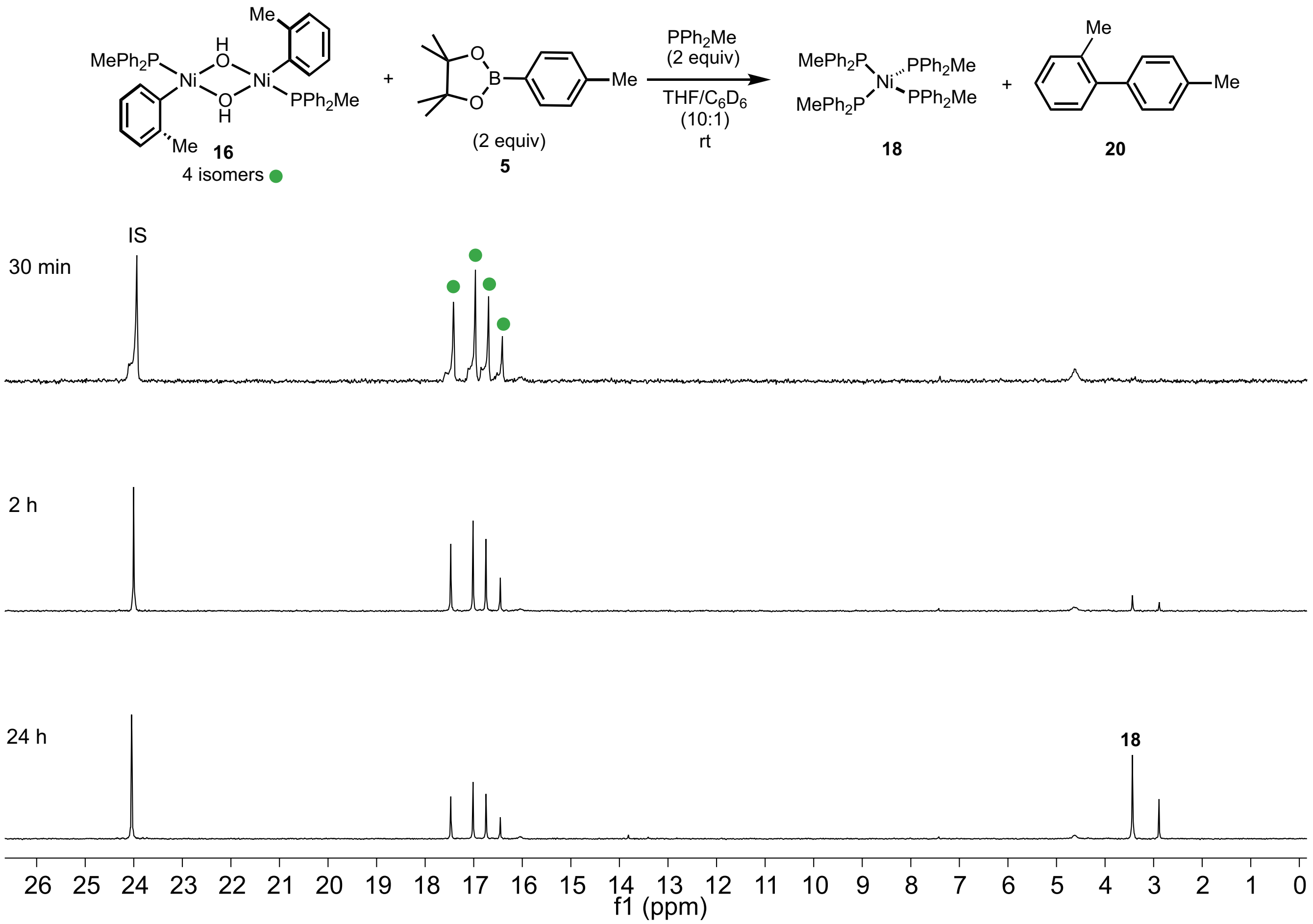

Figure S85 Representative $^{31}P\{^{1}H\}$ NMR (162.04 MHz, THF/C_6D_6) spectra of [Ni(PPh_2Me)(μ-OH)(*o*-Tol)]$_2$ (**16**) with B(*p*-Tol)Pin (**5**) overtime, showing the decrease of the Ni-OH complexes and increase of the Ni(PPh_2Me)$_4$ (**18**) throughout the reaction. Internal standard (IS): P(O)Ph_3 in a small capillary.

5.4.4 The reaction of 13 with 6 and bases

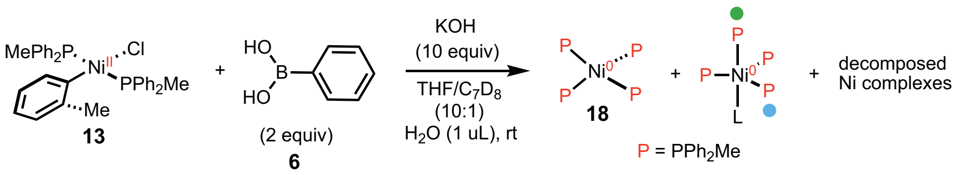

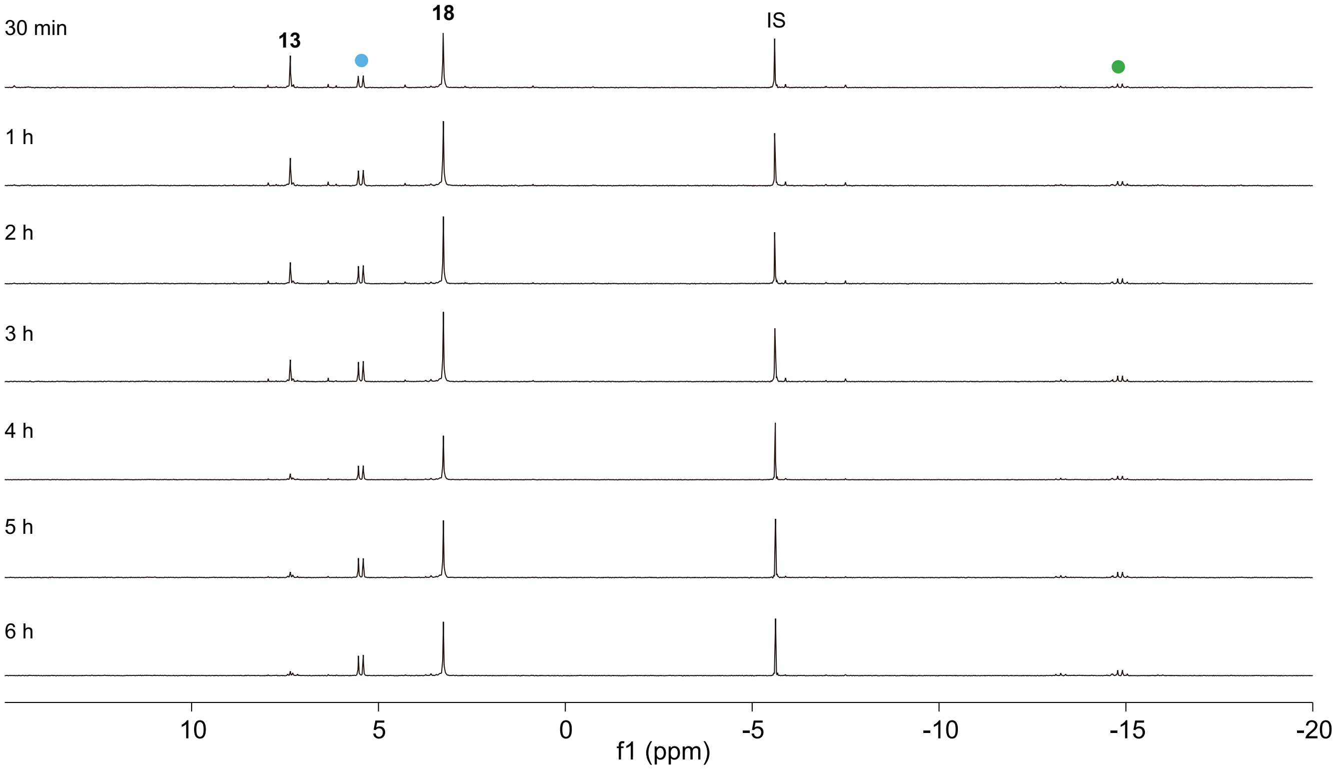

Figure S86 $^{31}P\{^1H\}$ NMR (162.04 MHz, THF/C_7D_8) spectra of Ni(PPh_2Me)$_2$Cl(*o*-Tol) (**13**) with BPh(OH)$_2$ (**6**) in the presence of excess KOH overtime. Internal standard (IS): PPh_3 in a small capillary.

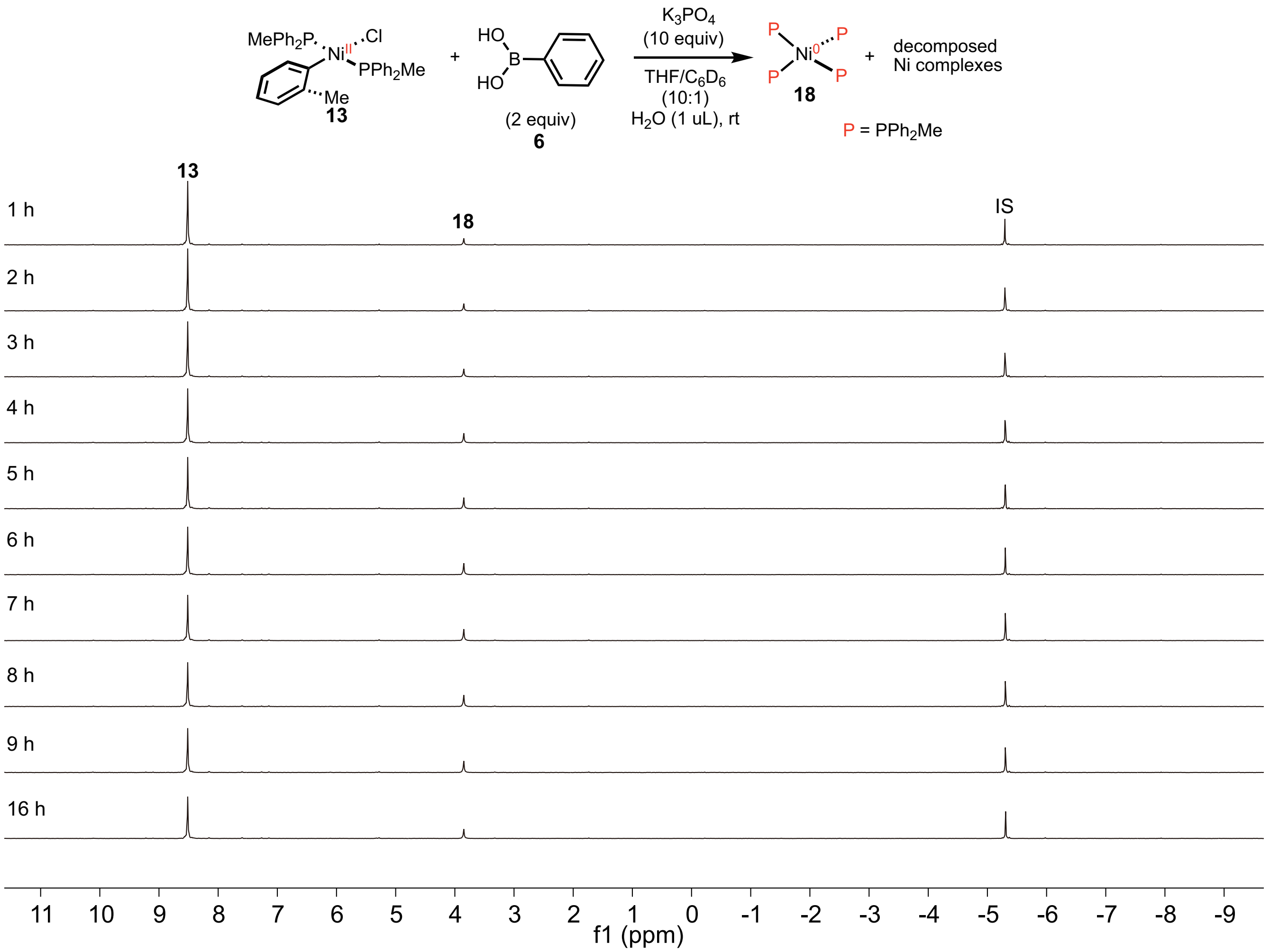

Figure S87 $^{31}P\{^{1}H\}$ NMR (162.04 MHz, THF/C_6D_6) spectra of Ni(PPh_2Me)$_2$Cl(*o*-Tol) (**13**) with BPh$(OH)_2$ (**6**) in the presence of excess K_3PO_4 overtime. Internal standard (IS): PPh_3 in a small capillary.

Scheme S3 Product Distribution of the NMR Monitoring Reactions after 24 h (Analyzed by GC).

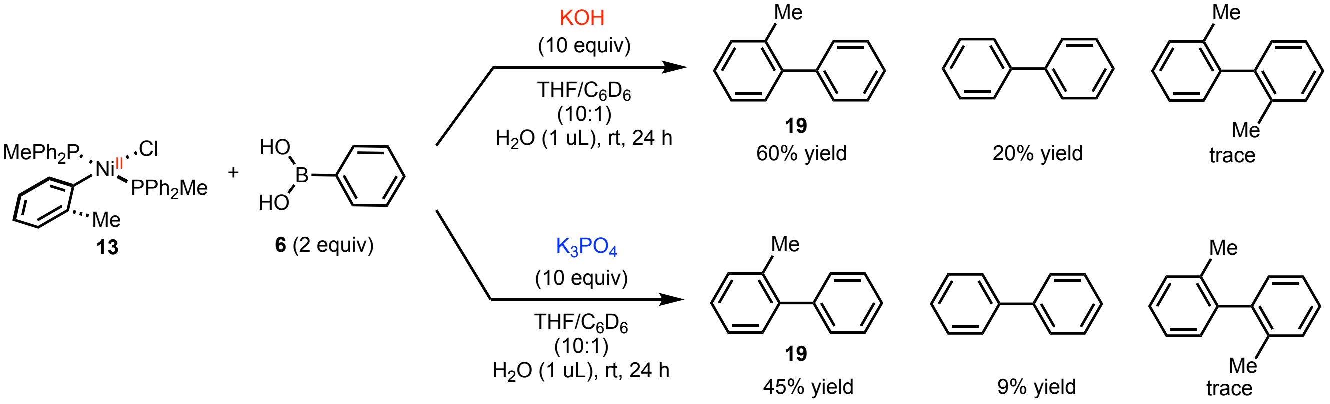

5.4.5 Reactions of complexes 13 and 16 with K[B(*p*-Tol)Pin(OH)] (15) in the presence of 18-crown-6

In the cases of complexes **13**, the reactions exhibit a slightly enhanced product conversion over 24 hours compared to reactions without 18-crown-6 (vide supra). This improvement is likely ascribed to the ability of 18-crown-6 to enhance the solubility of boronate in THF (Scheme S4). Noteworthy is the transmetalation reaction between **16** and boronate **15**, which displays the lower reactivity than that without 18-crown-6. This could be attributed to the competitive binding between Ni-OH and free OH anion for boron coordination (Scheme S4).

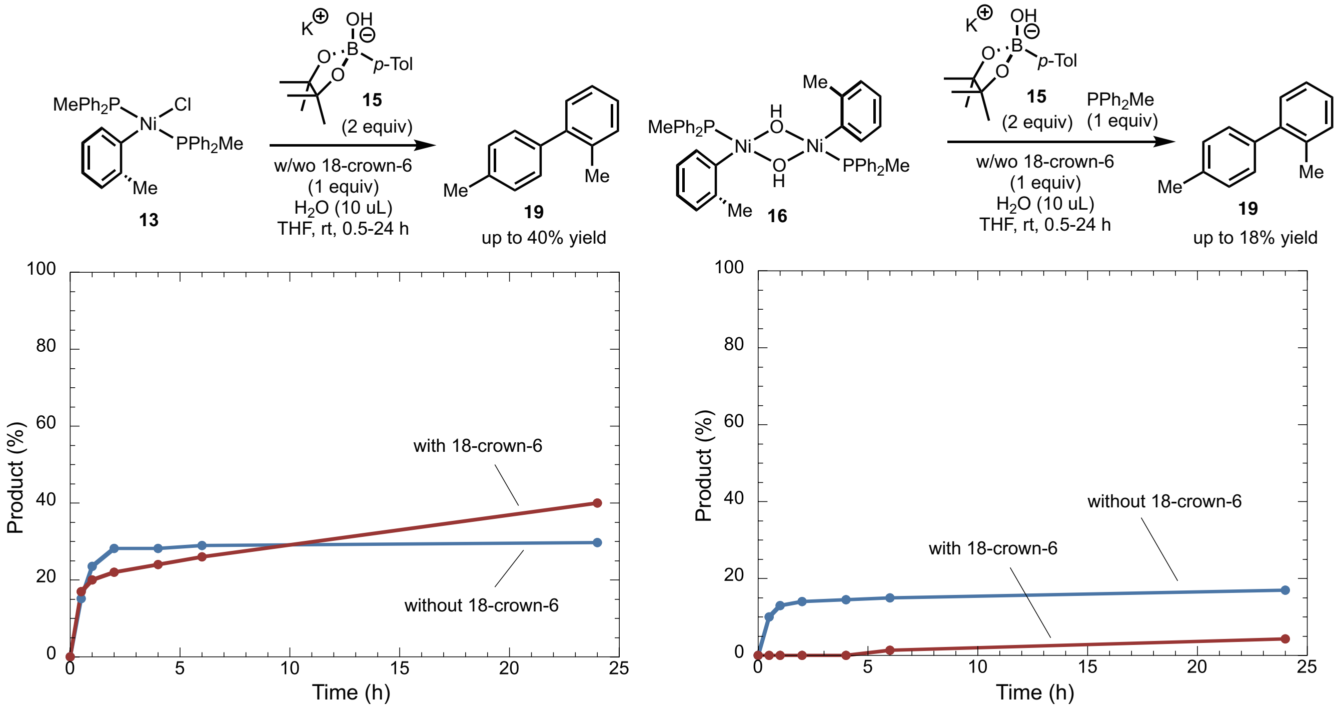

Figure S88 Stoichiometric transmetalation of complex **13**, **16** or **21** with KB[(*p*-Tol)Pin(OH)] (**15**) in the presence of 18-crown-6. %Yields of products **19** determined by GC.

Scheme S4 Proposed Mechanisms of Boronate **15** Reacting with Complexes **13** and **16**.

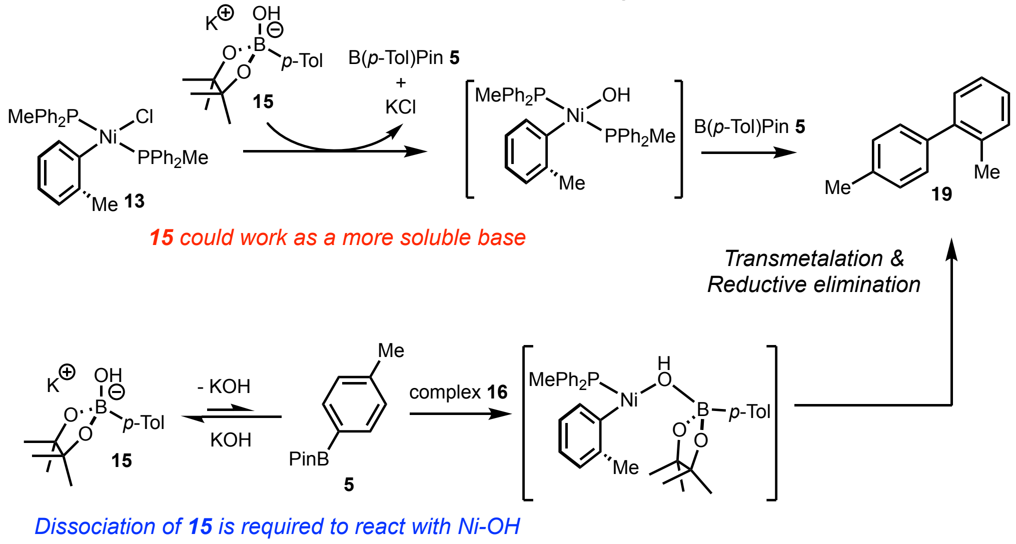

5.4.6 Comparison of rates of relevant reactions in the Ni-PPh_2Me transmetalation

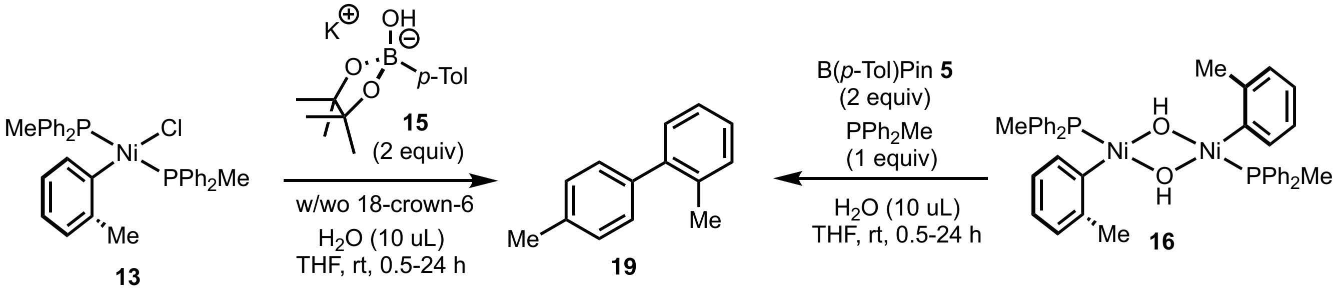

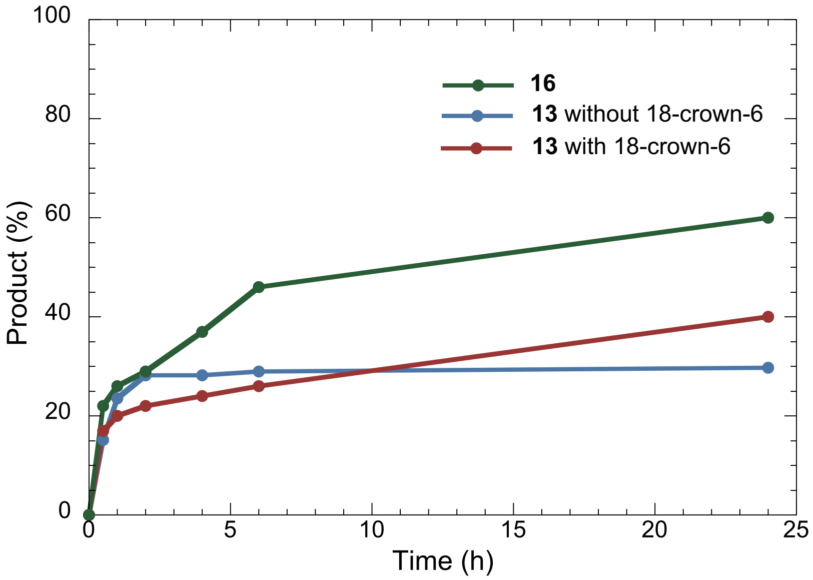

Figure S89 Comparison of stoichiometric transmetalation activity between [complex **13** + **15**] and [complex **16** and **5**]. %Yields of product **19** determined by GC.

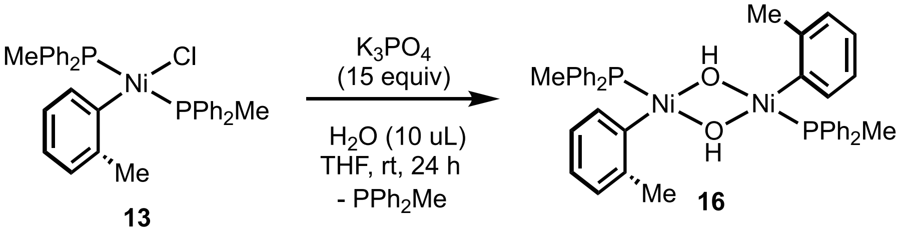

13 **16**

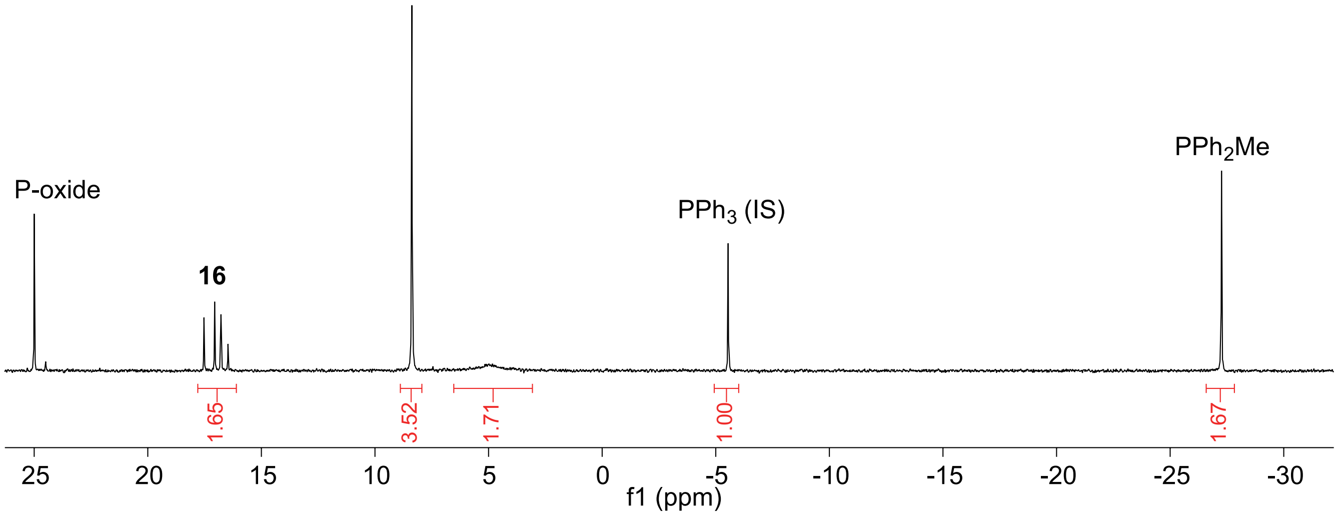

Figure S90 $^{31}P\{^{1}H\}$ NMR (162.04 MHz, THF/C_6D_6) spectra of $Ni(PPh_2Me)_2Cl$(*o*-Tol) (**13**) with excess K_3PO_4 (15 equiv) at 24 h. Internal standard (IS): PPh_3 in a small capillary. The broad signal around 5 ppm is due to unidentified species.

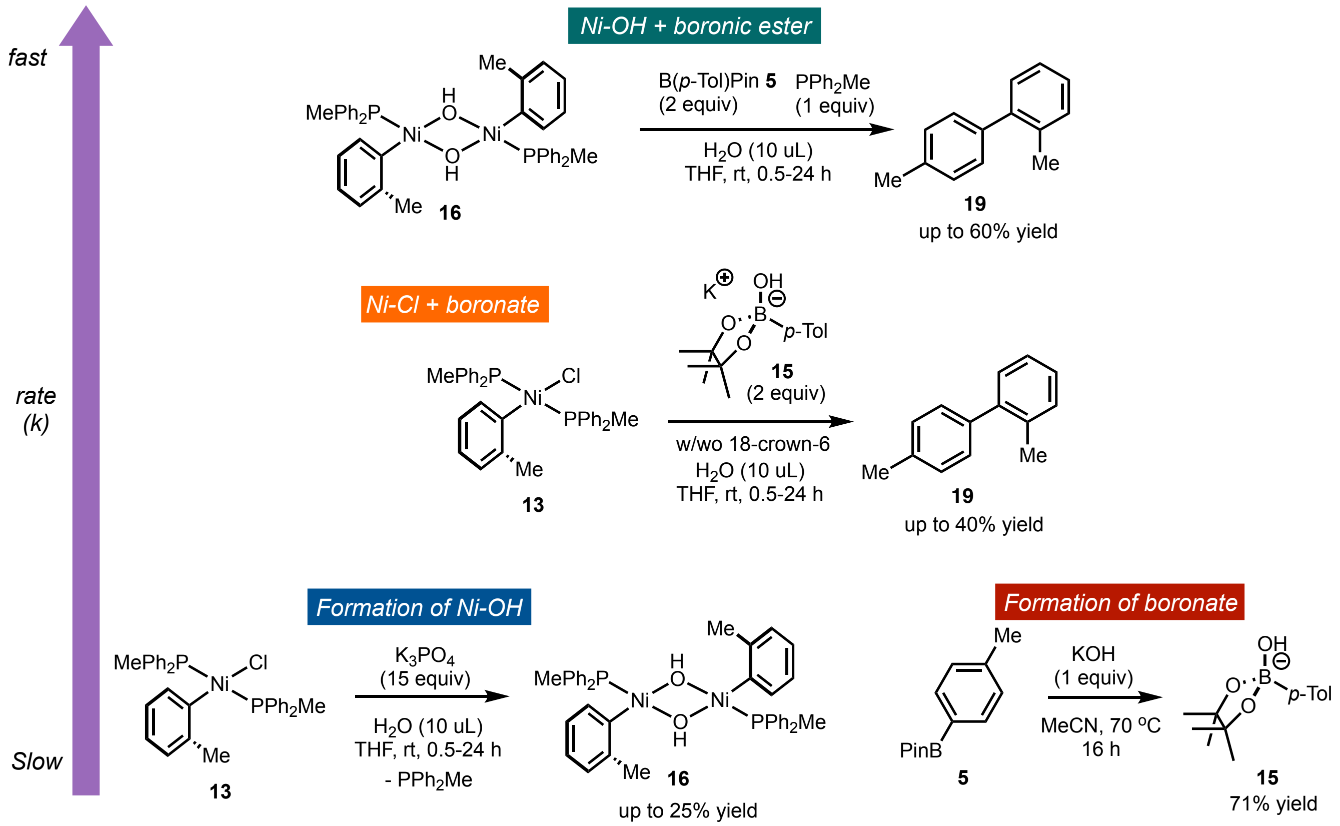

Figure S91 Relative rates of relevant reactions in the Ni-PPh_2Me transmetalation.

5.4.7 Reactions of complex Ni($PPh_2CH_2CH_2CH_2OH$)$_2$Cl(*o*-Tol) (21) with B(*p*-Tol)Pin (5), BPh$(OH)_2$ (6) and K[B(*p*-Tol)Pin(OH)] (15)

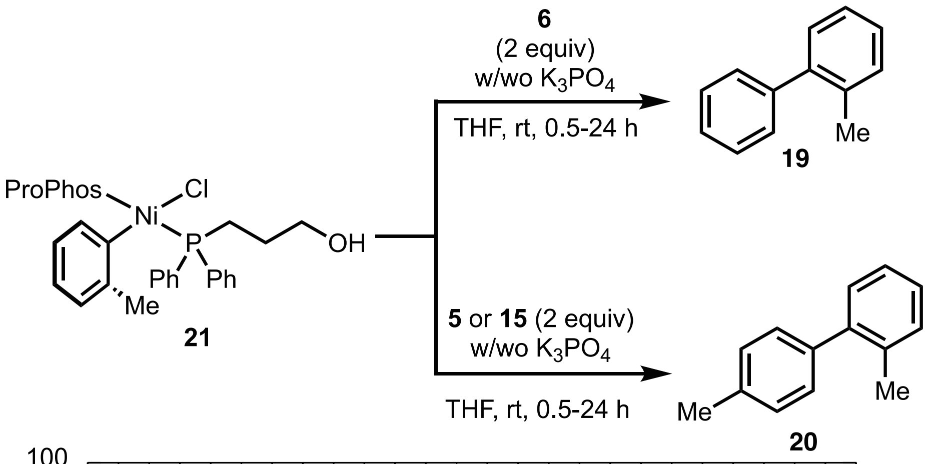

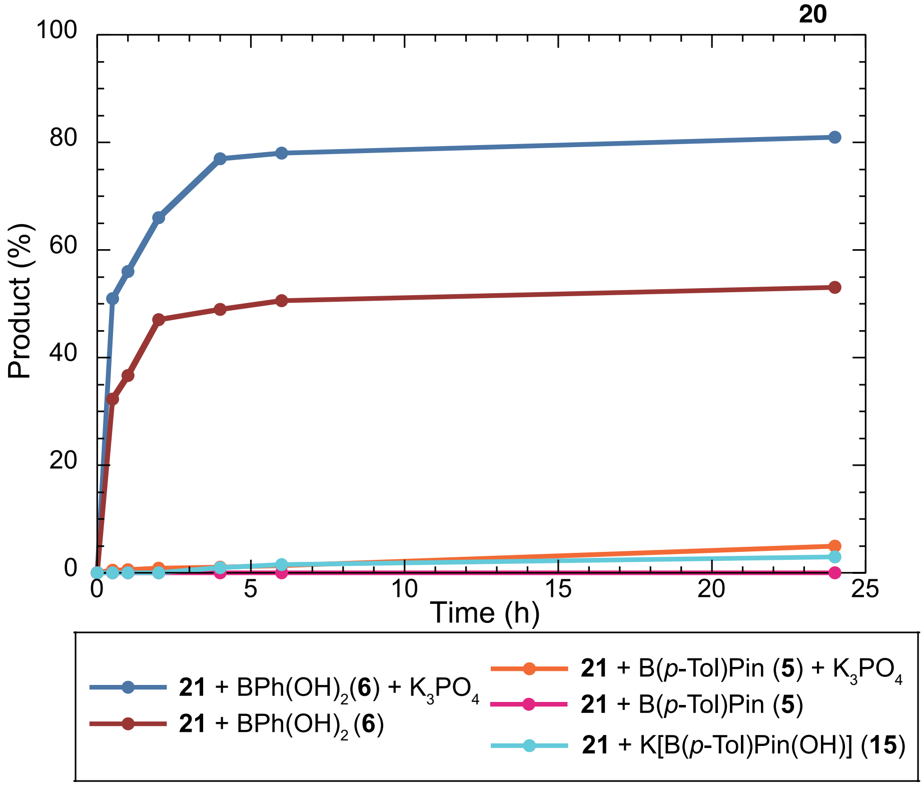

Figure S92 Stoichiometric transmetalation of complex Ni($PPh_2CH_2CH_2CH_2OH$)$_2$Cl(*o*-Tol) (**21**) with B(*p*-Tol)Pin (**5**), BPh$(OH)_2$ (**6**) and KB[(*p*-Tol)Pin(OH)] (**15**). %Yields of products **19** and **20** determined by GC.

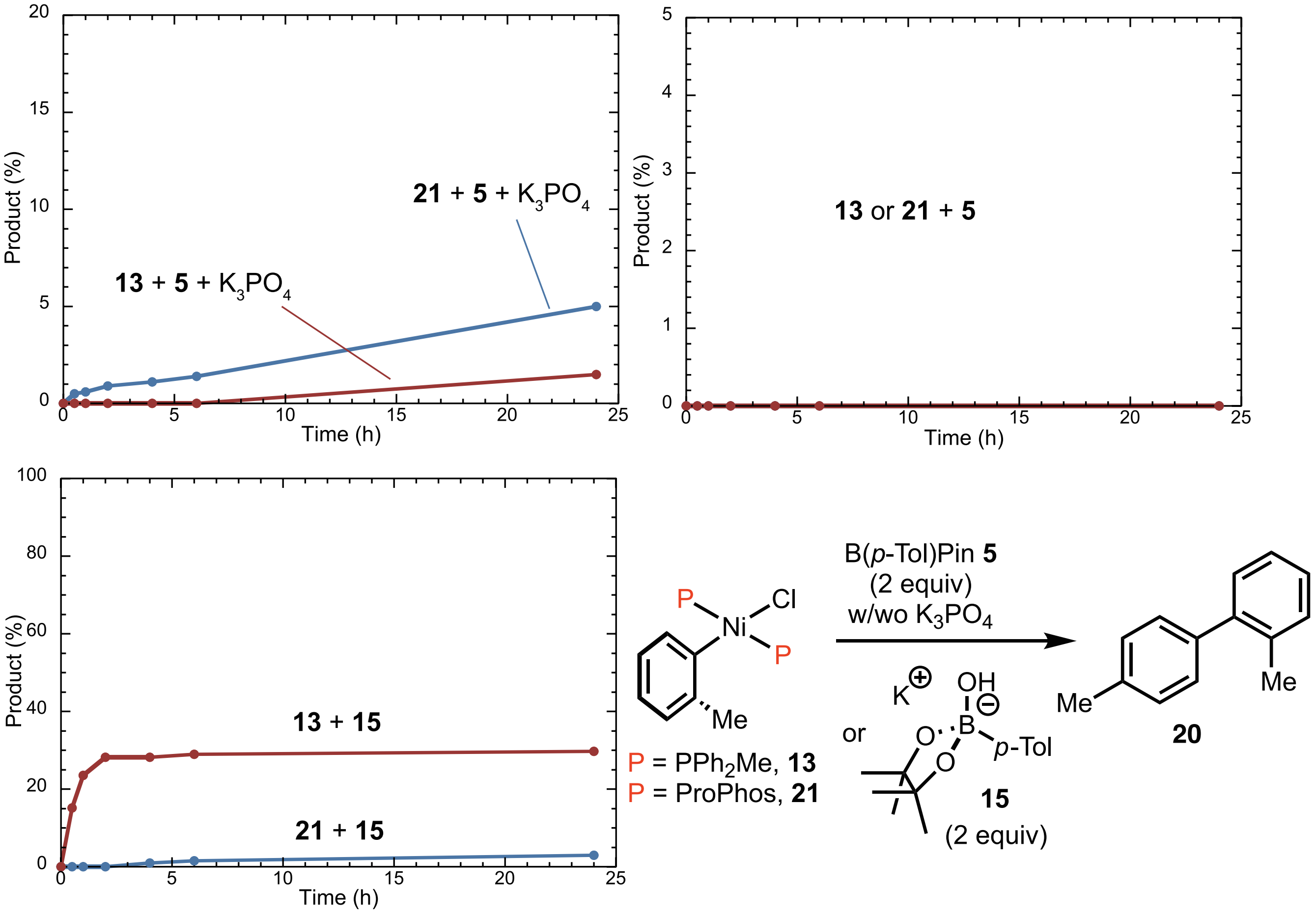

Figure S93 Comparison of stoichiometric transmetalation activity between complex **13** and **21** using **5** and **15**. %Yields of product **20** determined by GC.

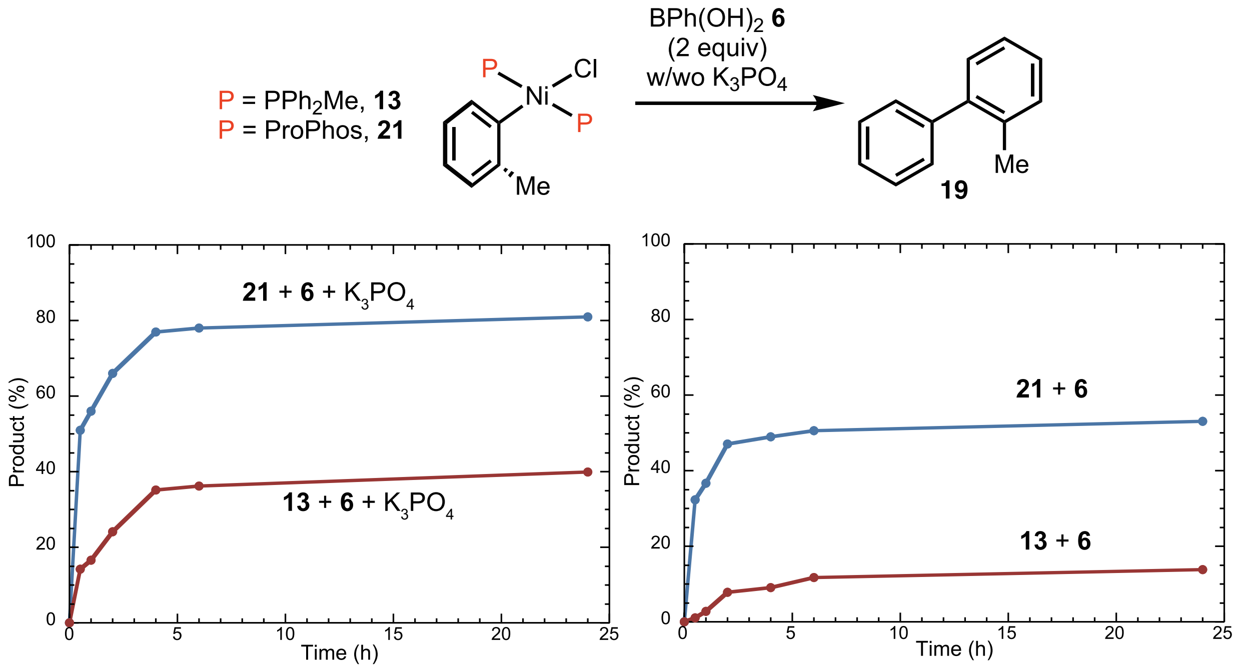

Figure S94 Comparison of stoichiometric transmetalation activity between complex **13** and complex **21** using **6**. %Yields of product **19** determined by GC.

5.4.8 The reaction of 21 with 6 and bases

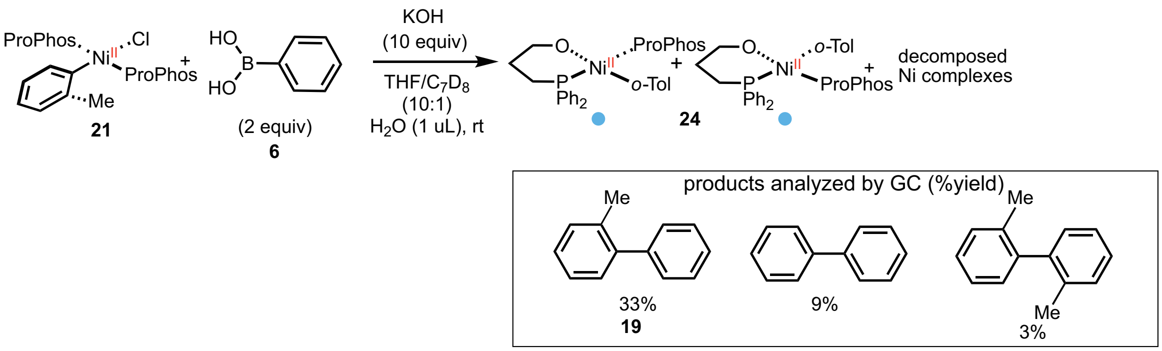

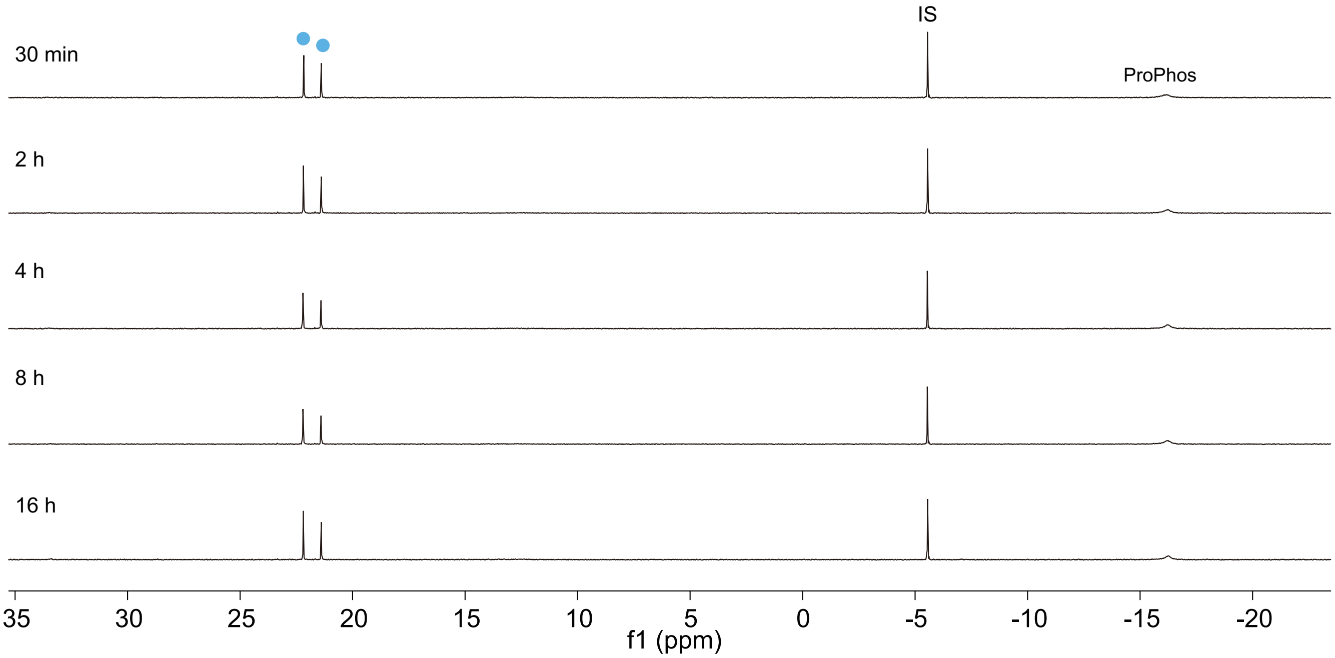

Figure S95 $^{31}P\{^1H\}$ NMR (162.04 MHz, THF/C_7D_8) spectra of complex Ni($PPh_2CH_2CH_2CH_2OH)_2Cl$(*o*-Tol) (**21**) with $BPh(OH)_2$ (**6**) in the presence of excess KOH overtime. Internal standard (IS): PPh_3 in a small capillary. Ni(κ^2-<u>P</u>$Ph_2CH_2CH_2CH_2$<u>O</u>)(*o*-Tol)(ProPhos) (**24**, •). 33% of **19** was detected by GC at 30 min and remains unchanged throughout the reaction.

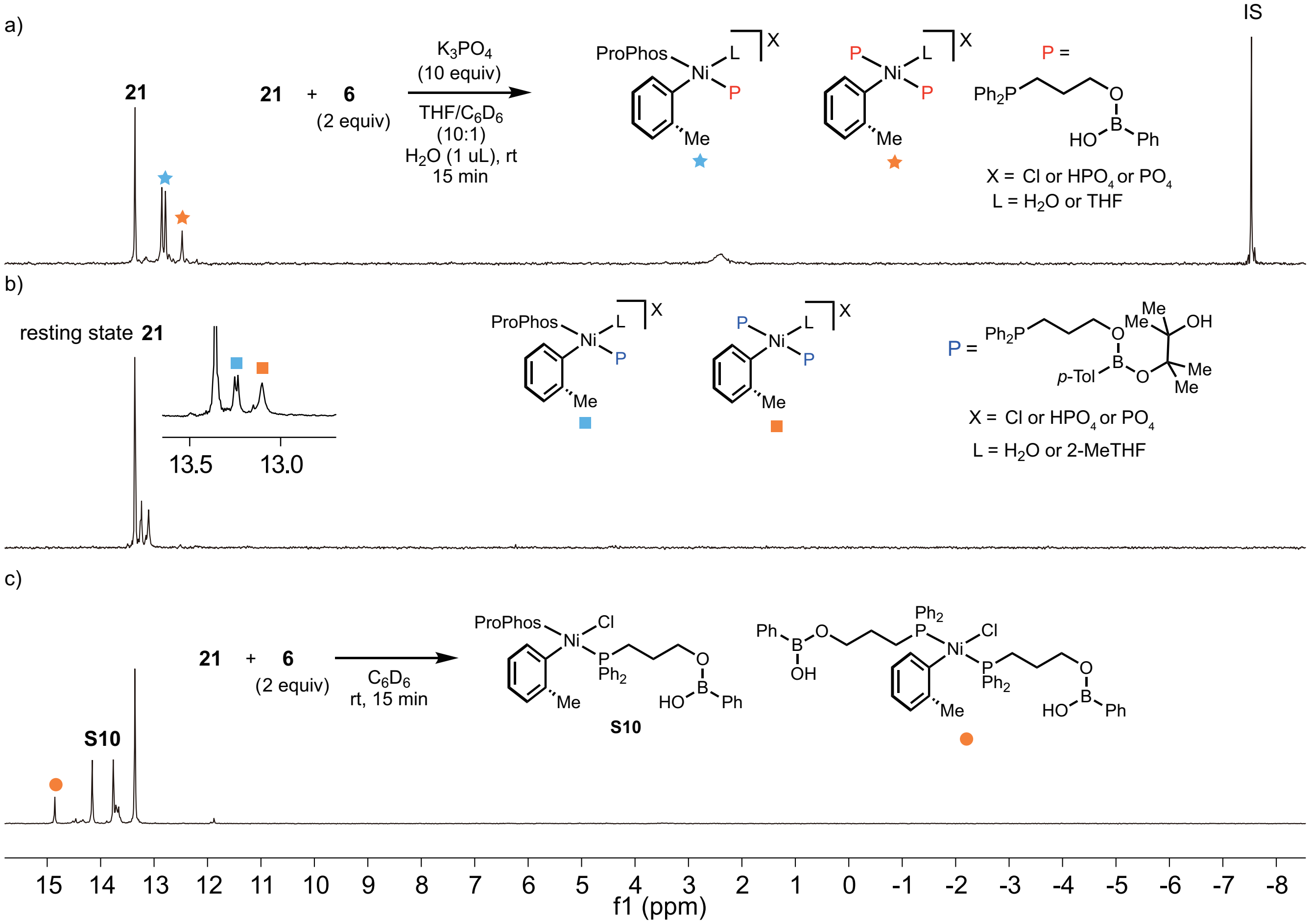

Figure S96 $^{31}P\{^1H\}$ NMR (162.04 MHz, THF/C_6D_6) spectra of: a) Ni($PPh_2CH_2CH_2CH_2OH$)$_2$Cl(*o*-Tol) (**21**) with BPh(OH)$_2$ (**6**) in the presence of excess K_3PO_4 at 15 min. Internal standard (IS): PPh_3 in a small capillary; b) the catalytic reaction mixture of 2-chlorotoluene and B(*p*-Tol)Pin (**5**) using 10 mol % Ni(cod)$_2$ and 40 mol% ProPhos after 16 h at 70 °C (2-MeTHF instead of THF); c) the reaction of complex **21** with **6** after 15 min at rt.

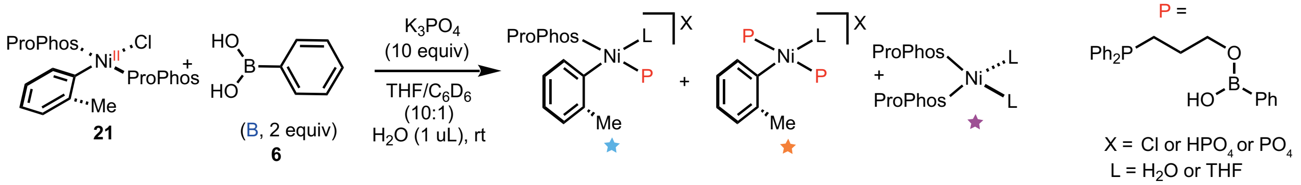

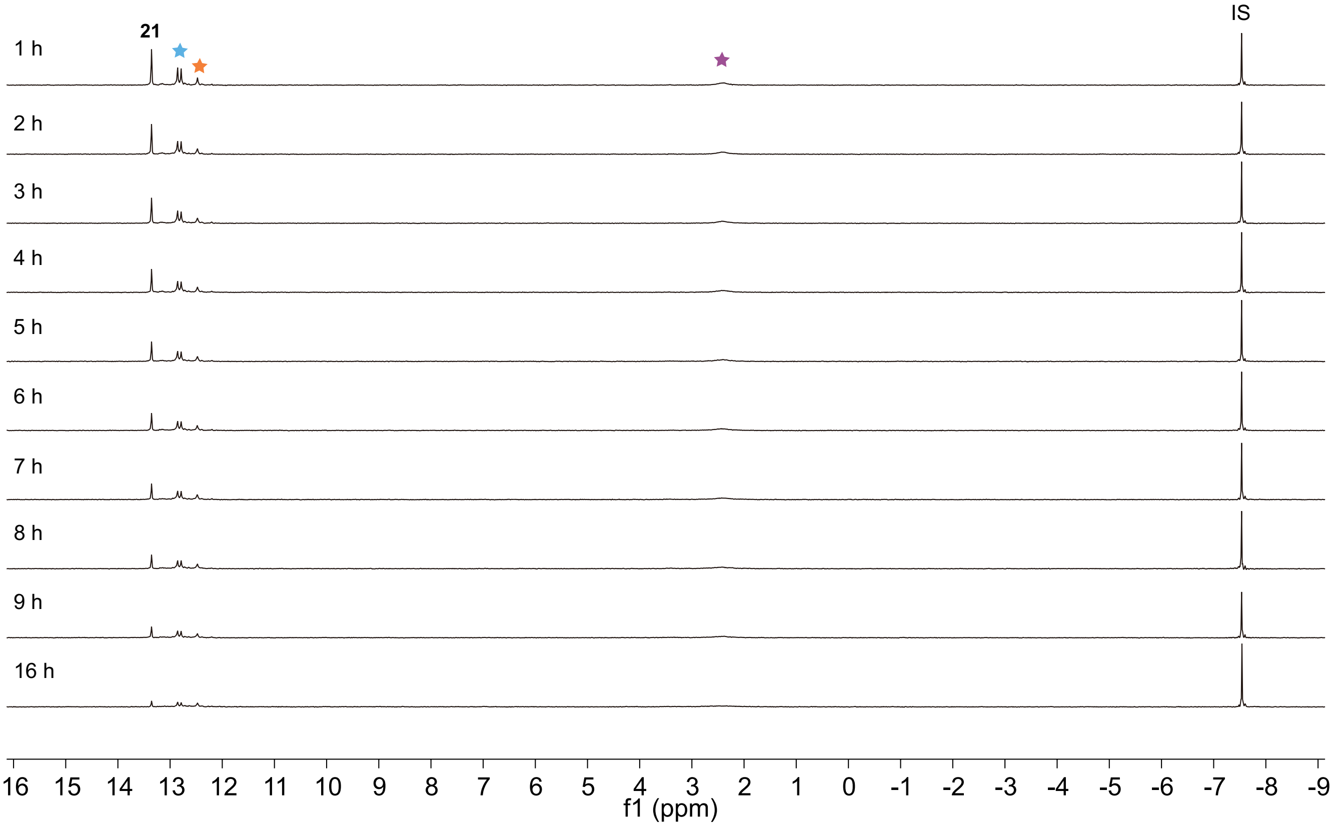

Figure S97 $^{31}P\{^{1}H\}$ NMR (162.04 MHz, THF/C_6D_6) spectra of Ni($PPh_2CH_2CH_2CH_2OH)_2$Cl(*o*-Tol) (**21**) with $BPh(OH)_2$ (**6**) in the presence of excess K_3PO_4 overtime. Internal standard (IS): PPh_3 in a small capillary. The broad ^{31}P signal at ~3 ppm is tentatively assigned to Ni($PPh_2CH_2CH_2CH_2OH)_2(L)_2$.

Scheme S5 Product Distribution Analyzed by GC.

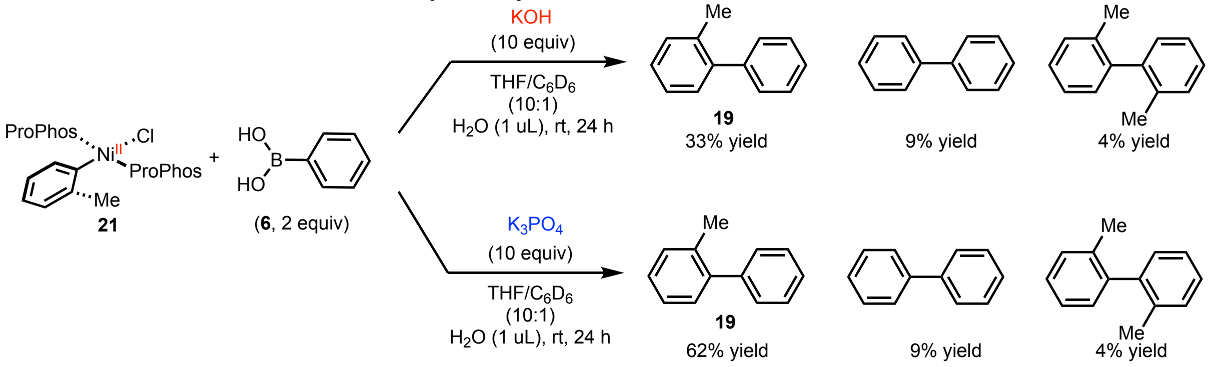

5.4.9 Role of the base in the Ni-ProPhos catalytic system

Addition of $BPh(OH)_2$ (**6**, 4.9 mg, 0.040 mmol, 2.0 equiv) to the isolated $PPh_2CH_2CH_2CH_2OH$ (**9**, 5.0 mg, 0.020 mmol, 1.0 equiv) in C_6D_6 leads to an immediate formation of the phosphine-borane adduct $PPh_2CH_2CH_2CH_2OB(OH)Ph$, as detected by ^1H (Figure S98) and $^{31}P\{^1H\}$ NMR (Figure S99b). The result is similar to our observation in the analogous reaction using **9** and **22** (vide supra). We added KOH (2.3 mg, 0.040 mmol, 2.0 equiv) along with a trace amount H_2O (1 μL) into this reaction mixture, resulting in a rapid regeneration of $PPh_2CH_2CH_2CH_2OH$ in situ as detected by ^1H and $^{31}P\{^1H\}$ NMR (Figure S99c). These results indicate that deboronation of the phosphine-borane adduct by base is feasible and rapid. This OH regeneration in the ligand will be a fast and important step in this catalytic system.

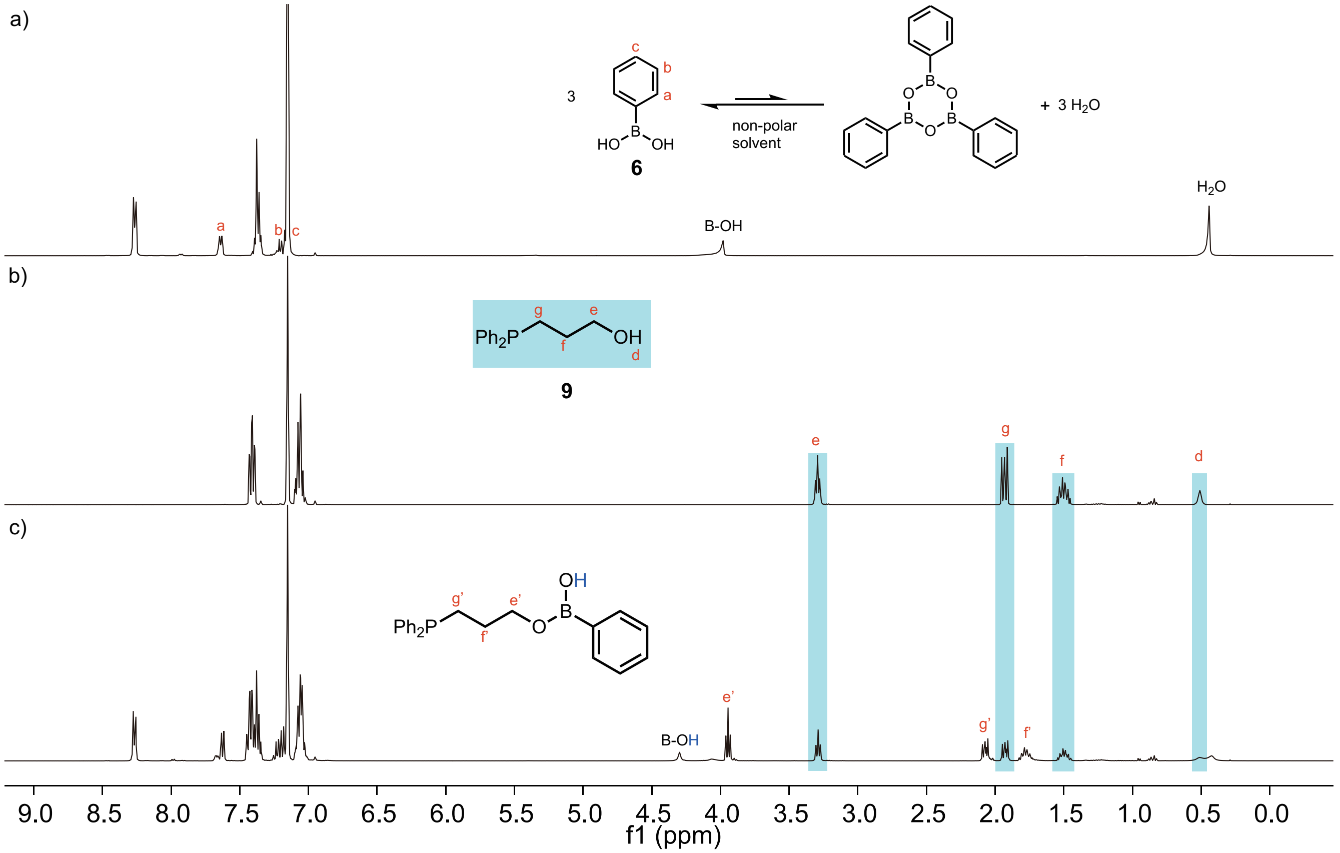

Figure S98 ^1H NMR (400.30 MHz, C_6D_6) spectra of: a) $BPh(OH)_2$ (**6**); b) isolated ProPhos **9**; c) the reaction of **9** with **6** (2 equiv) at rt.

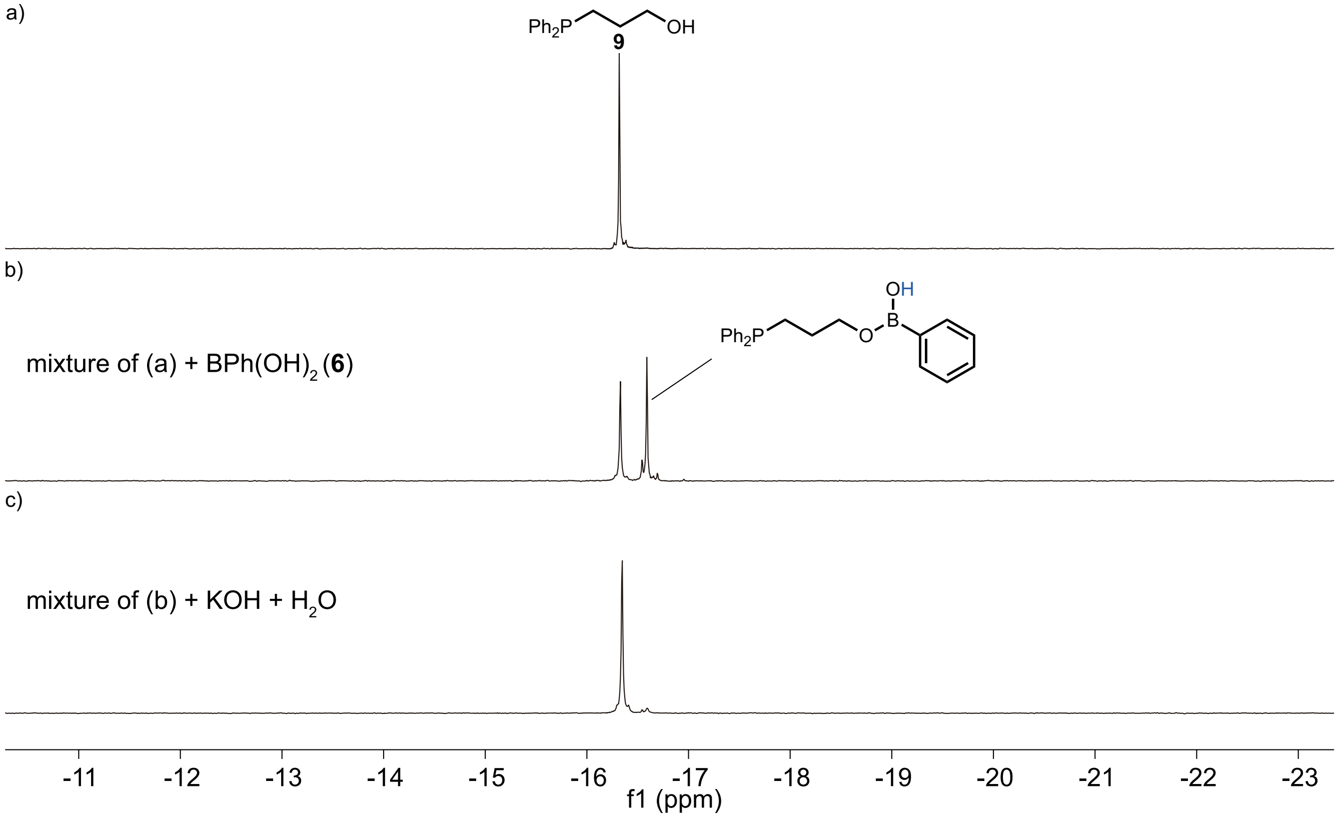

Figure S99 $^{31}P\{^1H\}$ NMR (162.04 MHz, C_6D_6) spectra of: a) isolated ProPhos **9**; b) the reaction of ProPhos **9** with **6** (2 equiv); c) the reaction mixture from (b) reacts with KOH (2.0 equiv) and H_2O (1 μL).

Intrigued by the remarkable transmetalation activity observed in the absence of a base, we embarked on a catalytic investigation involving a "base-free" reaction. Specifically, we explored the cross-coupling reaction between 4-bromoacetophenone **4** and phenylboronic acid **6**, employing our well-established kinetic experimental approach with a 0.5 mol% Ni loading. Notably, this reaction yielded an only 1% of the desired cross-coupled product (corresponding to a turnover number, TON, of 2). This result implies that a single phosphine ligand attached to the nickel center facilitated one transmetallation event, with the catalyst resting state featuring two phosphine ligands. Collectively, our studies revealed that while the Ni-ProPhos complex demonstrated significant stoichiometric "base-free" transmetalation reactivity, the presence of a base proved imperative to achieve catalytic transmetalation.

6. Substrate study using ProPhos 9

6.1 General procedure for determining the yield of the biaryl compounds

2-MeTHF/H_2O system

A vial was charged with Ni(cod)$_2$ (0.0002–0.006 mmol, 0.10–3.0 mol%), 2-MeTHF (4 mL) and $PPh_2CH_2CH_2CH_2OH$ (**9**, 0.0008–0.024 mmol, 0.40–12.0 mol%) and the resulting deep red stock solution was stirred for 0.5 h. This solution (0.4 mL) was transferred to a vial containing the aryl boronic ester or acid (0.22–0.30 mmol), aryl halide (if solid, 0.20 mmol), and K_3PO_4 (0.50 mmol). After that, aryl halide (if liquid, 0.20 mmol) and H_2O (80 μL) were added. The reaction was placed in a shaker, the agitation speed was set to 800 rpm, and heated to 70 °C for 16 h, then cooled to rt. The *n*-decane (internal standard, 0.1 mmol) was added to the reaction mixture. Then, the reaction mixture was diluted with EtOAc (10 mL) and washed with H_2O (2 x 5 mL). The combined aqueous layers were extracted with EtOAc (5 mL). An aliquot of the combined organic layer was analyzed by gas chromatography to obtain the GC yield (R_f values for each compounds determined, *vide infra*). The combined organic layers were concentrated in vacuo and the crude product was purified by flash chromatography (hexane:EtOAc).

i-PrOH system

A vial was charged with $NiCl_2$•$6H_2O$ (0.002–0.006 mmol, 1.0–3.0 mol%), *i*-PrOH (4 mL) and $PPh_2CH_2CH_2CH_2OH$ (**9**, 0.008–0.024 mmol, 4.0–12.0 mol %) and the resulting deep red stock solution was stirred for overnight *(Note: solubility of the Ni(II) precursor is poor in the i-PrOH. For expedited procedures, direct weighing of Ni and ligand precursors is feasible for 3 mol% catalysis. However, the stock solution is required for the 1 mol% catalysis.*) This solution (0.4 mL) was transferred to a vial containing the aryl boronic acid (0.24–0.30 mmol), aryl halide (if solid, 0.20 mmol), and K_3PO_4 (0.50 mmol). After that, aryl halide (if liquid, 0.20 mmol) was added. The reaction was placed in a shaker, the agitation speed was set to 800 rpm, and heated to 70 °C for 16 h, then cooled to rt. The *n*-decane (internal standard, 0.1 mmol) was added to the reaction mixture. Then, the reaction mixture was diluted with EtOAc (10 mL) and washed with H_2O (2 x 5 mL). The combined aqueous layers were extracted with EtOAc (5 mL). An aliquot of the combined organic layer was analyzed by gas chromatography to obtain the GC yield (R_f values for each compounds determined, *vide infra*). The combined organic layers were concentrated in vacuo and the crude product was purified by flash chromatography (hexane:EtOAc).

Air-stable complex $Ni(PPh_2CH_2CH_2CH_2OH)_2Cl$(*o*-Tol) (**21**, 0.006 mmol, 3.0 mol%) can also used as a precatalyst without adding **9** and a reducing reagent in *i*-PrOH. The results are presented in Scheme S7.

Scheme S6. GC Response Factor (R_f, navy) of Products Against *n*-Decane as the Internal Standard, Determined by Calibration Curves Using Corresponding Isolated Compounds.

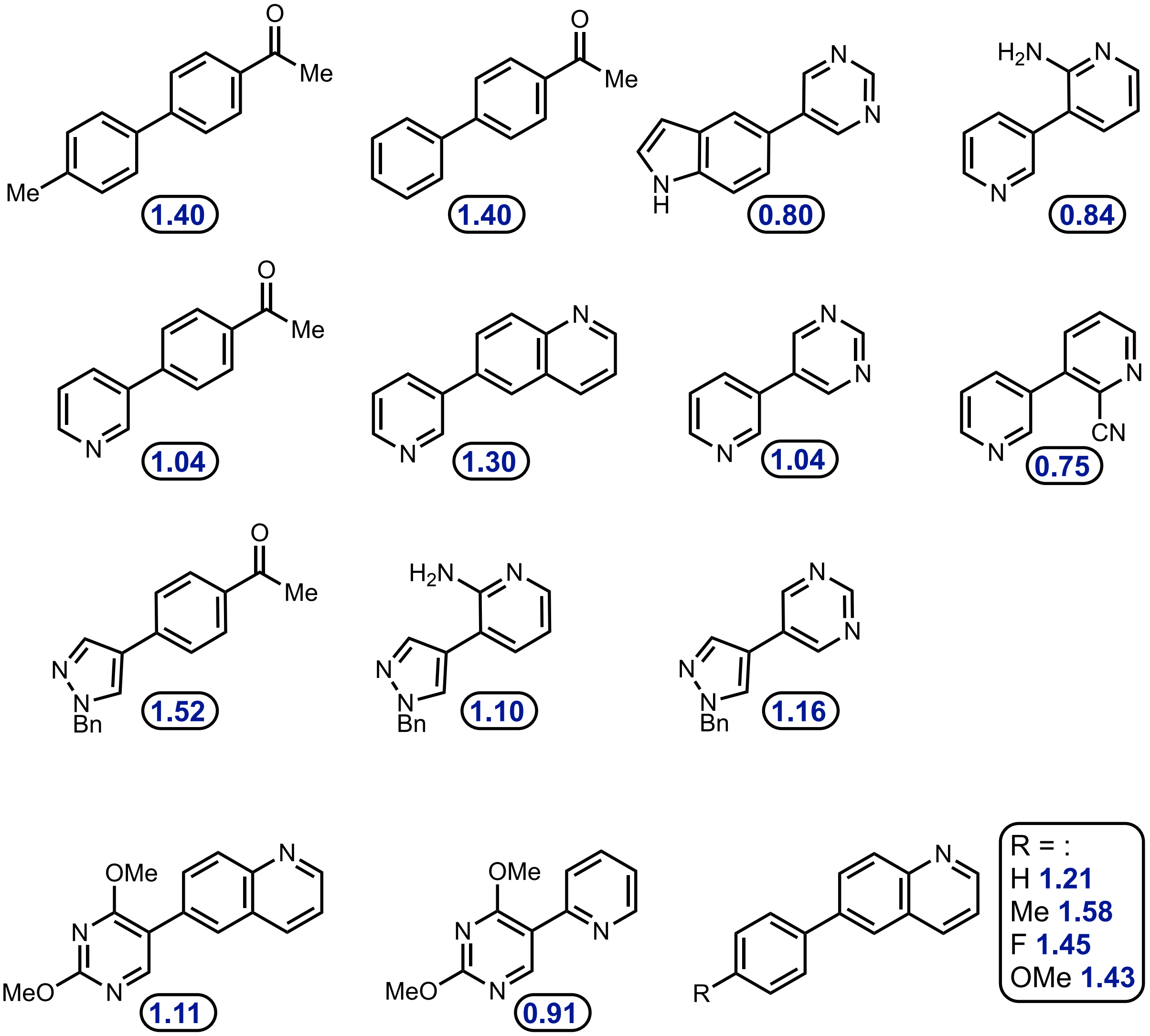

Scheme S7 SMC Catalyzed by Ni(ProPhos) Precatalyst **21**.

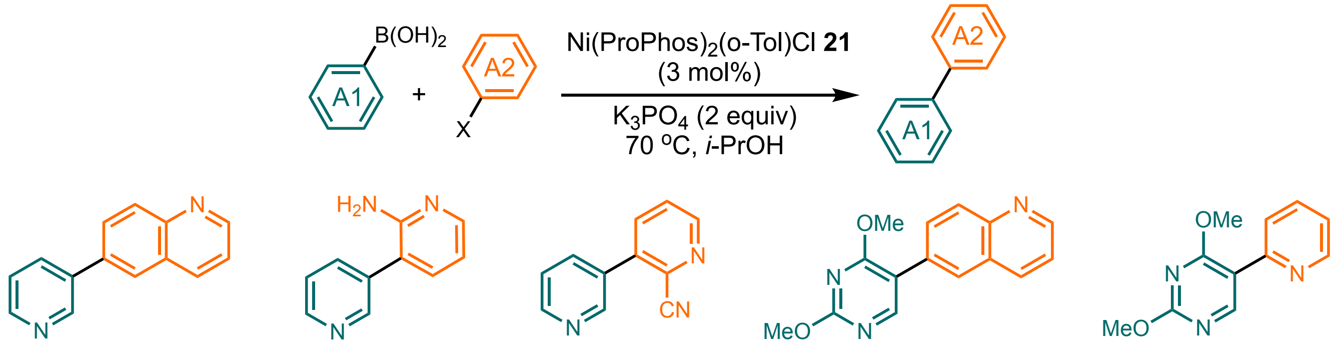

**32**	**41**	**42**	**43**	**44**
84% yield	52% yield	82% yield	95% yield	94% yield

6.2 Characterization data for Ni-SMC products

1-[4-(4-Tolyl)phenyl]ethenone[20] (7)

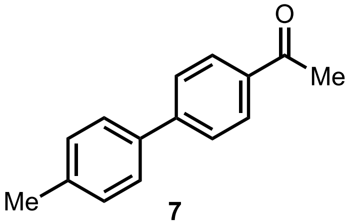

7

Multiple scales (0.2–1 mmol) were conducted for synthesizing this compound. For example, 4-bromophenylethanone (40 mg, 0.20 mmol, 1.0 equiv) and 4,4,5,5-tetramethyl-2-(*p*-tolyl)-1,3,2-dioxaborolane (48 mg, 0.22 mmol, 1.1 equiv) were used to give a white solid (41 mg, 0.19 mmol, 97%).

^{1}H NMR (400.30 MHz, $CDCl_3$) δ: 8.06 – 7.99 (m, 2H), 7.71 – 7.65 (m, 2H), 7.57 – 7.51 (m, 2H), 7.31 – 7.27 (m, 2H), 2.64 (s, 3H, $COCH_3$), 2.41 (s, 3H, CH_3).

$^{13}C\{^1H\}$ NMR (100.67 MHz, $CDCl_3$) δ: 197.9 (s, C=O), 145.9 (s), 138.4 (s), 137.1 (s), 135.8 (s), 129.8 (s), 129.1 (s), 127.3 (s), 127.1 (s), 26.8 (s, $COCH_3$), 21.3 (s, CH_3).

MS (ESI-TOF, CH_3CN) m/z: $[M + H]^+$ calculated for $C_{15}H_{15}O$ 211.11, found 211.11.

1-(4-Phenylphenyl)ethenone[21](8)

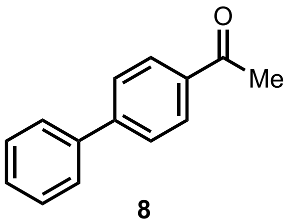

8

Multiple scales (0.2–1 mmol) were conducted for synthesizing this compound. For an example using 0.1 mol% Ni, 4-bromophenylethanone (40 mg, 0.20 mmol, 1.0 equiv) and phenylboronic acid (37 mg, 0.30 mmol, 1.5 equiv) were used. The reaction was repeated 5 times. The combined mixture gave a white solid (180 mg, 0.93 mmol, 93%).

^{1}H NMR (400.30 MHz, $CDCl_3$) δ:8.07 – 7.99 (m, 2H), 7.73 – 7.66 (m, 2H), 7.65 – 7.60 (m, 2H), 7.48 (tt, *J* = 6.7, 0.9 Hz, 2H), 7.43 – 7.38 (m, 1H), 2.64 (s, 3H, CH_3).

$^{13}C\{^1H\}$ NMR (100.67 MHz, $CDCl_3$) δ: 197.9 (s, C=O), 145.9 (s), 140.0 (s), 136.0 (s), 129.1 (s), 129.1 (s), 128.4 (s), 127.4 (s), 127.4 (s), 26.8 (s, CH_3).

MS (ESI-TOF, CH_3CN) m/z: $[M + Na]^+$ calculated for $C_{15}H_{11}NaO$ 218.07, found 218.07.

---

[20] Peter, C.; Derible, A.; Parmentier, J.; Le Drian, C.; Becht, J.-M. A Green Direct Preparation of a Magnetic Ordered Mesoporous Carbon Catalyst Containing Fe–Pd Alloys: Application to Suzuki–Miyaura Reactions in Propane-1,2-Diol. *New J. Chem.* **2017**, *41* (12), 4931–4936.

[21] Wang, P.; Liu, H.; Niu, J.; Li, R.; Ma, J. Entangled Pd Complexes over $Fe_3O_4@SiO_2$ as Supported Catalysts for Hydrogenation and Suzuki Reactions. *Catal. Sci. Technol.* **2014**, *4* (5), 1333–1339.

6-(4-Methylphenyl)quinoline[22] (30)

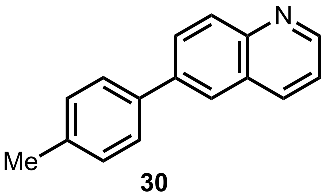

30

6-Chloroquinoline (33 mg, 0.20 mmol, 1.0 equiv) and 4,4,5,5-tetramethyl-2-(*p*-tolyl)-1,3,2-dioxaborolane (52 mg, 0.24 mmol, 1.2 equiv) were used to give a white solid (41 mg, 0.19 mmol, 93%).

^{1}H NMR (400.30 MHz, $CDCl_3$) δ: 8.91 (dd, *J* = 4.3, 1.7 Hz, 1H), 8.23 – 8.13 (m, 2H), 8.01 – 7.94 (m, 2H), 7.65 – 7.61 (m, 2H), 7.42 (dd, *J* = 8.3, 4.2 Hz, 1H), 7.35 – 7.29 (m, 2H), 2.43 (s, 3H, CH_3).

$^{13}C\{^1H\}$ NMR (100.67 MHz, $CDCl_3$) δ: 150.2 (s), 147.6 (s), 139.3 (s), 137.7 (s), 137.4 (s), 136.2 (s), 129.8 (s), 129.7 (s), 129.2 (s), 128.5 (s), 127.3 (s), 125.1 (s), 121.4 (s), 21.2 (s, CH_3).

MS (ESI-TOF, CH_3CN) m/z: $[M + K]^+$ calculated for $C_{16}H_{13}NK$ 258.06, found 258.06.

1-[4-(3-Pyridinyl)phenyl]ethanone[23] (31)

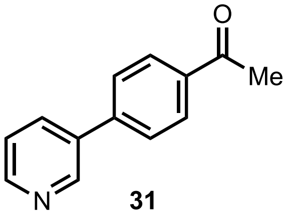

31

4-Bromophenylethanone (40 mg, 0.20 mmol, 1.0 equiv) and 3-(4,4,5,5-tetramethyl-1,3,2-dioxaborolan-2-yl)pyridine (49 mg, 0.24 mmol, 1.2 equiv) were used to give a white solid (37 mg, 0.19 mmol, 94%).

^{1}H NMR (400.30 MHz, $CDCl_3$) δ: 8.89 (dd, *J* = 2.4, 0.9 Hz, 1H), 8.65 (dd, *J* = 4.8, 1.6 Hz, 1H), 8.12 – 8.03 (m, 2H), 7.92 (ddd, *J* = 7.9, 2.4, 1.6 Hz, 1H), 7.75 – 7.63 (m, 2H), 7.41 (ddd, *J* = 7.9, 4.8, 0.8 Hz, 1H), 2.65 (s, 3H, CH_3).

$^{13}C\{^1H\}$ NMR (100.67 MHz, $CDCl_3$) δ: 197.7 (s, C=O), 149.5 (s), 148.5 (s), 142.5 (s), 136.7 (s), 135.6 (s), 134.6 (s), 129.3 (s), 127.5 (s), 123.8 (s), 26.9 (s, CH_3).

MS (ESI-TOF, CH_3OH) m/z: $[M + H]^+$ calculated for $C_{13}H_{12}NO$ 198.09, found 198.09.

---

[22] Wilson, K. L.; Murray, J.; Jamieson, C.; Watson, A. J. B. Cyrene as a Bio-Based Solvent for the Suzuki–Miyaura Cross-Coupling. *Synlett* **2018**, *29* (05), 650–654.

[23] Chen, G.-J.; Huang, J.; Gao, L.-X.; Han, F.-S. Nickel-Catalyzed Cross-Coupling of Phenols and Arylboronic Acids Through an In Situ Phenol Activation Mediated by PyBroP. *Chem. – A Eur. J.* **2011**, *17* (14), 4038–4042.

6-(Pyridin-3-yl)quinoline[8] (32)

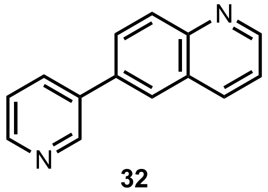

32

6-Chloroquinoline (33 mg, 0.20 mmol, 1.0 equiv) and 3-(4,4,5,5-tetramethyl-1,3,2-dioxaborolan-2-yl)pyridine (49 mg, 0.24 mmol, 1.2 equiv) were used to give a white solid (35 mg, 0.18 mmol, 85%).

^{1}H NMR (400.30 MHz, $(CD_3)_2S{=}O$) δ: 9.10 – 9.04 (m, 1H), 8.94 (dt, *J* = 4.5, 2.1 Hz, 1H), 8.63 (dt, *J* = 5.0, 1.3 Hz, 1H), 8.48 – 8.42 (m, 1H), 8.39 (d, *J* = 4.0 Hz, 1H), 8.26 (ddd, *J* = 8.3, 3.5, 2.0 Hz, 1H), 8.15 (p, *J* = 2.0 Hz, 2H), 7.66 – 7.51 (m, 2H).

$^{13}C\{^1H\}$ NMR (100.67 MHz, $(CD_3)_2S{=}O$) δ: 151.1 (s), 148.9 (s), 148.0 (s), 147.3 (s), 136.5 (s), 135.0 (s), 134.9 (s), 134.6 (s), 129.8 (s), 128.5 (s), 128.2 (s), 126.1 (s), 124.0 (s), 122.1 (s).

MS (ESI-TOF, CH_3OH) m/z: $[M + H]^+$ calculated for $C_{14}H_{11}N_2$ 207.09, found 207.09.

1-[4-[1-(Phenylmethyl)-1H-pyrazol-4-yl]phenyl]ethenone (33)

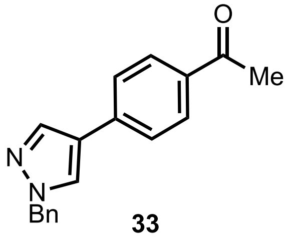

33

4-Bromophenylethanone (40 mg, 0.20 mmol, 1.0 equiv) and 1-benzyl-4-(4,4,5,5-tetramethyl-1,3,2-dioxaborolan-2-yl)-1H-pyrazole (68 mg, 0.24 mmol, 1.2 equiv) were used to give a white solid (51 mg, 0.18 mmol, 92%).

^{1}H NMR (400.30 MHz, $CDCl_3$) δ: 7.94 (dq, *J* = 8.2, 1.5 Hz, 2H), 7.89 (q, *J* = 1.1 Hz, 1H), 7.70 (q, *J* = 1.1 Hz, 1H), 7.54 (dq, *J* = 8.3, 1.6 Hz, 2H), 7.37 (tdd, *J* = 8.1, 6.5, 3.5 Hz, 3H), 7.28 (d, *J* = 7.6 Hz, 2H), 5.36 (s, 2H, CH_2 in Bn), 2.65 – 2.55 (2, 3H, CH_3).

$^{13}C\{^1H\}$ NMR (100.67 MHz, $CDCl_3$) δ: 197.6 (s, C=O), 137.5 (s), 137.4 (s), 136.2 (s), 135.2 (s), 129.3 (s), 129.1 (s), 128.5 (s), 128.0 (s), 127.0 (s), 125.4 (s), 122.6 (s), 56.6 (s, CH_2 in Bn), 26.7 (s, CH_3).

HRMS (ESI-TOF, CH_3CN) m/z: $[M + H]^+$ calculated for $C_{18}H_{17}N_2O$ 277.1335, found 277.1328.

3-(1-benzyl-1H-pyrazol-4-yl)pyridin-2-amine[8] (34)

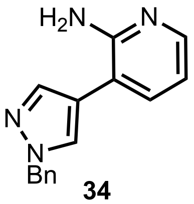

34

3-Bromopyridin-2-amine (33 mg, 0.20 mmol, 1.0 equiv) and 1-benzyl-4-(4,4,5,5-tetramethyl-1,3,2-dioxaborolan-2-yl)-1H-pyrazole (68 mg, 0.24 mmol, 1.2 equiv) were used to give a white solid (43 mg, 0.17 mmol, 87%).

^{1}H NMR (400.30 MHz, $(CD_3)_2S{=}O$) δ: 8.18 (d, *J* = 0.8 Hz, 1H), 7.87 (dd, *J* = 4.9, 1.8 Hz, 1H), 7.77 (d, *J* = 0.9 Hz, 1H), 7.49 (dd, *J* = 7.4, 1.8 Hz, 1H), 7.39 – 7.24 (m, 5H, Ph in Bn), 6.61 (dd, *J* = 7.4, 4.9 Hz, 1H), 5.59 (s, 2H, NH_2), 5.35 (s, 2H, CH_2 in Bn).

$^{13}C\{^1H\}$ NMR (100.67 MHz, $(CD_3)_2S{=}O$) δ: 156.2 (s), 145.9 (s), 138.0 (s), 137.4 (s), 135.9 (s), 128.6 (s), 128.5 (s), 127.8 (s), 127.7 (s), 118.0 (s), 113.2 (s), 112.2 (s), 55.0 (s, CH_2 in Bn).

MS (ESI-TOF, CH_3CN) m/z: $[M + H]^+$ calculated for $C_{15}H_{14}N_4$ 251.13, found 251.13.

5-[1-(Phenylmethyl)-1H-pyrazol-4-yl]pyrimidine[24] (35)

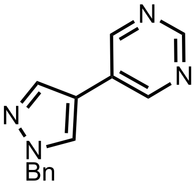

35

5-Bromopyrimidine (32 mg, 0.20 mmol, 1.0 equiv) and 1-benzyl-4-(4,4,5,5-tetramethyl-1,3,2-dioxaborolan-2-yl)-1H-pyrazole (68 mg, 0.24 mmol, 1.2 equiv) were used to give a white solid (41 mg, 0.18 mmol, 91%).

^{1}H NMR (400.30 MHz, $CDCl_3$) δ: 9.06 (s, 1H), 8.82 (s, 2H), 7.87 (d, *J* = 0.8 Hz, 1H), 7.71 (d, *J* = 0.8 Hz, 1H), 7.43 – 7.33 (m, 3H), 7.31 – 7.27 (m, 2H), 5.37 (s, 2H, CH_2 in Bn).

$^{13}C\{^1H\}$ NMR (100.67 MHz, $CDCl_3$) δ: 156.8 (s), 153.5 (s), 137.0 (s), 135.8 (s), 129.2 (s), 128.6 (s), 128.0 (s), 127.0 (s), 126.9 (s), 116.6 (s), 56.7 (s, CH_2 in Bn).

MS (ESI-TOF, CH_3CN) m/z: $[M + H]^+$ calculated for $C_{14}H_{13}N_4$ 237.11, found 237.11.

[24] Handa, S.; Wang, Y.; Gallou, F.; Lipshutz, B. H. Sustainable Fe–Ppm Pd Nanoparticle Catalysis of Suzuki-Miyaura Cross-Couplings in Water. *Science* **2015**, *349* (6252), 1087–1091.

5-(5-Pyrimidinyl)-1H-indole[25] (36)

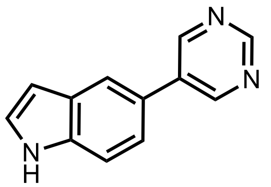

36

5-Bromopyrimidine (32 mg, 0.20 mmol, 1.0 equiv) and 5-(4,4,5,5-tetramethyl-1,3,2-dioxaborolan-2-yl)-1H-indole (58 mg, 0.24 mmol, 1.2 equiv) were used to give a white solid (36 mg, 0.18 mmol, 92%). This compound can be synthesized by one other literature procedure (no HRMS data reported). The NMR data was reported using $CDCl_3$, but we found its low solubility in $CDCl_3$. Thus, $(CD_3)_2S=O$ was used as the solvent.

^{1}H NMR (400.30 MHz, $(CD_3)_2S=O$) δ: 11.28 (s, 1H, NH), 9.13 (s, 2H), 9.11 (s, 1H), 8.01 – 7.96 (m, 1H), 7.58 – 7.48 (m, 2H), 7.43 (t, *J* = 2.8 Hz, 1H), 6.53 (ddd, *J* = 3.0, 2.0, 0.8 Hz, 1H).

^{13}C{^{1}H} NMR (100.67 MHz, $(CD_3)_2S=O$) δ: 156.2 (s), 154.4 (s), 136.2 (s), 134.8 (s), 128.4 (s), 126.7 (s), 124.5 (s), 120.0 (s), 118.8 (s), 112.4 (s), 101.8 (s).

HRMS (ESI-TOF, CH_3CN) m/z: $[M + H]^+$ calculated for $C_{12}H_{10}N_3$ 196.0869, found 196.0889.

5-(3-Pyridinyl)pyrimidine[26] (37)

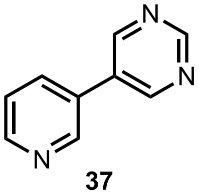

37

5-Bromopyrimidine (32 mg, 0.20 mmol, 1.0 equiv) and 3-(4,4,5,5-tetramethyl-1,3,2-dioxaborolan-2-yl)pyridine (49 mg, 0.24 mmol, 1.2 equiv) were used to give a white solid (18 mg, 0.11 mmol, 57%). Isolated mixture contains a small amount of 5,5'-bipyrimidine byproduct (15%), which was very difficult to be removed due to the similar polarity.[26]

^{1}H NMR (400.30 MHz, $CDCl_3$) δ: 9.28 (s, 1H), 8.98 (s, 2H), 8.87 (dd, *J* = 2.4, 0.9 Hz, 1H), 8.74 (dd, *J* = 4.9, 1.6 Hz, 1H), 7.91 (ddd, *J* = 7.9, 2.4, 1.6 Hz, 1H), 7.47 (ddd, *J* = 7.9, 4.9, 0.9 Hz, 1H).

^{13}C{^{1}H} NMR (100.67 MHz, $CDCl_3$) δ: 158.3 (s), 155.0 (s), 150.3 (s), 148.0 (s), 134.4 (s), 131.5 (s), 130.2 (s), 124.1 (s).

MS (ESI-TOF, CH_3CN) m/z: $[M + H]^+$ calculated for $C_9H_8N_3$ 158.07, found 158.07.

---

[25] Lou, S.; Fu, G. C. Palladium/Tris(Tert-Butyl)Phosphine-Catalyzed Suzuki Cross-Couplings in the Presence of Water. *Adv. Synth. Catal.* **2010**, *352* (11–12), 2081–2084.

[26] Ye, M.; Gao, G.-L.; Edmunds, A. J. F.; Worthington, P. A.; Morris, J. A.; Yu, J.-Q. Ligand-Promoted C3-Selective Arylation of Pyridines with Pd Catalysts: Gram-Scale Synthesis of (±)-Preclamol. *J. Am. Chem. Soc.* **2011**, *133* (47), 19090–19093.

6-Phenylquinoline[27] (38)

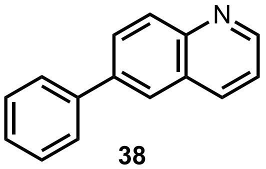

38

6-Chloroquinoline (33 mg, 0.20 mmol, 1.0 equiv) and phenylboronic acid (29 mg, 0.24 mmol, 1.2 equiv) were used to give a white solid (38 mg, 0.19 mmol, 93%).

^{1}H NMR (400.30 MHz, CDCl$_3$) δ: 8.92 (dd, *J* = 4.3, 1.7 Hz, 1H), 8.24 – 8.15 (m, 2H), 8.03 – 7.97 (m, 2H), 7.77 – 7.69 (m, 2H), 7.54 – 7.47 (m, 2H), 7.45 – 7.38 (m, 2H).

^{13}C{^{1}H} NMR (100.67 MHz, CDCl$_3$) δ: 150.5 (s), 147.9 (s), 140.5 (s), 139.5 (s), 136.4 (s), 130.1 (s), 129.4 (s), 129.1 (s), 128.6 (s), 127.9 (s), 127.6 (s), 125.6 (s), 121.6 (s).

MS (ESI-TOF, CH$_3$CN) m/z: [M + H]$^+$ calculated for $C_{15}H_{12}N$ 206.09, found 206.09.

6-(4-Methoxyphenyl)quinoline[28] (39)

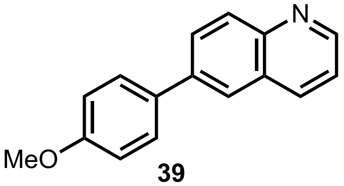

39

6-Chloroquinoline (33 mg, 0.20 mmol, 1.0 equiv) and (4-methoxyphenyl)boronic acid (37mg, 0.24 mmol, 1.2 equiv) were used to give a white solid (44 mg, 0.19 mmol, 94%).

^{1}H NMR (400.30 MHz, CDCl$_3$) δ: 8.90 (dd, *J* = 4.2, 1.7 Hz, 1H), 8.25 – 8.11 (m, 2H), 7.99 – 7.94 (m, 2H), 7.71 – 7.63 (m, 2H), 7.44 – 7.38 (m, 1H), 7.08 – 7.01 (m, 2H), 3.88 (s, 3H, OMe).

^{13}C{^{1}H} NMR (100.67 MHz, CDCl$_3$) δ: 159.7 (s), 150.3 (s), 147.6 (s), 139.1 (s), 136.2 (s), 132.9 (s), 123.0 (s), 129.2 (s), 128.7 (s), 128.7 (s), 124.8 (s), 121.6 (s), 114.6 (s), 55.6 (s, OMe).

MS (ESI-TOF, CH$_3$CN) m/z: [M + H]$^+$ calculated for $C_{16}H_{14}NO$ 236.10, found 236.10.

6-(4-fluorophenyl)quinoline[29] (40)

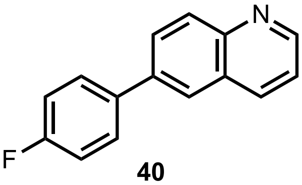

40

[27] Xu, L.; Li, B.-J.; Wu, Z.-H.; Lu, X.-Y.; Guan, B.-T.; Wang, B.-Q.; Zhao, K.-Q.; Shi, Z.-J. Nickel-Catalyzed Efficient and Practical Suzuki−Miyaura Coupling of Alkenyl and Aryl Carbamates with Aryl Boroxines. *Org. Lett.* **2010**, *12* (4), 884–887.

[28] Beromi, M. M.; Nova, A.; Balcells, D.; Brasacchio, A. M.; Brudvig, G. W.; Guard, L. M.; Hazari, N.; Vinyard, D. J. Mechanistic Study of an Improved Ni Precatalyst for Suzuki–Miyaura Reactions of Aryl Sulfamates: Understanding the Role of Ni(I) Species. *J. Am. Chem. Soc.* **2017**, *139* (2), 922–936.

[29] Sahoo, B.; Kreyenschulte, C.; Agostini, G.; Lund, H.; Bachmann, S.; Scalone, M.; Junge, K.; Beller, M. A Robust Iron Catalyst for the Selective Hydrogenation of Substituted (Iso)Quinolones. *Chem. Sci.* **2018**, *9* (42), 8134–8141.

6-Chloroquinoline (33 mg, 0.20 mmol, 1.0 equiv) and (4-fluorophenyl)boronic acid (34 mg, 0.24 mmol, 1.2 equiv) were used to give a white solid (41 mg, 0.18 mmol, 90%).

^{1}H NMR (400.30 MHz, $CDCl_3$) δ: 8.93 (dd, *J* = 4.2, 1.7 Hz, 1H), 8.25 – 8.13 (m, 2H), 7.99 – 7.90 (m, 2H), 7.72 – 7.63 (m, 2H), 7.44 (dd, *J* = 8.3, 4.2 Hz, 1H), 7.23 – 7.16 (m, 2H).

^{13}C{^{1}H} NMR (100.67 MHz, $CDCl_3$) δ: 164.1 (s), 161.7 (s), 150.6 (s), 147.8 (s), 138.5 (s), 136.6 (d, *J* = 3 Hz), 136.3 (s), 130.2 (s), 129.2 (d, *J* = 8 Hz), 128.6 (s), 125.5 (s), 121.7 (s), 116.0 (d, *J* = 22 Hz).

^{19}F{^{1}H} NMR (470.61 MHz, $CDCl_3$) δ: -114.88 (s).

MS (ESI-TOF, CH_3CN) m/z: $[M + H]^+$ calculated for $C_{15}H_{11}NF$ 224.08, found 224.08.

[3,3']-Bipyridinyl-2-ylamine[30] (41)

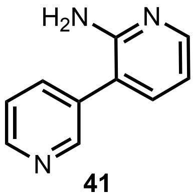

41

3-Bromopyridin-2-amine (33 mg, 0.20 mmol, 1.0 equiv) and pyridin-3-ylboronic acid (37 mg, 0.30 mmol, 1.5 equiv) were used to give a white solid (20 mg, 0.12 mmol, 58%).

^{1}H NMR (400.30 MHz, $(CD_3)_2S{=}O$) δ: 8.61 (dd, *J* = 2.4, 0.9 Hz, 1H), 8.56 (dd, *J* = 4.8, 1.6 Hz, 1H), 7.99 (dd, *J* = 5.0, 1.9 Hz, 1H), 7.86 (dt, *J* = 7.8, 2.0 Hz, 1H), 7.46 (ddd, *J* = 7.9, 4.8, 0.9 Hz, 1H), 7.36 (dd, *J* = 7.3, 1.9 Hz, 1H), 6.68 (dd, *J* = 7.3, 4.9 Hz, 1H), 5.70 (s, 2H, NH_2).

^{13}C{^{1}H} NMR (100.67 MHz, $(CD_3)_2S{=}O$) δ: 156.8 (s), 149.2 (s), 148.3 (s), 147.8 (s), 138.0 (s), 136.1 (s), 134.1 (s), 123.8 (s), 117.1 (s), 113.1 (s).

MS (ESI-TOF, CH_3OH) m/z: $[M + H]^+$ calculated for $C_{10}H_{10}N_3$ 172.09, found 172.09.

[3,3′-Bipyridine]-2-carbonitrile (42)

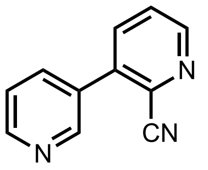

42

3-Chloropicolinonitrile (28 mg, 0.20 mmol, 1.0 equiv) and pyridin-3-ylboronic acid (37 mg, 0.30 mmol, 1.5 equiv) were used to give a white solid (30 mg, 0.17 mmol, 83%).

^{1}H NMR (400.30 MHz, $CDCl_3$) δ: 8.81 – 8.74 (m, 3H), 7.98 (ddd, *J* = 7.9, 2.4, 1.6 Hz, 1H), 7.89 (dd, *J* = 8.0, 1.6 Hz, 1H), 7.65 (dd, *J* = 8.0, 4.7 Hz, 1H), 7.49 (ddd, *J* = 7.9, 4.9, 0.9 Hz, 1H)

[30] Kearney, A. M.; Vanderwal, C. D. Synthesis of Nitrogen Heterocycles by the Ring Opening of Pyridinium Salts. *Angew. Chem. Int. Ed.* **2006**, *45* (46), 7803–7806.

$^{13}C\{^1H\}$ NMR (100.67 MHz, $CDCl_3$) δ: 150.8 (s), 150.4 (s), 149.4 (s), 138.9 (s), 137.7 (s), 136.2 (s), 132.7 (s), 131.5 (s), 127.0 (s), 123.8 (s), 116.6 (s, CN).

HRMS (ESI-TOF, CH_3CN) m/z: $[M + H]^+$ calculated for $C_{11}H_8N_3$ 182.0713, found 182.0698.

6-(2,4-Dimethoxy-5-pyrimidinyl)quinoline (43)

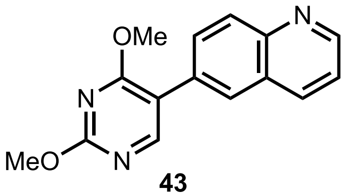

43

6-Chloroquinoline (33 mg, 0.20 mmol, 1.0 equiv) and (2,4-dimethoxypyrimidin-5-yl)boronic acid (55 mg, 0.30 mmol, 1.5 equiv) were used to give a white solid (51 mg, 0.19 mmol, 95%).

^1H NMR (400.30 MHz, $CDCl_3$) δ: 8.93 (dd, *J* = 4.2, 1.7 Hz, 1H), 8.38 (s, 1H), 8.22 – 8.12 (m, 2H), 7.94 – 7.82 (m, 2H), 7.47 – 7.40 (m, 1H), 4.07 (s, 3H, OCH_3), 4.07 (s, 3H, OCH_3).

$^{13}C\{^1H\}$ NMR (100.67 MHz, $CDCl_3$) δ: 168.4 (s), 165.0 (s), 158.1 (s), 150.9 (s), 147.8 (s), 136.3 (s), 131.9 (s), 130.7 (s), 129.7 (s), 128.4 (s), 127.6 (s), 121.6 (s), 115.7 (s), 55.1 (s, OCH_3), 54.4 (s, OCH_3).

HRMS (ESI-TOF, CH_3CN) m/z: $[M + H]^+$ calculated for $C_{15}H_{13}N_3O_2$ 268.1081, found 268.1061.

2,4-Dimethoxy-5-(2-pyridinyl)pyrimidine[31] (44)

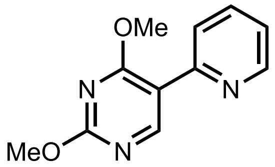

44

2-Chloropyridine (19 mL, 0.20 mmol, 1.0 equiv) and (2,4-dimethoxypyrimidin-5-yl)boronic acid (55 mg, 0.30 mmol, 1.5 equiv) were used to give a white solid (41 mg, 0.19 mmol, 94%).

^1H NMR (400.30 MHz, $CDCl_3$) δ: 8.90 (s, 1H), 8.67 (ddd, *J* = 4.9, 1.9, 1.0 Hz, 1H), 7.83 (dt, *J* = 8.1, 1.1 Hz, 1H), 7.72 (ddd, *J* = 8.1, 7.4, 1.8 Hz, 1H), 7.22 (ddd, *J* = 7.5, 4.9, 1.2 Hz, 1H), 4.09 (s, 3H, OCH_3), 4.06 (s, 3H, OCH_3).

$^{13}C\{^1H\}$ NMR (100.67 MHz, $CDCl_3$) δ: 168.4 (s), 165.2 (s), 160.0 (s), 152.0 (s), 149.8 (s), 136.4 (s), 124.1 (s), 122.3 (s), 114.8 (s), 55.2 (s), 54.3 (s).

MS (ESI-TOF, CH_3CN) m/z: $[M + H]^+$ calculated for $C_{11}H_{12}N_3O_2$ 218.09, found 218.09.

[31] Noël, T.; Musacchio, A. J. Suzuki–Miyaura Cross-Coupling of Heteroaryl Halides and Arylboronic Acids in Continuous Flow. *Org. Lett.* **2011**, *13* (19), 5180–5183.

6.3 NMR spectra for Ni-SMC products

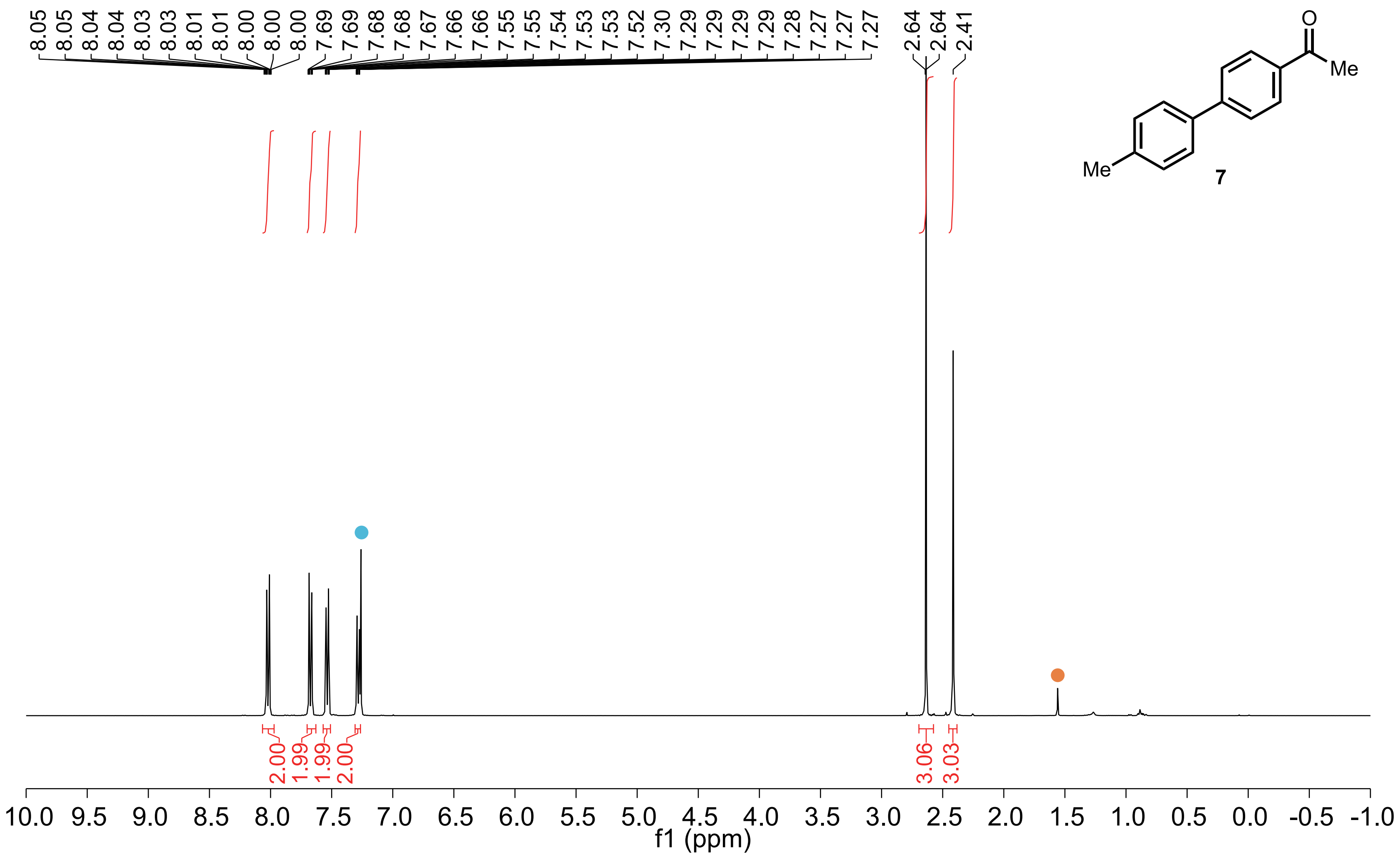

Figure S100 ^1H NMR (400.30 MHz, $CDCl_3$) spectrum of 1-[4-(4-tolyl)phenyl]ethenone (**7**). Residual proteo-solvent (●) and H_2O (●).

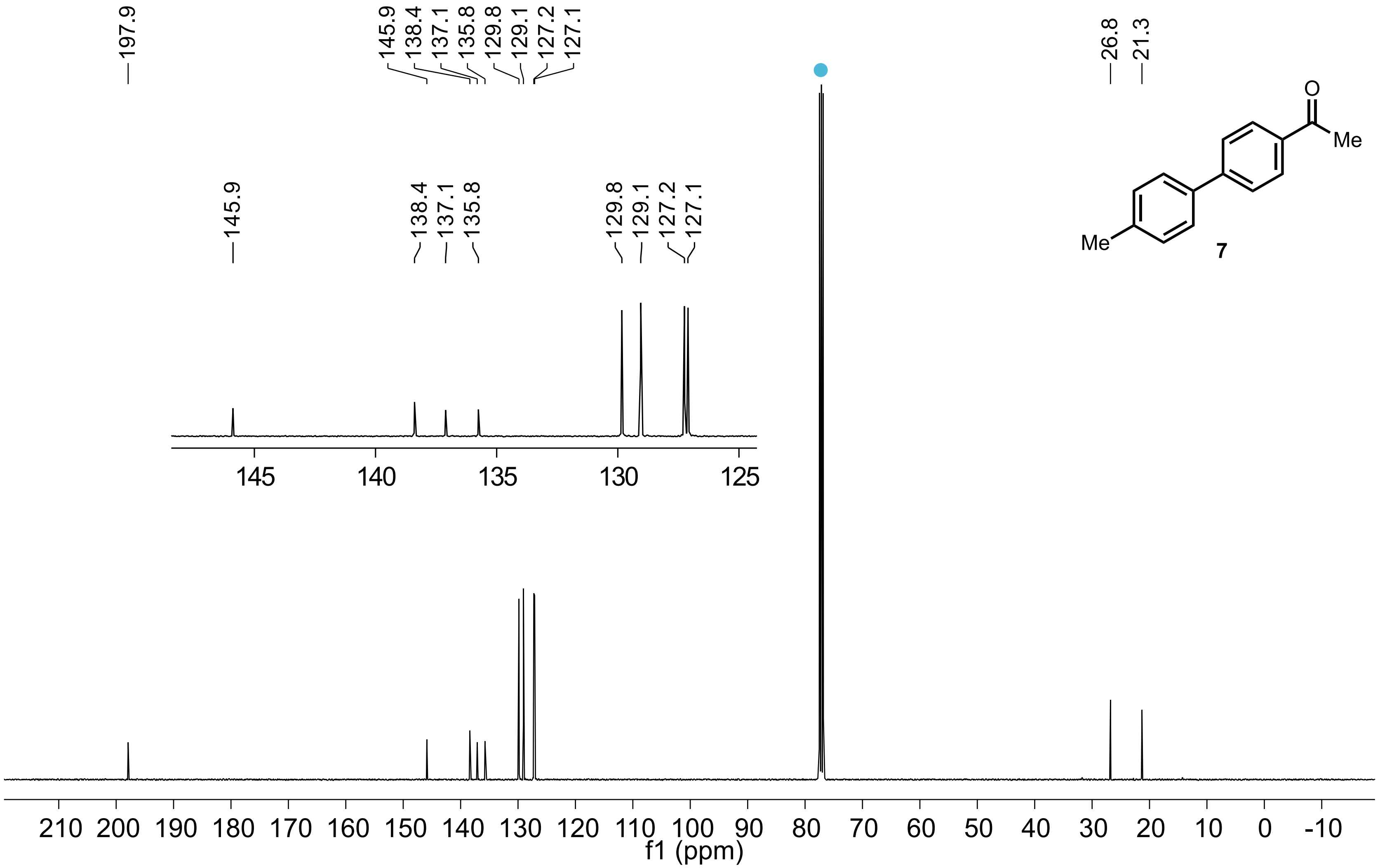

Figure S101 $^{13}C\{^{1}H\}$ NMR (100.67 MHz, $CDCl_3$) spectrum of 1-[4-(4-tolyl)phenyl]ethenone (**7**). Deuterated solvent (•).

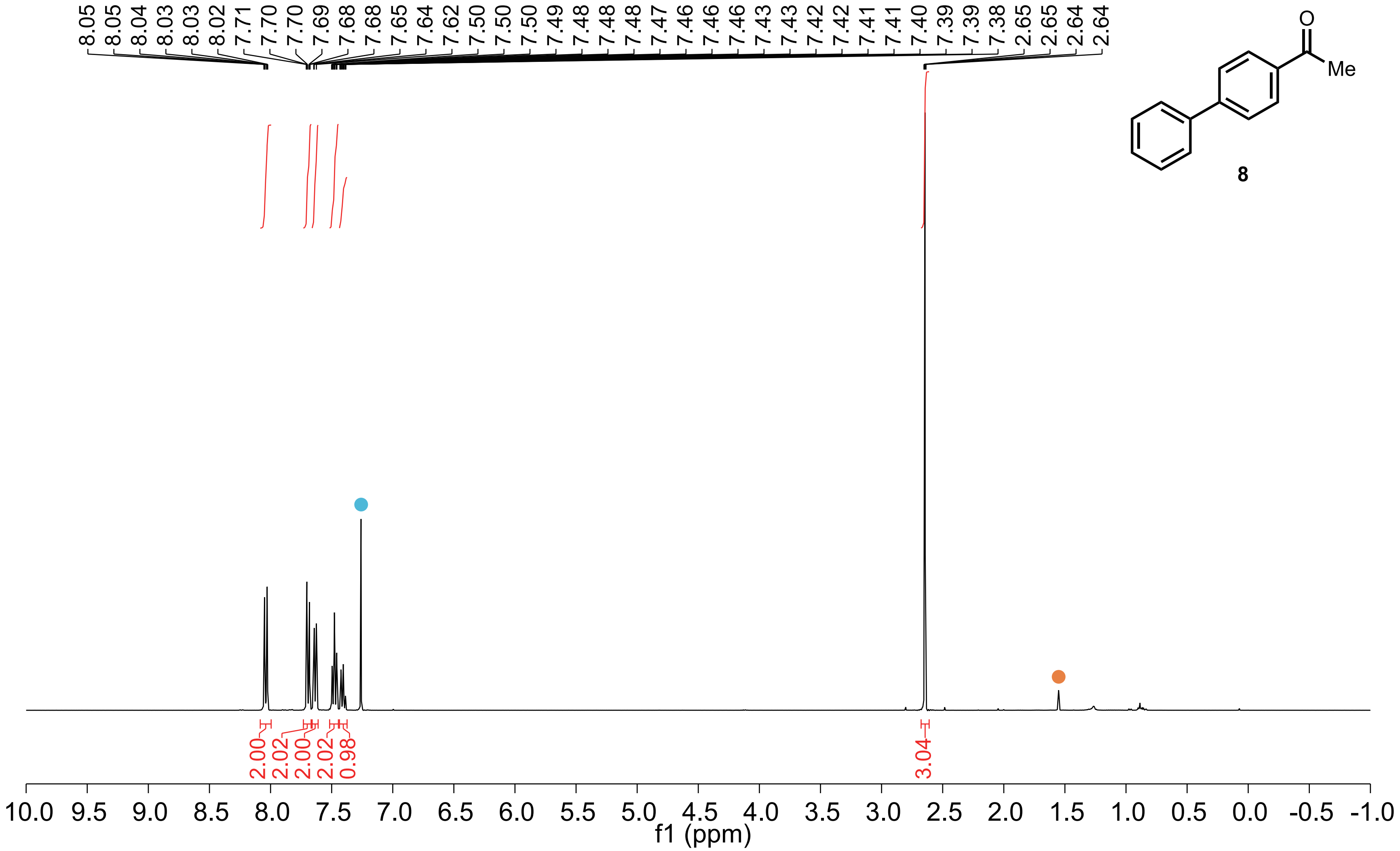

8

Figure S102 ^{1}H NMR (400.30 MHz, $CDCl_3$) spectrum of 1-(4-phenylphenyl)ethenone (**8**). Residual proteo-solvent (•) and H_2O (•).

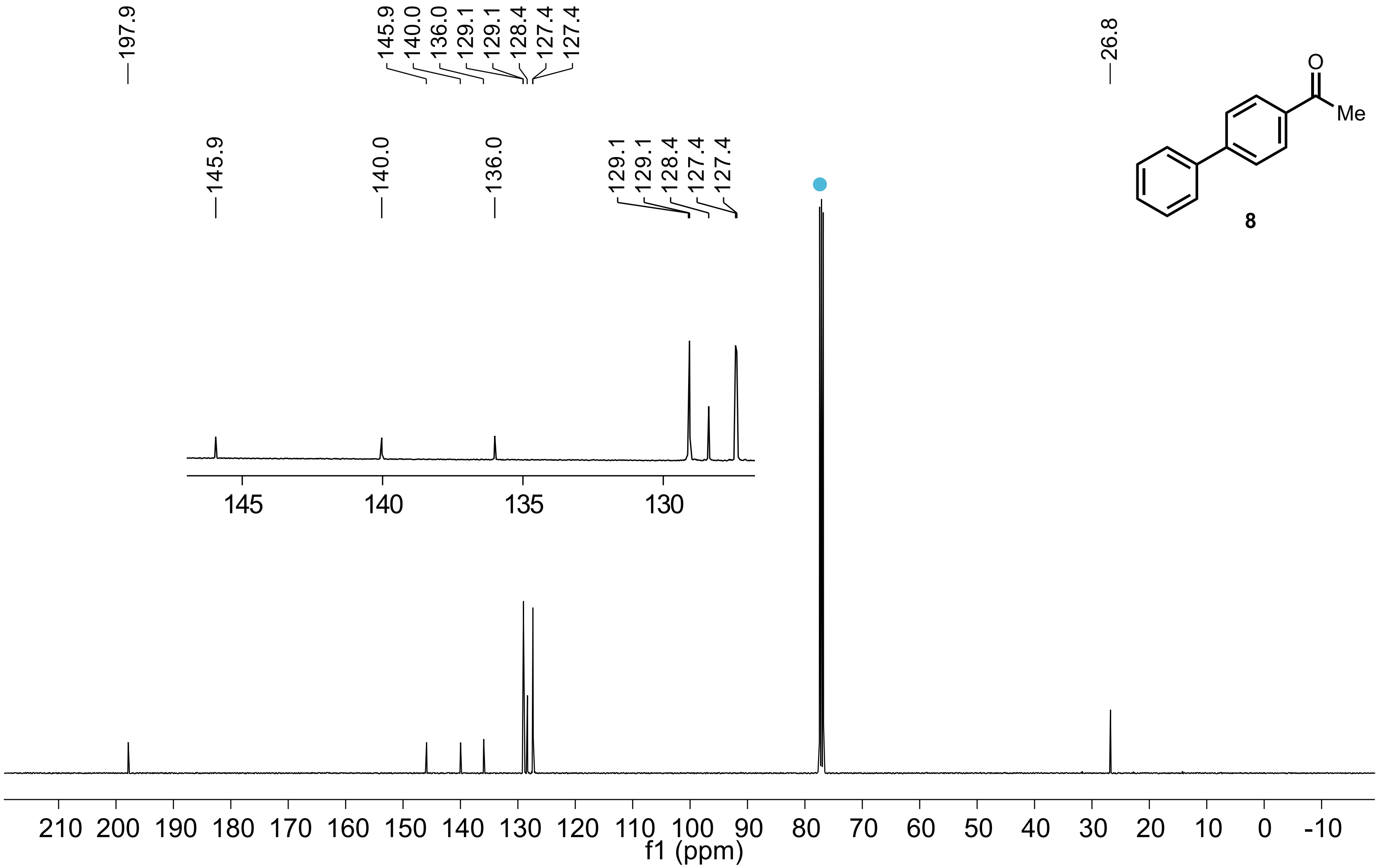

8

Figure S103 $^{13}C\{^1H\}$ NMR (100.67 MHz, $CDCl_3$) spectrum of 1-(4-phenylphenyl)ethenone (**8**). Deuterated solvent (•).

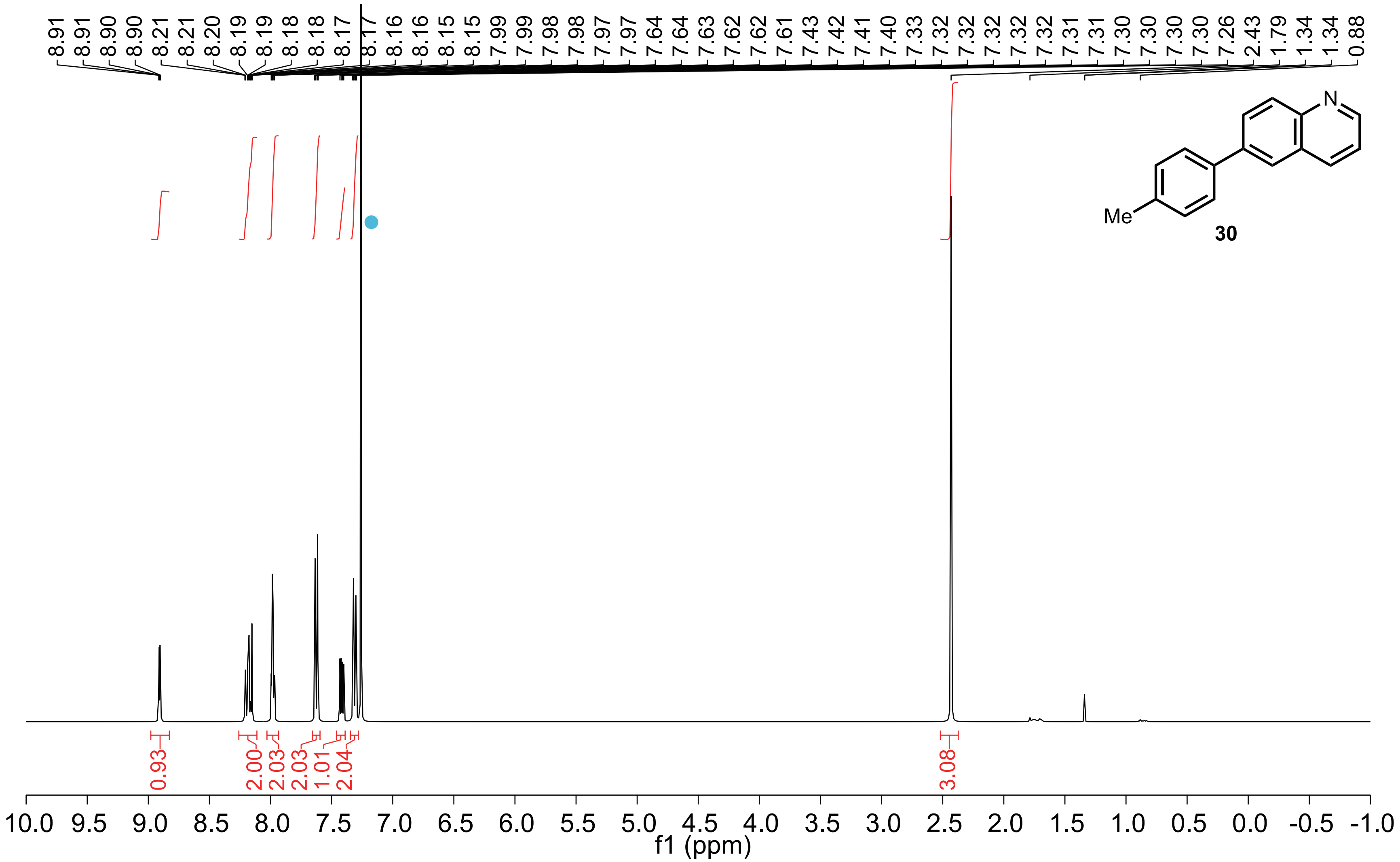

Figure S104 ^{1}H NMR (400.30 MHz, $CDCl_3$) spectrum of 6-(4-methylphenyl)quinoline (**30**). Residual proteo-solvent (•).

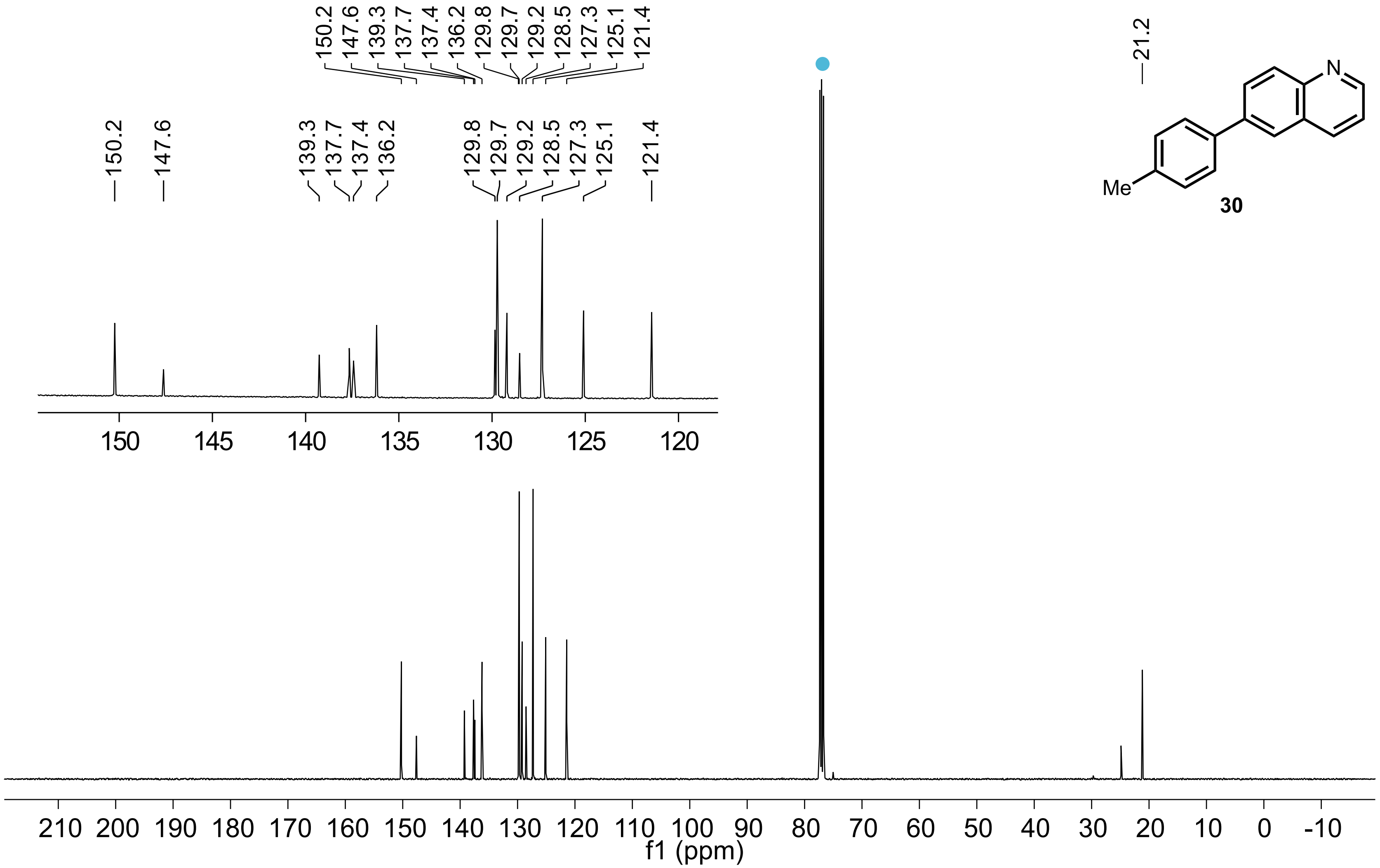

Figure S105 $^{13}C\{^{1}H\}$ NMR (100.67 MHz, $CDCl_3$) spectrum of 6-(4-methylphenyl)quinoline (**30**). Deuterated solvent (•).

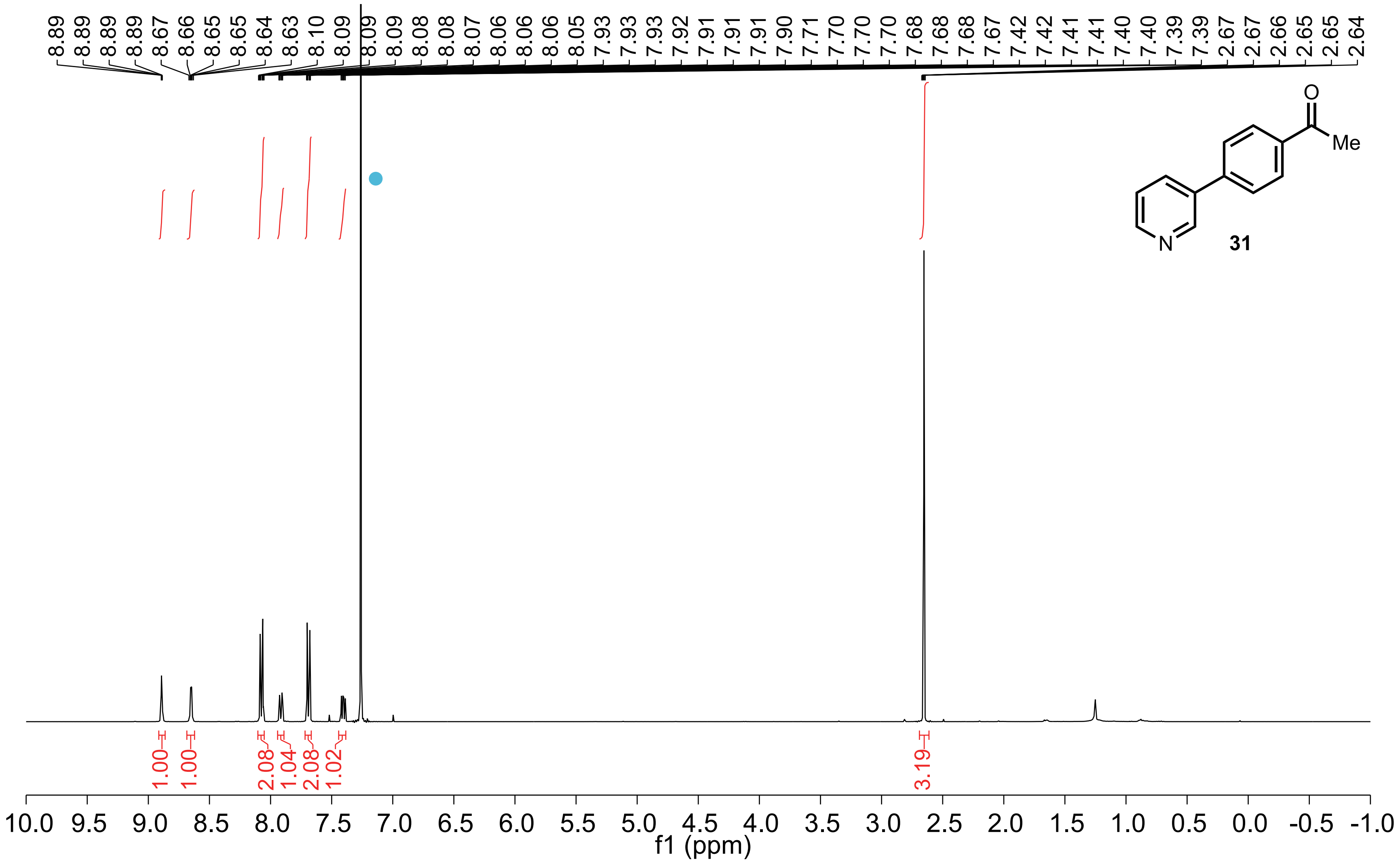

Figure S106 ^{1}H NMR (400.30 MHz, $CDCl_3$) spectrum of 1-[4-(3-pyridinyl)phenyl]ethenone (**31**). Residual proteo-solvent (•).

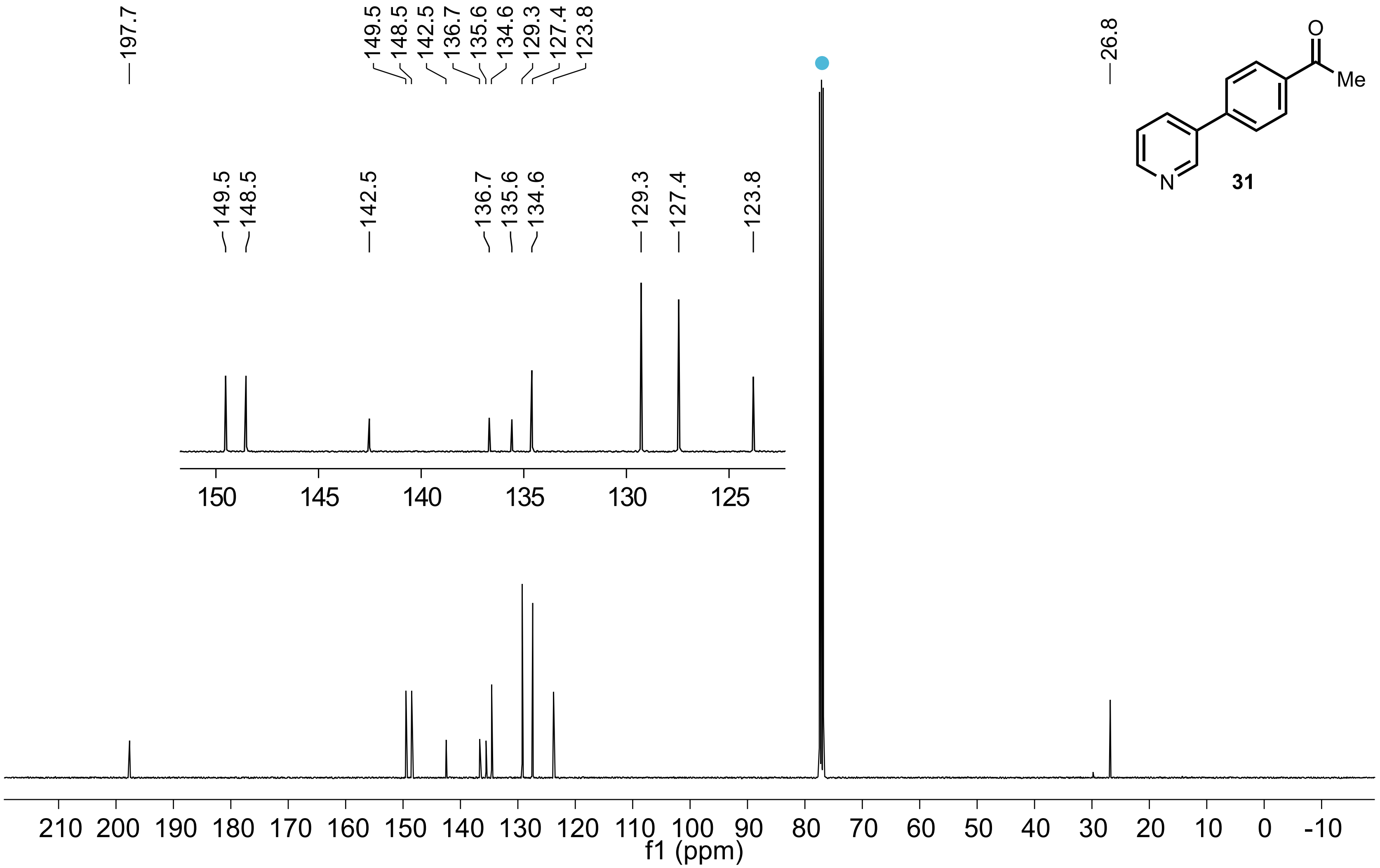

Figure S107 $^{13}C\{^1H\}$ NMR (100.67 MHz, $CDCl_3$) spectrum of 1-[4-(3-pyridinyl)phenyl]ethenone (**31**). Deuterated solvent (•).

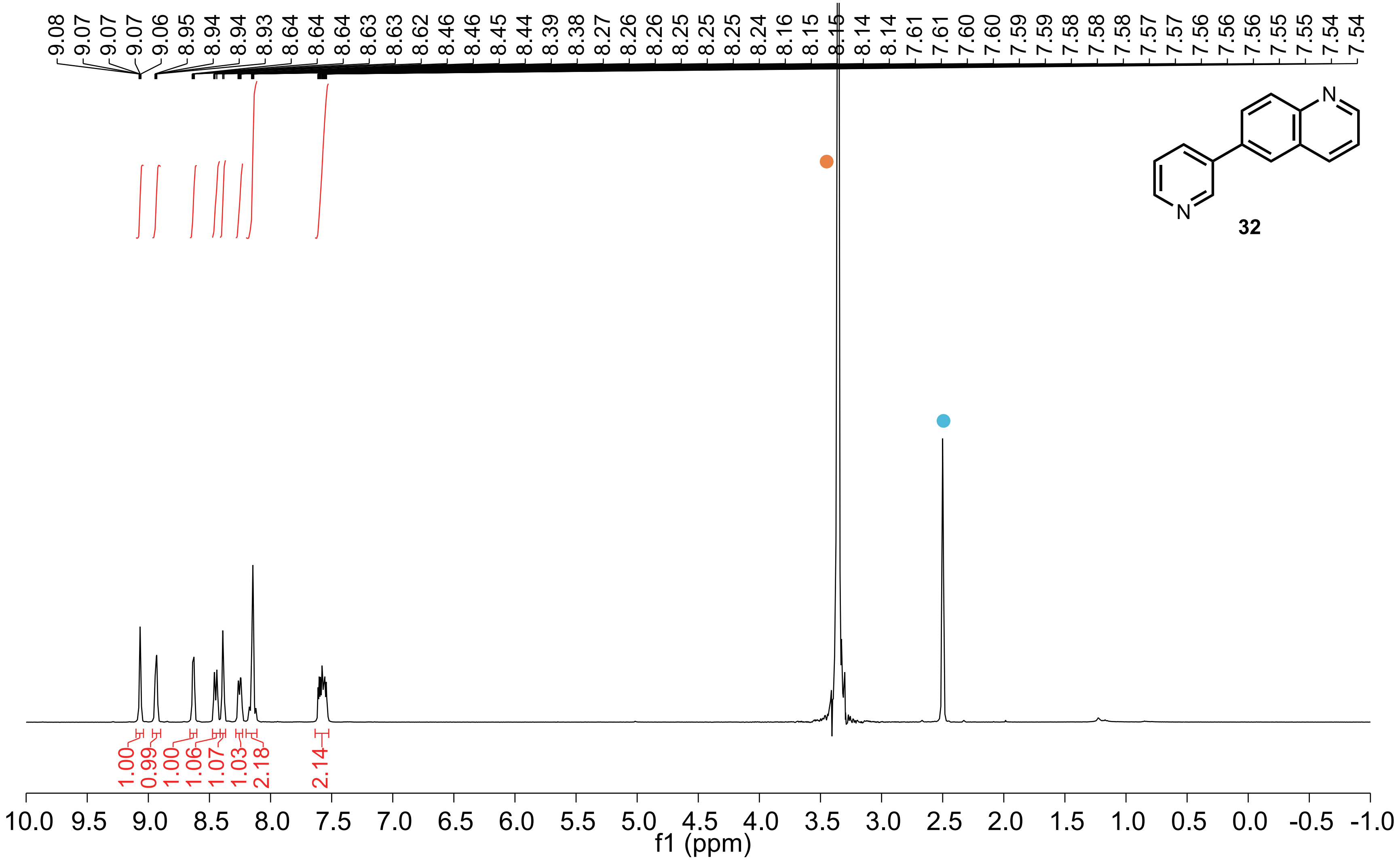

Figure S108 ^{1}H NMR (400.30 MHz, $(CD_3)_2SO$) spectrum of 6-(pyridin-3-yl)quinoline (**32**). Residual proteo-solvent (•) and H_2O (•).

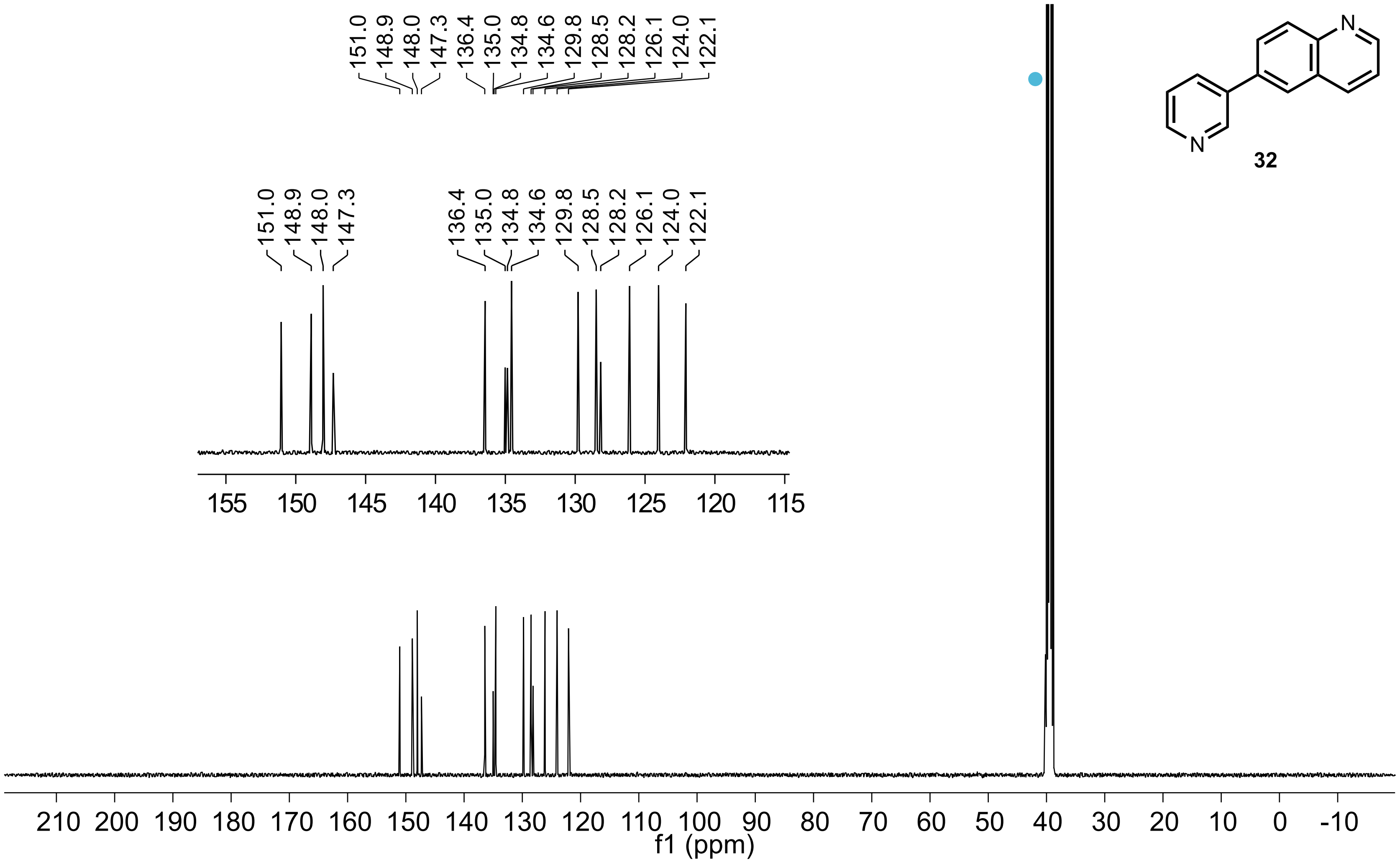

Figure S109 $^{13}C\{^{1}H\}$ NMR (100.67 MHz, $(CD_3)_2SO$) spectrum of 6-(pyridin-3-yl)quinoline (**32**). Deuterated solvent (•).

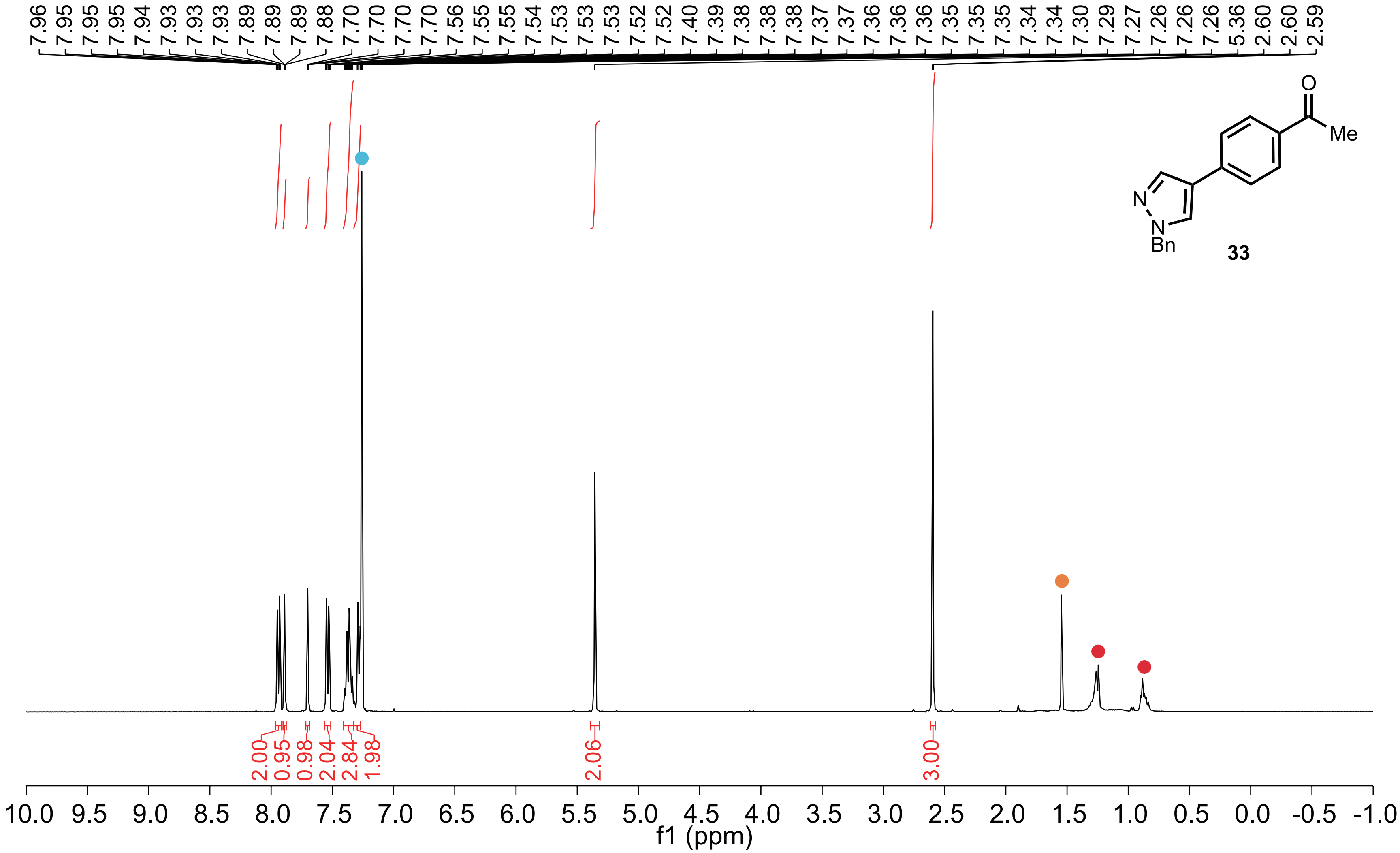

Figure S110 ^{1}H NMR (400.30 MHz, $CDCl_3$) spectrum of 1-[4-[1-(phenylmethyl)-1H-pyrazol-4-yl]phenyl]ethenone (**33**). Residual proteo-solvent (•), H_2O (•) and hexane (•).

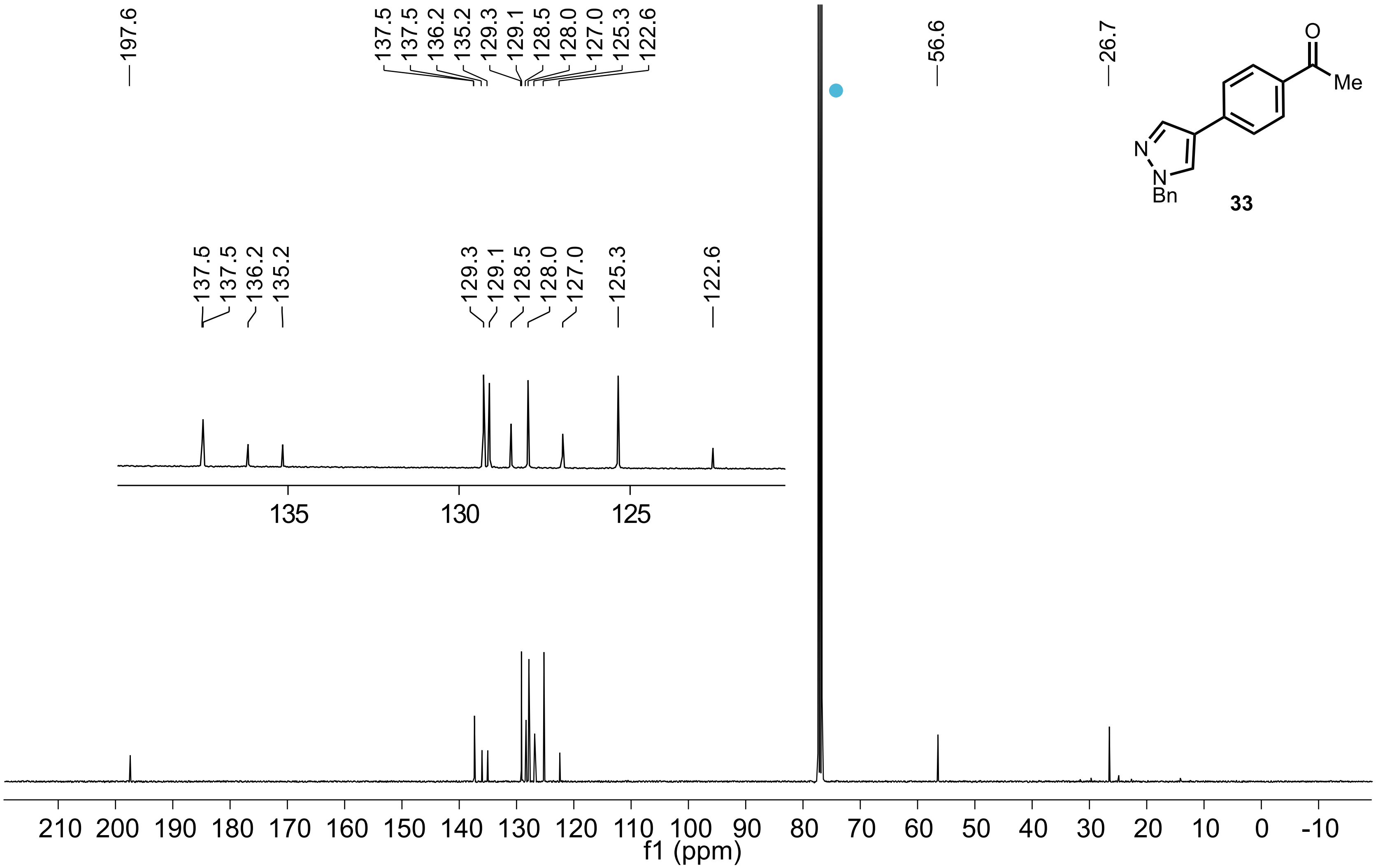

Figure S111 $^{13}C\{^1H\}$ NMR (100.67 MHz, $CDCl_3$) spectrum of 1-[4-[1-(phenylmethyl)-1H-pyrazol-4-yl]phenyl]ethenone (**33**). Deuterated solvent (•).

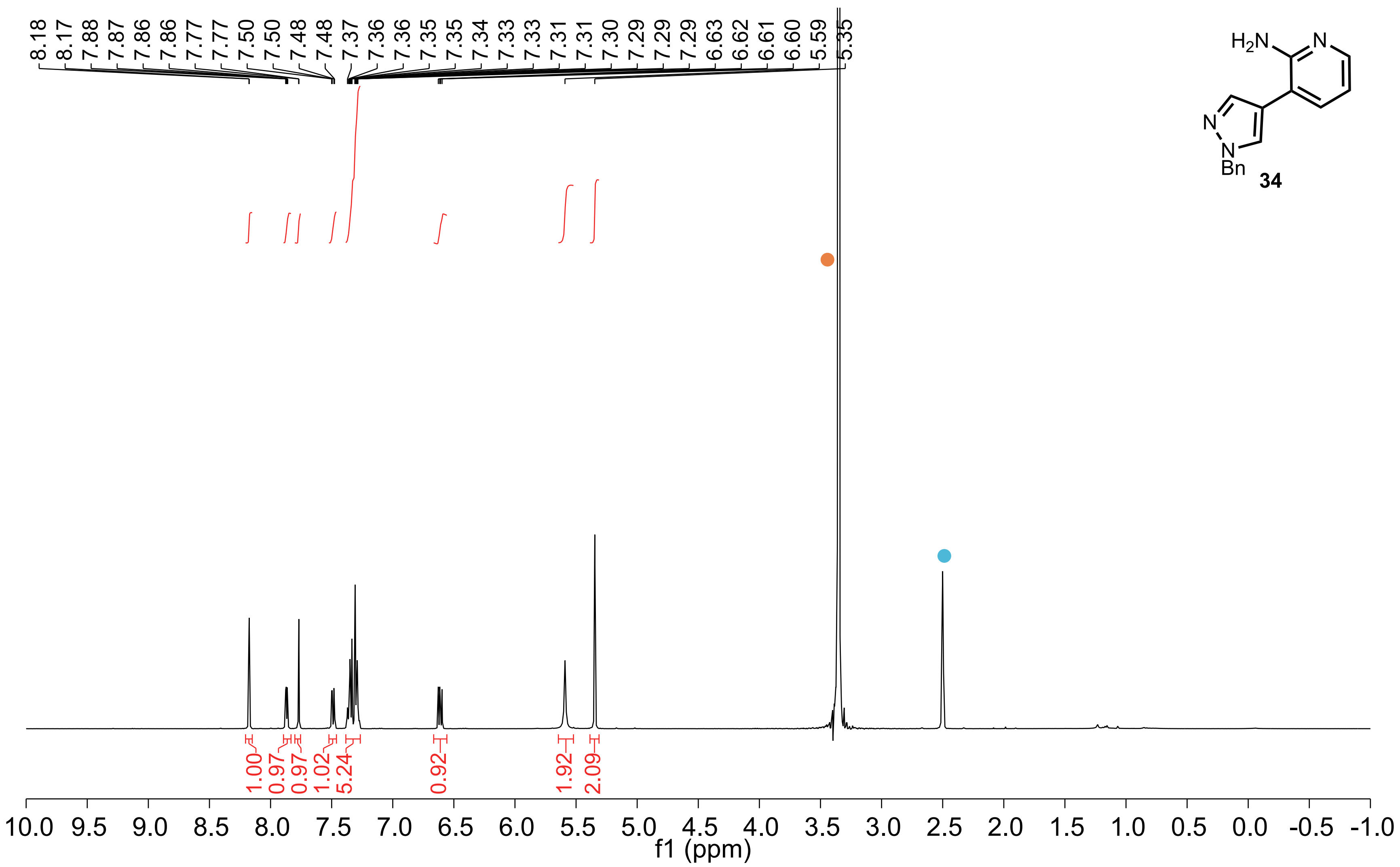

Figure S112 ^{1}H NMR (400.30 MHz, $(CD_3)_2SO$) spectrum of 3-(1-benzyl-1H-pyrazol-4-yl)pyridin-2-amine (**34**). Residual proteo-solvent (•) and H_2O (•).

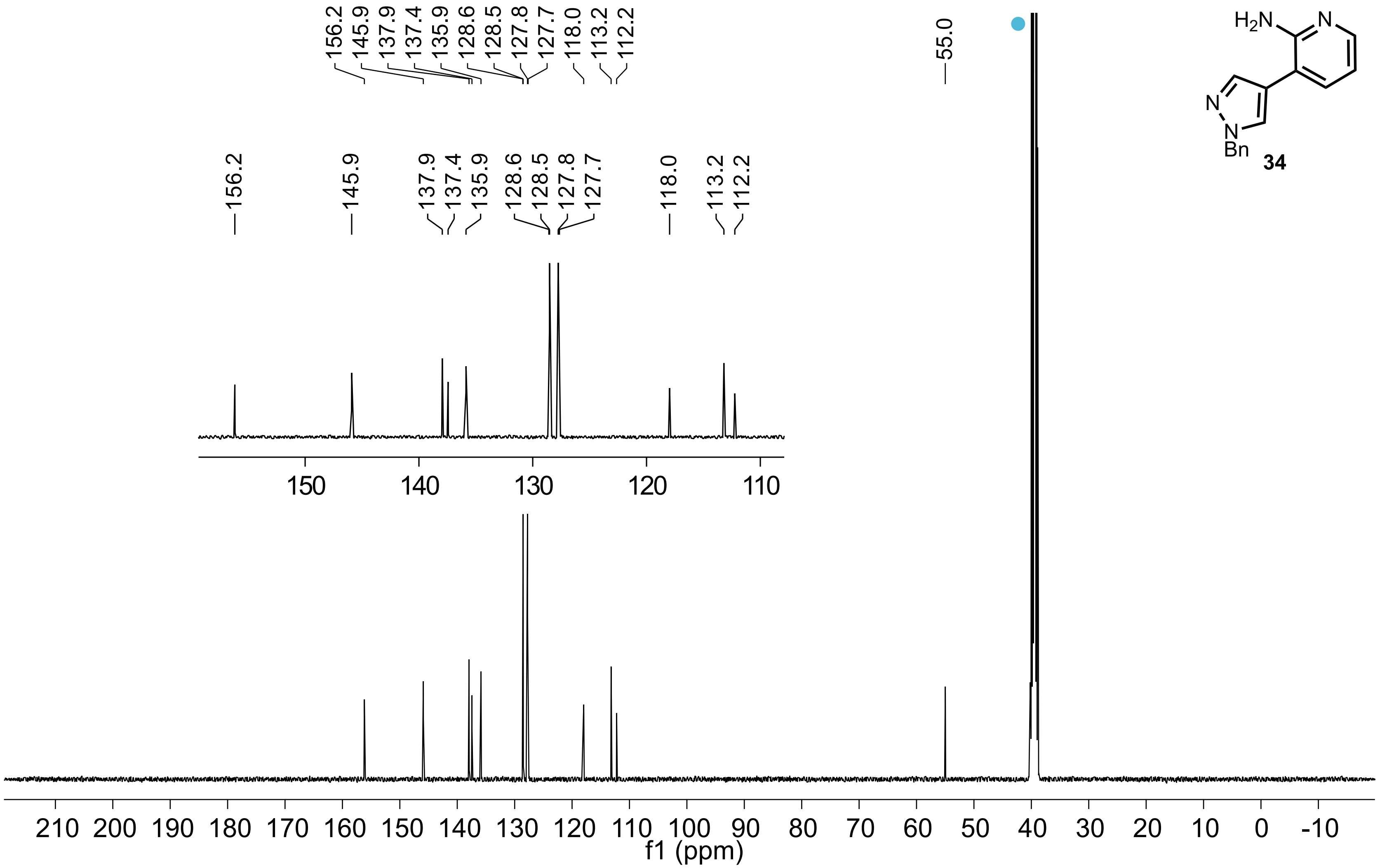

Figure S113 $^{13}C\{^{1}H\}$ NMR (100.67 MHz, $(CD_3)_2SO$) spectrum of 3-(1-benzyl-1H-pyrazol-4-yl)pyridin-2-amine (**34**). Deuterated solvent (•).

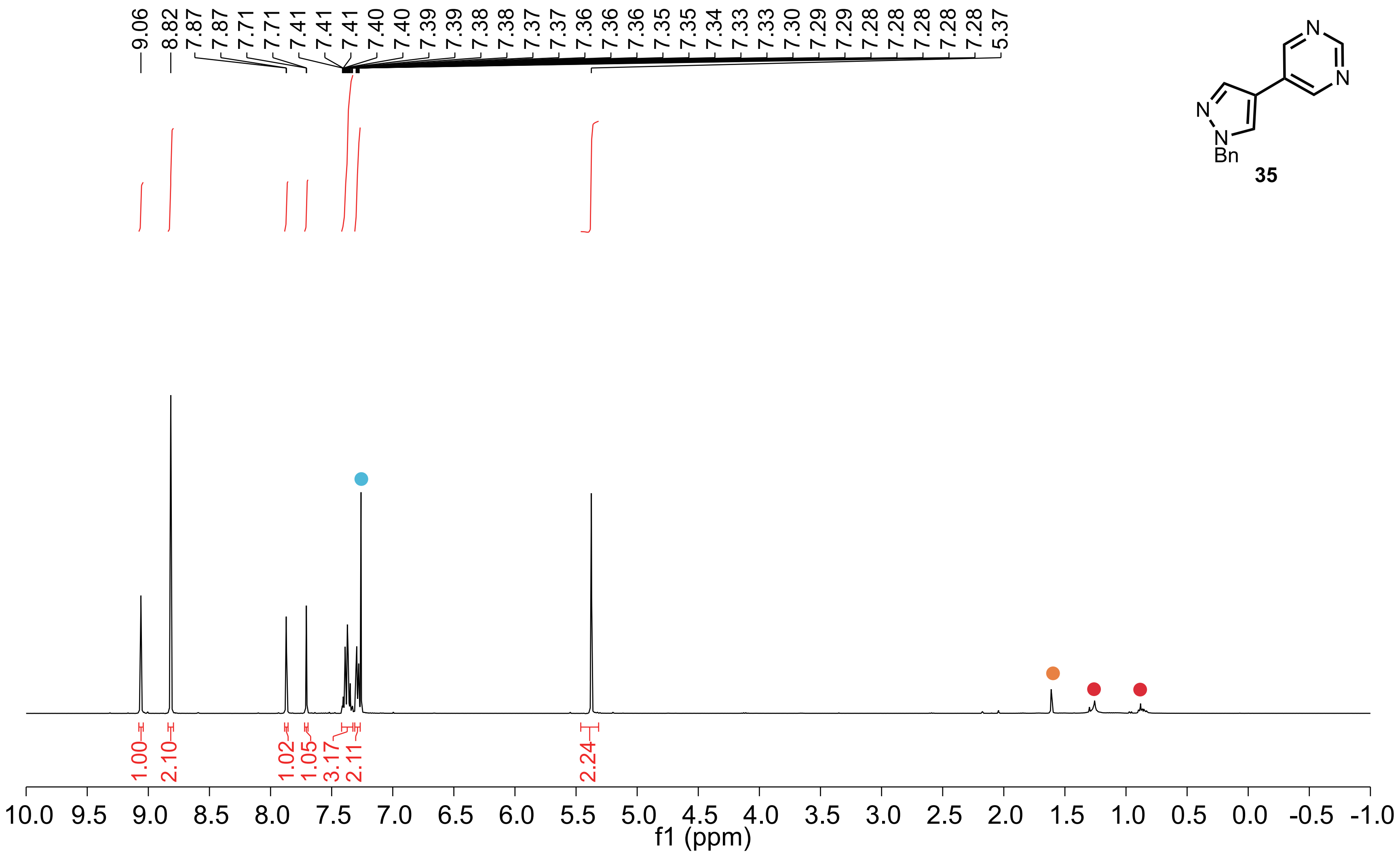

35

Figure S114 ^{1}H NMR (400.30 MHz, $CDCl_3$) spectrum of 5-[1-(phenylmethyl)-1H-pyrazol-4-yl]pyrimidine (**35**). Residual proteo-solvent (•), H_2O (•) and hexane (•).

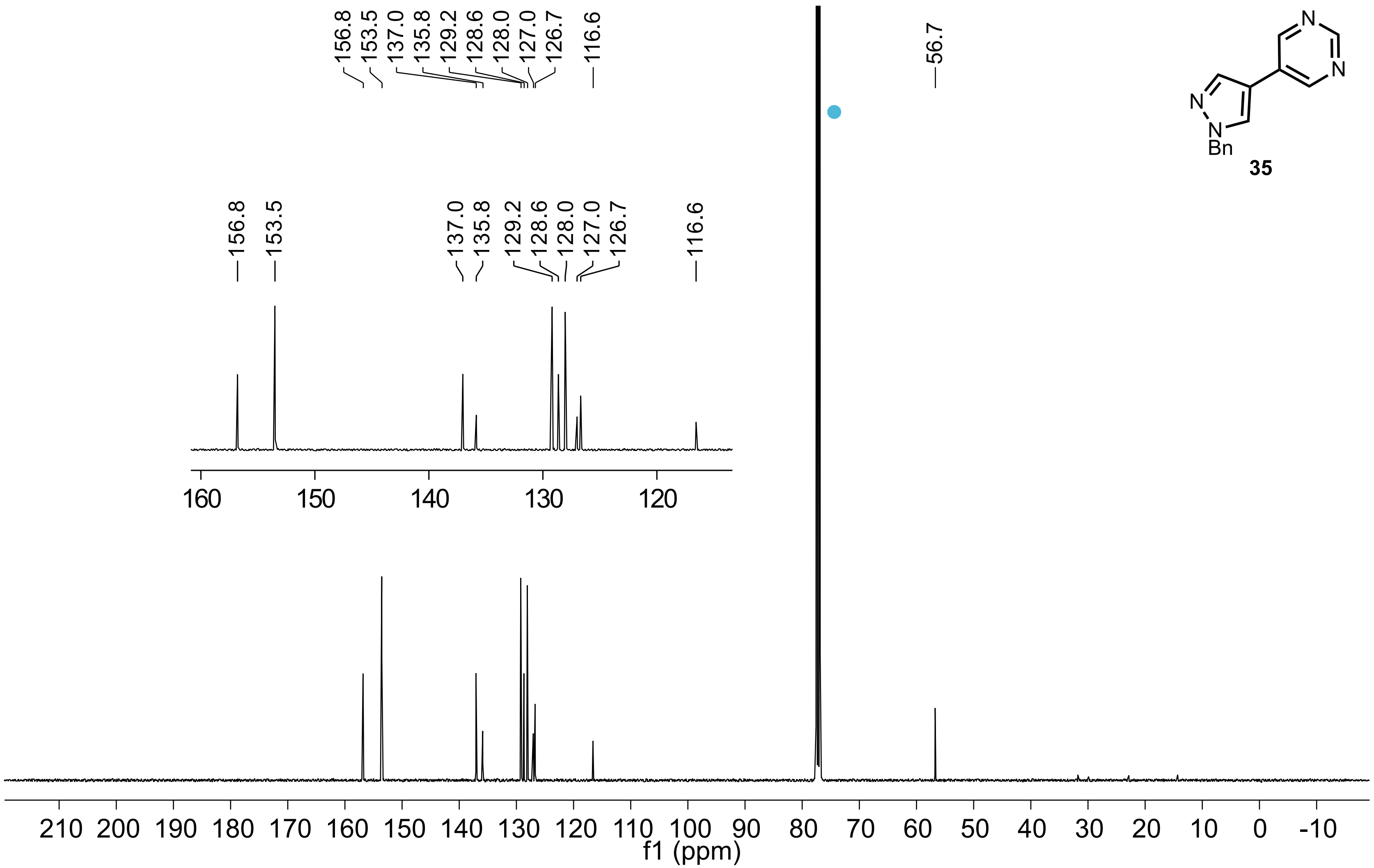

Figure S115 $^{13}C\{^{1}H\}$ NMR (100.67 MHz, $CDCl_3$) spectrum of 5-[1-(phenylmethyl)-1H-pyrazol-4-yl]pyrimidine (**35**). Deuterated solvent (•).

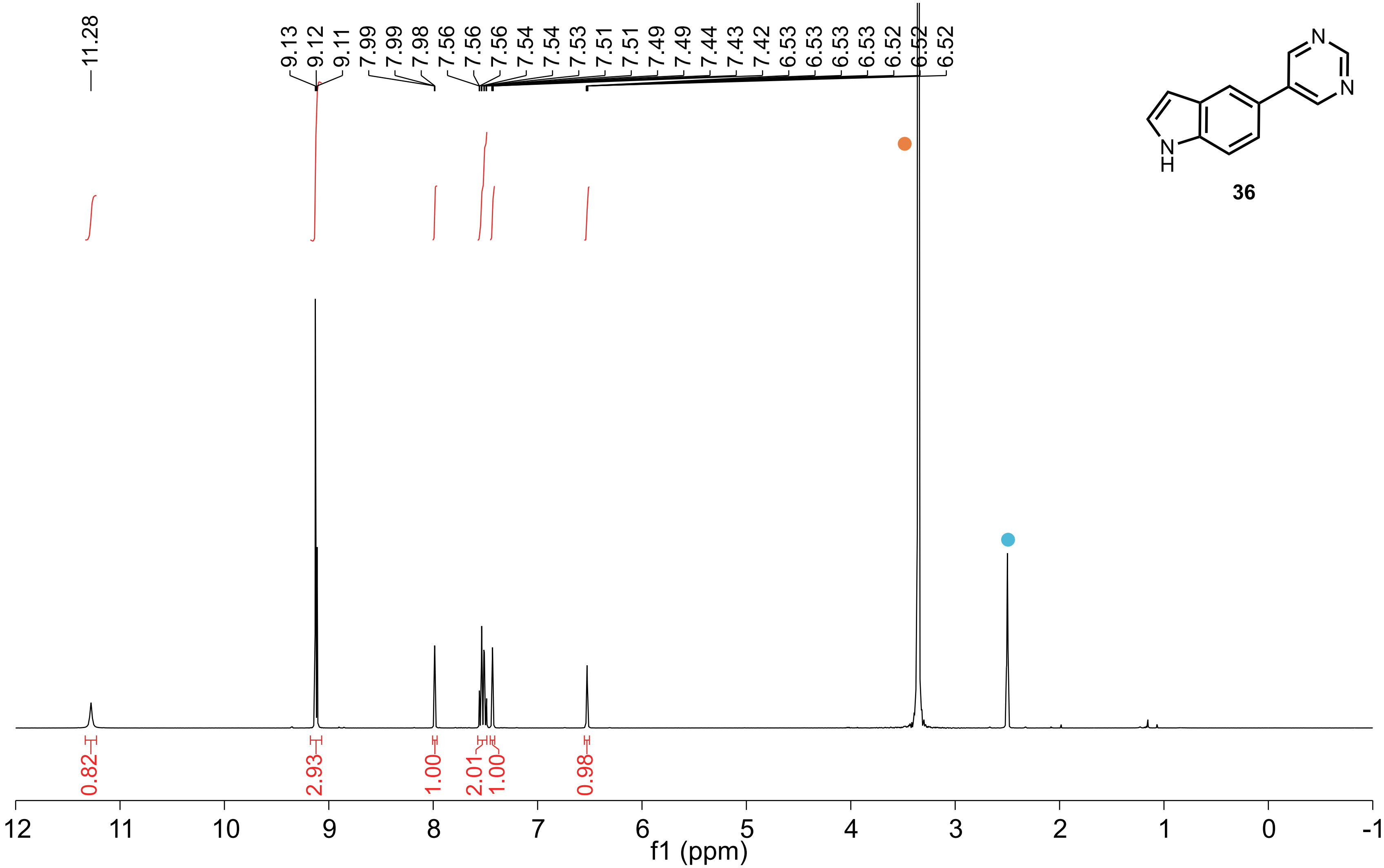

Figure S116 ^{1}H NMR (400.30 MHz, $(CD_3)_2SO$) spectrum of 5-(5-pyrimidinyl)-1H-indole (**36**). Residual proteo-solvent (•) and H_2O (•).

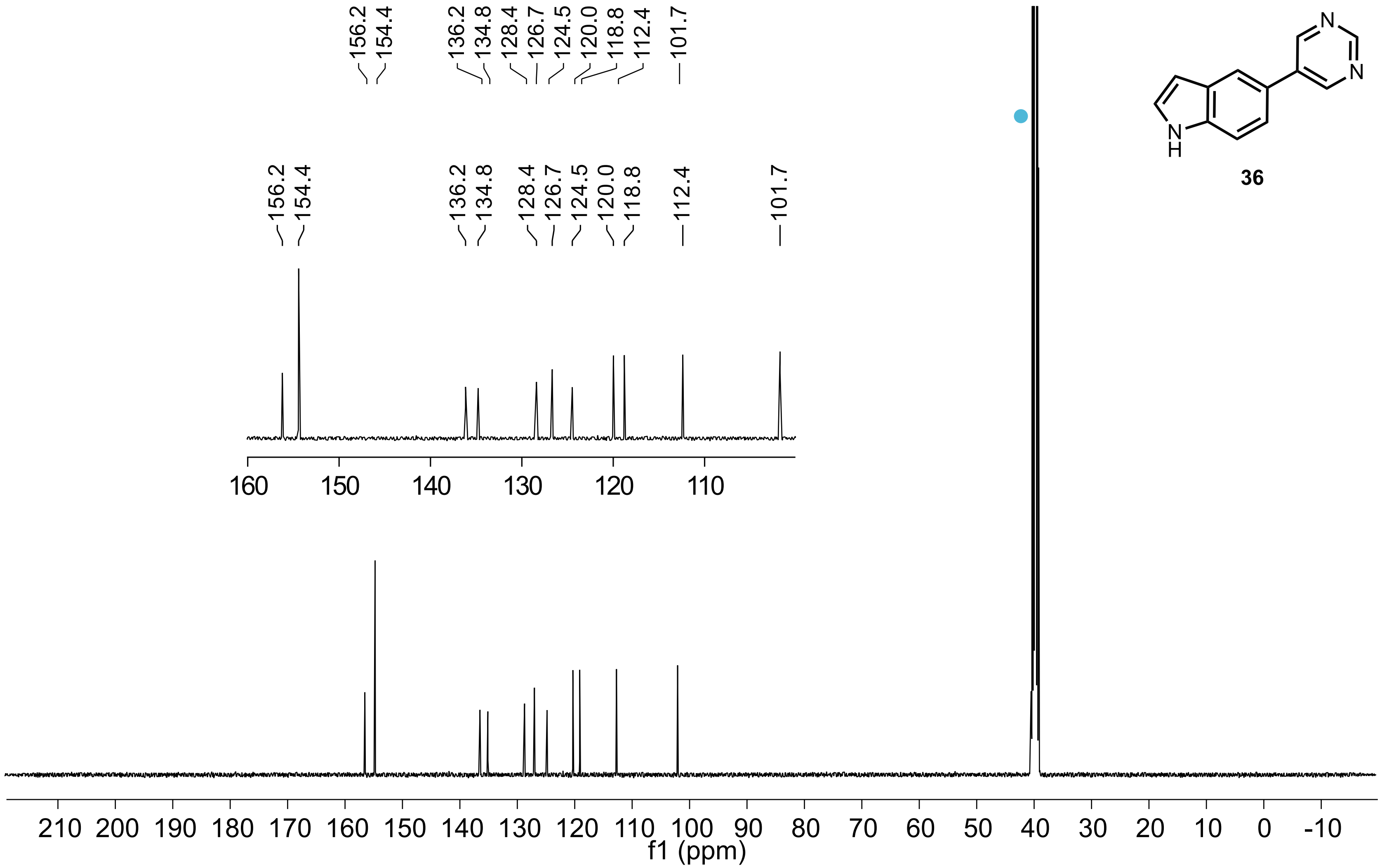

Figure S117 $^{13}C\{^{1}H\}$ NMR (100.67 MHz, $(CD_3)_2SO$) spectrum of 5-(5-pyrimidinyl)-1H-indole (**36**). Deuterated solvent (●).

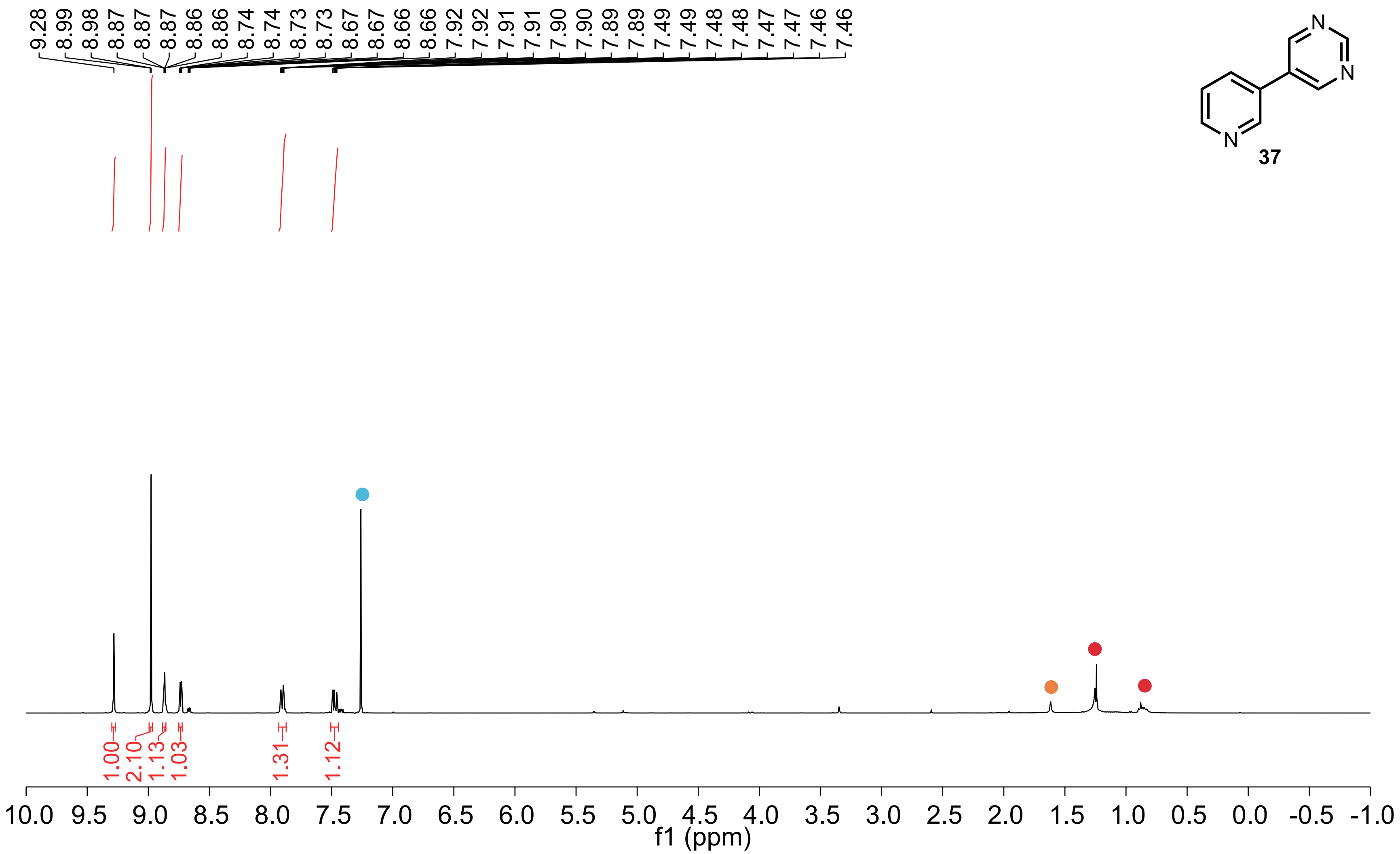

37

Figure S118 ^{1}H NMR (400.30 MHz, $CDCl_3$) spectrum of 5-(3-pyridinyl)pyrimidine (**37**). Residual proteo-solvent (•), H_2O (•), hexane and grease (•).

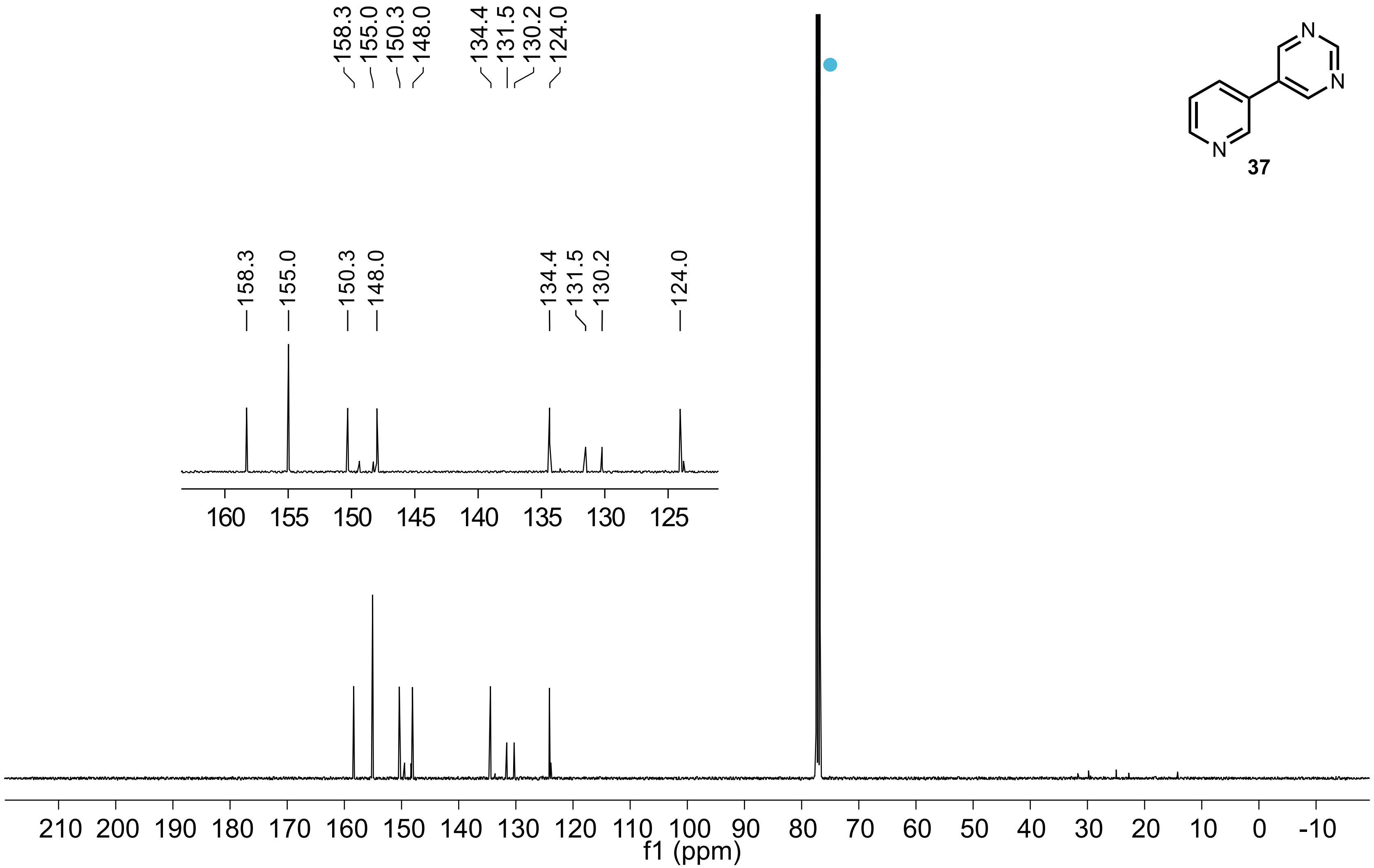

Figure S119 $^{13}C\{^{1}H\}$ NMR (100.67 MHz, $CDCl_3$) spectrum of 5-(3-pyridinyl)pyrimidine (**37**). Deuterated solvent (•).

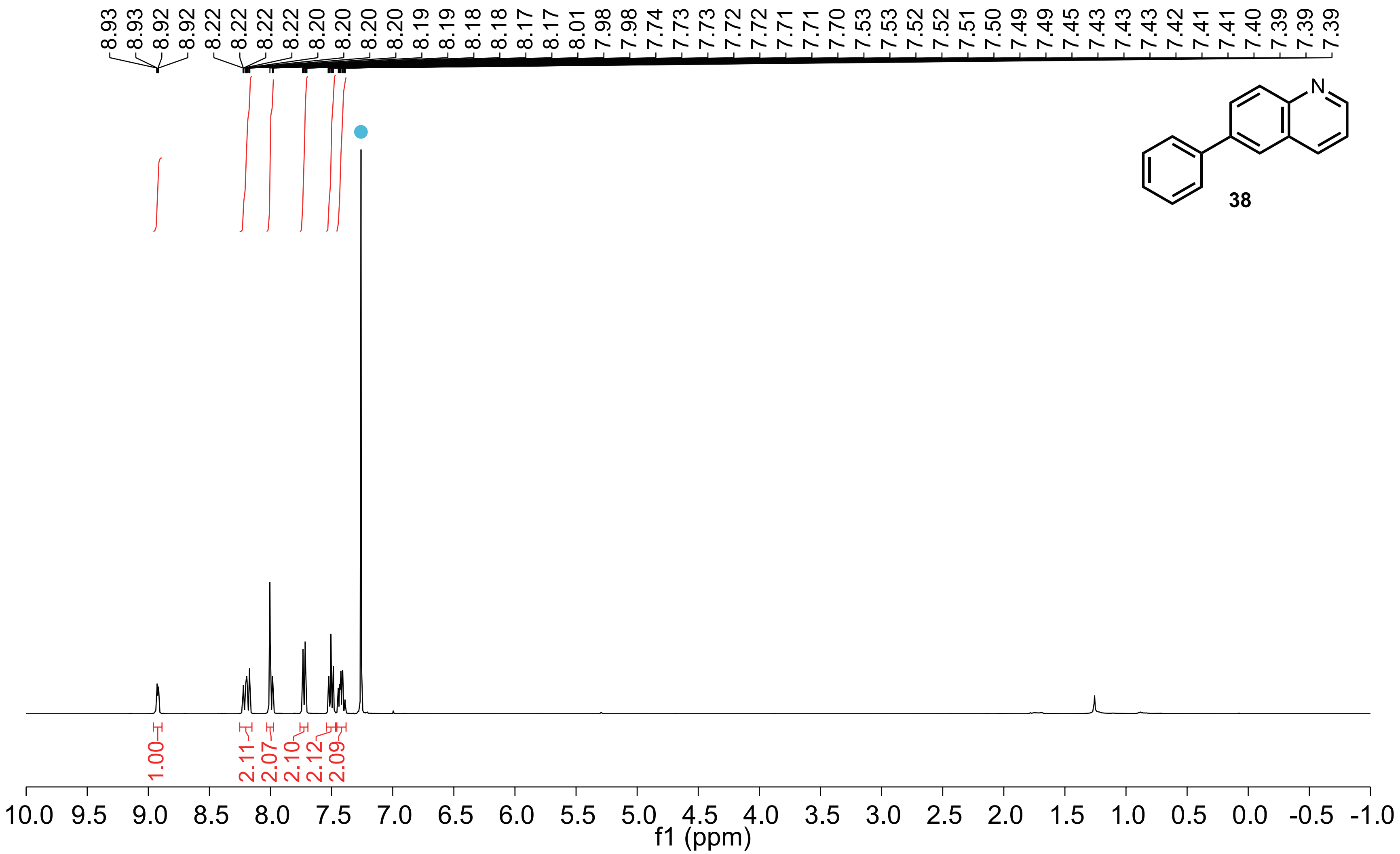

Figure S120 ^{1}H NMR (400.30 MHz, $CDCl_3$) spectrum of 6-phenylquinoline (**38**). Residual proteo-solvent (•).

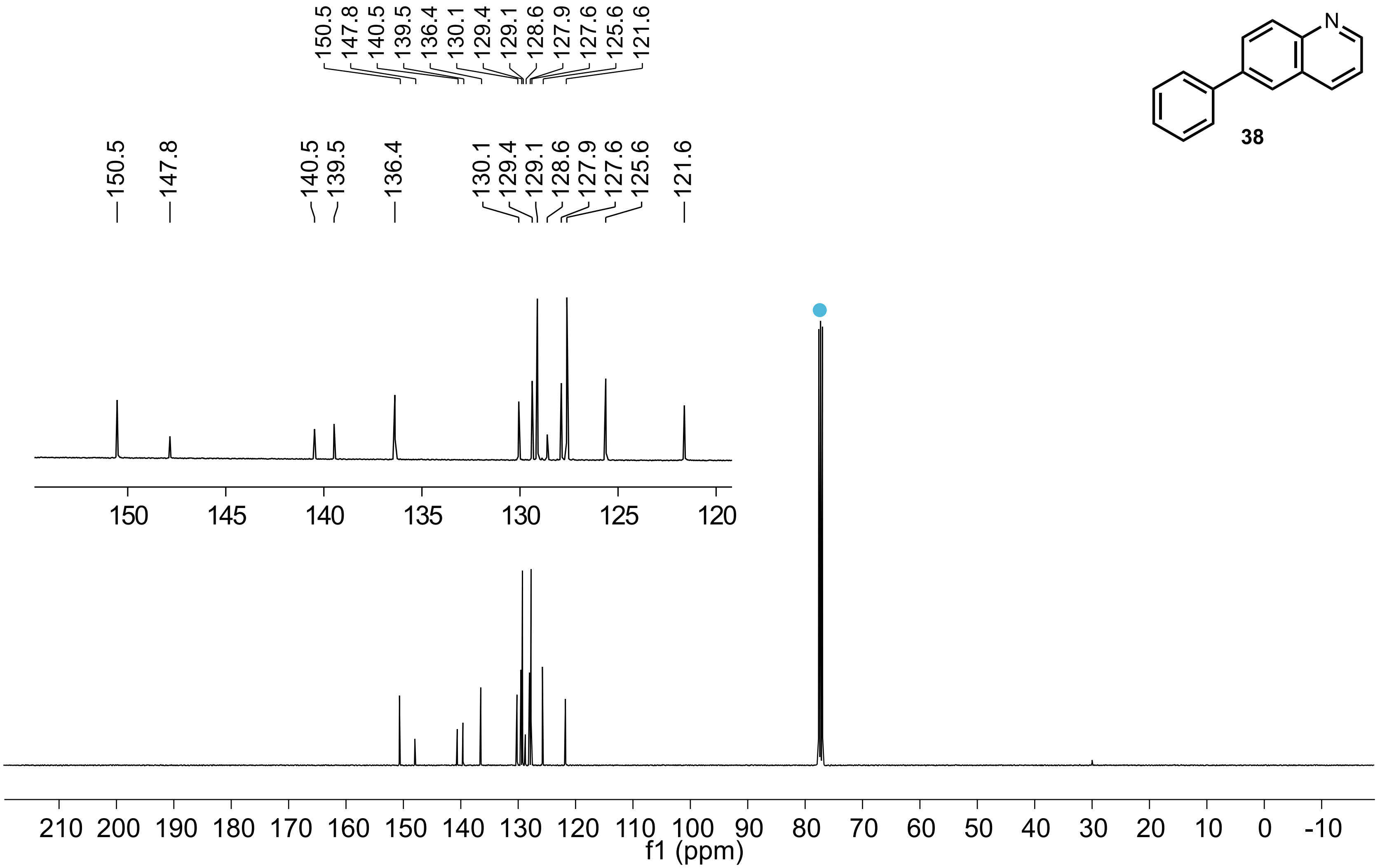

Figure S121 $^{13}C\{^1H\}$ NMR (100.67 MHz, $CDCl_3$) spectrum of 6-phenylquinoline (**38**). Deuterated solvent (•).

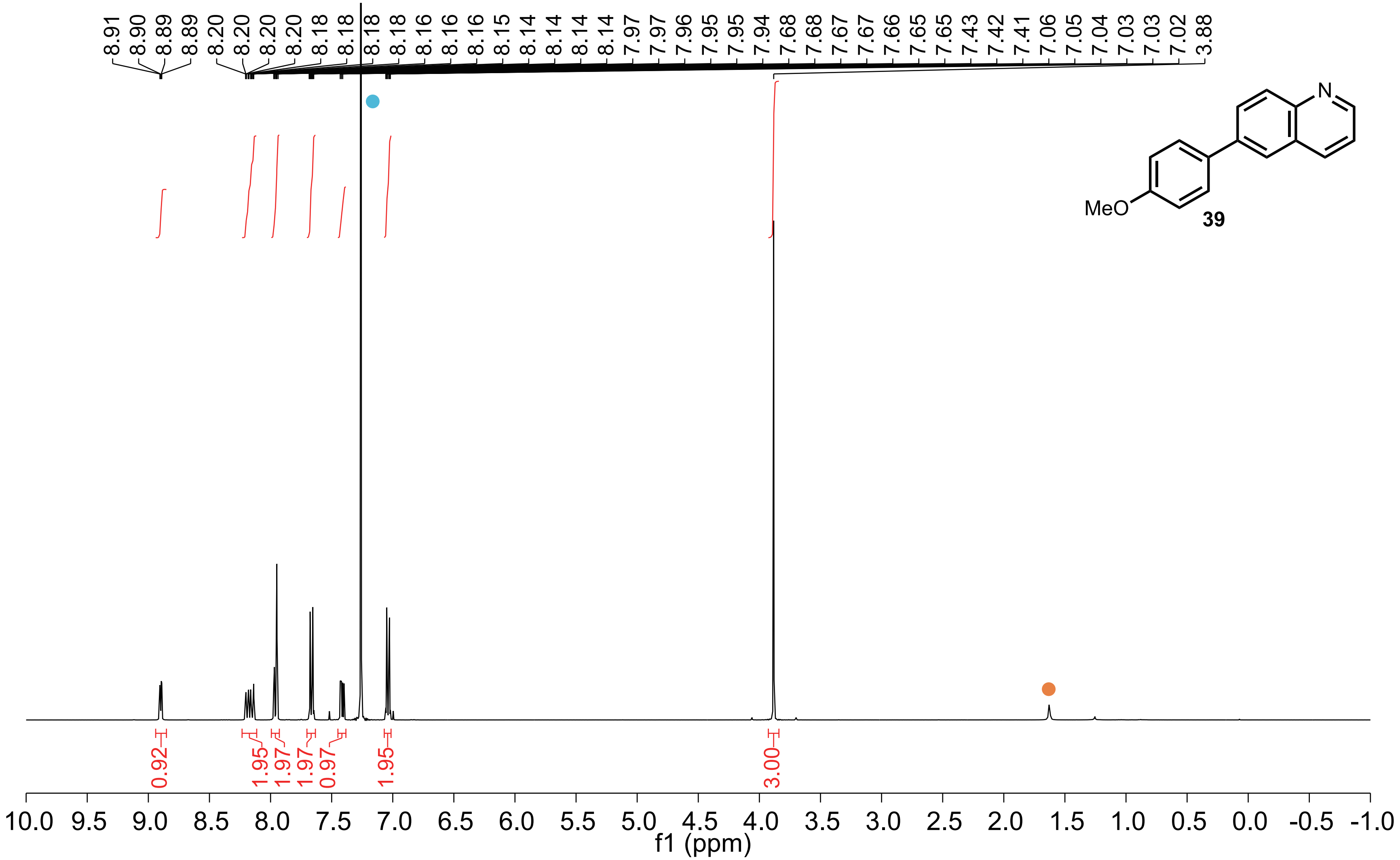

Figure S122 ^{1}H NMR (400.30 MHz, $CDCl_3$) spectrum of 6-(4-methoxyphenyl)quinoline (**39**). Residual proteo-solvent (•) and H_2O (•).

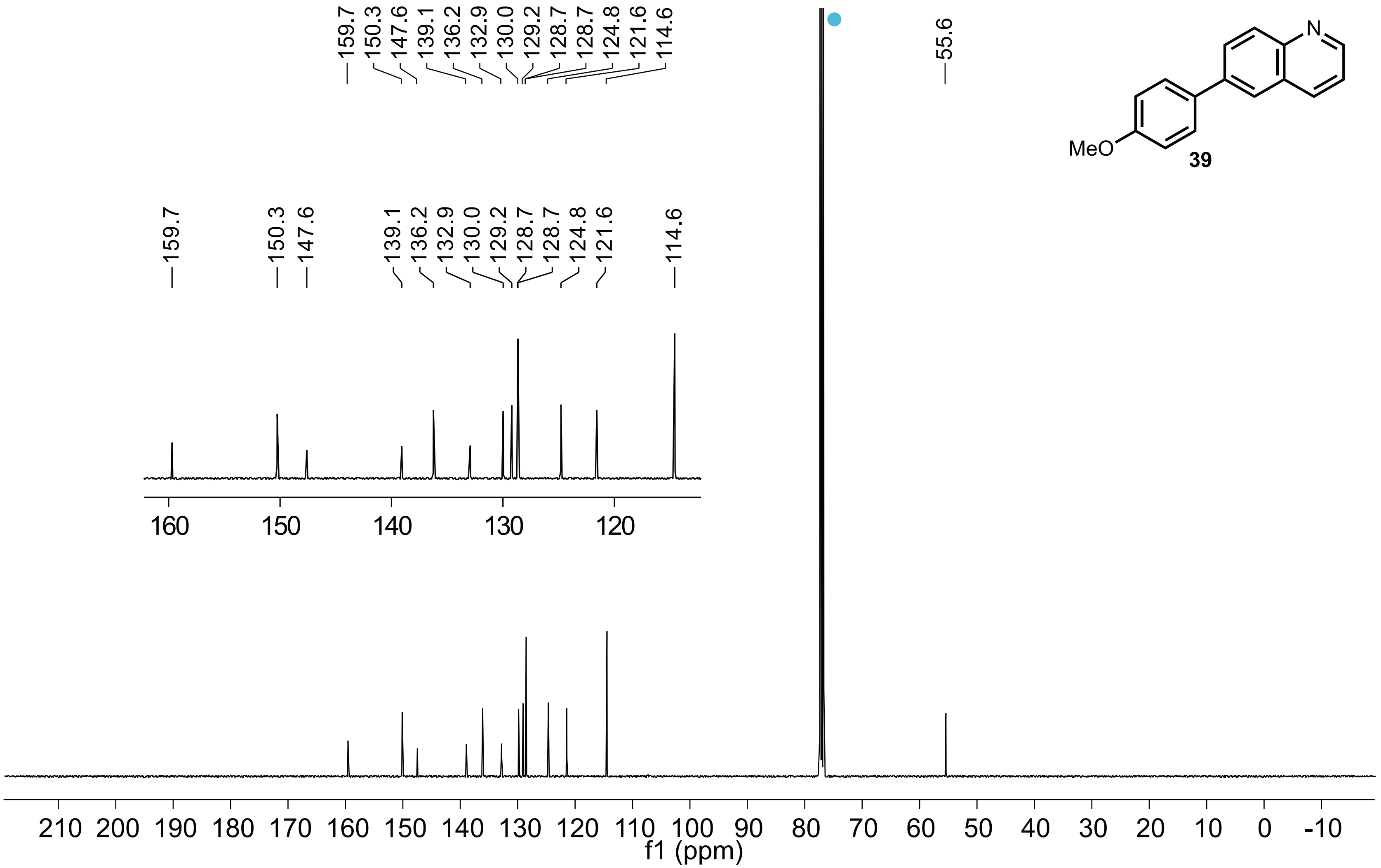

Figure S123 $^{13}C\{^{1}H\}$ NMR (100.67 MHz, $CDCl_3$) spectrum of 6-(4-methoxyphenyl)quinoline (**39**). Deuterated solvent (•).

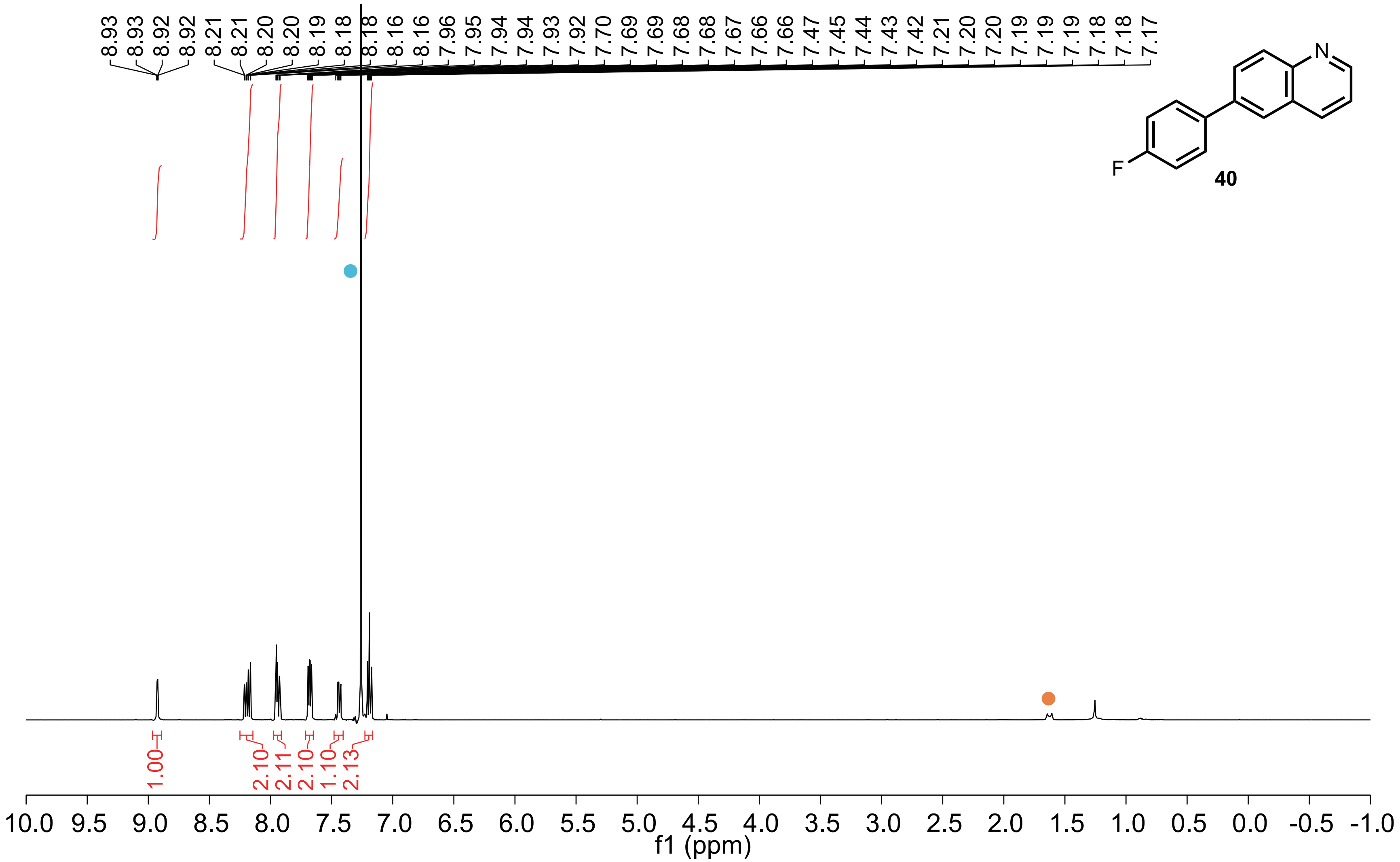

Figure S124 ^{1}H NMR (400.30 MHz, $CDCl_3$) spectrum of 6-(4-fluorophenyl)quinoline (**40**). Residual proteo-solvent (•) and H_2O (•).

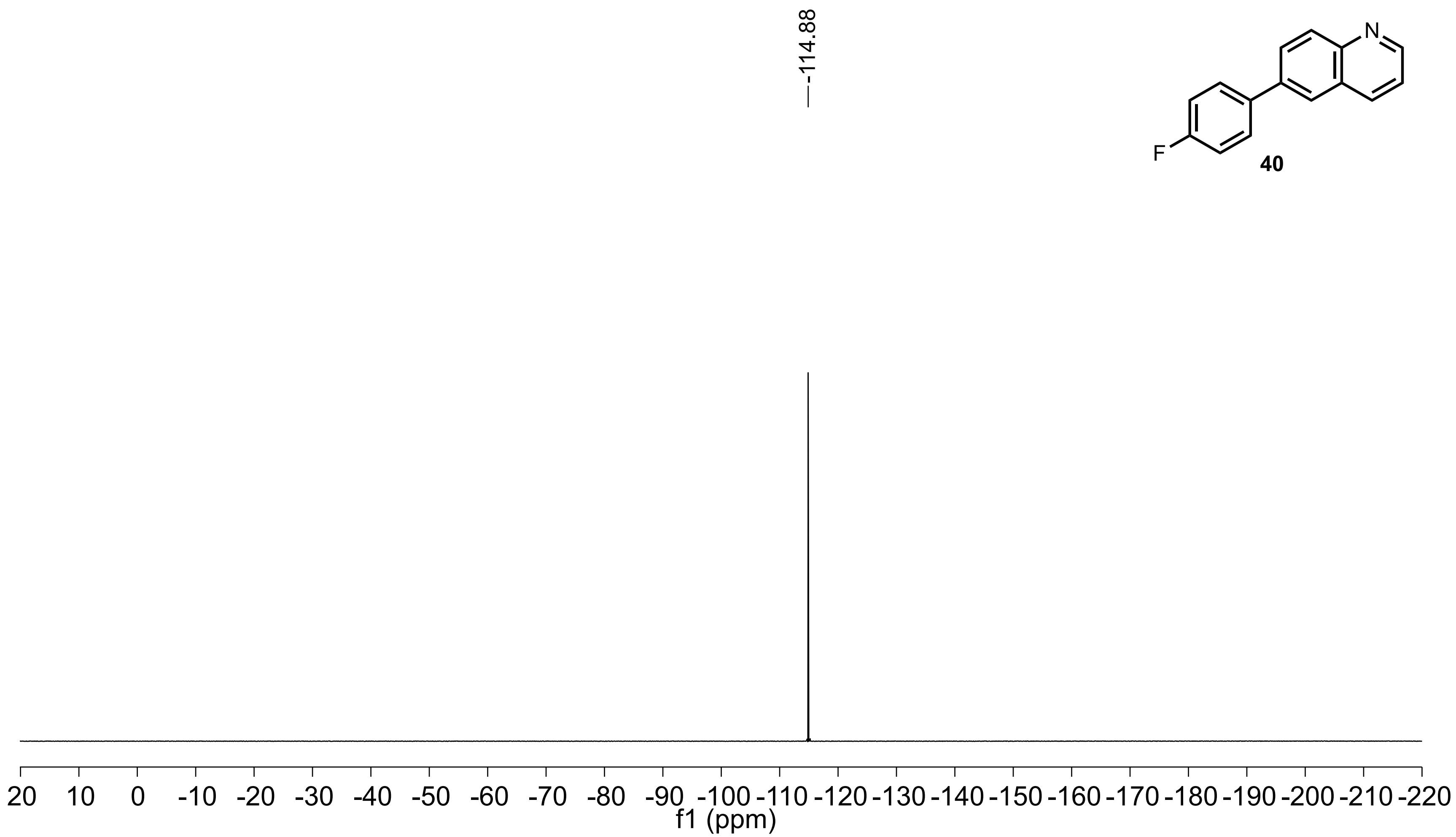

Figure S125 $^{19}F\{^1H\}$ NMR (470.61 MHz, $CDCl_3$) spectrum of 6-(4-fluorophenyl)quinoline (**40**).

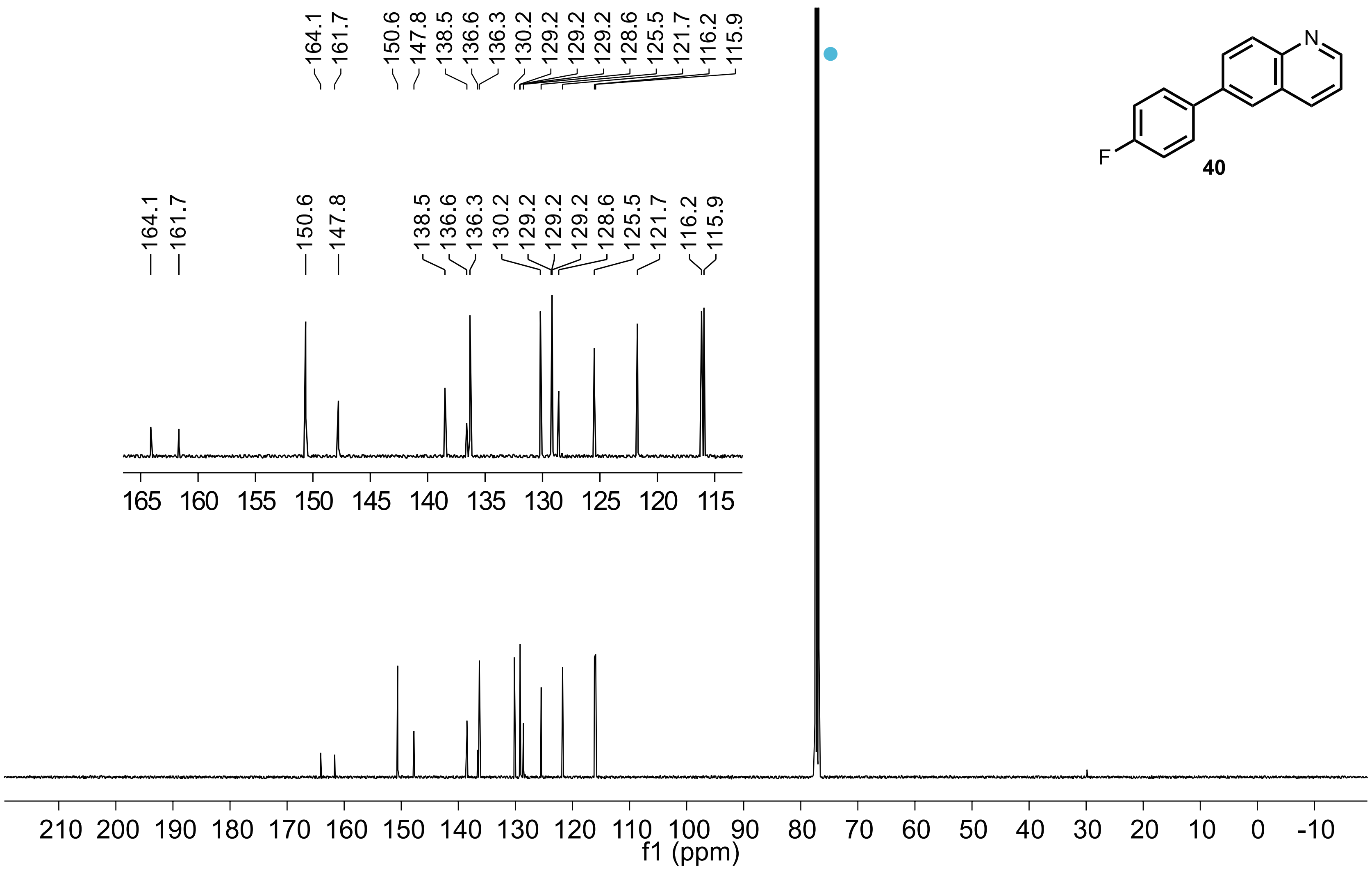

40

Figure S126 $^{13}C\{^{1}H\}$ NMR (100.67 MHz, $CDCl_3$) spectrum of 6-(4-fluorophenyl)quinoline (**40**). Deuterated solvent (•).

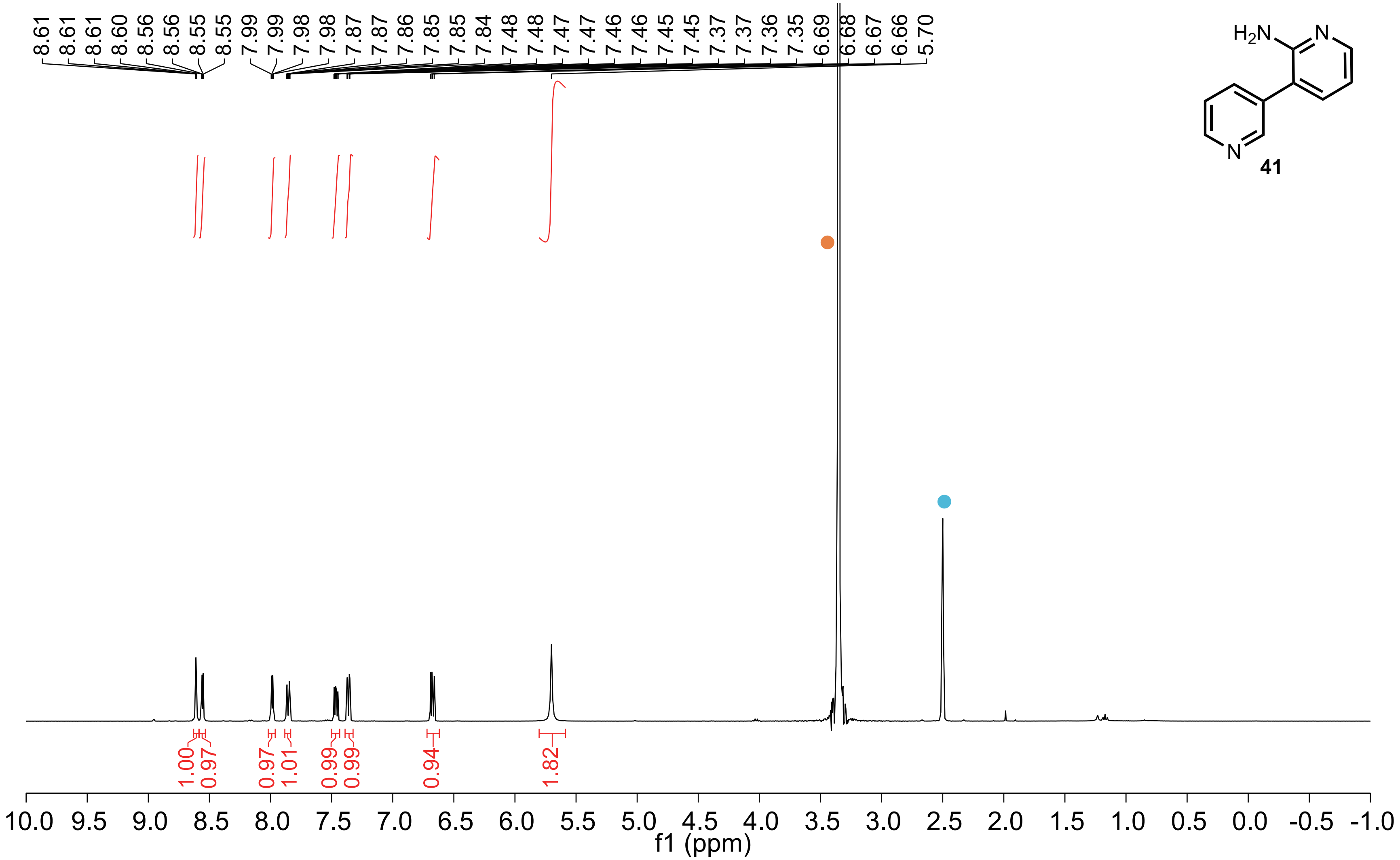

Figure S127 ^{1}H NMR (400.30 MHz, $(CD_3)_2SO$) spectrum of [3,3’]-bipyridinyl-2-ylamine (**41**). Residual proteo-solvent (•) and H_2O (•).

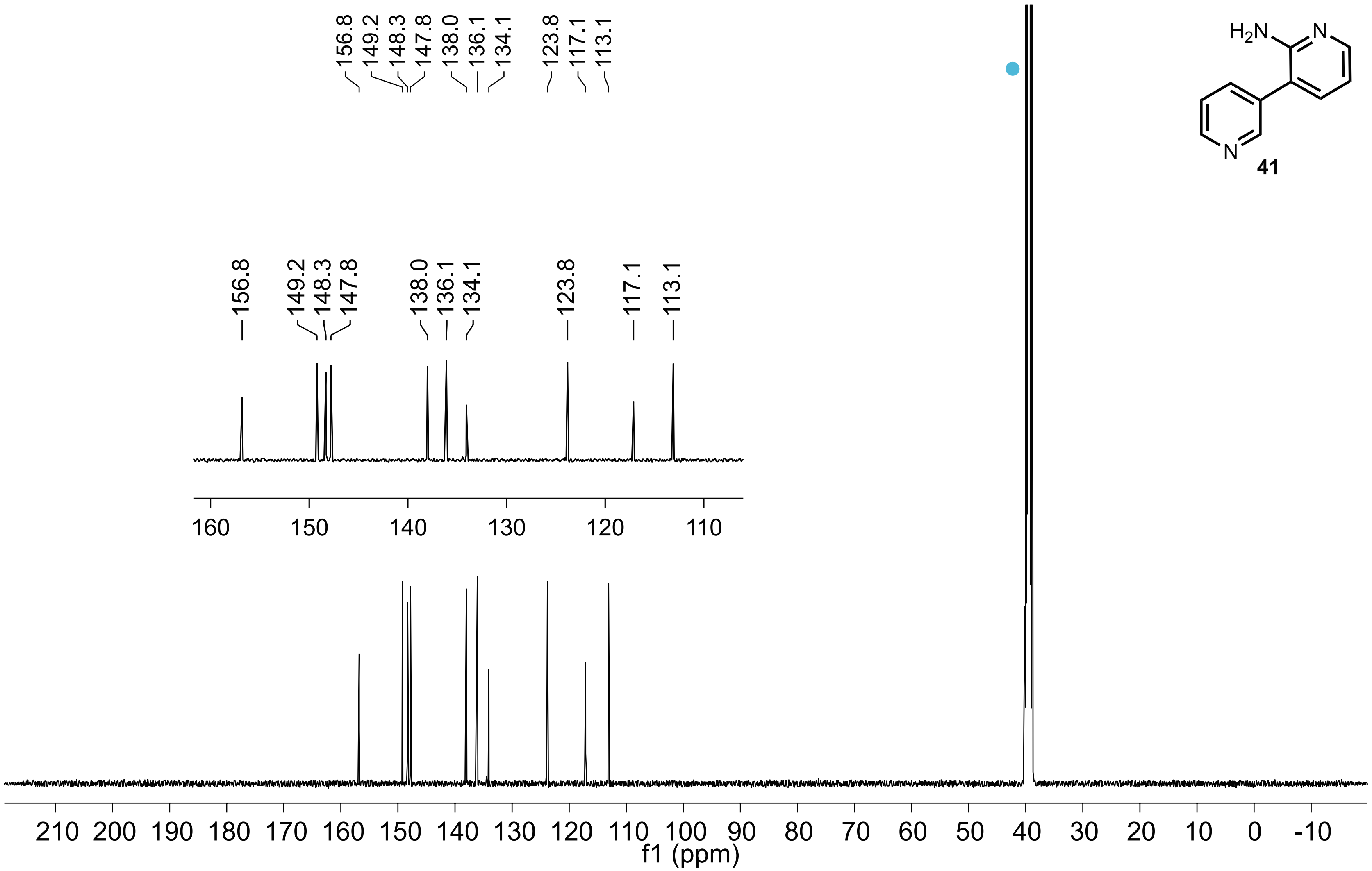

41

Figure S128 $^{13}C\{^{1}H\}$ NMR (100.67 MHz, $(CD_3)_2SO$) spectrum of [3,3’]-bipyridinyl-2-ylamine (**41**). Deuterated solvent (•).

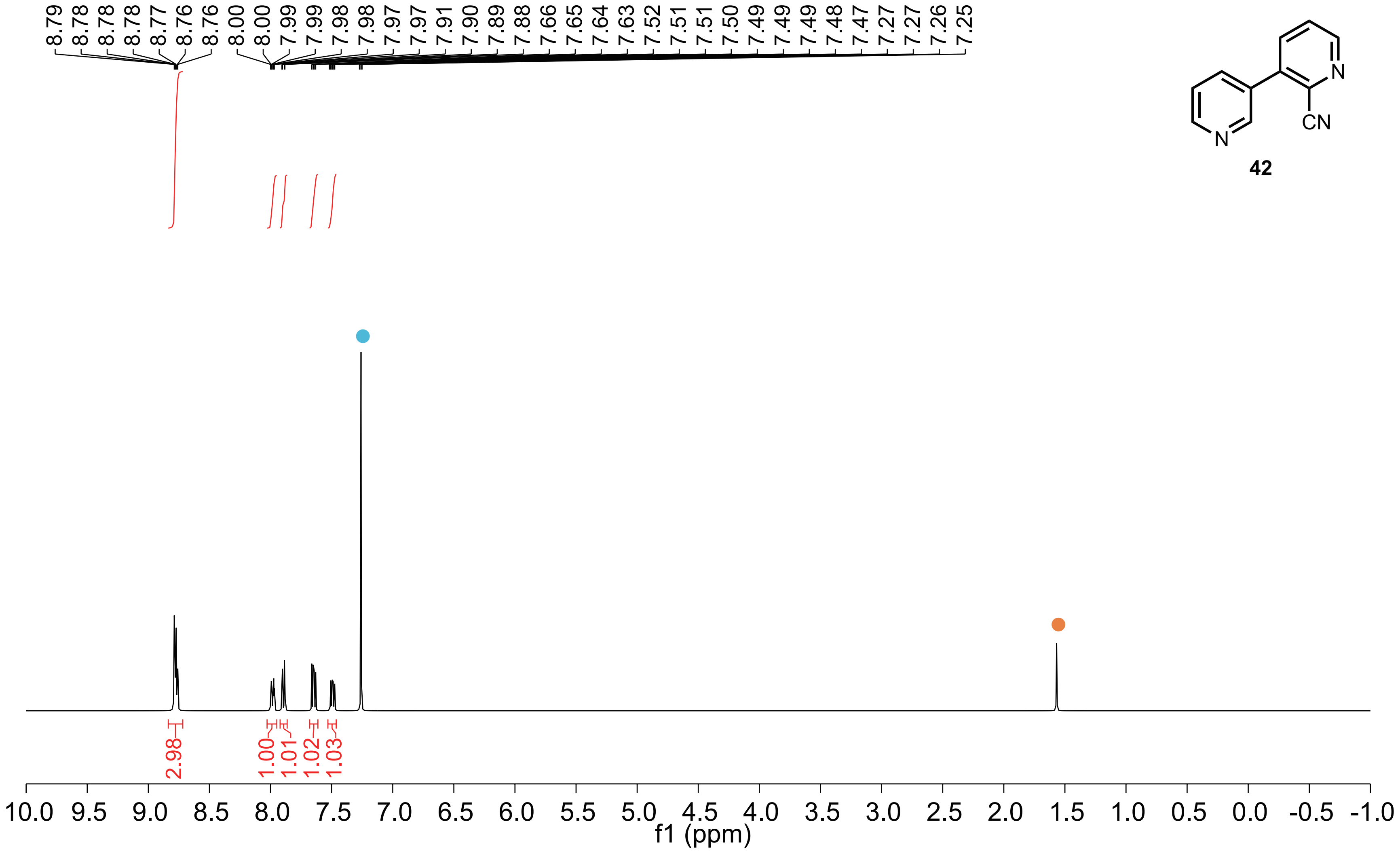

42

Figure S129 ^{1}H NMR (400.30 MHz, $CDCl_3$) spectrum of [3,3′-bipyridine]-2-carbonitrile (**42**). Residual proteo-solvent (•) and H_2O (•)

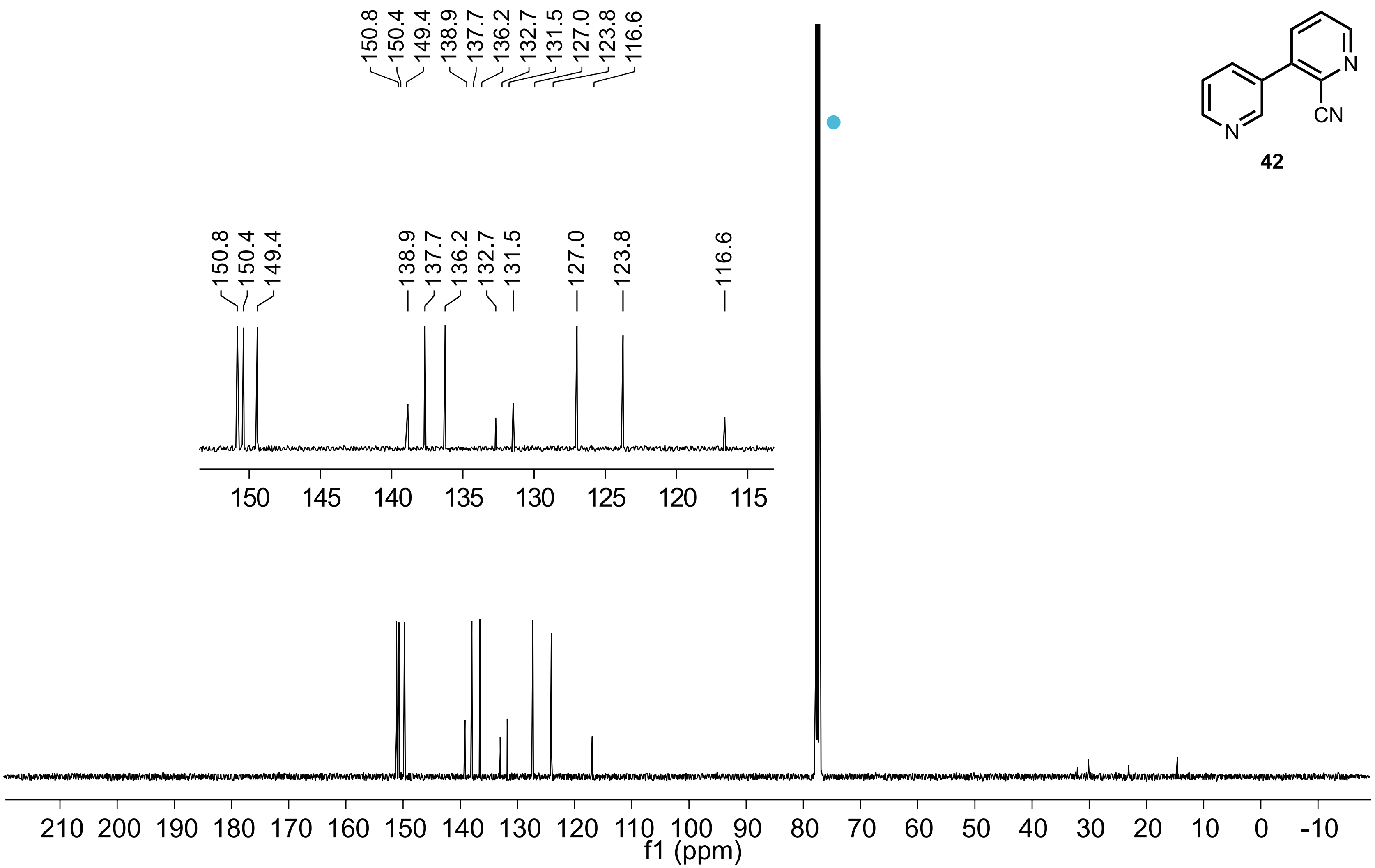

Figure S130 $^{13}C\{^{1}H\}$ NMR (100.67 MHz, $CDCl_3$) spectrum of [3,3′-bipyridine]-2-carbonitrile (**42**). Deuterated solvent (•).

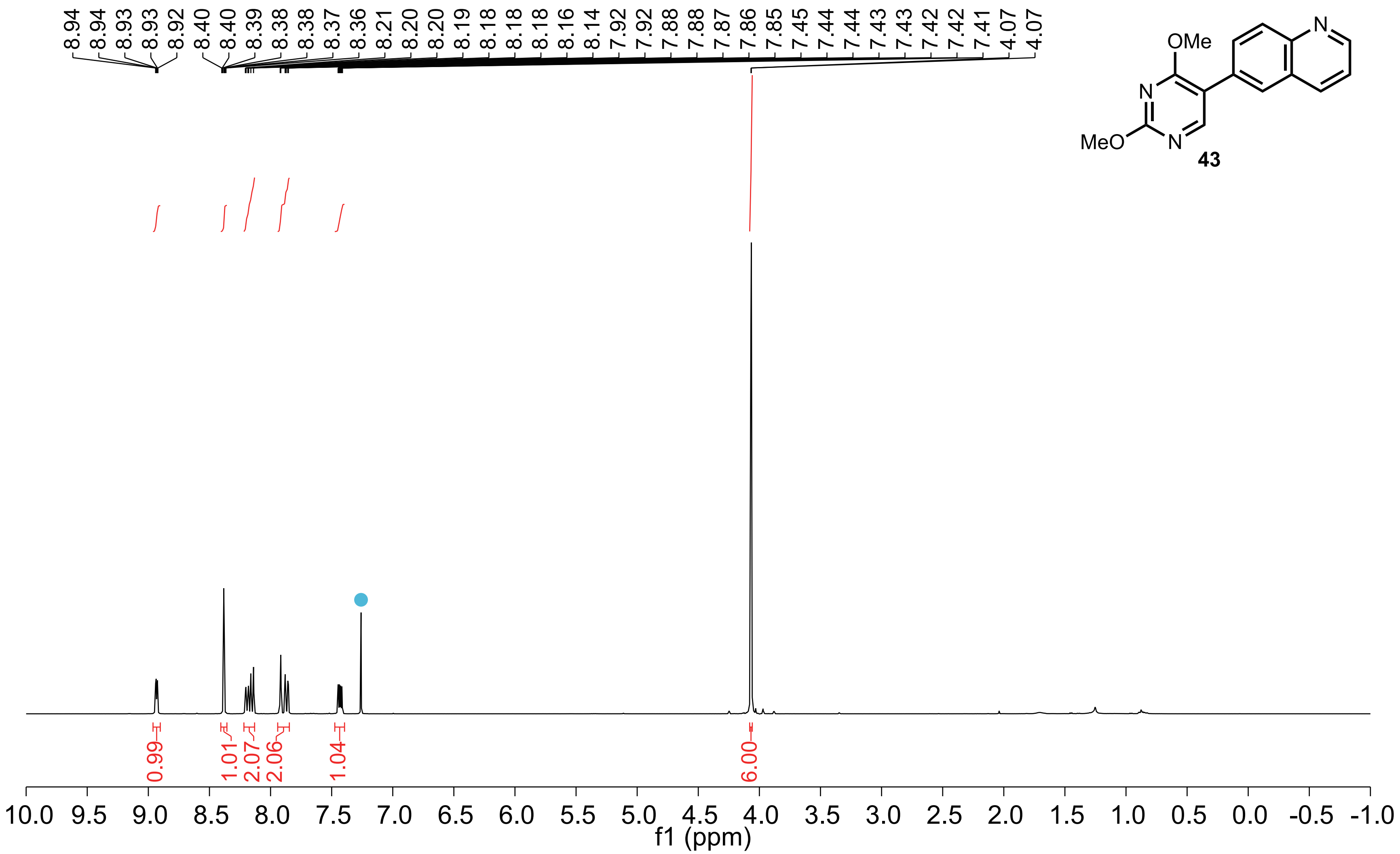

Figure S131 ^{1}H NMR (400.30 MHz, $CDCl_3$) spectrum of 6-(2,4-dimethoxy-5-pyrimidinyl)quinoline (**43**). Residual proteo-solvent (•).

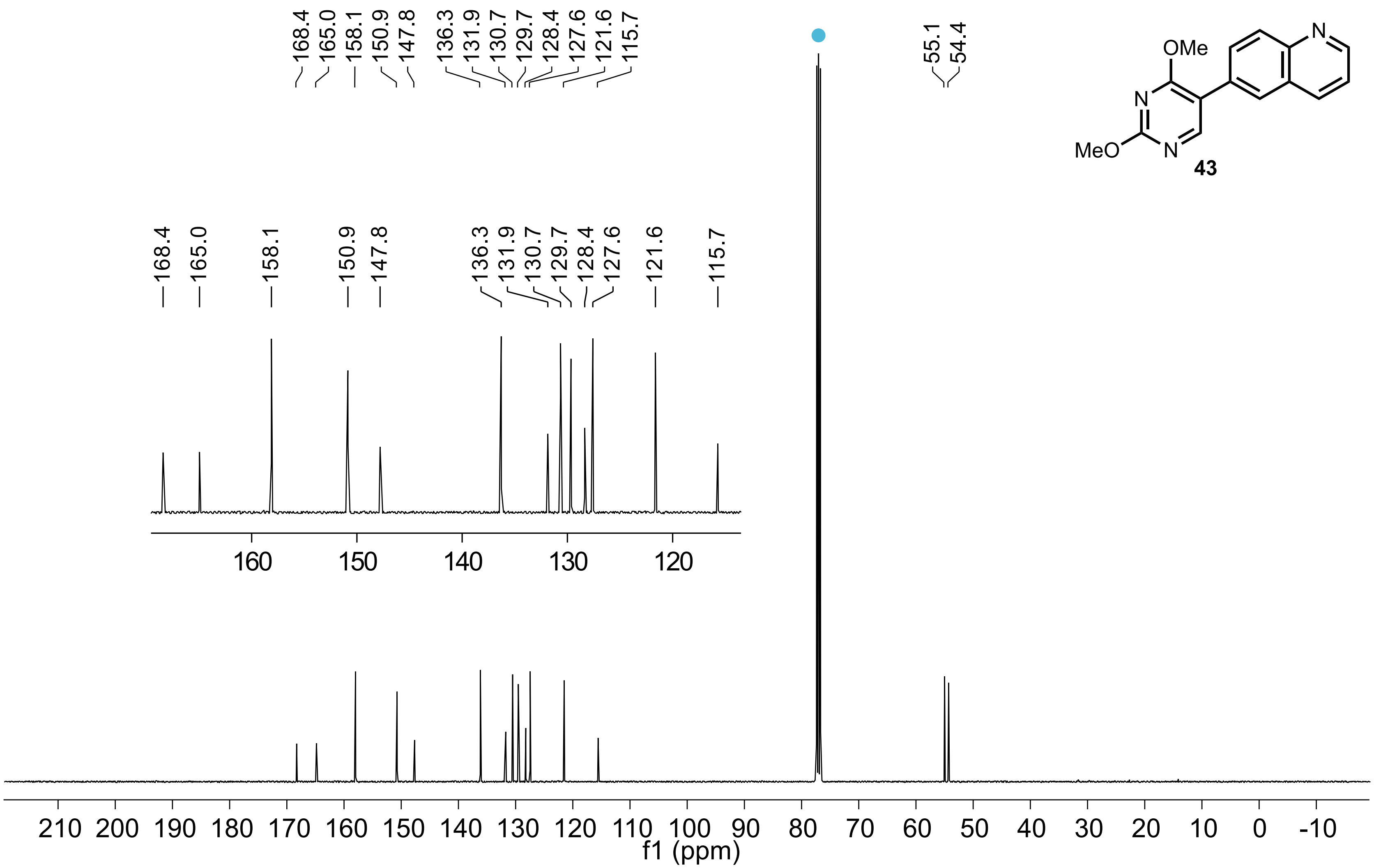

Figure S132 $^{13}C\{^{1}H\}$ NMR (100.67 MHz, $CDCl_3$) spectrum of 6-(2,4-dimethoxy-5-pyrimidinyl)quinoline (**43**). Deuterated solvent (•).

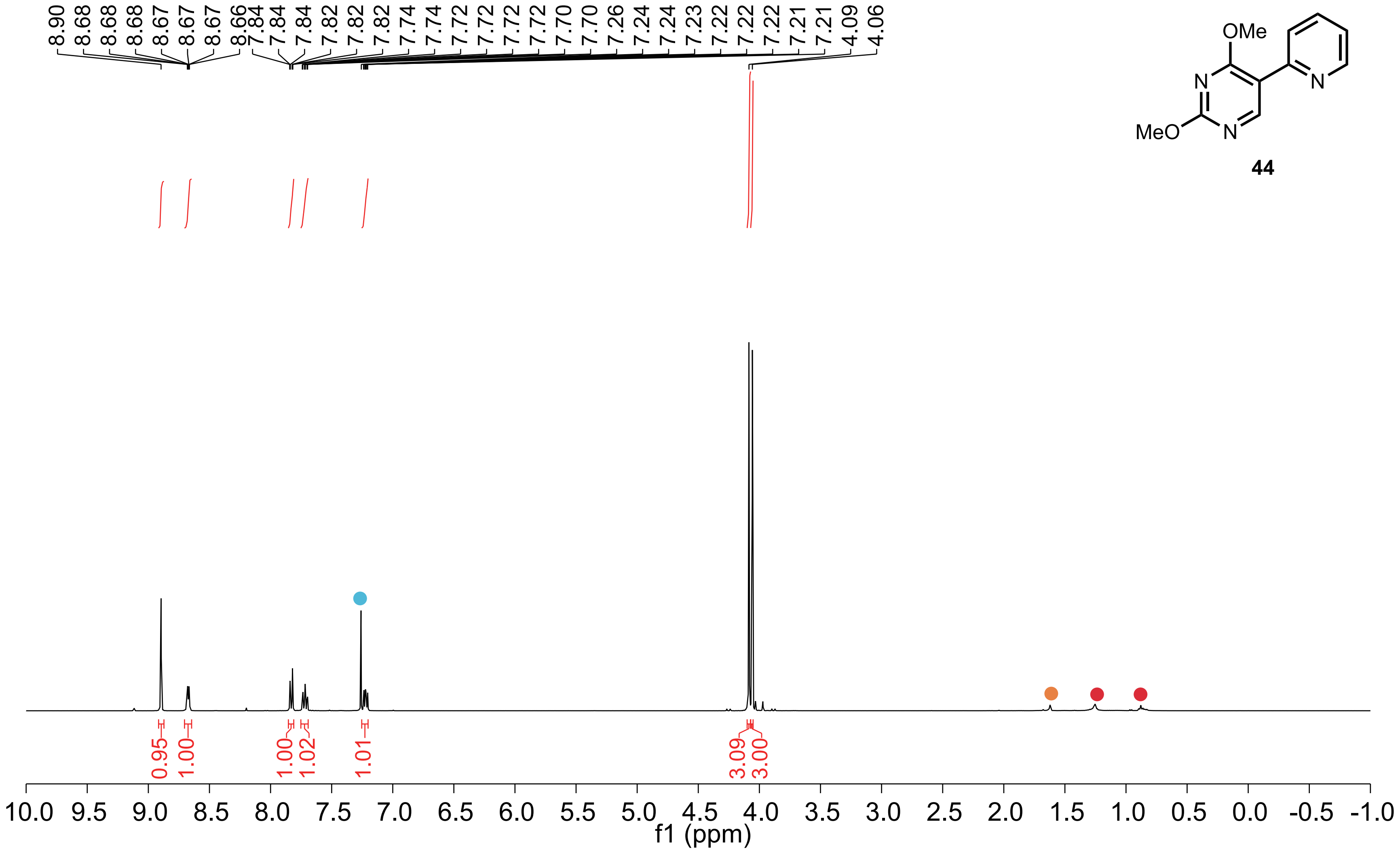

Figure S133 ^{1}H NMR (400.30 MHz, $CDCl_3$) spectrum of 2,4-dimethoxy-5-(2-pyridinyl)pyrimidine (**44**). Residual proteo-solvent (•), H_2O (•) and hexane (•).

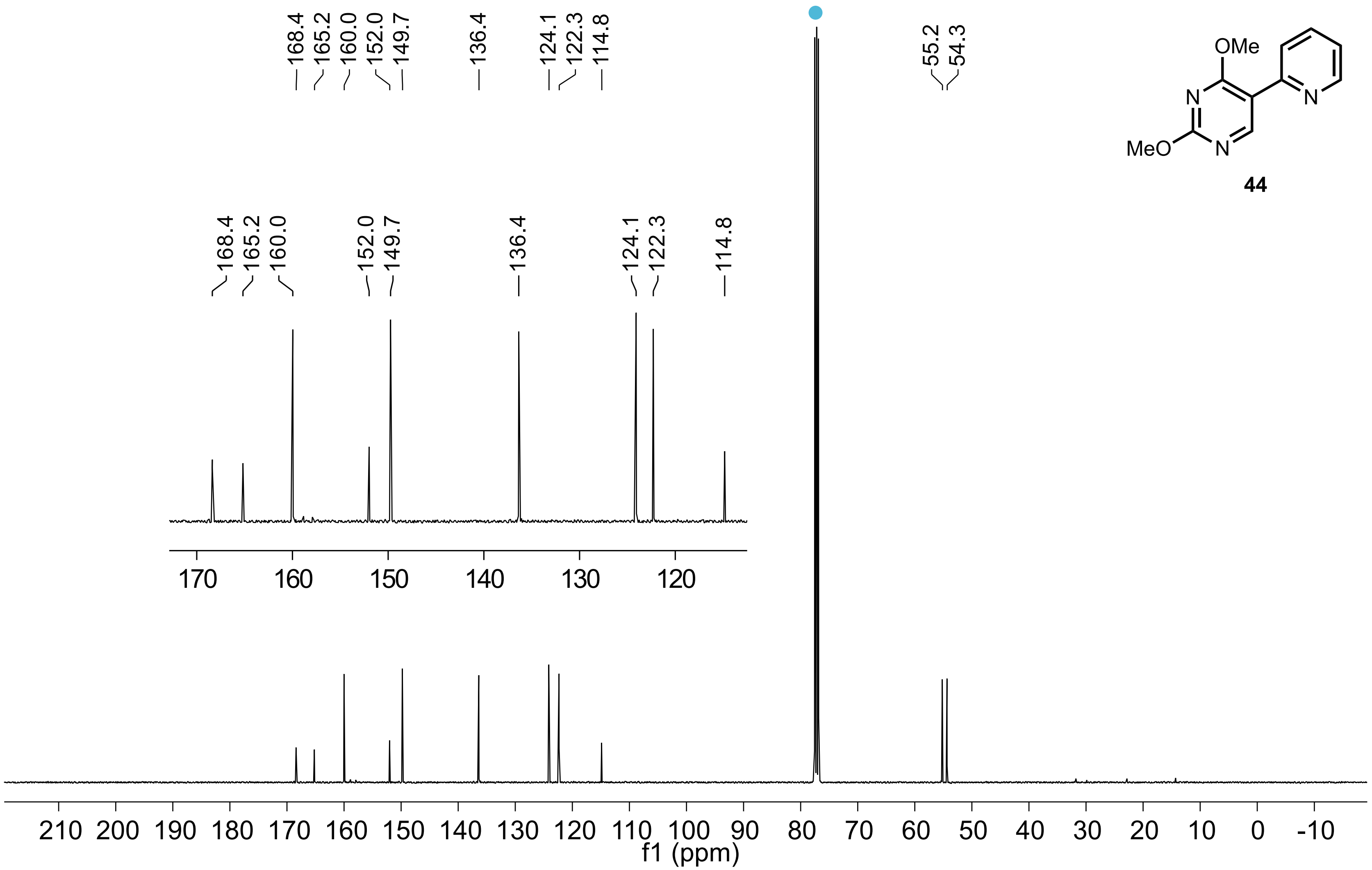

Figure S134 $^{13}C\{^{1}H\}$ NMR (100.67 MHz, $CDCl_3$) spectrum of 2,4-Dimethoxy-5-(2-pyridinyl)pyrimidine (**44**). Deuterated solvent (•).

7. Crystallographic data

X-ray diffraction data were collected on a Bruker D8 VENTURE diffractometer using Cu Kα radiation (for **11** and **17**) or Mo Kα radiation (**13**). Crystal data, data collection and refinement parameters are summarized in Table S6. The structures were solved using a dual-space method and standard difference map techniques, and were refined by full-matrix least-squares procedures on F^2 with SHELXTL (Version 2019/1).[32,33] All hydrogen atoms bound to carbon were placed in calculated positions and refined with a riding model [U_{iso}(H) = 1.2–1.5U_{eq}(C)] (For **11**, hydrogen atoms bound to oxygen were located on the difference map and refined with a riding model [U_{iso}(H) = 1.2U_{eq}(O)]).

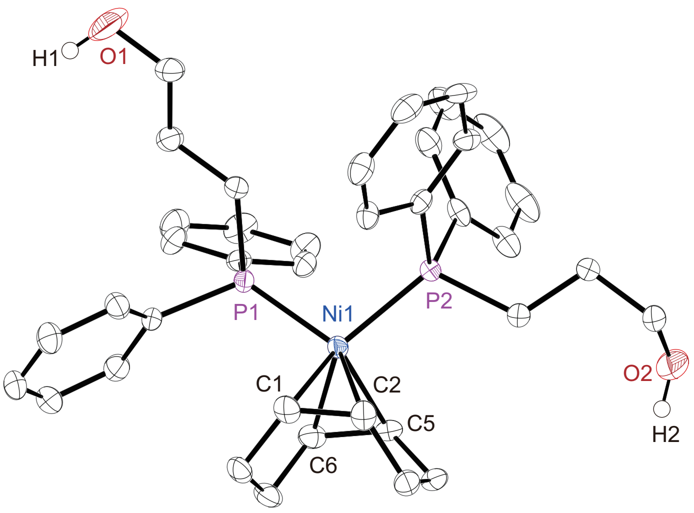

Figure S135 Molecular structure of Ni($PPh_2CH_2CH_2CH_2OH)_2$(cod) (**11**). The thermal ellipsoids are shown at 50% probability. Hydrogen atoms at carbons and THF have been omitted for clarity. Selected distances (Å) and bond angles (°): Ni1-C1 2.106(4), Ni1-C2 2.093(4); Ni1-C5 2.100(4); Ni1-C6 2.107(4); Ni1-P2 2.1679(10); Ni1-P1 2.1811(10); C1-C2 1.392(5); O1-H1 0.8400; O2-H2 0.8400; P2-Ni1-P1 106.48(4).

---

[32] Sheldrick, G. M. Crystal structure refinement with SHELXL. *Acta. Cryst.* **2015**, *C71*, 3–8.
[33] Sheldrick, G. M. SHELXTL, An Integrated System for Solving, Refining, and Displaying Crystal Structures from Diffraction Data; University of Göttingen, Göttingen, Federal Republic of Germany,1981.

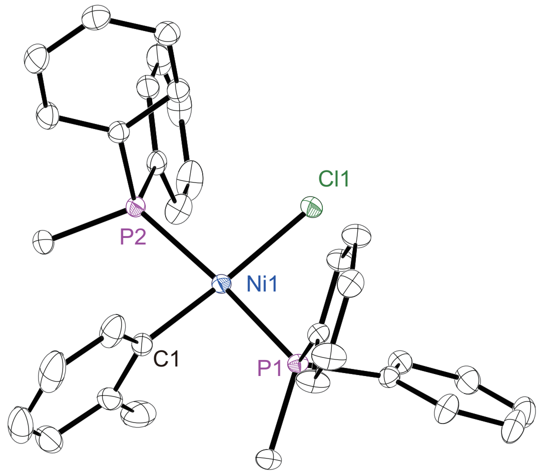

Figure S136 Molecular structure of $Ni(PPh_2Me)_2Cl$(*o*-Tol) (**13**). The thermal ellipsoids are shown at 50% probability. Hydrogen atoms at carbons have been omitted for clarity. Selected distances (Å) and bond angles (°): Ni1-C1 1.8967(14); Ni1-P2 2.1998(4); Ni1-P1 2.2035(4); Ni1-Cl1 2.2221(4); C1-Ni1-P2 86.21(4); C1-Ni1-P1 87.89(4); P2-Ni1-P1 172.495(16); C1-Ni1-Cl1 176.38(5); P2-Ni1-Cl1 91.760(14); P1-Ni1-Cl1 93.882(14).

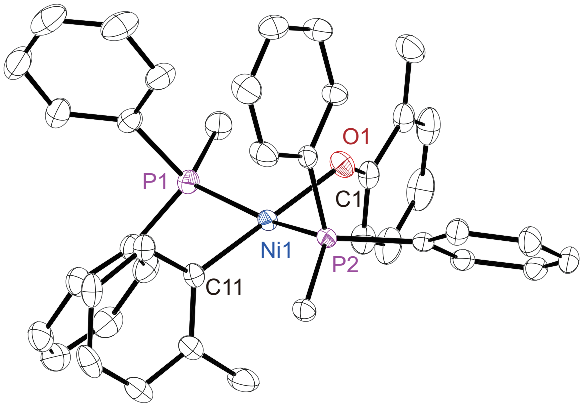

Figure S137 Molecular structure of $Ni(PPh_2Me)_2$(O-*o*-Tol)(*o*-Tol) (**17**). The thermal ellipsoids are shown at 50% probability. Hydrogen atoms at carbons have been omitted for clarity. Selected distances (Å) and bond angles (°): Ni1-O1 1.8928(12); Ni1-C11 1.9086(16); Ni1-P1 2.1894(5); Ni1-P2 2.2055(5); O1-C1 1.321(2); O1-Ni1-C11 176.97(6); O1-Ni1-P1 90.80(4); C11-Ni1-P1 86.47(5); O1-Ni1-P2 94.73(4); C11-Ni1-P2 87.50(5); P1-Ni1-P2 162.06(2); C1-O1-Ni1 122.39(11).

Table S6 Crystal, Intensity Collection, and Refinement Data for Complexes **11**, **13** and **17**.

Complex	**11•2(THF)**	**13**	**17**
lattice	Monoclinic	Orthorhombic	Monoclinic
formula	$C_{46}H_{62}NiO_4P_2$	$C_{33}H_{33}ClNiP_2$	$C_{40}H_{40}NiOP_2$
formula weight	799.60	585.69	657.37
space group	$P2_1/n$	$P2_12_12_1$	$P2_1/n$
a/Å	10.1990(4)	9.8306(7)	12.8908(5)
b/Å	23.3762(8)	14.8861(10)	19.7414(8)
c/Å	17.5064(6)	19.7166(11)	13.5144(5)
α/°	90	90	90
β/°	92.979(2)	90	95.397(2)
γ/°	90	90	90
V/Å^3	4168.1(3)	2885.3(3)	3423.9(2)
Z	4	4	4
temperature (K)	130(2)	130(2)	130(2)
radiation (λ, Å)	1.54178	0.71073	1.54178
ρ (calcd.) g cm^{-3}	1.274	1.348	1.275
μ mm^{-1}	1.719	0.897	1.921
θ max, deg.	74.996	32.640	74.561
no. of data	88116	103588	71561
no. of data	8460	10524	6950
no. of parameters	572	337	401
R_1 [$I > 2\sigma(I)$]	0.0751	0.0239	0.0480
wR_2 [$I > 2\sigma(I)$]	0.1559	0.0603	0.1262
R_1 [all data]	0.0943	0.0247	0.0518
wR_2 [all data]	0.1663	0.0609	0.1302
GOF	1.145	1.079	1.133
R_{int}	0.1238	0.0524	0.0638
